# DFT Visualization and Experimental Evidence of BHT-Mg-Catalyzed Copolymerization of Lactides, Lactones and Ethylene Phosphates

**DOI:** 10.3390/polym11101641

**Published:** 2019-10-10

**Authors:** Ilya Nifant’ev, Andrey Shlyakhtin, Maxim Kosarev, Dmitry Gavrilov, Stanislav Karchevsky, Pavel Ivchenko

**Affiliations:** 1 Chemistry Department, M.V. Lomonosov Moscow State University, 1–3 Leninskie Gory, 119991 Moscow, Russia; shlyahtinav@mail.ru (A.S.); komrad.kosarev.maksim@gmail.com (M.K.); gavrosdm@gmail.com (D.G.); phpasha1@yandex.ru (P.I.); 2 A.V. Topchiev Institute of Petrochemical Synthesis RAS, 29 Leninsky Pr., 119991 Moscow, Russia; 3 Joint-stock company “Institute of petroleum refining and petrochemistry”, 12 Iniciativnaya Str., 450065 Ufa, Republic of Bashkortostan, Russia; st_karchevsky@mail.ru

**Keywords:** ring-opening polymerization, random copolymers, ethylene phosphate, lactide, caprolactone, DFT, magnesium phenoxide

## Abstract

Catalytic ring-opening polymerization (ROP) of cyclic esters (lactides, lactones) and cyclic ethylene phosphates is an effective way to process materials with regulated hydrophilicity and controlled biodegradability. Random copolymers of cyclic monomers of different chemical nature are highly attractive due to their high variability of characteristics. Aryloxy-alkoxy complexes of non-toxic metals such as derivatives of 2,6-di-*tert*-butyl-4-methylphenoxy magnesium (BHT-Mg) complexes are effective coordination catalysts for homopolymerization of all types of traditional ROP monomers. In the present paper, we report the results of density functional theory (DFT) modeling of BHT-Mg-catalyzed copolymerization for lactone/lactide, lactone/ethylene phosphate and lactide/ethylene phosphate mixtures. *ε*-Caprolactone (*ε*-CL), *l*-lactide (*l*-LA) and methyl ethylene phosphate (MeOEP) were used as examples of monomers in DFT simulations by the Gaussian-09 program package with the B3PW91/DGTZVP basis set. Both binuclear and mononuclear reaction mechanistic concepts have been applied for the calculations of the reaction profiles. The results of calculations predict the possibility of the formation of random copolymers based on *l*-LA/MeOEP, and substantial hindrance of copolymerization for *ε*-CL/*l*-LA and *ε*-CL/MeOEP pairs. From the mechanistic point of view, the formation of highly stable five-membered chelate by the products of *l*-LA ring-opening and high donor properties of phosphates are the key factors that rule the reactions. The results of DFT modeling have been confirmed by copolymerization experiments.

## 1. Introduction

**Scheme 1:**
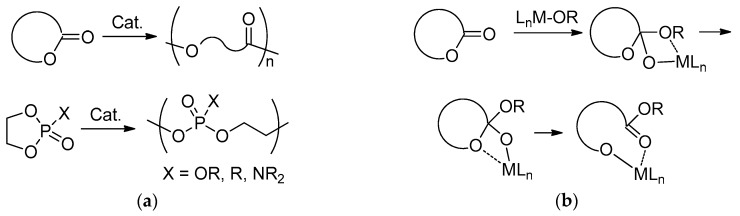
(**a**) Ring-opening polymerization; (**b**) coordination-insertion mechanism of ring-opening polymerization (ROP), catalyzed by metal alkoxides.

Biodegradable and biocompatible polymers have currently received great attention in industrial, biomedical and pharmaceutical applications [1,2,3,4,5,6,7,8]. Catalytic ring-opening polymerization of lactones, lactides, cyclic carbonates and phosphates (Scheme 1a) is the main synthetic approach to these materials with their given hydrophilicity, biodegradability and mechanical properties [2,9,10,11,12]. Metal alkoxy complexes are effective catalysts of ring-opening polymerization (ROP); the mechanistic paradigm for ROP initiated by metal alkoxides bases on “coordination-insertion” concept [13,14,15,16,17,18,19,20] (Scheme 1b). The density functional theory (DFT) modeling was successfully applied in the mechanistic study of coordination homopolymerization of lactones [21,22,23,24,25,26,27,28,29,30,31,32,33], lactides [31,34,35,36,37,38,39,40,41,42,43,44,45,46,47,48], cyclic carbonates [30,42,49,50] and phosphates [51].

Random copolymerization of cyclic substrates with different chemical nature offer a wide array of polymer characteristics. The complexes of non-toxic metals such as Al [52,53,54,55,56,57,58,59,60,61,62,63], Zn [64,65] and Ti [66,67,68,69,70,71] have been successfully applied as a catalysts for random copolymerization of *ε*-caprolactone/*l*-lactide ((*ε*-CL)/*l*-LA) [53,54,55,56,57,58,59,60,61,62,64,65,66,67,68,70,71], *ε*-CL/*rac*-LA [69], *l*-LA/methyl ethylene phosphate (MeOEP) [72], *rac*-LA/ethyl ethylene phosphate [52] and *l*-LA/ethyl ethylene phosphonate [63]. To date, the formation of random copolymers has not been studied in detail from the mechanistic point of view, with the application of quantum-chemical methods [70,73].

Aryloxy complexes of Mg demonstrated high catalytic activities in ROP of different cyclic monomers [15,74,75,76]. Recently, we studied heteroleptic 2,6-di-*tert*-butyl-4-methylphenoxy magnesium (BHT-Mg) complexes as catalysts for the synthesis of homopolymers and block copolymers of cyclic esters and phosphates [30,31,43,51,77,78,79,80,81]. In *ε*-CL and LA ROP, these dimeric complexes catalyze polymerization by binuclear mechanism were closely related to the mechanism theoretically studied for di-Zn complexes [39]. The prospects of BHT-Mg complexes in the synthesis of random copolymers remain unclear. In the present paper, we report the results of DFT simulations and experimental study of BHT-Mg-catalyzed ROP in *ε*-CL/*l*-LA, *ε*-CL/MeOEP and *l*-LA/MeOEP mixtures. DFT modeling of the homopolymerization mechanisms for lactides [31,43], lactones [30,31] and ethylene phosphates [51], performed using PRIRODA software [82] at Perdew–Burke–Ernzerhof (PBE)/3ζ level of theory [83] and the Gaussian-09 program package [84] at B3PW91/DGTZVP level of theory [85,86,87,88] was the background of our research.

## 2. Materials and Methods

### 2.1. DFT Calculations

The initial Cartesian coordinates of the stationary points were generated by PRIRODA software (version 4.0, Moscow, Russia) [82] using the 3ζ basis. The final calculations (structure optimization and determination of the thermodynamic parameters) were carried out using the Gaussian 09 program (Gaussian Inc., CT, USA) [84] for the gas phase at 298.15 K. The B3PW91 functional [85,87,88] and the DGTZVP basis set [86] were used in the optimization of the molecular geometries. The choice of this functional/basis set was due to the use of B3PW91/B3PW91 in our previous work on DFT modeling of *ε*-CL, *l*-LA and MeOEP polymerization [31,51,89,90]. Transition states were found by energy scanning with sequential changing of key geometric parameters with a step of 0.01 Å, Berny optimization, and was confirmed by intrinsic reaction coordinate (IRC) method. The plots of the molecular structures, energy parameters and Cartesian coordinates for all stationary points and transition states mentioned in the article have been presented in the Supporting Information.

### 2.2. General Experiment Remarks

All of the synthetic and polymerization experiments were performed under a purified argon atmosphere. CH_2_Cl_2_ was washed with aqueous Na_2_CO_3_, stirred with CaCl_2_ powder, refluxed over CaH_2_ for 8 h and distilled. *l*-Lactide (Merck, NJ, USA) was purified by recrystallization and subsequent sublimation. *ε*-Caprolactone (Merck, NJ, USA) was distilled prior to use under argon over CaH_2_. Methyl ethylene phosphate (MeOEP) [78] and [(BHT)Mg(μ-OBn)(THF)]_2_ [31] were synthesized according to the literature procedures.

CDCl_3_ (D 99.8%, Cambridge Isotope Laboratories, Inc., MS, USA) was distilled over P_2_O_5_ and stored over 4 Å molecular sieves. The ^1^H (400 MHz) and ^31^P (162 MHz) NMR spectra were recorded on a Bruker AVANCE 400 spectrometer (Bruker, MS, USA) at 20 °C. The chemical shifts were reported in ppm relative to the solvent residual peak (*δ* = 7.26 ppm).

### 2.3. Polymerization Experiments

Polymerization was carried out at 20 °C in a 1 M solutions for each of the comonomers in CH_2_Cl_2_. A preheated glass ampoule was equipped with a magnetic stir bar and a septum and then filled with dry argon. Comonomers (10 mmol + 10 mmol) were placed into the ampule. Then, CH_2_Cl_2_ was added to achieve the required concentration. Next, 0.4 mL of a 0.25 M (100 μmol) solution of [(BHT)Mg(μ-OBn)(THF)]_2_ (50:1 comonomer/Mg ratio) in CH_2_Cl_2_ was injected into the stirred monomer solution at the given temperature. After a certain time period, a 5-fold excess of acetic acid was injected into the ampoule to stop the reaction. The monomer conversion was determined using ^1^H NMR spectroscopy by integration of the monomer and polymer resonance signals:

For PCL, C*H*_2_OC=O, δ = 4.2 ppm (monomer) and 4.0 ppm (polymer);
for PLA, C*H*(CH_3_)OC=O, δ = 5.0 ppm (monomer) and 5.1–5.2 ppm (polymer);
for poly(MeOEP), C*H*_2_O, δ = 4.4 ppm (monomer) and 4.2 ppm (polymer).

End-group analysis of ^1^H NMR spectra of polymers was used for the determination of *M*_n_^NMR^ by comparative integration of the signals of polymer protons (see above) and aromatic protons of benzyl groups at 7.3–7.4 ppm.

Size exclusion chromatography (SEC) was performed on an Agilent PL-GPC 220 chromatograph (Agilent Technologies, CA, USA) equipped with a PLgel column, using THF (*ε*CL, LA polymers) or DMF (MeOEP polymers) as the eluents (1 mL/min). The measurements were recorded with universal calibration according to polystyrene standards (*ε*-CL, LA polymers) or poly(ethylene glycol) standards (MeOEP polymers) at 40 °C.

## 3. Results

### 3.1. DFT Modeling of ε-CL/l-LA Copolymerization

**Scheme 2:**
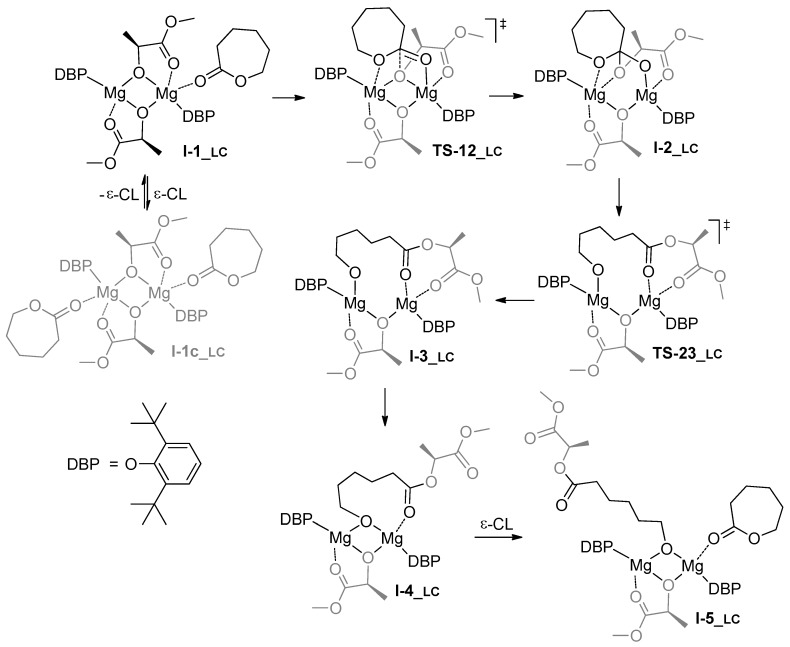
Binuclear reaction mechanism proposed for *ε*-caprolactone/*l*-lactide (*ε*-CL/*L*-LA) copolymerization.

As was demonstrated in our previous work [31], binuclear reaction mechanism was preferable for homopolymerization of both *ε*-CL and *l*-LA. For the simulations of *ε*-CL/*l*-LA copolymerization, we took into account the relatively high stability of binuclear chelates formed by (BHT)Mg and lactate fragments that was experimentally proved by the X-ray diffraction method [31]. To avoid unproductive increasing of the time of calculations, we used 2,6-di-*tert*-butylphenoxy (DBP) fragment instead of BHT to build a model of the initial binuclear complexes **I-1_lc** and **I-1c_lc** that differ by a number of coordinated *ε*-CL molecules (Scheme 2). The calculated free energy (G) of **I-1_lc** was 8 kcal/mol lover than G(**I-1c_lc**), and we had chosen **I-1_lc** as a starting stationary point in the modeling of the reaction profile.

**Figure 1:**
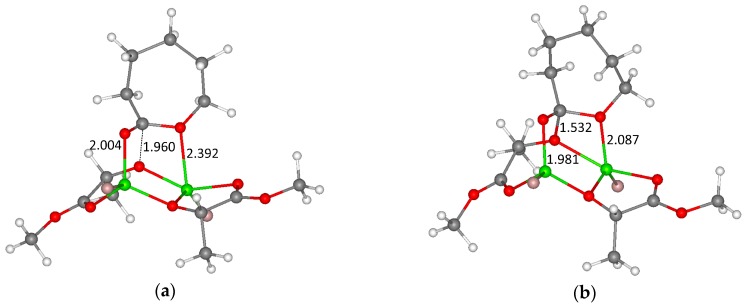
Calculated geometries of (**a**) **TS-12_lc**; (**b**) **I-2_lc**. The 2,6-di-*tert*-butylphenoxy (DBP) fragments are omitted for clarity except the oxygen atom (marked by a rose). The distances O(lactate)–C(*ε*-CL carbonyl C atom) and O(*ε*-CL)–Mg are specified (Å).

In the transition state, **TS-12_lc** of *ε*-CL insertion stage, we noted the distinct cooperative effect (Figure 1): In contrast with mononuclear reaction mechanisms (see [31] and calculations below), the nucleophilic attack of the carbonyl C atom of *ε*-CL by the alkoxy group and the coordination of the endocyclic oxygen atom of *ε*-CL occurred simultaneously due to the presence of two Mg atoms in the catalytic center. The activation barrier of the insertion stage was relatively low, G(**TS-12_lc**) – G(**I-1_lc**) = 18.2 kcal/mol. Note that this value was substantially less than the *l*-LA insertion barrier calculated previously (23.5 kcal/mol, [31]). In the product of the first stage **I-2_lc** we observed a loss of the coordination at one of Mg atoms of the lactate fragment that was involved in the reaction with *ε*-CL (Figure 1b).

The limiting stage of the reaction was a formation of the ring-opening product **I-3_lc**. Specifically, the ring-opening of lactone cycle went easily, but the loss of the coordination of lactate oxygen atom was highly endergonic; this process was constrained by conformational transitions of the “opened” *ε*-CL ring. By the scanning and Berny optimization of the structures between stationary points **I-2_lc** and **I-3_lc**, we found a high-energy transition state; **TS-23_lc**. The value of ΔG = G(**TS-23_lc**) – G(**I-1_lc**) was too high (34.6 kcal/mol) for fast reactions at ambient conditions (for *l*-LA homopolymerization, the activation barrier was estimated by the value of 29 kcal/mol [31]; the ROP of *l*-LA goes slowly in 1 M solution and requires several hours for 100% conversion at 20 °C). The loss of the coordination of the carbonyl group of the lactate fragment (**I-4_lc**) followed by coordination of *ε*-CL molecule resulted in **I-5_cl**. This heteroligand complex was evidently able to catalyze ROP, but we did not see the need for further calculations tailored to the possibility of the coordination of different monomers (*ε*-CL or *l*-LA) and the nucleophilic attack by different alkoxy fragments (opened or chelate). We thought that the comparison of the activation barriers for *l*-LA homopolymerization [31] and for *ε*-CL/*l*-LA copolymerization in the frames of the binuclear mechanism was sufficient to draw a conclusion about the difficulty of *ε*-CL/*l*-LA random copolymerization (see Section 3 for discussion).

### 3.2. DFT Modeling of ε-CL/MeOEP Copolymerization

**Scheme 3:**
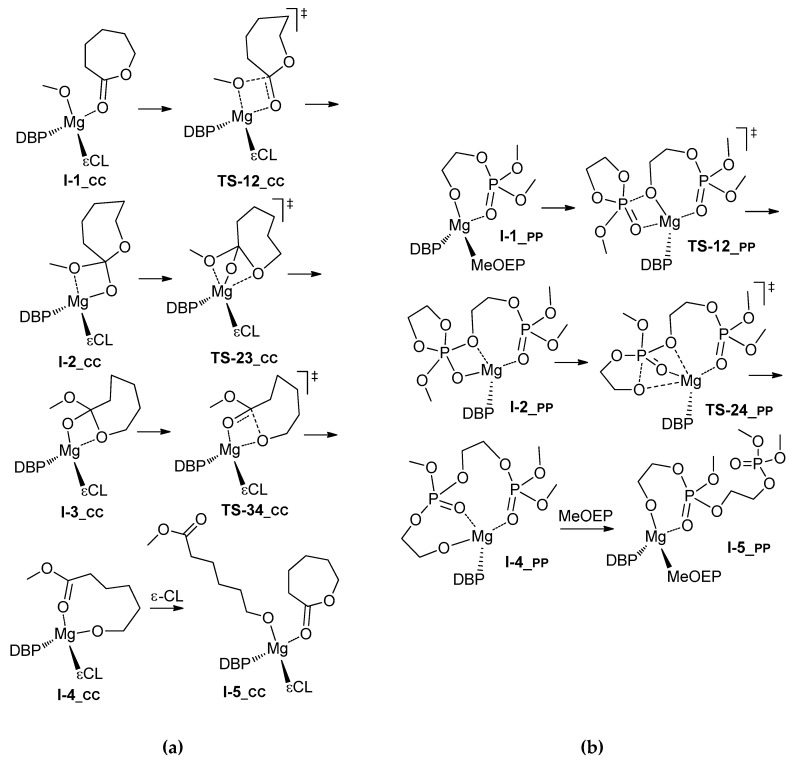
Mononuclear reaction mechanisms of homopolymerization for (**a**) *ε*-CL (CC sequence) [31] and (**b**) MeOEP (PP sequence) [51].

In our recent work [51], we have performed comparative DFT calculations for different mechanisms of the homopolymerization of MeOEP. The results of these calculations indicated the preference of the mononuclear mechanism of the ROP of ethylene phosphates, and this assessment was confirmed by polymerization experiments [51]. We proposed that copolymerization involving MeOEP should occur via the mononuclear reaction mechanism regardless of the type of comonomer. The mononuclear mechanism for homopolymerization of *ε*-CL was simulated in our previous research [31]. These mechanisms are presented in Scheme 3, the coordination number of Mg atom (CN_Mg_) is equal to four for both *ε*-CL and MeOEP homopolymerization mechanisms (CC and PP sequences, respectively). For *ε*-CL, the stationary points and transition states (Scheme 3a) are typical for the coordination-insertion mechanism of ROP; the free energies of the transition states of the insertion and ring-opening stages (**TS-12_cc** and **TS-34_cc**, respectively) have been estimated by the value of ~15 kcal/mol. The mechanism of MeOEP ROP (Scheme 3b) differs significantly from the mechanism of the ROP of lactones by the chelate nature of the catalytic complex and by the absence of the explicit transition state of the ring-opening: The “pendulum” **TS-24_pp** is a transition state between orthophosphate complex **I-2_pp** and ring-opening product **I-4_pp**. The perception of such difference is important when analyzing copolymerization (see below). The values of the calculated free energies and free enthalpies for structures presented in Scheme 3 are given in Table 1.

**Scheme 4:**
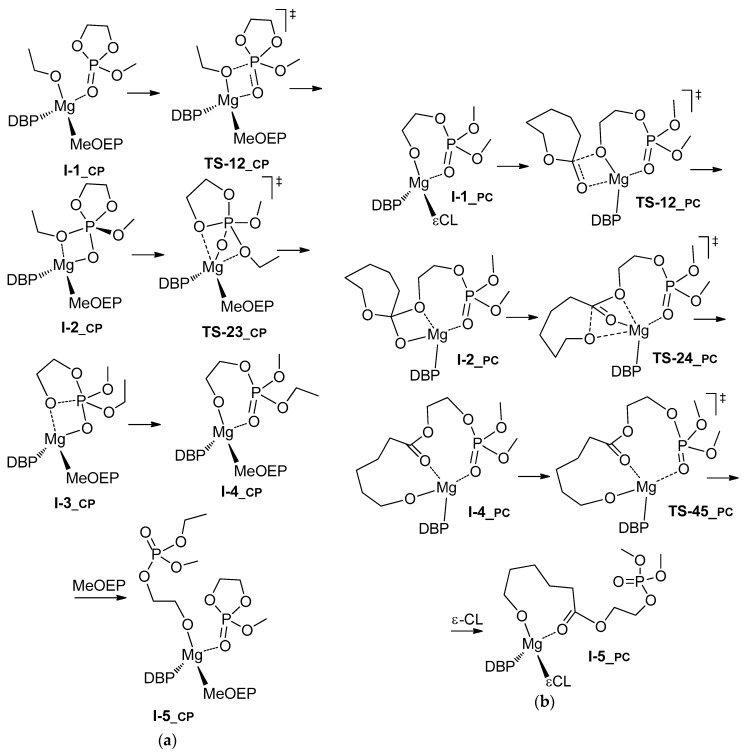
Mononuclear reaction mechanisms of *ε*-CL/MeOEP copolymerization: (**a**) MeOEP in DBP-Mg-OEt complex (PC sequence), and (**b**) *ε*-CL in the DBP-Mg-phosphate complex (CP sequence).

For the analysis of *ε*-CL/MeOEP copolymerization, two processes should be examined in addition to mechanisms of homopolymerization of *ε*-CL and MeOEP. The first is the MeOEP ring-opening in the reaction complex formed by previous *ε*-CL ring-opening (CP sequence). We assumed that the mechanism of this reaction could be analyzed using the simple model, namely, a model of the initiation stage of MeOEP polymerization (Scheme 4a). This reaction was discussed in our previous work [51]. The second process was a coordination and ring-opening of the *ε*-CL molecule in the complex formed by BHT-Mg and OCH_2_CH_2_OP(OMe)_2_ fragment that mimics the growing polyphosphate chain (Scheme 4b).

**Figure 2:**
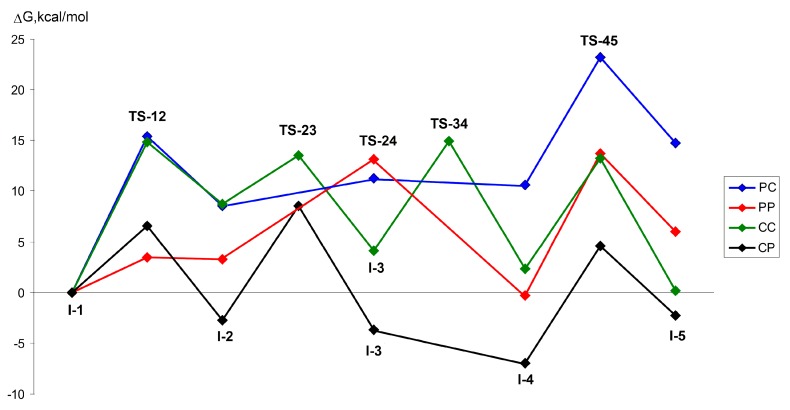
Calculated free energy profiles for all comonomer sequences of *ε*-CL/MeOEP copolymerization based on data obtained earlier [26,46] and in this work.

The first process (CP sequence) should be fast; the calculated relative free energy of the pendulum transition state **TS-23_cp** was only 8.5 kcal/mol [51]. The second reaction, studied in this work (PC sequence), proved to be significantly hindered (Table 1). The calculated energy of the insertion transition state **TS-12_pc** was the highest for the sequences under study (15.4 kcal/mol); however, the free energy of the pendulum **TS-24_pc** was only 11.3 kcal/mol. Probably, the reaching of high-energy stationary point **I-4_pc** was facilitated by the strained ring-opened *ε*-CL fragment (see the SI for details of the molecular geometries of **TS-24_pc** and **I-4_pc**). In contrast with all other sequences, in the case of PC sequence the most hindrance occurred at the final stage, the calculated relative free energy of **TS-45_pc** was 23.2 kcal/mol. This value was much higher than relative free energies of other transition states for all comonomer sequences. Finally, both free energy and free enthalpy of the complex **I-5_pc** were highly positive relatively to **I-1_pc**, thus, the insertion and ring-opening of *ε*-CL after ethylene phosphate seems to be thermodynamically forbidden. The free energy profiles of all possible comonomer sequences for *ε*-CL/MeOEP copolymerization are presented in Figure 2.

### 3.3. DFT Modeling of l-LA/MeOEP Copolymerization

**Scheme 5:**
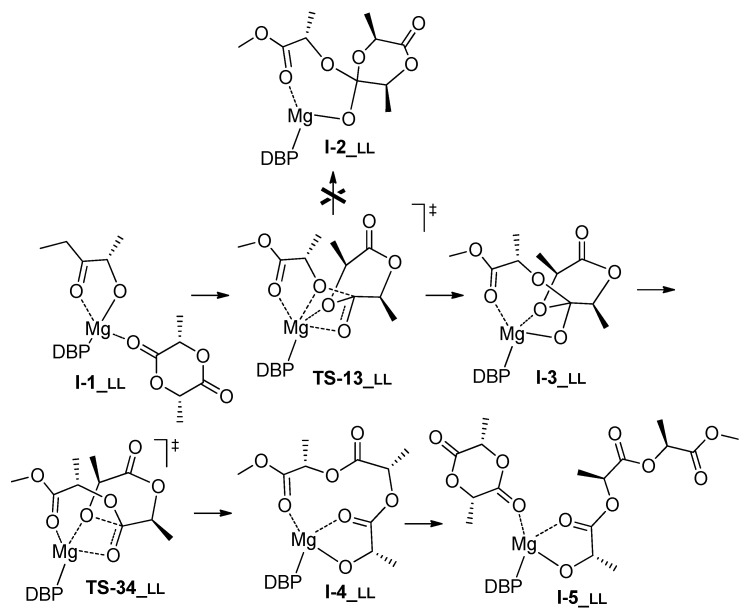
Mononuclear reaction mechanisms of *l*-LA homopolymerization (LL sequence, CN_Mg_ = 4).

The mononuclear concept was also applied for the modeling of copolymerization of *l*-LA and MeOEP. Recently [31], we calculated the energy profile of homopolymerization of lactide for mononuclear mechanism (CN_Mg_ = 5), the free activation energy was estimated by the value of 18 kcal/mol. However, the reaction scheme involving stationary points and transition states with CN_Mg_ = 4 was also applicable for the modeling of *l*-LA polymerization [43]. In this work, we tried to refine the mononuclear mechanism of *l*-LA homopolymerization and used DBP-Mg complex **I-1_ll** (CN_Mg_ = 4) as a starting stationary point of the reaction profile (LL sequence, Scheme 5).

**Scheme 6:**
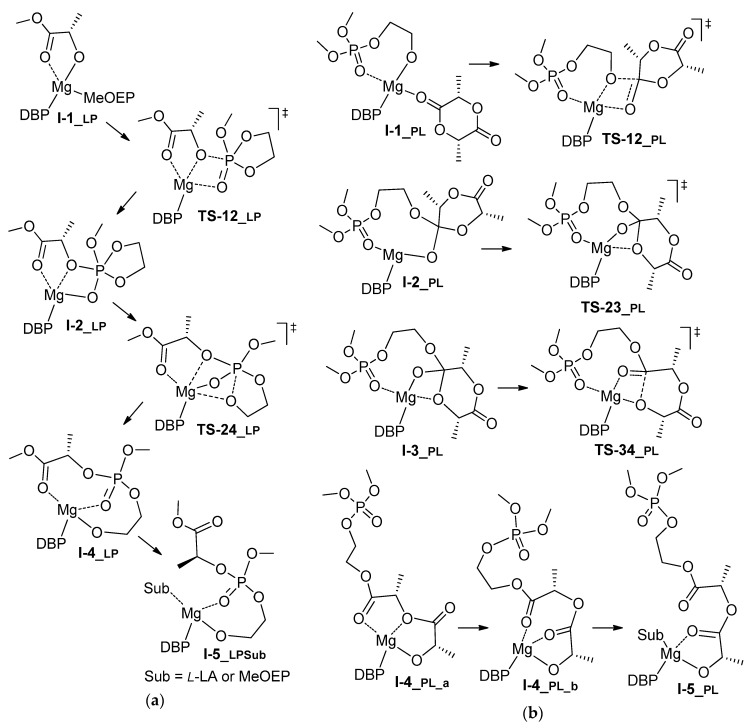
Mononuclear reaction mechanisms of *l*-LA/MeOEP copolymerization: (**a**) LP sequence and (**b**) PL sequence.

**Figure 3:**
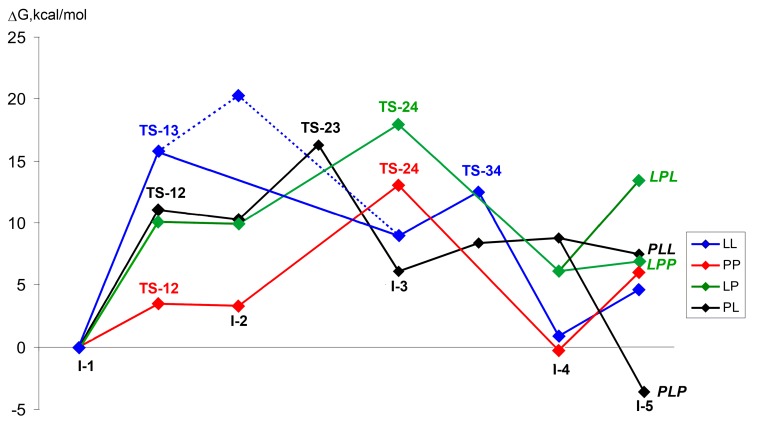
Calculated free energy profiles for all comonomer sequences of *l*-LA/MeOEP copolymerization based on data obtained earlier [51] and in this work.

We made calculations for *re*- and *si*-attack of methyl lactate to the coordinated *l*-LA molecule and we found *re*-attack to be energetically preferable. For *re*-attack, there was no explicit insertion transition state **TS-12_ll**, the formation of the novel C–O bond went hand in hand with the rotation of the coordinated *l*-LA molecule resulting in complex **I-3_ll**. The relative free energy of the corresponding **TS-13_LL** revealed the value of 15.8 kcal/mol (Table 2). To complete the reaction profile in the framework of common ROP mechanism, we found the stationary point **I-2_ll**. The relative free energy of this complex was 20.3 kcal/mol, consequently, **I-2_ll** should be omitted from the reaction profile (Figure 3). The complex analysis of *l*-LA/MeOEP copolymerization should include examining of PP, LP and PL sequences in addition to LL sequence. The data for PP the sequence (Scheme 3b) [51] are duplicated in Table 2 for comparison. Calculations for LP and PL sequences were performed in accordance with Scheme 6. For the LP sequence, the calculated relative free energy of nucleophilic attack of MeOEP molecule by methyl lactate fragment (**TS-12_lp**) was 10.1 kcal/mol that is 6.6 kcal/mol higher than the free energy of **TS-12_pp**. Transition via **TS-24_lp** resulted in ring-opened complex **I-4_pp** in the same way as we found for CP and PP sequences (Scheme 4). The relative free energy of **TS-24_lp** was 17.9 kcal/mol. For the PL sequence, the free activation energy estimated by the value of ΔG(**TS-23_pl**) was 16.3 kcal/mol.

The free energy profiles of all possible comonomer sequences for *l*-LA/MeOEP copolymerization are presented in Figure 3. In summary, the free activation energies for all comonomer sequences discussed above did not exceeded 18 kcal/mol. Thus, all reactions may proceed at ambient conditions. However, the activation barrier of the homopolymerization of MeOEP was minimal (ΔG^≠^ = 13.1 kcal/mol) which suggests the preference of poly(MeOEP) formation even in the presence of *l*-LA.

In recent papers [89,90] we reported the results of DFT simulations of transesterification which accompanies MeOEP homopolymerization. Obviously, such a process may occur in *l*-LA/MeOEP mixture—this issue is discussed in Section 3.

### 3.4. Copolymerization Experiments

Copolymerization experiments were performed in CH_2_Cl_2_ solutions of *ε*-CL/*l*-LA, *ε*-CL/MeOEP and *l*-LA/MeOEP mixtures of comonomers taken in 1 M concentrations ([Mon^1,2^]/[Mg] = 50), [(BHT)Mg(μ-OBn)(THF)]_2_ [31] was used as a catalyst (Table 3, runs 1–6). Comparative homopolymerization experiments using 100:1 [Mon]/[Mg] ratios under the same conditions were also performed (Table 3, runs c1–c3).

When 1:1 *ε*-CL/*l*-LA mixture was introduced into the reaction, we detected homopolymerization of *l*-LA with a formation of PLLA. After 10 min at 5 °C, the conversion of *l*-LA was 86% (Table 3, run 1a, see Figure S1 in the Supporting Information). This value was substantially higher than detected in homopolymerization of *l*-LA (Table 3, run c2). After 30 h of the reaction, *l*-LA was completely polymerized, but *ε*-CL did not react and remained in the reaction mixture (Table 3, run 1b, see Figure S2 in Supporting Information). This kind of catalytic behavior was earlier observed in Zn dinuclear systems [91].

Similarly, in the case of 1:1 *ε*-CL/MeOEP mixture we detected the fast formation of poly(MeOEP) (Table 3, run 2a, see Figure S3 in Supporting Information). The rate of the ROP was comparable with the reaction rate in homopolymerization experiment (Table 3, run c3). The polymer obtained in the presence of *ε*-CL after 10 min at 5 °C was moderately branched (see Figure S4 in the Supporting Information). After 48 h of the reaction, we detected 28% *ε*-CL conversion. The ^31^P NMR spectrum (see Figure S4 in Supporting Information) reflected the formation of the transesterification products via the scission of polyphosphate chain by alkoxy fragments formed by the *ε*-CL ring-opening. This process was very slow, and its synthetic prospects were questionable.

**Figure 4:**
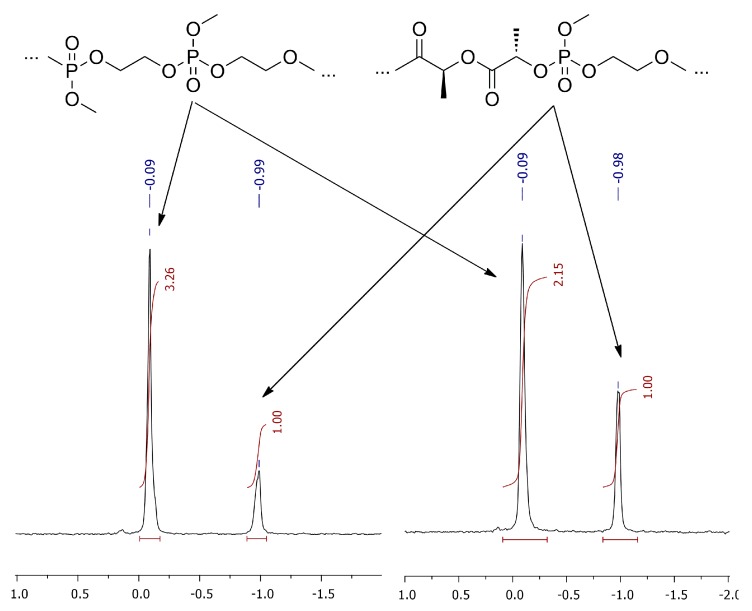
The ^31^P NMR spectra of *l*-LA/MeOEP copolymers (Table 3, runs 3a and 3b).

A qualitatively different result was obtained for 1:1 *l*-LA/MeOEP mixture (Table 3, run 3). After 20 min at 5 °C we detected the full conversion of MeOEP and 89% conversion of *l*-LA with a formation of the polymer containing considerable amount of lactide-phosphate fragments (^31^P NMR data, see Figure 4). After 1 h (Table 3, run 3b) the full conversion of *l*-LA was reached, and the rate of lactide-phosphate fragments was considerably increased.

## 4. Discussion

For *ε*-CL/*l*-LA mixture we detected the formation of PLLA, the polymer formed did not contain even traces of *ε*-CL ring-opening products. This fact can be explained by the difference in free activation energies for *l*-LA and *ε*-CL reactions with binuclear lactate complex (more than 5 kcal/mol). From the other hand, the presence of *ε*-CL resulted in acceleration of *l*-LA polymerization. It’s probably safe to assume that higher ability of *ε*-CL to coordinate the Mg atom can result in dissociation of bimetallic complexes with a formation of mononuclear catalytic species. These species have substantially lower activation barriers in *l*-LA ROP. At the same time, these species constitute stable chelate complexes containing coordinated lactate fragment that are active in *l*-LA ROP but less active in *ε*-CL ROP since the ring-opening of *ε*-CL results in loss of favorable lactate coordination. It’s probably safe to assume that such comparative advantage of lactate chelate coordination leads to slowing down the reaction when *l*-LA have almost been depleted, without involvement of *ε*-CL in ROP. In the absence of polymerization that maintains BHT-Mg species in active mononuclear form, these species can turn into inactive clusters. The formation of such polymetallic species by the reaction of BHT-Mg complexes with lactates in the absence of ROP monomers had been demonstrated in our recent publication [92].

When *ε*-CL/MeOEP mixture was introduced to polymerization, we detected the formation of poly(MeOEP). The modest reduction of the polymerization rate in the presence of *ε*-CL can be explained by competitive inhibition of the catalyst by reversible *ε*-CL coordination. MeOEP is a stronger donor in comparison with *ε*-CL [31], and the latter is unable to hamper the coordination and polymerization of MeOEP. However, the results of DFT modeling of *ε*-CL coordination and ring-opening initiated by BHT-Mg-(MeO)_2_P(O)OCH_2_CH_2_O species (PC sequence, see Figure 3) demonstrates inability of this reaction for the reasons of the value of the activation barrier that is 10 kcal/mol higher than for PP sequence, and an endergonic character of the reaction (G(**I-5_pc**)-G(**I-1_pc**) = 14.7 kcal/mol). Thus, DFT modeling data were in good agreement with the experimental results. The additional factor leading to inertness of *ε*-CL in copolymerization with MeOEP may be highly exergonic coordination of polyphosphate at Mg atom that prevent *ε*-CL coordination.

**Scheme 7:**
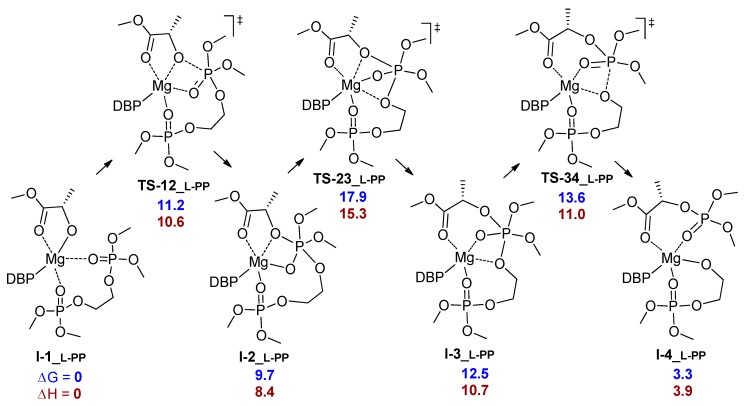
Stationary points and transition states of the process modeling transesterification of polyphosphate by BHT-Mg-lactate. The values of relative free energies (blue) and free enthalpies (dark red) are also given.

Promising results were obtained for the 1:1 *l*-LA/MeOEP mixture. The energy profiles for all possible comonomer sequences (Figure 4) indicate that the most competitive reaction pathway was a formation of poly(MeOEP). However, PL and LP sequences are not forbidden, the free activation energies for these reactions are only 3–4 kcal/mol higher relative to ΔG^≠^_PP_. Another important aspect of DFT modeling of *l*-LA/MeOEP copolymerization is a more readily *l*-LA polymerization initiated by phosphate (PL sequence) in comparison with *l*-methyl lactate-initiated process (LL sequence). Generally, the results presented in Figure 4 and in Table 2 predicted the possibility of the formation of random copolymer. The MeOEP homopolymerization profile had the lowest calculated activation barrier among four reaction sequences, and the formation of polyphosphate blocks was highly probable. In our experiments, we detected a priority in formation of oligophosphate fragments. Increasing the time of polymerization resulted in an increasing of lactide-phosphate fragments. Our assumption was that at the final stage of copolymerization BHT-Mg-lactate species that catalyze *l*-LA polymerization can also cut the oligophosphate fragments. To evaluate the possibility of such a transesterification process, we preformed DFT modeling of scission of the polyphosphate chain by BHT-Mg methyl lactate complex using the simple model presented in Scheme 7.

The results of DFT simulations indicate the possibility of the transesterification process. The calculated value of the activation barrier for Mg-lactate initiated scission of polyphosphate (ΔG^≠^ = 17.9 kcal/mol) was not much higher than the activation energy of *l*-LA homopolymerization (ΔG^≠^ = 15.8 kcal/mol). The comparison of these activation barriers should be done accounting the relative stability of the starting stationary points **I-1**: the product of the coordination of phosphate **I-1_l_pp** was found to be 6.4 kcal/mol more stable than **I-1_ll**. An additional factor facilitating the transesterification is a solution behavior of poly(MeOEP) that forms dense globule in common organic solvents [89].

In summary, it is probably safe to assume that at the beginning of *l*-LA/MeOEP copolymerization, the product contains long polyphosphate fragments, but after lowering MeOEP concentration, the BHT-Mg-lactate species cut the oligophosphate fragments with a formation of lactide-phosphate copolymers and BHT-Mg-phosphate catalytic species. These reactions resulted in an increase of the homogeneity of the distribution of phosphate and lactide fragments through the copolymer chain with a formation of a random copolymer.

## 5. Conclusions

In our research, we have analyzed the outlook for BHT-Mg catalysts in the synthesis of random copolymers from the monomers with substantially different chemical nature (lactones, lactides, cyclic phosphates). DFT modeling of the reaction profiles for *ε*-CL/*l*-LA, *ε*-CL/MeOEP and *l*-LA/MeOEP comonomer pairs predicted the possibility of random copolymerization only for *l*-LA/MeOEP comonomer mixture. Our estimate has been verified experimentally and confirmed by the synthesis of random *l*-LA/MeOEP copolymers.

DFT modeling of the transesterification in *l*-LA/MeOEP reaction mixture referred to the possibility of the fast break of the polyphosphate chains by BHT-Mg-lactate species. The formation of highly statistical lactide/phosphate copolymers due to fast copolymerization and transesterification processes under the mild reaction conditions opens the way to materials with regulated hydrophilicity and biodegradability that are highly promising for biomedical applications.

## Figures and Tables

**Table 1:** Calculated free energies and free enthalpies (kcal/mol) of stationary points and transition states for different comonomer sequences in *ε*-CL/methyl ethylene phosphate (MeOEP) copolymerization. ^1.^

Comonomer Sequence		I-1	TS-12	I-2	TS-23	I-3 or TS-24	TS-34	I-4	TS-45	I-5
**PP**	G	0.0	3.5	3.3	n.d.	13.1 ^2^	n.d.	–0.3	13.7	6.0
	H	0.0	1.5	2.0	n.d.	9.1 ^2^	n.d.	–2.1	13.0	–3.9
**CC**	G	0.0	14.8	8.7	13.5	4.1	14.9	2.3	13.2	0.2
	H	0.0	12.8	5.7	9.0	0.3	11.2	0.8	–0.9	–8.9
**CP**	G	0.0	6.6	–2.8	8.5	–3.7	n.d.	–7.0	4.6	–2.3
	H	0.0	3.5	–6.0	3.6	–7.1	n.d.	–8.9	–13.3	–12.4
**PC**	G	0.0	15.4	8.5	n.d. ^3^	11.3 ^2^	n.d.	10.6	23.2	14.7
	H	0.0	13.0	7.5	n.d.	9.2 ^2^	n.d.	9.1	21.9	6.5

^1^ Relative to corresponding **I-1**. ^2^ **TS-24**. ^3^ Not determined.

**Table 2:** Calculated free energies and free enthalpies (kcal/mol) of stationary points and transition states for different comonomer sequences in *l*-LA/MeOEP copolymerization. ^1.^

Comonomer Sequence		I-1	TS-12	I-2	TS-23	I-3 or TS-24	TS-34	I-4	I-5
**LL**	G	0.0	15.8 ^2^	20.3	n.d.	8.9	12.5	0.9	4.6
	H	0.0	10.1 ^2^	15.7	n.d.	2.9	7.5	–3.1	–7.4
**PP**	G	0.0	3.5	3.3	n.d.	13.1 ^2^	n.d.	–0.3	6.0
	H	0.0	1.5	2.0	n.d.	9.1 ^2^	n.d.	–2.1	–3.9
**LP**	G	0.0	10.1	9.9	n.d.	17.9 ^2^	n.d.	6.1	13.4/6.9 ^4^
	H	0.0	6.9	5.3	n.d.	13.5 ^2^	n.d.	4.2	1.1/–6.1 ^4^
**PL**	G	0.0	11.0	10.3	16.3	6.1	8.4	8.8	7.5/–3.7 ^4^
	H	0.0	8.8	8.6	13.3	4.1	5.6	12.1	–2.0/–13.3 ^4^

^1^ Relative to corresponding **I-1**. ^2^ **TS-13**. ^3^ Not determined. ^3^ **TS-24**. ^4^ Monomer coordination, L/P.

**Table 3:** Polymerization experimental data for BHT-Mg catalyzed copolymerization in *ε*-CL/*l*-LA, *ε*-CL/MeOEP and *l*-LA/MeOEP mixtures. Reaction conditions: CH_2_Cl_2_, [Mon] = 1 M, [Mon]/[Mg] = 100 in homopolymerization, [Mon^1,2^]/[Mg] = 50 in copolymerization.

Run	Mon^1^	Mon^2^	Reaction time, min	Reaction T, °C	Conv., Mon^1^/ Mon^2^, %	*M_n_*^theo^ ×10^3 a)^	*M_n_*^NMR^ ×10^3 b)^	*M_n_*^SEC^ ×10^3^	*Ð_M_*
c1	*ε*-CL	–	10	5	54	6.3	6.8	6.4 ^c)^	1.18 ^c)^
c2	*l*-LA	–	10	5	21	3.1	4.6	8.1 ^c)^	1.17 ^c)^
c3	MeOEP	–	10	5	>99	13.9	13.7	9.8 ^d)^	1.35 ^d)^
1a	*ε*-CL	*l*-LA	10	5	0/86	6.3	7.0	8.5 ^c)^	1.31 ^c)^
1b	*ε*-CL	*l*-LA	1800	20	0/100	7.3	10.3	10.6 ^c)^	1.28 ^c)^
2a	*ε*-CL	MeOEP	10	5	0/98	6.9	9.3	11.6 ^d)^	1.32 ^d)^
2b	*ε*-CL	MeOEP	2880	20	28/100	8.6	14.1	15.1 ^d)^	1.48 ^d)^
3a	*l*-LA	MeOEP	20	5	89/100	13.5	13.7	16.9 ^d)^	1.21 ^d)^
3b	*l*-LA	MeOEP	60	5	100/100	14.2	16.7	19.4 ^d)^	1.25 ^d)^

M(Mon^1^), M(Mon^2^)—molecular weights of—comonomers; ^a)^ *M_n_*^theo^ = M(Mon^1^) × Conv.^1^ + M(Mon^2^) × Conv.^2^ + M_I_; M_I_—molecular weight of BnOH. ^b)^ Determined by the analysis of the NMR spectra of the reaction mixtures, see Section 2.3 for details. ^c)^ Determined by size exclusion chromatography (SEC) in THF *vs* polystyrene standards and corrected by the factors of 0.56 (*ε*CL) and 0.58 (*rac*-LA). ^d)^ Determined by SEC in MeOEP polymers (DMF) vs PEG standards.

## Supplementary Materials

The following are available online at https://www.mdpi.com/2073-4360/11/10/1641/s1, DFT calculations data: Plots of the molecular geometries, energies and cartesian coordinates for all stationary points and transition states, 20 animation files (gif) that illustrate vibrations related to imaginary frequencies for TS discussed in the manuscript; Figure S1: ^1^H NMR spectrum of the probe taken from *ε*-CL/*l*-LA reaction mixture after 10 min (Table 3, run 1a), Figure S2: ^1^H NMR spectrum of the probe taken from *ε*-CL/*l*-LA reaction mixture after 30 h (Table 3, run 1b), Figure S3: ^1^H NMR spectrum of the probe taken from *ε*-CL/MeOEP reaction mixture after 10 min (Table 3, run 2a), Figure S4: ^31^P {^1^H} NMR spectra of the probes taken from *ε*-CL/*l*-LA reaction mixture after 10 min and 30 h (Table 3, runs 1a and 1b, respectively).
