# Supplementary material for: DFT Visualization and Experimental Evidence of BHT-Mg-Catalyzed Copolymerization of Lactides, Lactones and Ethylene Phosphates
Source: Polymers (Basel). 2019 Oct 10;11(10):1641. doi: 10.3390/polym11101641 (PMC6836241; doi:10.3390/polym11101641)
Supplement: Supplementary file 1 [file polymers-11-01641-s001.zip › 4-polymers-605538-SI-proof done/IN_CoROP_DFT__SI.pdf]

DFT Visualization and Experimental Evidence of BHT-Mg-Catalyzed Copolymerization of Lactides, Lactones and Ethylene Phosphates

Ilya Nifant'ev,[1,2,*] Andrey Shlyakhtin [1], Maxim Kosarev [1], Dmitry Gavrilov[1], Stanislav Karchevsky [3] and Pavel Ivchenko [1,2]

[1] Chemistry Department, M.V.Lomonosov Moscow State University,1-3 Leninskie Gory, 119991 Moscow, Russia; shlyahtinav@mail.ru (A.S.); komrad.kosarev.maksim@gmail.com (M.K.); phpasha1@yandex.ru (P.I.),

[2] A.V. Topchiev Institute of Petrochemical Synthesis RAS, 29 Leninsky Pr., 119991 Moscow, Russia

[3] Joint-stock company "Institute of petroleum refining and petrochemistry", 12 Iniciativnaya Str., 450065 Ufa, Republic of Bashkortostan, Russia; st_karchevsky@mail.ru

* Correspondence: ilnif@yahoo.com or inif@org.chem.msu.ru; Tel.: +7-495-939-4098

SUPPORTING INFORMATION

S1. DFT calculations 2

S1.1. Monomers 2

S1.2. LC sequence, binuclear mechanism 5

S1.3. CC sequence, mononuclear mechanism 23

S1.4. PP sequence, mononuclear mechanism 38

S1.5. CP sequence, mononuclear mechanism 49

S1.6. PC sequence, mononuclear mechanism 62

S1.7. LL sequence, mononuclear mechanism 73

S1.8. LP sequence, mononuclear mechanism 84

S1.9. PL sequence, mononuclear mechanism 95

S1.10. Transesterification of PP 110

S2. Polymerization experiments 123

S1. DFT calculations data: molecular structures, energies and cartesian coordinates

The data for all stationary points and transition states mentioned in the manuscript. Note that the data for monomers (S1.1), ε-CL and MeOEP homopolymerization (S1.3. and S1.4, respectively) have been published previously [26,46,81].

S1.1. Monomers

ε-CL

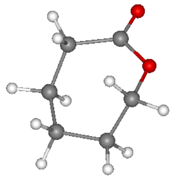

Zero-point vibrational energy	413924.6 (Joules/Mol)
	98.93035 (Kcal/Mol)
Zero-point correction=	0.157656 (Hartree/Particle)
Thermal correction to Energy=	0.164853
Thermal correction to Enthalpy=	0.165798
Thermal correction to Gibbs Free Energy=	0.126149
Sum of electronic and zero-point Energies=	-384.860246
Sum of electronic and thermal Energies=	-384.853048
Sum of electronic and thermal Enthalpies=	-384.852104
Sum of electronic and thermal Free Energies=	-384.891753

cartesian

8	-2.49640012	0.09650000	-0.41440001	1	0.34410000	1.60000002	-1.28910005
6	-1.36909997	-0.02400000	0.01080000	1	-0.27320001	0.89160001	1.62860000
8	-0.77679998	-1.24290001	-0.07160000	1	-1.30460000	1.95330000	0.67290002
6	-0.58899999	1.13250005	0.60570002	6	1.86199999	0.67570001	-0.04630000
6	0.55019999	-1.48150003	0.42449999	1	0.60909998	-1.21550000	1.48740005
6	0.62889999	1.55929995	-0.23090000	1	1.42149997	-0.92960000	-1.44739997
6	1.64820004	-0.79979998	-0.38229999	1	2.58159995	-1.34459996	-0.19520000
1	0.65160000	-2.56620002	0.35339999	1	2.20040011	0.75480002	0.99610001
1	0.88940001	2.58470011	0.05370000	1	2.67910004	1.06920004	-0.66170001

ʟ-LA

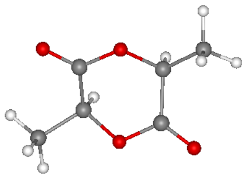

Zero-point vibrational energy	370616.7 (Joules/Mol)
	88.57951 (Kcal/Mol)
Zero-point correction=	0.141160 (Hartree/Particle)
Thermal correction to Energy=	0.150579
Thermal correction to Enthalpy=	0.151523
Thermal correction to Gibbs Free Energy=	0.106579
Sum of electronic and zero-point Energies=	-534.087278
Sum of electronic and thermal Energies=	-534.077859
Sum of electronic and thermal Enthalpies=	-534.076915
Sum of electronic and thermal Free Energies=	-534.121860

cartesian

8	-0.25319999	-1.35280001	-0.11950000	1	-3.39499998	-0.39100000	0.48490000
6	-1.25360000	-0.46730000	0.42940000	1	-2.73480010	-1.02769995	-1.02980006
6	1.03890002	-0.96079999	-0.06110000	1	-2.71370006	-2.03559995	0.43599999
6	-1.03890002	0.96079999	-0.06120000	1	1.14250004	0.46200001	1.52349997
6	-2.61220002	-1.01740003	0.05490000	1	2.71329999	2.03600001	0.43540001
1	-1.14269996	-0.46180001	1.52359998	1	3.39499998	0.39160001	0.48590001
6	1.25360000	0.46730000	0.42940000	1	2.73539996	1.02680004	-1.02960002
6	2.61220002	1.01740003	0.05500000	8	1.92960000	-1.71019995	-0.37459999
8	0.25319999	1.35270000	-0.11990000	8	-1.92960000	1.71029997	-0.37439999

MeOEP

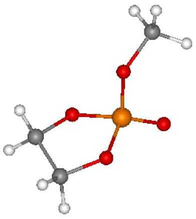

Zero-point vibrational energy	303039.1 (Joules/Mol)
	72.42807 (Kcal/Mol)
Zero-point correction=	0.115421 (Hartree/Particle)
Thermal correction to Energy=	0.123826
Thermal correction to Enthalpy=	0.124770
Thermal correction to Gibbs Free Energy=	0.081434

Sum of electronic and zero-point Energies= -760.559526
Sum of electronic and thermal Energies= -760.551121
Sum of electronic and thermal Enthalpies= -760.550177
Sum of electronic and thermal Free Energies= -760.593513

cartesian

8	1.01199996	1.91747999	-0.66900665	1	-2.53900003	-0.69611996	-1.28200662
15	0.33869994	0.72608006	-0.13230667	1	-1.50830007	-1.59751987	-0.13230667
8	-0.78800011	0.88518006	1.01729333	8	1.31199992	-0.35001999	0.54839331
8	-0.57990009	-0.08291996	-1.20180666	6	2.68899989	-0.42441994	0.12929332
6	-2.06210017	0.40018004	0.56029332	1	3.11589980	0.57578003	0.03799333
6	-1.74150014	-0.60941994	-0.54280668	1	2.76249981	-0.95062000	-0.82600665
1	-2.57720017	-0.05011997	1.40999329	1	3.20859981	-0.98942000	0.90299332
1	-2.64270020	1.24588001	0.17999333				

S1.2. LC sequence, binuclear mechanism

I-1_LC

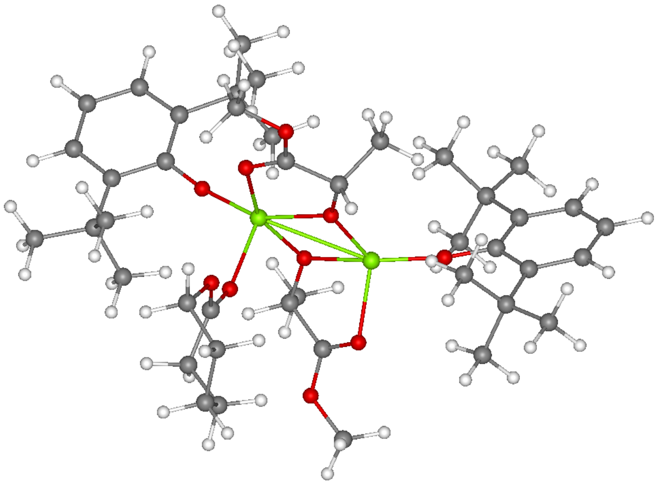

Zero-point vibrational energy	2702940.5 (Joules/Mol)
	646.01829 (Kcal/Mol)
Zero-point correction=	1.029496 (Hartree/Particle)
Thermal correction to Energy=	1.093004
Thermal correction to Enthalpy=	1.093948
Thermal correction to Gibbs Free Energy=	0.926001
Sum of electronic and zero-point Energies=	-2791.161249
Sum of electronic and thermal Energies=	-2791.097741
Sum of electronic and thermal Enthalpies=	-2791.096796
Sum of electronic and thermal Free Energies=	-2791.264744

cartesian

12	-1.29932415	-0.08807253	0.27690756	1	6.19297600	3.72722745	1.21070755
12	1.67837584	0.03872747	-0.43909246	1	6.84967566	2.37932754	2.14260745
8	-2.88052416	-1.08357251	-0.04679247	6	4.92237568	2.38472748	-0.72739244
8	3.49627590	-0.26527253	-0.19529246	1	5.76547575	2.31842756	-1.42409241
8	-0.05022418	-0.47627252	-1.28979242	1	4.08747578	1.83242750	-1.15959239
8	1.68627584	1.25552750	-2.20109248	1	4.63807583	3.44222760	-0.64449245
8	0.66347581	1.46302748	-4.18579245	6	4.18777561	2.02972746	1.66990757
6	-0.25562420	-0.12067255	-2.61529255	1	3.29257584	1.47602749	1.38370752
6	-0.17412420	-1.31647253	-3.57279253	1	4.49407578	1.68292749	2.66320753
1	-1.23572421	0.36192748	-2.75829268	1	3.92997575	3.09382749	1.74900758
6	0.78507584	0.92992753	-2.98069263	6	4.83037567	-4.45317268	-0.75949246
6	1.67627585	2.41482759	-4.57479239	1	5.67487574	-4.44207287	-1.45669246
1	1.40827584	2.72262740	-5.58369255	1	5.18137598	-4.84357262	0.20150754
1	2.65957570	1.94262755	-4.56239271	1	4.09417582	-5.16497278	-1.14959240

6	-3.93402410	-1.88177252	-0.10859247	6	2.98617578	-3.25327253	0.34820756
6	-5.21152401	-1.35957253	-0.50549245	1	2.45847583	-2.31757259	0.53970754
6	-6.30742407	-2.22457242	-0.57029247	1	2.26637578	-3.97747254	-0.05089247
1	-7.27852440	-1.84467244	-0.86999243	1	3.34147573	-3.62687254	1.31490755
6	-6.20202398	-3.57577252	-0.26129246	6	3.67507577	-2.68547249	-2.03049254
6	-4.96482420	-4.08047295	0.12350753	1	4.51347589	-2.66987252	-2.73549247
1	-4.89862442	-5.13707256	0.36070755	1	2.94057584	-3.41497254	-2.39299250
6	-3.82342410	-3.27817249	0.20930754	1	3.22037578	-1.69317245	-2.04549265
6	-5.39882421	0.12762748	-0.85649246	1	-7.07102442	-4.22677279	-0.32049245
6	-2.48612428	-3.91127253	0.63570756	1	8.39237595	-1.72547245	0.94900757
6	-6.84272432	0.46312749	-1.27149248	1	1.67427576	3.26872754	-3.89549255
1	-7.15812397	-0.09197253	-2.16119266	1	-0.34592420	-1.01427245	-4.60979271
1	-7.56272411	0.26662749	-0.47009245	1	-0.94012421	-2.03737259	-3.28149247
1	-6.90952396	1.53082752	-1.51389241	1	0.80167580	-1.80437243	-3.49699259
6	-5.08052397	1.00202751	0.37250754	8	0.53277582	0.24012746	1.13790751
1	-5.17872429	2.06832743	0.12170753	8	-1.75272417	0.29522747	2.38000751
1	-5.78632402	0.78602749	1.18250751	8	-0.83182418	0.74212754	4.37730742
1	-4.07492399	0.81562752	0.74930757	6	0.65077579	0.44322747	2.50670743
6	-4.50152397	0.51032752	-2.04989266	6	1.48687577	-0.63387251	3.20590734
1	-4.78872442	-0.06237253	-2.93879247	1	1.10067582	1.42502749	2.72960734
1	-4.61112404	1.57712746	-2.28959250	6	-0.75922418	0.48712748	3.07700753
1	-3.45322418	0.30292749	-1.83609247	6	-2.15512419	0.73782754	4.95080757
6	-2.60442424	-5.42187262	0.90720755	1	-2.77602410	1.49882746	4.47530746
1	-3.30572414	-5.64677286	1.71800756	1	-2.61892414	-0.24087255	4.81900740
1	-2.91582417	-5.97977257	0.01770753	1	-2.01192403	0.95832753	6.00710726
1	-1.62442422	-5.80997276	1.20790756	1	1.57417583	-0.43837252	4.27830744
6	-1.99052417	-3.27487254	1.94950759	1	2.48747587	-0.64187247	2.76790738
1	-1.94262421	-2.18777251	1.87990761	1	1.03957582	-1.62087250	3.05840755
1	-2.67602420	-3.51627254	2.76990747	8	-1.67612422	1.88772750	-0.42379245
1	-0.99712420	-3.65827250	2.21500754	6	-2.02112412	3.02252746	-0.11489246
6	-1.43522418	-3.75307250	-0.48029244	6	-2.35272408	4.05212736	-1.16509247
1	-1.25542414	-2.70957255	-0.73909247	8	-2.07042408	3.33622742	1.17680752
1	-0.48152420	-4.20107269	-0.17449246	6	-1.39252424	5.25542736	-1.18559241
1	-1.77092421	-4.26887274	-1.38739240	1	-3.38452411	4.39502716	-1.02109241
6	4.74117565	-0.63677251	0.07610753	1	-2.32252407	3.51982760	-2.11619258
6	5.14377594	-2.00097251	-0.08949247	6	-2.53562427	4.62742710	1.63860750
6	6.45717573	-2.35337257	0.23460753	1	-0.35792419	4.89832735	-1.12049246
1	6.78337574	-3.38217258	0.12360753	1	-1.48932421	5.73482704	-2.16509247
6	7.37837601	-1.42157245	0.70050758	1	-3.54062414	4.81082726	1.24420750
6	6.98787594	-0.09347253	0.83380753	1	-2.62252426	4.48152733	2.71570754
1	7.72397566	0.62182748	1.18590760	6	-1.66392422	6.29822731	-0.10239246

6	5.69247580	0.33382750	0.52690756	1	-2.66202426	6.72672749	-0.26449245
6	4.17087603	-3.06917238	-0.62099242	1	-0.95502418	7.12492704	-0.21899246
6	5.32697582	1.82522750	0.65160757	6	-1.58002424	5.76892710	1.32880759
6	6.50817585	2.67922759	1.14610755	1	-0.56072420	5.44392729	1.56870759
1	7.36277580	2.63892746	0.46280757	1	-1.81512415	6.58342743	2.02390742

I-1c_LC

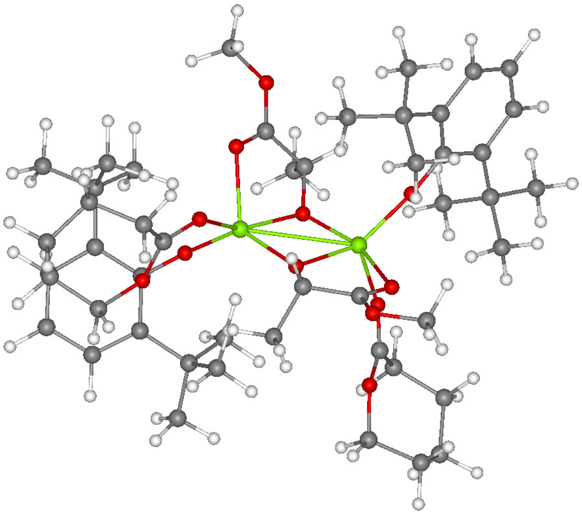

Zero-point vibrational energy	3121106.5 (Joules/Mol)
	745.96236 (Kcal/Mol)
Zero-point correction=	1.188767 (Hartree/Particle)
Thermal correction to Energy=	1.260893
Thermal correction to Enthalpy=	1.261838
Thermal correction to Gibbs Free Energy=	1.076890
Sum of electronic and zero-point Energies=	-3176.031992
Sum of electronic and thermal Energies=	-3175.959865
Sum of electronic and thermal Enthalpies=	-3175.958921
Sum of electronic and thermal Free Energies=	-3176.143869

cartesian

12	-1.12493479	-0.62537104	-0.02796813	1	6.87866497	1.21532893	-2.77986813
12	1.88406515	0.12092903	0.35233185	1	7.75796509	-0.00697098	-1.85756803
8	-2.57043457	0.16362903	-1.05466807	6	4.38136530	0.27232900	-2.51996803
8	3.42436504	-1.00177097	0.13943186	1	4.36896515	-0.42497095	-3.36526799
8	0.61606514	-0.45347098	-1.08866799	1	3.42936516	0.18942903	-1.99726796
8	-0.92373478	-2.57457089	-0.94016814	1	4.46046495	1.28962898	-2.92636800
8	0.15176520	-3.63957095	-2.59706807	6	5.64456511	0.98212898	-0.43596816
8	0.12746519	0.10702901	1.39893198	1	4.78506517	0.89902896	0.22933185
8	2.49246550	0.70662898	2.36933208	1	6.54976511	0.81282896	0.15823185
8	1.79416513	0.62772900	4.50123167	1	5.68946505	2.00622916	-0.83066815

6	0.86076522	-1.40207100	-2.07086802	6	3.61686516	-5.04387093	1.97563195
6	0.66026521	-0.85107100	-3.48876810	1	3.51756525	-5.78757095	1.17733192
1	1.88486516	-1.80297101	-1.99696803	1	4.60286522	-5.16417074	2.43663192
6	-0.05823481	-2.58837080	-1.81756806	1	2.86776543	-5.28427076	2.73923206
6	-0.68433475	-4.78827095	-2.36536813	6	3.48506546	-2.68527079	2.70223188
1	-0.55203485	-5.14977074	-1.34406805	1	3.39056540	-1.63847101	2.41293192
1	-1.73183489	-4.53207111	-2.53036809	1	2.70606518	-2.92837095	3.43773198
6	0.16336519	0.21562903	2.77763200	1	4.45886517	-2.81497097	3.18843198
1	-0.07423481	-0.74587101	3.26603198	6	1.95336521	-3.58037090	0.89613187
6	1.59386516	0.54272896	3.18913198	1	1.87796521	-4.26487112	0.04333187
6	3.14866543	0.89162898	4.91753197	1	1.22016513	-3.89607096	1.64893198
1	3.11916542	0.90212899	6.00593185	1	1.67846513	-2.58357096	0.55183184
1	3.48116541	1.85722899	4.53183174	1	-7.38053465	2.19562912	-1.48136806
6	-3.75413465	0.70062900	-1.27866805	1	7.18136501	-4.42417097	-1.15986800
6	-4.84553480	-0.09107098	-1.78646803	1	3.81356525	0.10622901	4.55543184
6	-6.12633467	0.46992901	-1.82086802	1	-0.35213482	-5.53607082	-3.08376813
1	-6.96543503	-0.12697098	-2.16416812	1	0.84456515	-1.61517107	-4.24946833
6	-6.37243462	1.78862894	-1.45056808	1	1.36456513	-0.02967098	-3.63826799
6	-5.28853464	2.59802914	-1.12426805	1	-0.35293481	-0.45847097	-3.60666800
1	-5.47793484	3.64832902	-0.92686814	6	-0.78323483	1.28022897	3.34863186
6	-3.98073483	2.10612917	-1.06796801	1	-0.73893476	1.30422902	4.44163179
6	-4.63453484	-1.51337099	-2.34176803	1	-1.80533481	1.05482900	3.03913188
6	-2.81723452	3.08632898	-0.82606816	1	-0.52853477	2.26882911	2.95803189
6	-5.88273478	-2.05227089	-3.06456804	8	1.71316516	3.78922915	0.87113184
1	-6.73213482	-2.19937086	-2.38806796	6	2.14686537	3.27112913	-0.27836815
1	-6.20363474	-1.39617097	-3.88056803	6	2.53006506	4.17012930	-1.42726803
1	-5.64953470	-3.03057098	-3.50066805	1	1.66086519	4.76972914	-1.72426808
6	-3.50013494	-1.49607098	-3.38516808	6	1.51196516	5.21092892	1.04243195
1	-3.33293486	-2.50357080	-3.78776813	1	0.99796522	5.26882887	2.00253201
1	-3.76293468	-0.84037101	-4.22286844	6	2.80306530	6.01452923	1.07733202
1	-2.57173491	-1.13097107	-2.94716811	1	0.82246518	5.57512903	0.27323186
6	-4.30923462	-2.51227093	-1.21886802	1	3.52556515	5.48252916	1.70763195
1	-5.15083504	-2.57947087	-0.51886815	6	3.39706516	6.32012892	-0.29696813
1	-4.14263487	-3.51647091	-1.62976801	1	2.58166504	6.95872927	1.58883202
1	-3.41703463	-2.22447085	-0.66696817	1	2.75746536	3.49822903	-2.25496793
6	-3.27433491	4.55612898	-0.87086815	6	3.73176527	5.08652925	-1.13386798
1	-3.75893450	4.80992889	-1.81956804	1	4.52536535	4.50232887	-0.65466815
1	-3.96093464	4.80872917	-0.05536813	1	4.13506508	5.41462898	-2.09736800
1	-2.39853477	5.20792913	-0.76406813	1	2.69116545	6.94902897	-0.85596812
6	-1.75423479	2.92162919	-1.93086803	1	4.30466509	6.92022896	-0.16876814
1	-1.34663486	1.91062903	-1.95366812	8	2.24286509	2.05592918	-0.36586815

1	-2.18813467	3.13972902	-2.91306806	8	-3.93443489	-0.21407098	1.81663203
1	-0.92823482	3.62762904	-1.76766801	6	-3.29003477	-1.32057106	2.15543199
6	-2.18493462	2.86102915	0.55503184	6	-3.81803465	-2.21857095	3.24633193
1	-1.77333486	1.85822904	0.65363187	1	-3.93753481	-1.63307106	4.16693163
1	-1.36723483	3.57252908	0.72913188	6	-5.19763470	0.18552904	2.40143204
1	-2.92783451	3.00272918	1.34833193	1	-5.38333464	1.14502895	1.91883194
6	4.39496517	-1.84977102	-0.15226814	6	-6.34333467	-0.77237099	2.12213206
6	4.41276503	-3.17017078	0.41713184	1	-5.05413485	0.35582903	3.47563195
6	5.41596508	-4.06177092	0.02713187	1	-6.31883478	-1.03107095	1.05843198
1	5.43026495	-5.06747103	0.43443185	6	-6.37473488	-2.01817107	3.00673199
6	6.41706514	-3.70687079	-0.87036812	1	-7.26893473	-0.20527098	2.27523208
6	6.43536520	-2.41017079	-1.37186801	1	-3.03563452	-2.95777082	3.42373204
1	7.23786497	-2.13647079	-2.04866815	6	-5.14373493	-2.91707087	2.89803195
6	5.46446514	-1.46427107	-1.03126800	1	-5.07103491	-3.34887099	1.89403200
6	3.38016510	-3.60897088	1.47213197	1	-5.26043463	-3.76017094	3.58723187
6	5.57266521	-0.03467098	-1.59276807	1	-6.49633503	-1.70857108	4.05413151
6	6.84486532	0.17512903	-2.43436813	1	-7.26473475	-2.60767078	2.75953197
1	6.86776495	-0.46127096	-3.32556796	8	-2.24503469	-1.58527100	1.56383193

TS-12_LC

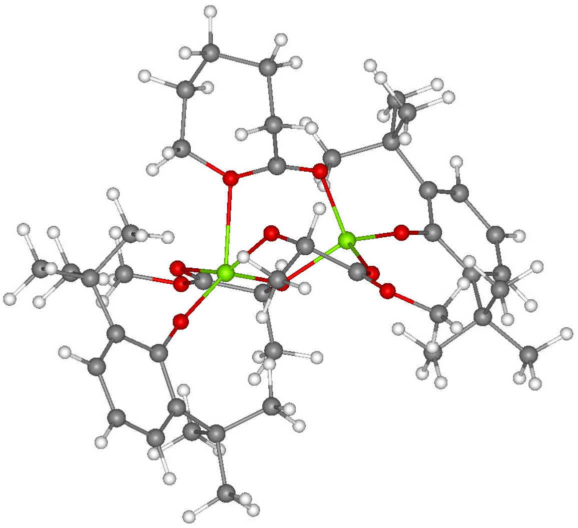

Zero-point vibrational energy	2702706.9 (Joules/Mol)
	645.96245 (Kcal/Mol)
Zero-point correction=	1.029407 (Hartree/Particle)
Thermal correction to Energy=	1.091539
Thermal correction to Enthalpy=	1.092483
Thermal correction to Gibbs Free Energy=	0.929877
Sum of electronic and zero-point Energies=	-2791.136244
Sum of electronic and thermal Energies=	-2791.074112
Sum of electronic and thermal Enthalpies=	-2791.073167

Sum of electronic and thermal Free Energies= -2791.235774

cartesian

12	1.71657324	-0.04279250	-0.57503676	1	-6.79682684	3.04540753	-0.01013676
12	-1.43102682	0.35830751	0.34796324	1	-7.30182695	1.71920753	-1.05573678
8	3.54487324	-0.27829251	-0.30373675	6	-4.94552660	1.85910749	1.46476328
8	-3.15132689	-0.38309249	0.59086323	1	-5.62112665	1.55390751	2.27166319
8	0.41207317	-0.27179250	0.89276320	1	-3.95522690	1.46590745	1.69456327
8	-1.27572680	1.18320751	2.31186318	1	-4.90332699	2.95650744	1.46326327
8	-0.01552683	1.08120751	4.16436338	6	-4.60312700	1.87600756	-1.04673672
6	0.75107312	-0.13379250	2.24496317	1	-3.58682680	1.47900748	-1.01103675
6	0.83517313	-1.47649252	2.97816324	1	-5.03602695	1.58550751	-2.01073670
1	1.71957314	0.37500751	2.36546326	1	-4.55972672	2.97330761	-1.02003670
6	-0.27602684	0.77270752	2.90556335	6	-3.68892670	-4.75629234	1.17276323
6	-0.98302692	1.91170752	4.83996344	1	-4.53852701	-4.89559269	1.84906328
1	-0.61192685	2.01380754	5.85776329	1	-3.93822670	-5.21149254	0.20816323
1	-1.96282685	1.43250751	4.82916307	1	-2.84532690	-5.31909227	1.58826327
6	4.77327299	-0.46189249	0.17036323	6	-2.06762671	-3.24519253	0.10206324
6	5.54307318	0.65360749	0.63486320	1	-1.66532683	-2.23799253	-0.01083677
6	6.79867315	0.40960750	1.20036328	1	-1.26402676	-3.87969255	0.49516323
1	7.39177322	1.23690748	1.57496333	1	-2.33792686	-3.62089252	-0.89123678
6	7.33077335	-0.87109250	1.29626322	6	-2.90402675	-2.79359245	2.45016336
6	6.60927296	-1.94019246	0.77746320	1	-3.75722671	-2.89339256	3.13006330
1	7.05517340	-2.92789245	0.82736319	1	-2.08252692	-3.39969254	2.85256338
6	5.34787321	-1.77399254	0.19796324	1	-2.60362673	-1.74429250	2.44316316
6	5.04377317	2.10480762	0.49286327	1	8.30687332	-1.03099251	1.74766326
6	4.63277340	-2.98669243	-0.42763674	1	-7.71632671	-2.74429250	-0.43583676
6	6.08127308	3.13160753	0.98266321	1	-1.04472685	2.88680744	4.35396338
1	6.29877329	3.02960753	2.05136323	1	1.10957313	-1.33659256	4.02726316
1	7.02387333	3.06080747	0.43026325	1	1.59997320	-2.08749247	2.49626327
1	5.68727303	4.14240742	0.82656324	1	-0.11812682	-2.00609255	2.92246318
6	4.77537298	2.42600751	-0.99173677	8	-1.07622683	0.60030752	-1.61673677
1	4.40187311	3.45210743	-1.10003674	8	0.91757315	-1.27339256	-2.01073670
1	5.70307302	2.34310746	-1.56863678	8	-0.15942682	-2.10129261	-3.77653670
1	4.04287338	1.74690747	-1.42823672	6	-1.13682687	-0.16899250	-2.76903677
6	3.77477312	2.34110761	1.33316326	6	-2.52582693	-0.77259248	-3.00253677
1	3.96667314	2.12580752	2.39026332	1	-0.90022689	0.45470750	-3.65033674
1	3.44707322	3.38510752	1.25256324	6	-0.03442683	-1.22979248	-2.79193664
1	2.95087314	1.70910752	1.00016332	6	0.87947315	-3.09829259	-3.90243673
6	5.47627306	-4.27189255	-0.35163677	1	1.83937323	-2.61719251	-4.09343672
1	6.43377304	-4.17099237	-0.87323678	1	0.94087315	-3.69039249	-2.98903680

1	5.67697334	-4.57879257	0.68026322	1	0.57937312	-3.71599245	-4.74643660
1	4.92827320	-5.09079266	-0.83163679	1	-2.55472684	-1.35999250	-3.92323661
6	4.37407303	-2.71949244	-1.92423677	1	-3.24752688	0.04160750	-3.08143663
1	3.76957321	-1.82379246	-2.07243681	1	-2.82612681	-1.40359247	-2.16403675
1	5.32287312	-2.57939243	-2.45363665	8	1.22317314	1.69040751	-1.45193672
1	3.85757327	-3.57439256	-2.37973666	6	0.07177316	2.18160748	-1.47063673
6	3.31297326	-3.29319239	0.30446324	6	-0.36612684	2.99220753	-2.66583681
1	2.62667322	-2.44639254	0.27896324	8	-0.47642687	2.47390747	-0.22923677
1	2.80717325	-4.15379238	-0.15113677	6	0.23677316	4.40840769	-2.64813662
1	3.50757313	-3.53679252	1.35476327	1	-1.45582676	3.02870750	-2.74063683
6	-4.32192659	-0.97569251	0.39376324	1	0.01377317	2.45670748	-3.53833675
6	-4.44342661	-2.40099239	0.50646323	6	-1.33742678	3.62790751	-0.05513677
6	-5.66732693	-2.99769258	0.18826324	1	1.29267323	4.34370756	-2.36333680
1	-5.77142668	-4.07609272	0.24256323	1	0.21647316	4.79700756	-3.67193675
6	-6.77892685	-2.25239253	-0.18823677	1	-2.22542691	3.52880740	-0.68693680
6	-6.68222666	-0.86579251	-0.20403677	1	-1.65712678	3.53670740	0.98396319
1	-7.57102680	-0.29629251	-0.45393676	6	-0.51072687	5.38540745	-1.74133670
6	-5.49022675	-0.19869250	0.09386324	1	-1.52322686	5.53570747	-2.13953662
6	-3.29052687	-3.27529240	1.03736329	1	-0.01792683	6.36320734	-1.78743672
6	-5.46882677	1.34160745	0.10906324	6	-0.62092686	4.95310736	-0.27833676
6	-6.86932659	1.95360756	-0.07743676	1	0.36807317	4.89620733	0.19116323
1	-7.56972694	1.62370753	0.69676322	1	-1.18222678	5.71780729	0.27206326

I-2_LC

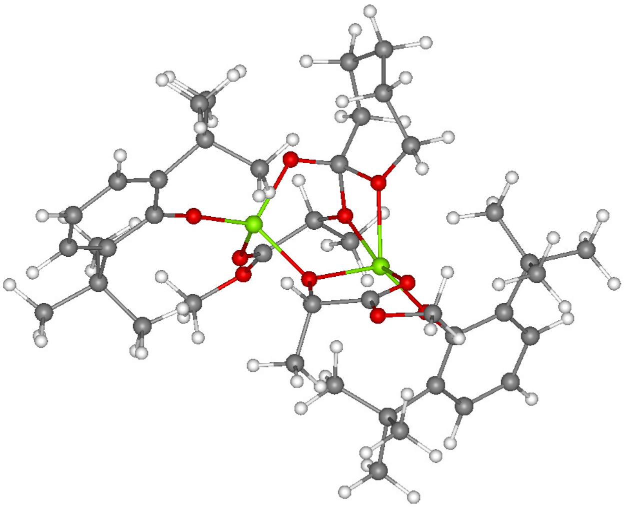

Zero-point vibrational energy	2706139.2 (Joules/Mol)
	646.78280 (Kcal/Mol)
Zero-point correction=	1.030714 (Hartree/Particle)
Thermal correction to Energy=	1.092950
Thermal correction to Enthalpy=	1.093894

Thermal correction to Gibbs Free Energy= 0.932805
Sum of electronic and zero-point Energies= -2791.141338
Sum of electronic and thermal Energies= -2791.079102
Sum of electronic and thermal Enthalpies= -2791.078157
Sum of electronic and thermal Free Energies= -2791.239246

cartesian

12	-1.65489089	0.10832592	-0.60988498	1	6.42560911	-3.58597422	0.03031500
12	1.43380904	-0.48197407	0.49941498	1	7.03100920	-2.33637404	-1.05478501
8	-3.47919083	0.28642592	-0.28138500	6	4.75140905	-2.19807410	1.53451502
8	3.16860914	0.21312591	0.67711502	1	5.48440933	-1.96077418	2.31361508
8	-0.38359100	0.30502594	0.91441500	1	3.81860924	-1.69837415	1.79661500
8	1.10980904	-1.22967410	2.46311498	1	4.58740902	-3.28327417	1.54991496
8	-0.22469100	-0.95467407	4.24431515	6	4.33190918	-2.21587420	-0.96718502
6	-0.76729101	0.28062594	2.26001501	1	3.36130905	-1.72187412	-0.91168499
6	-0.68279099	1.65832591	2.92781496	1	4.76260900	-1.98397410	-1.94788504
1	-1.79729092	-0.08777409	2.37671494	1	4.17590904	-3.30157423	-0.91858500
6	0.12550899	-0.70807409	2.99481511	6	4.17310905	4.50142574	1.19891500
6	0.62380898	-1.85447419	4.98881531	1	5.05370903	4.55802584	1.84671497
1	0.19010898	-1.89747417	5.98581505	1	4.43680906	4.92202568	0.22241500
1	1.64280903	-1.46687412	5.02391529	1	3.40550923	5.15062571	1.63511503
6	-4.73509073	0.40662593	0.13511500	6	2.37270927	3.15282583	0.19351499
6	-5.46679068	-0.74597406	0.56891501	1	1.86960900	2.18862581	0.10831501
6	-6.76079082	-0.56727409	1.06831503	1	1.65050900	3.86932588	0.60451502
1	-7.32799101	-1.42377412	1.41631496	1	2.64900923	3.49112582	-0.81178498
6	-7.36349106	0.68362594	1.13151503	6	3.22890925	2.63902593	2.51961493
6	-6.67079067	1.78942585	0.65211499	1	4.10580921	2.66062593	3.17581511
1	-7.16769075	2.75322580	0.68001497	1	2.48120928	3.32552576	2.93661499
6	-5.37409067	1.68892586	0.13941500	1	2.82700920	1.62422585	2.53321505
6	-4.88309097	-2.16827416	0.46641499	1	-8.36929131	0.79232591	1.53001499
6	-4.68309069	2.94142580	-0.43178502	1	7.91550922	2.07182598	-0.52848500
6	-5.88029099	-3.24607420	0.92991501	1	0.62140900	-2.84207416	4.52491522
1	-6.15229082	-3.13807416	1.98551500	1	-1.00659096	1.61212587	3.97121501
1	-6.79929066	-3.24187422	0.33481500	1	-1.33769095	2.34502578	2.38921499
1	-5.42139101	-4.23437452	0.81201500	1	0.33730900	2.04672575	2.88491488
6	-4.53669071	-2.49187422	-1.00118494	8	1.13110900	-0.44267407	-1.65878499
1	-4.11959076	-3.50387406	-1.08168495	8	-0.75819099	1.62712586	-1.71488500
1	-5.43989086	-2.45247412	-1.61978495	8	0.51380897	2.71832585	-3.18688512
1	-3.81329083	-1.78947413	-1.41578496	6	1.13250899	0.46712592	-2.74368501
6	-3.63759089	-2.32267404	1.35991502	6	2.56680918	0.85042590	-3.08658504
1	-3.88299084	-2.10367417	2.40511489	1	0.66660899	-0.01177408	-3.61568499

1	-3.25209093	-3.34887409	1.31101501	6	0.21020900	1.65352583	-2.46878505
1	-2.83799076	-1.64707410	1.05241501	6	-0.35999101	3.86262584	-3.04778504
6	-5.58609104	4.18702555	-0.38168502	1	-1.37629092	3.59352589	-3.33708501
1	-6.50849104	4.05362558	-0.95638502	1	-0.34879100	4.21182585	-2.01518512
1	-5.85509062	4.46502542	0.64291501	1	0.05080899	4.61542559	-3.71738505
1	-5.05009079	5.03772545	-0.81798500	1	2.59470916	1.55062592	-3.92348504
6	-4.33369064	2.71292591	-1.91638505	1	3.11180925	-0.05417408	-3.36238503
1	-3.68269086	1.84782588	-2.04878497	1	3.06810927	1.29872584	-2.22698498
1	-5.24479103	2.53992581	-2.49918509	8	-1.01439095	-1.34057415	-1.79978502
1	-3.83339071	3.59802580	-2.33068490	6	0.23290899	-1.67987418	-1.75708497
6	-3.41849089	3.28832579	0.37621498	6	0.73070902	-2.58327413	-2.88588500
1	-2.69839072	2.47002578	0.36701500	8	0.65450901	-2.18097425	-0.42908502
1	-2.92889094	4.17992544	-0.03558500	6	-0.22909100	-3.73497415	-3.19498491
1	-3.67839074	3.49932575	1.41951501	1	1.74140906	-2.93777418	-2.65888500
6	4.39080906	0.67282593	0.43941501	1	0.81070900	-1.96697414	-3.78658509
6	4.66170931	2.07792592	0.53331500	6	0.67010897	-3.58747411	-0.12038500
6	5.92800903	2.54242587	0.16451499	1	-1.23629093	-3.31757402	-3.29028511
1	6.14450932	3.60482597	0.20111500	1	0.03650899	-4.15487432	-4.17198467
6	6.94190931	1.68202591	-0.24148500	1	1.64270902	-4.00077438	-0.41658500
6	6.70320940	0.31242591	-0.23408501	1	0.61950898	-3.61037421	0.97061497
1	7.51910925	-0.34967408	-0.50398499	6	-0.22549102	-4.86127424	-2.16218495
6	5.46020937	-0.22527409	0.11371500	1	0.73320901	-5.39637423	-2.20868492
6	3.62140918	3.06822586	1.09131503	1	-0.99419099	-5.59227419	-2.43828511
6	5.28130913	-1.75497413	0.15461500	6	-0.46199101	-4.41387415	-0.71818501
6	6.60620928	-2.50877404	-0.06028500	1	-1.40559089	-3.86757421	-0.62448502
1	7.35940933	-2.23867416	0.68721497	1	-0.55099100	-5.30847454	-0.08928500

TS-23_LC

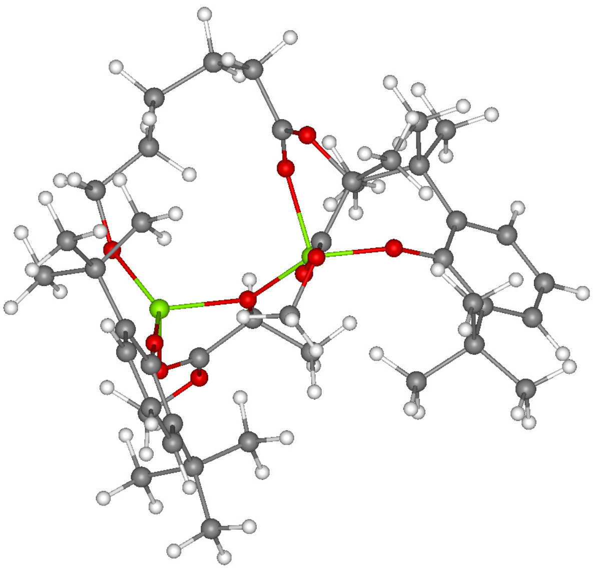

Zero-point vibrational energy	2700224.7 (Joules/Mol)
	645.36920 (Kcal/Mol)
Zero-point correction=	1.028461 (Hartree/Particle)
Thermal correction to Energy=	1.091343
Thermal correction to Enthalpy=	1.092287
Thermal correction to Gibbs Free Energy=	0.929516
Sum of electronic and zero-point Energies=	-2791.110764
Sum of electronic and thermal Energies=	-2791.047882
Sum of electronic and thermal Enthalpies=	-2791.046938
Sum of electronic and thermal Free Energies=	-2791.209709

cartesian

12	1.08600318	0.24337737	0.04757904	1	-5.62019682	3.39997721	-1.46772099
12	-1.92779672	0.36827737	2.00847888	1	-5.35489655	2.23697710	-2.76722097
8	2.81670332	-0.22502263	-0.51302099	6	-5.15259695	1.87597740	0.66537905
8	-3.09209681	-0.36762264	0.72177905	1	-6.15329695	1.45887744	0.82217902
8	0.08590332	-0.11182262	1.67387903	1	-4.48649693	1.45337749	1.41737902
8	-1.55179679	-0.85022265	3.66227913	1	-5.20799685	2.95787716	0.83567905
8	0.14360332	-1.23502254	5.07457924	6	-3.27749681	2.18887711	-1.00642097
6	0.71430337	-0.48972264	2.87567902	1	-2.52429676	1.70957744	-0.37822098
6	1.70510328	-1.64362252	2.69587898	1	-2.98079681	2.07177711	-2.05642104
1	1.24400318	0.36607736	3.32327914	1	-3.28469682	3.26117730	-0.77822095
6	-0.34619668	-0.87532270	3.89887905	6	-3.74049687	-4.71992254	0.18927902
6	-0.82639664	-1.55652261	6.09527922	1	-4.81889677	-4.90792274	0.22097903
1	-0.24049668	-1.79282260	6.98137951	1	-3.36929679	-5.00642252	-0.80102098
1	-1.42489672	-2.41472268	5.78657913	1	-3.27159667	-5.39202261	0.91677904
6	4.00960302	-0.80262268	-0.32462096	6	-1.85559678	-3.15992284	0.51267904
6	5.05800343	-0.09002262	0.34117904	1	-1.50869679	-2.15832281	0.76527905
6	6.26690340	-0.75242269	0.58027905	1	-1.40759683	-3.87142277	1.21807909
1	7.07020330	-0.24032263	1.09827900	1	-1.47979677	-3.40632272	-0.48712099
6	6.48640347	-2.06082273	0.16827902	6	-3.91559672	-3.03082275	1.98207903
6	5.48790312	-2.72252274	-0.53582096	1	-4.99879694	-3.19002271	2.02107906
1	5.68890333	-3.73172283	-0.87802094	1	-3.44579673	-3.73412275	2.68117905
6	4.25290346	-2.12712288	-0.81182098	1	-3.71179676	-2.01782274	2.32837915
6	4.91090345	1.38547742	0.76587903	1	7.43380308	-2.55242276	0.37527904
6	3.21490312	-2.89932275	-1.65082097	1	-6.10559654	-2.21432281	-3.14092088
6	6.21070337	1.95627749	1.36377907	1	-1.47669673	-0.69942266	6.27447939
1	6.50250340	1.44817746	2.28877902	1	2.18010330	-1.90452254	3.64497900
1	7.04780340	1.90157747	0.66047901	1	2.49430323	-1.35862255	1.99477899
1	6.05930328	3.01327729	1.60967898	1	1.19460320	-2.52552271	2.29987907
6	4.57630301	2.25337720	-0.46362096	8	0.56600332	2.70737720	-2.63292098

1	4.44360304	3.30227733	-0.16972098	8	-0.16379668	-0.13352263	-1.54732096
1	5.39210320	2.20787716	-1.19312096	8	-0.91129667	0.02747737	-3.64652085
1	3.66420317	1.90657747	-0.95002091	6	0.97310340	1.37867749	-3.06212091
6	3.83490324	1.55317748	1.85497904	6	1.25790322	1.46687746	-4.54572058
1	4.07650328	0.94737732	2.73457909	1	1.87300324	1.09887743	-2.50342107
1	3.76380324	2.59967732	2.17347908	6	-0.10089668	0.35487735	-2.67782092
1	2.84480333	1.25597739	1.50567901	6	-1.92029679	-0.98822272	-3.35852098
6	3.75170326	-4.25142241	-2.15472102	1	-2.44359684	-1.13182259	-4.29992056
1	4.64630318	-4.13562250	-2.77532101	1	-1.42279673	-1.90262258	-3.03682089
1	3.98610306	-4.93992233	-1.33632100	1	-2.59789681	-0.63372266	-2.58182096
1	2.98590326	-4.73392248	-2.77222085	1	1.58190322	0.49287736	-4.91852093
6	2.84730315	-2.08362269	-2.90572095	1	2.06110334	2.18717718	-4.71542072
1	2.47010326	-1.09992254	-2.62602091	1	0.37140331	1.77887750	-5.09982061
1	3.72830319	-1.94122255	-3.54092097	8	0.98420334	2.26727724	-0.45842099
1	2.08430314	-2.60672283	-3.49602103	6	0.74420333	3.06887722	-1.35572100
6	1.95410323	-3.21532273	-0.82622099	6	0.68490332	4.56127739	-1.18952096
1	1.45900321	-2.31342268	-0.46492094	8	-2.02479672	2.00597715	2.88157892
1	1.22730327	-3.77252269	-1.42892098	6	0.62490332	5.07077742	0.24927902
1	2.20600319	-3.82792282	0.04537904	1	-0.15419668	4.93007755	-1.78902102
6	-3.90489674	-0.83322269	-0.20702097	1	1.58650327	4.93087769	-1.69812095
6	-4.04229689	-2.24922276	-0.40742096	6	-1.97659671	3.37567711	2.71537900
6	-4.82359695	-2.70322275	-1.47402096	1	1.45000327	4.62867737	0.81737906
1	-4.92409658	-3.76892281	-1.65152097	1	0.82910335	6.14597750	0.20857903
6	-5.50209665	-1.83112252	-2.32142091	1	-2.77009678	3.72977710	2.02717900
6	-5.43369675	-0.46422264	-2.07252097	1	-2.17599678	3.88587713	3.67557907
1	-6.00619698	0.20007737	-2.71152091	6	-0.71869665	4.79997730	0.96027905
6	-4.66359663	0.06607737	-1.03122091	1	-1.42879677	4.36547756	0.24507904
6	-3.39159679	-3.26502275	0.55007905	1	-1.17079675	5.74887753	1.27257907
6	-4.67489672	1.58397746	-0.77182096	6	-0.61119664	3.86097717	2.16257906
6	-5.63689661	2.33207726	-1.71252096	1	-0.06039668	2.96287727	1.86397898
1	-6.67069674	1.98897743	-1.60052097	1	-0.01089668	4.33877754	2.94867897

I-3_LC

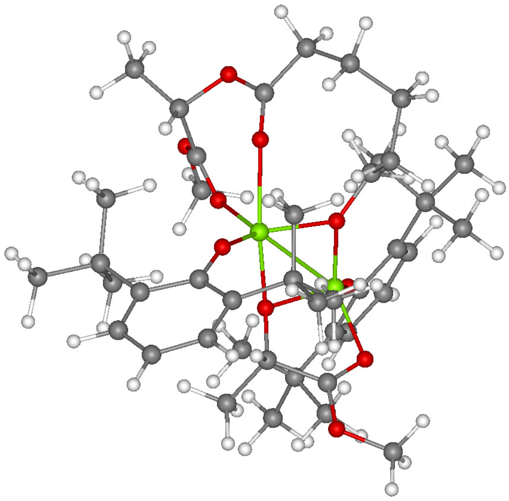

Zero-point vibrational energy	2705348.6 (Joules/Mol)
	646.59384 (Kcal/Mol)
Zero-point correction=	1.030413 (Hartree/Particle)
Thermal correction to Energy=	1.093067
Thermal correction to Enthalpy=	1.094011
Thermal correction to Gibbs Free Energy=	0.932463
Sum of electronic and zero-point Energies=	-2791.143767
Sum of electronic and thermal Energies=	-2791.081113
Sum of electronic and thermal Enthalpies=	-2791.080169
Sum of electronic and thermal Free Energies=	-2791.241717

cartesian

12	-1.17284894	0.46157163	0.05415335	1	3.42635083	3.18437171	-1.35194671
12	1.44325101	-0.68472838	-0.76404667	1	2.97565079	1.78487158	-0.35844666
8	-3.01134920	0.00067163	0.15125336	6	5.96015072	2.51137161	-1.91524673
8	3.24725080	-0.59282833	-0.27964664	1	6.42765093	3.04017162	-1.07814670
8	-0.15884900	-1.29292846	0.26915336	1	6.74635077	2.00717163	-2.48684669
8	0.94865096	-2.39032841	-1.87644672	1	5.51615095	3.26927161	-2.57104659
8	-0.46814901	-4.12502861	-1.87624669	6	4.26225090	0.92267162	-2.73194671
6	-0.66224903	-2.54612827	-0.07014666	1	5.03515100	0.38497165	-3.29154658
6	-0.44624901	-3.58912826	1.03415334	1	3.46725082	0.20977162	-2.50714660
1	-1.74104893	-2.50292826	-0.28594664	1	3.85745072	1.70507157	-3.38604665
6	0.01075100	-3.01022840	-1.35724664	6	5.06435061	-3.67292833	2.35585332
6	0.15635100	-4.59752846	-3.08844662	1	5.87185097	-4.16922855	1.80775332
1	-0.37344900	-5.51282835	-3.34464669	1	5.49865103	-3.15982819	3.22055340
1	1.21425104	-4.79782867	-2.91264677	1	4.40345097	-4.45752859	2.74145341
6	-4.04824924	-0.82142836	0.23915336	6	3.12715077	-2.15082836	2.35195327
6	-4.68224907	-1.32152843	-0.94674665	1	2.45785069	-1.48932838	1.80095327

6	-5.68714905	-2.28462839	-0.81214666	1	2.52685070	-2.95982838	2.78575325
1	-6.15774918	-2.69992828	-1.69714665	1	3.56465077	-1.58282840	3.18165326
6	-6.12624931	-2.72892833	0.43055335	6	3.66765070	-3.58522820	0.32435337
6	-5.59114933	-2.15172839	1.57715333	1	4.47765064	-4.06572866	-0.23574665
1	-5.98694944	-2.46222830	2.53855324	1	3.02535081	-4.37772846	0.72945333
6	-4.57984924	-1.18832839	1.52045333	1	3.08845091	-2.99062824	-0.38274664
6	-4.32594919	-0.78212833	-2.34594679	1	-6.90334940	-3.48582840	0.50375336
6	-4.09564924	-0.51372838	2.81815338	1	8.35735035	-0.52302837	0.90605330
6	-5.25484943	-1.33922839	-3.44044662	1	0.04815099	-3.85392833	-3.87944674
1	-5.15564919	-2.42322826	-3.56294656	1	-0.86854905	-4.55882835	0.75645334
1	-6.30714941	-1.11022842	-3.24284673	1	-0.94464904	-3.23492837	1.93775332
1	-4.99424934	-0.88032836	-4.40094662	1	0.61835098	-3.70542836	1.25125337
6	-4.49404907	0.75127167	-2.36704659	8	0.30655101	0.83517164	-1.26484668
1	-4.22504902	1.15107155	-3.35334659	6	-1.32824898	3.75177169	0.02375335
1	-5.53824902	1.01887155	-2.17014670	6	-1.29814899	4.84867144	-1.00834668
1	-3.87434912	1.23237157	-1.60904670	6	-0.62944901	2.25587177	-3.02234674
6	-2.89354920	-1.16902840	-2.75824666	6	0.50255096	2.00767159	-2.01724672
1	-2.78454924	-2.25932837	-2.78324676	8	-1.76694894	2.62437177	-0.13374664
1	-2.66284919	-0.78412837	-3.75924659	1	0.60475099	2.87477160	-1.34394670
1	-2.14544916	-0.76772839	-2.07274675	1	-0.27654901	5.24487162	-1.03864670
6	-4.94014931	-0.91562837	4.04095316	8	-0.83574903	4.18607140	1.20235336
1	-5.99934912	-0.67222834	3.90735340	8	-0.02214901	1.23337162	1.69885337
1	-4.85854912	-1.98252845	4.27315331	8	1.10425103	2.53837180	3.12395334
1	-4.58404922	-0.36732838	4.92105341	6	-1.01684892	3.33897161	2.36655331
6	-4.22914934	1.01747155	2.68465328	6	-1.01294899	4.24177122	3.58125329
1	-3.68974924	1.38617158	1.81005335	1	-1.96834898	2.81187177	2.26385331
1	-5.28174925	1.29787159	2.56785321	6	0.06925099	2.26187181	2.35875344
1	-3.85134912	1.51427162	3.58915329	6	2.17105079	1.55337155	3.15695333
6	-2.64344931	-0.90952837	3.14355326	1	1.77745104	0.59917164	3.50665331
1	-1.95734894	-0.70322835	2.32165337	1	2.60275078	1.43517160	2.16365337
1	-2.28604913	-0.37582836	4.03445339	1	2.90305090	1.95547163	3.85345340
1	-2.58384919	-1.98222840	3.35765338	1	-1.14484894	3.64747167	4.48855352
6	4.54455090	-0.58302838	0.01305335	1	-1.84294891	4.94807148	3.50755334
6	5.10325098	-1.58482838	0.87275332	1	-0.07684901	4.79617167	3.66045332
6	6.46825075	-1.52842844	1.17085338	1	1.45745099	1.95697153	-2.55724669
1	6.90795088	-2.27682829	1.82125330	1	-1.90244901	5.66107130	-0.58474666
6	7.29905081	-0.53952837	0.65755332	6	-1.81324899	4.49037123	-2.40514660
6	6.75525093	0.42447165	-0.18294664	1	-0.41214901	1.70017159	-3.94164658
1	7.41645098	1.18677163	-0.58084667	6	-0.86874902	3.73487163	-3.35364676
6	5.39915085	0.43497163	-0.52314669	1	-1.55304897	1.82627153	-2.62344670
6	4.24835062	-2.72222829	1.46175337	1	-2.75124931	3.93307161	-2.30804658

6	4.86065102	1.53857160	-1.45174670	1	-2.07344913	5.44117165	-2.88454676
6	3.81585073	2.38977170	-0.70374668	1	0.08935099	4.26917124	-3.42604661
1	4.27705097	2.86667180	0.16905336	1	-1.31584895	3.80117178	-4.35204649

I-4_LC

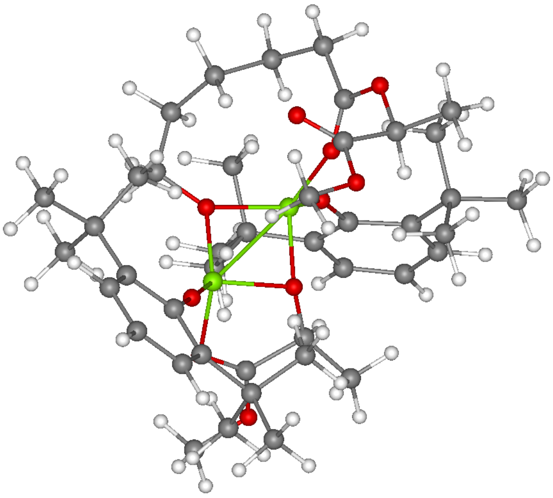

Zero-point vibrational energy	2705362.1 (Joules/Mol)
	646.59707 (Kcal/Mol)
Zero-point correction=	1.030418 (Hartree/Particle)
Thermal correction to Energy=	1.093424
Thermal correction to Enthalpy=	1.094368
Thermal correction to Gibbs Free Energy=	0.931135
Sum of electronic and zero-point Energies=	-2791.147590
Sum of electronic and thermal Energies=	-2791.084584
Sum of electronic and thermal Enthalpies=	-2791.083640
Sum of electronic and thermal Free Energies=	-2791.246873

cartesian

12	1.34161520	0.34419581	0.42331010	1	-3.21248484	1.01079571	3.49460983
12	-1.18218482	-1.16100430	0.36841011	1	-2.81448483	0.71969587	1.78861010
8	3.21521521	0.17199580	0.35571012	6	-5.63348532	-0.12190420	3.58470988
8	-3.02098489	-1.01010430	0.10571011	1	-6.20078516	0.79059589	3.37310982
8	0.30591515	-0.69970411	-0.92188984	1	-6.34048510	-0.95140415	3.69160986
8	-0.49798486	-3.08500409	-0.08728988	1	-5.14578485	0.01769580	4.55600977
8	0.90381515	-4.17940378	-1.44938982	6	-3.80778480	-1.66410422	2.98940992
6	0.87881517	-1.78120422	-1.59758985	1	-4.50248528	-2.50490403	3.09080982
6	0.58081514	-1.75960422	-3.10029006	1	-3.03378487	-1.96930420	2.28260994
1	1.97301507	-1.80170429	-1.46778989	1	-3.33988476	-1.49470425	3.96680999
6	0.36491516	-3.07440424	-0.97358984	6	-5.02748489	-1.71590424	-3.78948998
6	0.43391514	-5.42530394	-0.88958985	1	-5.75548506	-2.52410412	-3.66329002

1	0.99241519	-6.20020390	-1.41058981
1	-0.63708484	-5.53340387	-1.06508982
6	4.34301472	-0.33470419	-0.14768989
6	4.95131493	-1.48370421	0.45561013
6	6.07861471	-2.04170418	-0.15548989
1	6.54261494	-2.92530417	0.26981011
6	6.64621496	-1.49540424	-1.30068982
6	6.10291481	-0.33120421	-1.83058989
1	6.58561468	0.10639581	-2.69799018
6	4.97421503	0.28199580	-1.27728987
6	4.43211508	-2.08290410	1.77681017
6	4.46331501	1.60519576	-1.87968981
6	5.30591488	-3.24710417	2.27760983
1	5.29851484	-4.10040379	1.59061015
1	6.34521484	-2.94420409	2.44010997
1	4.91651487	-3.60130405	3.23880982
6	4.46601486	-1.00360429	2.87700987
1	4.03871489	-1.38900423	3.81130981
1	5.49941492	-0.70160413	3.07950997
1	3.91091514	-0.11430420	2.57621002
6	3.01021504	-2.65380406	1.62001014
1	2.99501514	-3.42420411	0.84051013
1	2.67591524	-3.11650419	2.55671000
1	2.28141522	-1.88560426	1.35911012
6	5.39781475	2.15439582	-2.97319007
1	6.41311502	2.32379580	-2.60009003
1	5.45631504	1.49479580	-3.84529018
1	5.01271486	3.11859584	-3.32459021
6	4.39211512	2.69089580	-0.78698987
1	3.77551508	2.36689591	0.05171011
1	5.39461470	2.90799594	-0.40218988
1	3.98221517	3.62239575	-1.19918990
6	3.08941507	1.40779579	-2.54649019
1	2.33831525	1.03289580	-1.85108984
1	2.72081518	2.35399580	-2.96309018
1	3.16661525	0.68859583	-3.36889005
6	-4.34318495	-0.93260413	-0.01708989
6	-4.96328497	-1.14880431	-1.29148984
6	-6.35338497	-1.03240430	-1.38988984
1	-6.84078503	-1.18980432	-2.34599018
6	-7.15008497	-0.72370416	-0.29358989
1	-5.56928492	-0.80430412	-4.06399012
1	-4.39048529	-1.98250425	-4.64049006
6	-3.14948487	-0.39720419	-2.89229012
1	-2.44728494	-0.19870420	-2.08169007
1	-2.57458496	-0.65840411	-3.78939009
1	-3.68748474	0.53229582	-3.10889006
6	-3.41258478	-2.85570407	-2.31318998
1	-4.13688517	-3.66370416	-2.16219020
1	-2.80018497	-3.11450410	-3.18689013
1	-2.77168489	-2.81910419	-1.43198991
1	7.51921511	-1.95500422	-1.75748980
1	-8.22908592	-0.64250416	-0.39998990
1	0.63861513	-5.45430374	0.18141012
1	1.07311511	-2.58870411	-3.61539006
1	0.95321512	-0.81830412	-3.50879002
1	-0.49518484	-1.80940425	-3.27969003
8	-0.00658486	-0.30800420	1.71731019
6	0.89531517	3.47769594	0.52011013
6	1.60341513	3.91269588	1.77391016
6	0.84481514	0.66879588	3.80690980
6	-0.10308486	-0.32020420	3.13320994
8	0.80431515	2.31609583	0.12221012
1	-1.13298488	-0.08450420	3.42900991
1	0.98671520	4.68419600	2.24550986
8	0.38481516	4.49549627	-0.15948988
8	-2.08688474	3.72709584	0.48281011
8	-2.71188474	3.55959582	-1.68668985
6	-0.48188484	4.20419598	-1.27398992
6	-0.54028487	5.45089626	-2.14069009
1	-0.08278485	3.35499597	-1.83428991
6	-1.84528482	3.80409575	-0.70098984
6	-4.03398514	3.14869595	-1.26698983
1	-3.98518491	2.18859577	-0.75278986
1	-4.46708488	3.90259576	-0.60758984
1	-4.61158514	3.06039596	-2.18469000
1	-1.19268489	5.27059603	-2.99629021
1	0.45981514	5.69649601	-2.50539017
1	-0.92618483	6.30189610	-1.57428992
1	0.11641515	-1.33050430	3.50940990
1	2.52691507	4.41169596	1.45411015
6	1.90201521	2.75549579	2.72110987

6	-6.54588509	-0.53270411	0.94341016	1	0.69931513	0.58649588	4.89081001
1	-7.18128490	-0.30160421	1.79161012	6	0.64061517	2.12139583	3.32980990
6	-5.16208506	-0.62970412	1.11921012	1	1.87651515	0.34759581	3.62110996
6	-4.14588499	-1.51920426	-2.54269004	1	2.49911523	2.00699592	2.18570995
6	-4.55978489	-0.40340421	2.51770997	1	2.56141520	3.11859584	3.51721001
6	-3.63508487	0.82869583	2.49880981	1	-0.16688487	2.13219595	2.59070992
1	-4.19788504	1.72339571	2.21270990	1	0.29131514	2.75189590	4.15491009

I-5_LC

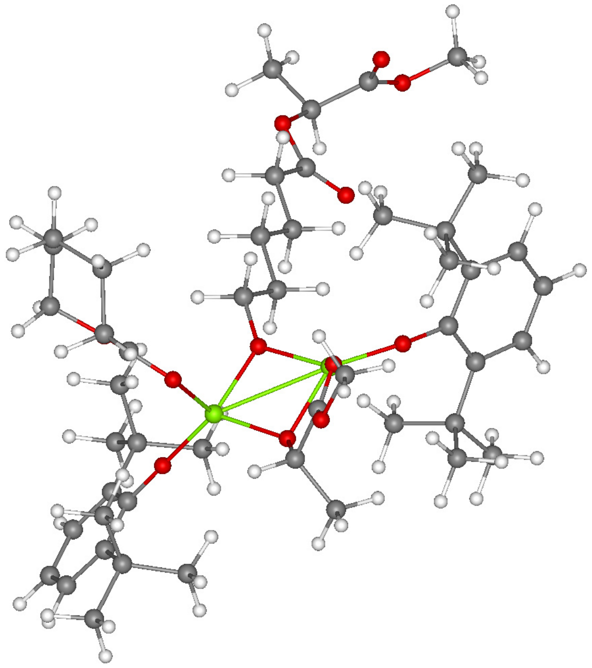

Zero-point vibrational energy	3116563.3 (Joules/Mol)
	744.87651 (Kcal/Mol)
Zero-point correction=	1.187036 (Hartree/Particle)
Thermal correction to Energy=	1.260692
Thermal correction to Enthalpy=	1.261636
Thermal correction to Gibbs Free Energy=	1.064951
Sum of electronic and zero-point Energies=	-3176.019960
Sum of electronic and thermal Energies=	-3175.946304
Sum of electronic and thermal Enthalpies=	-3175.945360
Sum of electronic and thermal Free Energies=	-3176.142045

cartesian

12	-2.52937508	-0.24490061	-0.04085302	1	1.82562494	3.15809917	3.27634716
12	-0.80797499	2.15919924	0.00774698	6	1.73722494	3.28939915	-4.74675322
8	-3.37567520	-1.65160060	-0.92995298	1	1.95642495	4.32989931	-5.00905323
8	0.62042499	3.24799919	-0.45665303	1	2.64762497	2.69799924	-4.88975334

8	-2.63727498	1.64199936	-0.68085301	1	0.99492496	2.92039919	-5.46345329
8	-2.06747508	3.45979929	1.12854695	6	0.89302498	1.65689933	-3.10735297
8	-4.15217495	4.28569937	1.15714693	1	0.50392497	1.44209933	-2.11055303
6	-3.66387510	2.55039930	-0.44015303	1	0.16772497	1.28729939	-3.84185290
6	-4.02267504	3.37209916	-1.68415308	1	1.81552494	1.07769942	-3.22085285
1	-4.57757521	2.04719925	-0.08645302	6	-0.13797504	3.97049928	-3.29725289
6	-3.22117519	3.47159934	0.69034702	1	0.06032497	5.01629925	-3.55735302
6	-3.74297500	5.20639944	2.19174719	1	-0.85327500	3.56939936	-4.02575302
1	-4.63347530	5.78549957	2.42844701	1	-0.60087502	3.95869923	-2.30945301
1	-2.94547510	5.85359955	1.82454693	1	-5.45527506	-5.74300051	-3.46665287
6	-3.89487505	-2.67650080	-1.59855306	1	5.28592491	5.02989960	-2.03175282
6	-5.29097509	-2.69260073	-1.92325306	1	-3.39367509	4.65699959	3.06734705
6	-5.81307507	-3.80700064	-2.58675289	1	-4.84107494	4.06879950	-1.48295307
1	-6.86827517	-3.84020066	-2.83605289	1	-4.32917500	2.68169928	-2.47225285
6	-5.02097511	-4.88940048	-2.95185304	1	-3.15327501	3.93139935	-2.04035282
6	-3.66137505	-4.85380030	-2.66545296	8	-0.70757502	0.26269940	0.52024704
1	-3.05247498	-5.69630051	-2.97565293	6	0.32682496	-0.44980061	1.17764699
6	-3.06797504	-3.77370071	-2.00515294	6	1.59482491	-0.56760055	0.33914694
6	-6.21997499	-1.51380062	-1.57815301	1	0.56812495	0.04849939	2.13004708
6	-1.54967511	-3.77950072	-1.75295305	6	2.69942498	-1.34370065	1.05814695
6	-7.66407490	-1.73560059	-2.06165290	1	1.95272493	0.43689939	0.08314697
1	-7.72327518	-1.85900068	-3.14805293	1	1.34412491	-1.06060064	-0.60735297
1	-8.13277531	-2.60590076	-1.58985305	8	-3.51767516	-0.04710060	1.81164694
1	-8.26857471	-0.85930055	-1.80025303	6	-3.61217499	-0.85270059	2.73864698
6	-6.29477501	-1.31880069	-0.05165302	6	-4.44497490	-0.55500060	3.95574713
1	-6.93797493	-0.46490061	0.19904697	8	-2.93457508	-1.98580062	2.63594699
1	-6.71827507	-2.21100068	0.42434695	6	-3.63307500	-0.47680059	5.26164675
1	-5.30537510	-1.14580059	0.36994699	1	-5.23107529	-1.31500065	4.04534674
6	-5.72087526	-0.22750062	-2.26605296	1	-4.93747520	0.39639941	3.75184703
1	-5.75837517	-0.34030059	-3.35505295	6	-3.03537512	-3.05600071	3.61124706
1	-6.35867500	0.62439942	-1.99575305	1	-2.73557520	0.13099940	5.09694672
1	-4.69017506	0.00449940	-1.99475300	1	-4.24097490	0.06849940	5.99074697
6	-0.86087501	-5.03780031	-2.31105304	1	-4.08617496	-3.34760070	3.70934701
1	-1.23247504	-5.95730066	-1.84615302	1	-2.50537515	-3.87270069	3.12184715
1	-0.97907501	-5.12770033	-3.39585304	6	-3.25707507	-1.83370066	5.85574675
1	0.21402496	-4.98040056	-2.10515285	1	-4.17687511	-2.37510085	6.11384678
6	-1.25637507	-3.76150084	-0.23985302	1	-2.72487497	-1.67080069	6.79914665
1	-1.74737513	-2.92390084	0.25794700	6	-2.40047503	-2.71690083	4.94904661
1	-1.62347507	-4.68440056	0.22464697	1	-1.42177510	-2.25930071	4.76354694
1	-0.17677501	-3.69750071	-0.05535302	1	-2.20437503	-3.66700077	5.45934677
6	-0.89087504	-2.57860065	-2.46045303	1	-0.02797503	-1.45840061	1.43574691

1	-1.30307508	-1.62500060	-2.12795281	6	3.96772480	-1.50540066	0.21864697
1	0.19062497	-2.56890082	-2.28055286	1	2.32212496	-2.33710074	1.34064698
1	-1.05017507	-2.64340067	-3.54205298	1	2.94992495	-0.83450055	1.99914706
6	1.79432487	3.72119927	-0.86455297	6	5.04772472	-2.29240084	0.95404702
6	2.14622498	3.68769932	-2.25125289	1	4.36132479	-0.52290058	-0.06015302
6	3.40502501	4.16329956	-2.63105297	1	3.72712493	-2.01190066	-0.72285300
1	3.69842482	4.13829947	-3.67515302	6	6.31742477	-2.46410084	0.15974697
6	4.31032515	4.67519951	-1.70835304	1	4.69302511	-3.28720069	1.24764693
6	3.94582486	4.73939943	-0.36805305	1	5.33022499	-1.79310060	1.88954699
1	4.65742493	5.15909958	0.33504698	8	7.16562510	-3.32420087	0.78634703
6	2.70322490	4.28649950	0.08554698	8	6.58372498	-1.92500067	-0.89245301
6	1.17272496	3.15749931	-3.32075286	6	8.44272518	-3.49160075	0.16804697
6	2.33252501	4.42939949	1.57424700	6	9.27692509	-2.22500086	0.35104695
6	3.44442487	5.10809946	2.39404702	1	8.31452465	-3.66070080	-0.90425301
1	3.66642499	6.11769962	2.03284717	6	9.11262512	-4.68710041	0.83134699
1	3.11812496	5.19959927	3.43644714	8	10.29522514	-2.21340084	-0.52805299
1	4.37492514	4.53029966	2.39444709	8	9.08222485	-1.37430060	1.18894696
6	1.07432485	5.30949926	1.72124696	6	11.17032528	-1.07680058	-0.43575302
1	1.26952493	6.31499958	1.33194697	1	11.92112446	-1.22330058	-1.21095300
1	0.22762498	4.89299965	1.17514694	1	11.63882446	-1.03370059	0.54984701
1	0.79592496	5.40589952	2.77894711	1	10.61172485	-0.15560061	-0.61115301
6	2.10012484	3.04919934	2.21924710	1	10.09252453	-4.86450052	0.38254699
1	1.30472493	2.49859929	1.71444690	1	8.49662495	-5.57880068	0.69094700
1	3.01042485	2.44219923	2.16674709	1	9.23722458	-4.51350069	1.90314698

S1.3. CC sequence, mononuclear mechanism

I-1_CC

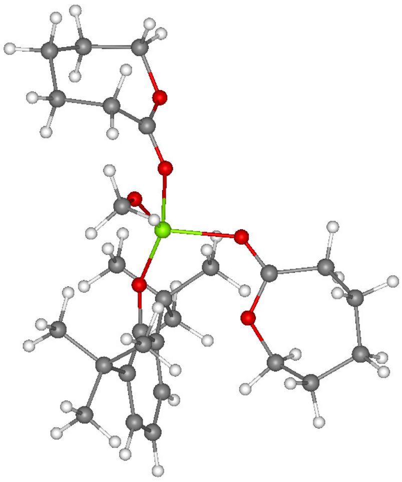

Zero-point vibrational energy	1784683.0 (Joules/Mol)
	426.54948 (Kcal/Mol)
Zero-point correction=	0.679750 (Hartree/Particle)
Thermal correction to Energy=	0.719103
Thermal correction to Enthalpy=	0.720047
Thermal correction to Gibbs Free Energy=	0.604580
Sum of electronic and zero-point Energies=	-1705.797246
Sum of electronic and thermal Energies=	-1705.757893
Sum of electronic and thermal Enthalpies=	-1705.756949
Sum of electronic and thermal Free Energies=	-1705.872416

cartesian

8	-1.63320374	0.75279218	-2.54900122	1	-0.22190370	0.54409218	1.85919869
6	-1.40160370	0.82269216	-3.91410136	8	0.18789630	2.31889224	-0.29470125
1	-1.03700376	-0.13080782	-4.33640146	6	1.31929624	2.47839212	0.17269874
1	-0.65780371	1.59449220	-4.18610144	8	2.16109633	1.46659219	0.10809873
12	-0.80780369	0.56899220	-0.88910127	6	1.73529625	3.79649210	0.77209872
8	0.13459630	-0.89550781	-0.14480126	6	3.46119618	1.46709216	0.74919873
6	1.22249627	-1.51050782	0.28239873	6	2.92789626	4.46169233	0.06189874
6	2.20499635	-1.99840784	-0.64440125	6	4.46029615	2.38929224	0.07159874
6	3.39919639	-2.52220774	-0.13730127	1	3.76279640	0.42199215	0.67419875
1	4.16589642	-2.87290788	-0.82050127	1	2.92339635	5.52069235	0.34079874
6	3.64339638	-2.62950778	1.22909880	1	2.76799631	4.43219233	-1.02230132
6	2.64119625	-2.25680780	2.12049866	1	1.96859622	3.64189219	1.83319879
1	2.82149625	-2.40140772	3.18099880	1	0.85299629	4.43609238	0.72429872
6	1.42389631	-1.72120786	1.68899870	6	4.29299641	3.86909223	0.41459873

6	1.96959639	-1.98390794	-2.16870117	1	3.33159637	1.70519221	1.81019878
6	0.32569629	-1.39340782	2.71959877	1	4.40389633	2.22809219	-1.01120126
6	2.00169635	-0.54830784	-2.72600126	1	5.45969629	2.06109214	0.38019875
1	1.22779632	0.08459218	-2.29080129	1	4.47169638	4.00689220	1.48959875
1	1.84669626	-0.55240786	-3.81130123	1	5.07019615	4.44599199	-0.09900127
1	2.97029638	-0.07790782	-2.52240133	8	-2.44980359	0.88429219	0.33769873
6	0.62799633	-2.66250777	-2.51400137	6	-3.67240381	0.92199218	0.20779873
1	-0.21590370	-2.15730786	-2.04460120	6	-4.36730385	0.67469215	-1.10120130
1	0.63289630	-3.70440793	-2.17460132	8	-4.36890364	1.18019211	1.31499875
1	0.47189629	-2.66190791	-3.59970117	6	-5.16870356	-0.64060783	-1.11420131
6	3.05809641	-2.76440787	-2.93000126	1	-5.03230381	1.51969218	-1.32210124
1	3.12749624	-3.80500793	-2.59570122	1	-3.56980371	0.66189218	-1.85710120
1	4.04889631	-2.30600786	-2.83710122	6	-5.81350374	1.21099222	1.31009877
1	2.80979633	-2.77670789	-3.99730134	1	-4.55880356	-1.44370782	-0.68430126
6	0.69249630	-1.87250781	4.13579845	1	-5.32900381	-0.91320783	-2.16200137
1	1.56999624	-1.35650778	4.54119873	1	-6.16040373	1.96529222	0.59519875
1	0.88229632	-2.95060778	4.16879845	1	-6.05050373	1.56709218	2.31359863
1	-0.14370370	-1.66490781	4.81319857	6	-6.52230358	-0.56210786	-0.40940127
6	-0.98840368	-2.10860777	2.34219885	1	-7.16130352	0.15109217	-0.94790125
1	-1.32730365	-1.81810784	1.34779871	1	-7.02310371	-1.53420782	-0.48150125
1	-1.77440369	-1.86410785	3.06779885	6	-6.45480347	-0.14640781	1.06049871
1	-0.84570372	-3.19510794	2.35229874	1	-5.91880369	-0.89490783	1.65619874
6	0.09229630	0.12619218	2.81439877	1	-7.47280359	-0.10070782	1.46559870
1	1.01079631	0.63259214	3.13669872	1	-2.32560372	1.07689226	-4.46610165
1	-0.69010371	0.35279217	3.54929876	1	4.58229637	-3.04120779	1.59229875

TS-12_CC

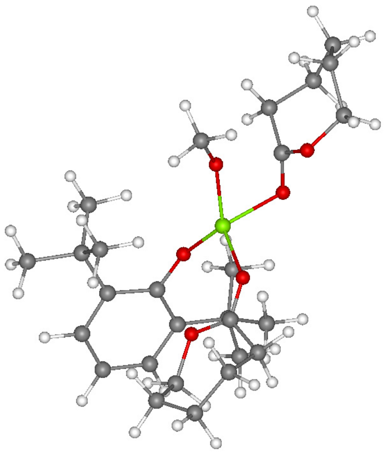

Zero-point vibrational energy 1783379.8 (Joules/Mol)

426.23801 (Kcal/Mol)

Zero-point correction= 0.679254 (Hartree/Particle)

Thermal correction to Energy=	0.717627
Thermal correction to Enthalpy=	0.718571
Thermal correction to Gibbs Free Energy=	0.606288
Sum of electronic and zero-point Energies=	-1705.775815
Sum of electronic and thermal Energies=	-1705.737442
Sum of electronic and thermal Enthalpies=	-1705.736497
Sum of electronic and thermal Free Energies=	-1705.848780

cartesian

8	0.41141027	-1.15696788	-0.07369613	1	-2.06328964	1.48363209	-2.98629618
6	1.69001031	-1.49026787	-0.03679613	8	-2.59148979	-0.21696796	1.08880389
6	2.30881023	-1.84046793	1.20880389	12	-0.98018974	0.10603204	-0.06809613
6	3.68981028	-2.06046796	1.22960389	1	5.53981018	-2.16926789	0.12200387
1	4.18301010	-2.30446792	2.16520381	6	-3.57408977	-0.00136795	0.32490388
6	4.46741009	-1.99406791	0.07620387	6	-4.36918974	-1.17096794	-0.22409612
6	3.84031034	-1.76226795	-1.14559615	8	-4.13858986	1.23363209	0.39440387
1	4.44991016	-1.77586794	-2.04339623	6	-5.60708952	-1.49636793	0.62680387
6	2.46371031	-1.53326786	-1.24389613	1	-4.65528965	-0.97646797	-1.25979614
6	1.48361027	-2.01206779	2.50000381	1	-3.67498970	-2.01206779	-0.23259613
6	1.80941033	-1.37266791	-2.63079619	6	-5.53198957	1.45693207	0.67950386
6	0.91351026	-0.66596794	2.98640370	1	-5.38068962	-1.31876791	1.68450391
1	0.25141028	-0.21386795	2.24790382	1	-5.82098961	-2.56746793	0.55030388
1	0.33271030	-0.80276799	3.90650368	1	-5.63078976	2.53813219	0.56390387
1	1.72361028	0.04093205	3.20330381	1	-5.70948982	1.22123206	1.73700392
6	0.33601028	-3.01746798	2.26750374	6	-6.84738970	-0.70086795	0.19940388
1	-0.32858971	-2.69306779	1.46680391	1	-7.33648968	-1.20586789	-0.64189613
1	0.74171031	-3.99906802	1.99850392	1	-7.57548952	-0.70156795	1.01930380
1	-0.25518972	-3.13586783	3.18390369	6	-6.53958988	0.74493206	-0.21359614
6	2.32831025	-2.57186794	3.65860367	1	-7.46748972	1.32913208	-0.19399613
1	2.78131032	-3.53636789	3.40650368	1	-6.17908955	0.79373205	-1.24669611
1	3.12621021	-1.88756788	3.96840382	8	-0.20808974	1.96393204	0.47260389
1	1.68311024	-2.72986794	4.53010368	6	0.94761026	2.36193204	0.65290385
6	2.79891038	-1.65776789	-3.77539635	8	1.93371034	1.60823214	0.21490386
1	3.62341022	-0.93686795	-3.81029630	6	1.21631026	3.68193221	1.32550383
1	3.22491026	-2.66426802	-3.70729613	6	1.97771025	4.68893194	0.44520387
1	2.27051020	-1.58856785	-4.73289633	1	1.77321029	3.49763203	2.25290370
6	0.65211028	-2.38096786	-2.79249620	1	0.23911029	4.07623196	1.60690391
1	-0.11508973	-2.24266791	-2.03059626	6	3.33221030	1.87703204	0.49130389
1	0.18671027	-2.26826787	-3.77949619	1	1.52491033	4.71423197	-0.55309612
1	1.02981031	-3.40636802	-2.71309614	1	1.81881022	5.68303204	0.87590390
6	1.29531026	0.06283205	-2.84889627	1	3.46591020	1.96803212	1.57440388

1	2.12041020	0.78233200	-2.80179620	1	3.81631017	0.95493203	0.16850388
1	0.81961030	0.15553205	-3.83279634	6	3.48191023	4.43523169	0.34190387
1	0.55941027	0.35193205	-2.09759617	1	3.92411017	4.51903200	1.34390390
8	-2.42238975	0.30173206	-1.32939613	1	3.93341017	5.23423195	-0.25669613
6	-2.89018965	1.03173208	-2.41749620	6	3.86871028	3.08373213	-0.25889611
1	-3.55088973	1.85293210	-2.09219623	1	3.54871035	3.01313210	-1.30499613
1	-3.45608974	0.39653206	-3.11979628	1	4.96131039	2.99503207	-0.26549613

I-2_CC

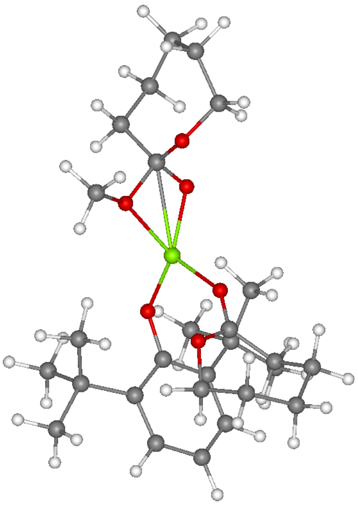

Zero-point vibrational energy	1790760.0 (Joules/Mol)
	428.00192 (Kcal/Mol)
Zero-point correction=	0.682064 (Hartree/Particle)
Thermal correction to Energy=	0.719931
Thermal correction to Enthalpy=	0.720875
Thermal correction to Gibbs Free Energy=	0.610171
Sum of electronic and zero-point Energies=	-1705.786682
Sum of electronic and thermal Energies=	-1705.748816
Sum of electronic and thermal Enthalpies=	-1705.747872
Sum of electronic and thermal Free Energies=	-1705.858576

cartesian

8	0.25038970	-1.54128444	-0.52589613	1	-3.08721042	2.34221578	-2.03589606
6	1.55038965	-1.49038446	-0.25979611	8	-3.96351027	1.47891557	0.02840389
6	2.04138970	-1.90178442	1.02160394	6	-3.55441022	0.16831554	-0.34559610
6	3.38618970	-1.66128445	1.32230389	8	-2.88151026	-0.48128444	0.59690386
1	3.77998972	-1.92938447	2.29760385	6	-4.69181013	-0.64958447	-0.97319609
6	4.25868988	-1.09088445	0.39690387	12	-1.07961035	-0.25568444	-0.06869611
6	3.80318975	-0.83638448	-0.89599609	1	-5.21001053	-0.06388445	-1.74139607
1	4.51898956	-0.47988445	-1.63059604	1	5.29918957	-0.91248447	0.65930390
6	2.47238970	-1.05808449	-1.26819611	6	-4.86801052	1.53341556	1.13160396

6	1.14478970	-2.68048429	2.00710392	1	-4.53901052	0.83841556	1.91380394
6	2.04238963	-0.95208442	-2.74569607	6	-6.31771040	1.29591560	0.71040386
6	-0.09141032	-1.88158441	2.47390389	1	-4.76451015	2.54961562	1.52370393
1	-0.89181030	-1.89168441	1.73110390	1	-6.97701025	1.77911556	1.44270396
1	-0.52021033	-2.33808422	3.37380385	6	-6.75461054	-0.17248444	0.54540390
1	0.15808968	-0.84528446	2.72550392	1	-6.46301031	1.84231555	-0.22909610
6	0.66888970	-3.97908449	1.32170391	1	-4.21801043	-1.50208449	-1.46999609
1	0.11828968	-3.75778437	0.40500388	6	-5.67481041	-1.16488445	0.08390389
1	1.52198970	-4.61748409	1.06830394	1	-5.08111048	-1.50418448	0.93860388
1	0.00998968	-4.54408455	1.99260390	1	-6.17911053	-2.05678439	-0.30569613
6	1.91628969	-3.08808422	3.27440405	1	-7.59651041	-0.19698445	-0.15749609
1	2.79738975	-3.69548440	3.04440403	1	-7.15621042	-0.53948444	1.49780393
1	2.23938966	-2.22208428	3.86430383	8	0.05928968	1.24481559	0.70280391
1	1.26478970	-3.69228435	3.91470385	6	1.14918971	1.81891561	0.66840386
6	3.21938968	-0.56968445	-3.65929604	8	1.43688965	2.51341558	-0.42329612
1	3.61468959	0.42821556	-3.43489599	6	2.09968972	1.78121555	1.83130395
1	4.04348946	-1.28758442	-3.59569597	6	2.75248981	3.08281565	-0.64669609
1	2.87978959	-0.55708444	-4.70069599	6	2.42978978	3.15791559	2.43530393
6	1.53138971	-2.33438444	-3.20529604	6	3.04598975	4.29811573	0.21720390
1	0.68218970	-2.65698433	-2.59939599	1	2.71668959	3.36051559	-1.70069611
1	1.21378970	-2.29388428	-4.25479603	1	2.81878972	2.98181558	3.44340396
1	2.32318974	-3.08648443	-3.12059617	1	1.50578964	3.73481560	2.56270385
6	0.93418968	0.09231555	-2.98299599	1	3.01308966	1.26161551	1.51130390
1	1.19908965	1.06801558	-2.56409597	1	1.62688971	1.14341557	2.57770395
1	0.76808971	0.22291555	-4.05949593	6	3.45868969	3.96971560	1.65070391
1	-0.01091032	-0.23308444	-2.54619598	1	3.50038958	2.29251575	-0.52989614
8	-2.51511025	0.44661555	-1.40109611	1	2.16978979	4.95741558	0.20140390
6	-2.79721022	1.37281561	-2.44719601	1	3.85688972	4.85051584	-0.27229610
1	-3.59261036	0.99051553	-3.09539604	1	4.40668964	3.41641569	1.63060391
1	-1.88301027	1.47491550	-3.03479600	1	3.66058969	4.90341568	2.18740392

TS-23_CC

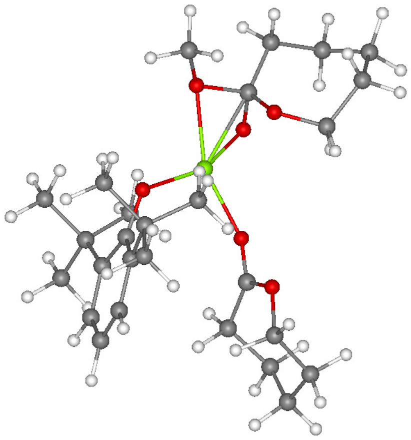

Zero-point vibrational energy	1788747.1 (Joules/Mol)
	427.52081 (Kcal/Mol)
Zero-point correction=	0.681298 (Hartree/Particle)
Thermal correction to Energy=	0.718427
Thermal correction to Enthalpy=	0.719371
Thermal correction to Gibbs Free Energy=	0.611018
Sum of electronic and zero-point Energies=	-1705.780691
Sum of electronic and thermal Energies=	-1705.743562
Sum of electronic and thermal Enthalpies=	-1705.742618
Sum of electronic and thermal Free Energies=	-1705.850971

cartesian

8	0.24478835	-1.67412949	-0.32297692	1	-3.45271158	-1.51352954	-2.20877695
6	1.54718840	-1.43452954	-0.26237693	8	-3.17851162	0.34757045	-0.68777692
6	2.27778840	-1.73972952	0.93122309	6	-3.53521156	-0.62462956	0.37402308
6	3.61268830	-1.33162951	1.01452315	8	-2.83101153	-0.37542954	1.45772302
1	4.18368816	-1.52022958	1.91822302	6	-5.04001188	-0.79232955	0.56262308
6	4.25228834	-0.68542951	-0.04217692	12	-1.18441164	-0.62062955	0.38092306
6	3.56978846	-0.51442951	-1.24617696	1	-5.53341198	-0.97352952	-0.39847693
1	4.11058807	-0.08632953	-2.08467698	1	5.29098797	-0.37612954	0.05032308
6	2.23468852	-0.90412956	-1.40257692	6	-3.56971169	1.70197046	-0.43477693
6	1.64438844	-2.59272933	2.04992318	1	-3.29821157	1.97757041	0.59212309
6	1.56468844	-0.87412953	-2.79167700	6	-5.04951191	1.94217050	-0.72647691
6	0.33978835	-1.99292958	2.62472296	1	-2.95571160	2.29797053	-1.11557698
1	-0.52971166	-2.25762939	2.01732302	1	-5.18411160	2.99567056	-1.00187695
1	0.13608834	-2.40432930	3.61992311	6	-6.03201199	1.60237050	0.40952307
1	0.39358833	-0.90442955	2.73692298	1	-5.29131174	1.36547041	-1.62707698
6	1.33308840	-3.99182940	1.47692311	1	-5.15571165	-1.69632959	1.16902304
1	0.65868837	-3.91782951	0.62072307	6	-5.66531181	0.39937046	1.29302311
1	2.25298834	-4.48992920	1.15282309	1	-4.96581173	0.70217049	2.07842302
1	0.85648835	-4.62072945	2.23932314	1	-6.57301188	0.06577048	1.80862308
6	2.60698843	-2.78162932	3.23542309	1	-7.02021170	1.43997049	-0.03827692
1	3.54128838	-3.26732945	2.93682313	1	-6.14401197	2.47607064	1.06302309
1	2.85348845	-1.83252954	3.72592306	8	-0.11161165	1.08457041	0.78702307
1	2.13558841	-3.42522931	3.98582315	6	0.87358832	1.80287051	0.62592310
6	2.52298832	-0.35772952	-3.87887692	8	0.89628834	2.57047057	-0.45817691
1	2.82088852	0.68407047	-3.71087694	6	1.98798847	1.86657047	1.63232303
1	3.42988849	-0.96622956	-3.95687699	6	2.08128834	3.30927062	-0.84657693
1	2.02058840	-0.39762953	-4.85157681	6	2.22078848	3.25917053	2.24472308
6	1.17138839	-2.31702948	-3.17457700	6	2.34998846	4.53097057	0.01702308

1	0.48208833	-2.74002934	-2.44107699	1	1.85418844	3.60627055	-1.87127697
1	0.68588835	-2.33132935	-4.15847683	1	2.77268839	3.11157060	3.17872310
1	2.05758834	-2.95882940	-3.22597694	1	1.25848842	3.70307064	2.52732301
6	0.31018835	0.01947048	-2.84337687	1	2.90358829	1.48577046	1.16032302
1	0.51898837	1.03187048	-2.48517704	1	1.72428846	1.15067041	2.41042304
1	-0.05021165	0.09747048	-3.87647700	6	3.00998831	4.22187042	1.35942304
1	-0.50371164	-0.39632952	-2.24847698	1	2.93148851	2.62047052	-0.87437695
8	-2.91061163	-1.82912958	-0.21447693	1	1.40648842	5.07177067	0.15862308
6	-3.37271166	-2.31852937	-1.47297692	1	3.00338840	5.19577074	-0.56037694
1	-4.34011173	-2.81902933	-1.35757697	1	4.00668812	3.79937053	1.17652309
1	-2.63431168	-3.04852939	-1.80907691	1	3.16958833	5.15917063	1.90412307

I-3_CC

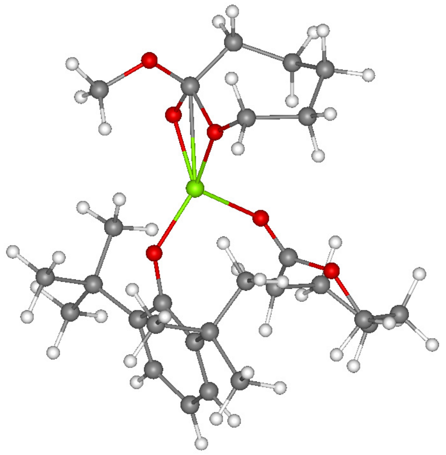

Zero-point vibrational energy	1789228.3 (Joules/Mol)
	427.63583 (Kcal/Mol)
Zero-point correction=	0.681481 (Hartree/Particle)
Thermal correction to Energy=	0.719233
Thermal correction to Enthalpy=	0.720177
Thermal correction to Gibbs Free Energy=	0.610751
Sum of electronic and zero-point Energies=	-1705.795191
Sum of electronic and thermal Energies=	-1705.757440
Sum of electronic and thermal Enthalpies=	-1705.756495
Sum of electronic and thermal Free Energies=	-1705.865921

cartesian

12	-1.32603967	-0.55938852	0.35671797	1	0.27376026	-3.02628875	3.33641791
8	-4.71623993	-1.63358855	0.46611795	1	-0.46963978	-2.26688862	1.95091796
6	-4.11123991	-2.89658856	0.21591796	8	-2.79233980	-0.95398855	1.56241798
1	-3.42143965	-2.85068870	-0.63568205	6	-3.83493972	-0.60638857	0.82461792
1	-4.92984009	-3.57638860	-0.02838204	6	-4.70974016	0.52411151	1.37811792

1	-3.57683969	-3.26008868	1.09751797	8	-3.13833976	-0.13808854	-0.49248204
8	-0.00773977	-1.66278851	-0.46178207	6	-4.15483999	1.94351149	1.23261797
6	1.30666029	-1.47548854	-0.49968204	1	-5.70673990	0.46231148	0.92911792
6	1.90356016	-0.80138850	-1.61558211	1	-4.82863998	0.27191147	2.43601799
6	3.25946021	-0.46368855	-1.53578210	6	-3.89053965	0.54091144	-1.49598205
1	3.73326039	0.07141146	-2.35298204	1	-3.07163978	1.94511151	1.40541792
6	4.04646015	-0.82498848	-0.44348204	1	-4.58614016	2.55911136	2.03121805
6	3.48826027	-1.61348855	0.56121790	1	-4.92884016	0.19221146	-1.44818211
1	4.13585997	-1.95308852	1.36291790	1	-3.48633981	0.22941145	-2.46498203
6	2.13806033	-1.97798848	0.55391794	6	-4.49064016	2.61881137	-0.10438204
6	1.11556029	-0.56578851	-2.92128205	1	-5.57764006	2.56281137	-0.25488204
6	1.59136033	-2.96728873	1.60391796	1	-4.25224018	3.68751144	-0.03338205
6	-0.10053977	0.36361146	-2.74048209	6	-3.78433967	2.05161142	-1.33808208
1	-0.88833976	-0.12358854	-2.16378212	1	-2.71853971	2.31041145	-1.31108212
1	-0.52963978	0.61281145	-3.71888208	1	-4.19553995	2.52441144	-2.23998213
1	0.17136024	1.30241144	-2.24838209	8	-0.24823976	1.09011149	0.85171795
6	0.62716019	-1.93038857	-3.45228195	6	0.79226023	1.74681151	0.77931792
1	-0.01243977	-2.42758870	-2.72028208	8	0.82596022	2.71761131	-0.12328205
1	1.47666025	-2.58598852	-3.67118216	6	1.95486021	1.51011145	1.69951797
1	0.05546023	-1.79538846	-4.37898207	6	2.03556037	3.47061133	-0.39678204
6	1.99446034	0.06441145	-4.01548195	6	2.31036019	2.70961142	2.59651804
1	2.86916018	-0.55048859	-4.25058174	6	2.40736032	4.44751120	0.70651793
1	2.34226036	1.06831145	-3.74498200	1	1.78336024	4.00291157	-1.31458211
1	1.40946031	0.16221146	-4.93638182	1	2.90166020	2.32081127	3.43161798
6	2.69586039	-3.44798875	2.56231785	1	1.39566028	3.12281132	3.03861785
1	3.10186028	-2.63328862	3.17311788	1	2.81986022	1.19611144	1.10011792
1	3.52486038	-3.92758870	2.03181791	1	1.67886031	0.65101147	2.30941796
1	2.27756023	-4.19068861	3.25031781	6	3.11096025	3.81011128	1.90301788
6	1.04066026	-4.21328878	0.87841791	1	2.84346032	2.76741147	-0.62218207
1	0.24966024	-3.94058871	0.17741796	1	1.50166023	4.98011160	1.02041793
1	0.63056022	-4.92578840	1.60491788	1	3.06866026	5.19961119	0.26011795
1	1.83736026	-4.71878862	0.32201797	1	4.07066011	3.39391136	1.57021797
6	0.47646022	-2.36618853	2.48481798	1	3.35196018	4.59041119	2.63341784
1	0.76066023	-1.39088857	2.89521790	1	5.09935999	-0.55408859	-0.40948203

TS-34_CC

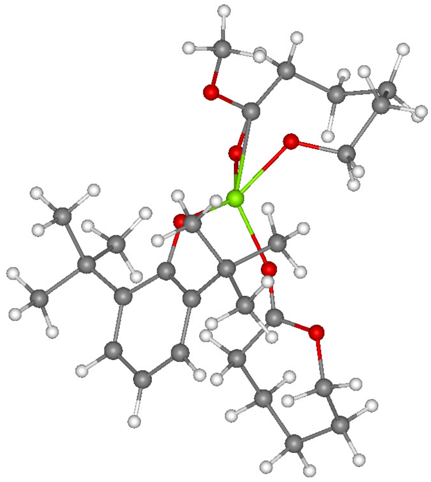

Zero-point vibrational energy	1786096.1 (Joules/Mol)
	426.88722 (Kcal/Mol)
Zero-point correction=	0.680288 (Hartree/Particle)
Thermal correction to Energy=	0.717896
Thermal correction to Enthalpy=	0.718841
Thermal correction to Gibbs Free Energy=	0.609286
Sum of electronic and zero-point Energies=	-1705.777674
Sum of electronic and thermal Energies=	-1705.740065
Sum of electronic and thermal Enthalpies=	-1705.739121
Sum of electronic and thermal Free Energies=	-1705.848676

cartesian

8	0.05624477	-1.57122314	-0.39618218	1	-4.37425518	-1.57902312	-1.53418219
6	1.37754476	-1.45982313	-0.42278218	8	-2.96445537	0.19687694	-0.64928216
6	2.17114472	-1.98902309	0.64811784	6	-3.60495520	-0.69972306	0.84801787
6	3.53784466	-1.69412315	0.66631782	8	-2.68215537	-0.44902307	1.69331777
1	4.15784454	-2.05532289	1.48061776	6	-5.01335526	-0.20202306	1.10591781
6	4.14734459	-0.94912302	-0.34208214	12	-1.25645530	-0.36332306	0.27131784
6	3.39364481	-0.57122302	-1.45228219	1	-5.68015528	-0.45692307	0.27911782
1	3.90454483	-0.07602306	-2.27248216	1	5.21164465	-0.72922307	-0.29788220
6	2.02414465	-0.84532303	-1.54648221	6	-3.21575522	1.56867695	-0.91658217
6	1.56134474	-2.92422295	1.71291780	1	-2.77495527	2.21737695	-0.14088216
6	1.27204478	-0.61362302	-2.87488222	6	-4.70205545	1.88307691	-1.07418227
6	0.53204477	-2.21162295	2.61091781	1	-2.69615531	1.80627692	-1.85488224
1	-0.37365526	-1.93732309	2.06961775	1	-4.81645536	2.74487710	-1.74368227
1	0.21684477	-2.87582302	3.42471790	6	-5.44525528	2.18587708	0.23511782
1	0.95464474	-1.31182313	3.07161784	1	-5.16145515	1.03587687	-1.59728217
6	0.88294476	-4.12022305	1.01161778	1	-5.34085512	-0.79462302	1.96971774
1	0.09744477	-3.78632307	0.33181781	6	-5.08565521	1.29307687	1.43031776

1	1.61744475	-4.69512320	0.43711782	1	-4.12705517	1.59347689	1.86421776
1	0.43584475	-4.79092312	1.75561774	1	-5.83135509	1.45707691	2.21591783
6	2.63744473	-3.50162292	2.64991784	1	-6.52445507	2.12237692	0.04461783
1	3.41394472	-4.04512310	2.10171771	1	-5.25455523	3.22717690	0.52411783
1	3.12154484	-2.72942305	3.25911784	8	-0.09695524	1.24047685	0.77591783
1	2.16844463	-4.20982313	3.34151769	6	0.98724478	1.82047689	0.70571786
6	2.20404482	-0.06312306	-3.96858239	8	1.11084473	2.75087690	-0.23328216
1	2.58974481	0.93377697	-3.72438216	6	2.10854483	1.54397690	1.66541779
1	3.05534482	-0.72442305	-4.15958214	6	2.37964463	3.39537692	-0.51068217
1	1.64584470	0.02707694	-4.90678215	6	2.53144479	2.75407696	2.51761770
6	0.73364478	-1.97232306	-3.37228227	6	2.80394483	4.39097691	0.55671787
1	0.05994476	-2.41722298	-2.63718224	1	2.18824482	3.90377712	-1.45628226
1	0.18374476	-1.84152305	-4.31278181	1	3.07084465	2.36207700	3.38581777
1	1.55724478	-2.67072296	-3.55558228	1	1.64034474	3.25537705	2.91441774
6	0.09504475	0.37497693	-2.75848222	1	2.96204472	1.14247692	1.10251784
1	0.39314476	1.31187689	-2.27808213	1	1.75554478	0.73607695	2.30501771
1	-0.28235525	0.62177694	-3.75858212	6	3.43004465	3.75827694	1.79781783
1	-0.74295521	-0.05692307	-2.20898223	1	3.13574481	2.62367702	-0.68698215
8	-3.47085524	-1.95982313	0.29901785	1	1.93604469	5.00587654	0.82391787
6	-4.32905531	-2.35782290	-0.76918215	1	3.53104472	5.06857681	0.09331783
1	-5.33185530	-2.60052299	-0.40168220	1	4.36224461	3.25577712	1.50871778
1	-3.87985539	-3.25822306	-1.19058216	1	3.71384478	4.55097675	2.49901772

I-4_CC

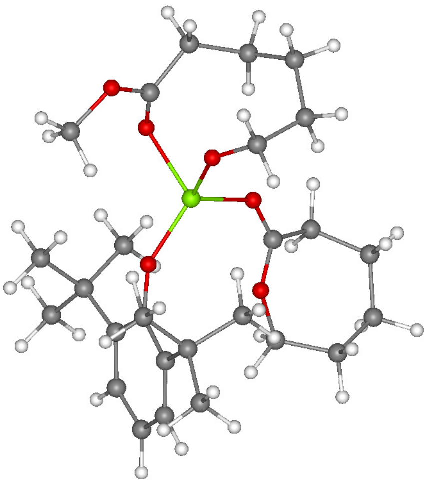

Zero-point vibrational energy	1785379.5 (Joules/Mol)
	426.71594 (Kcal/Mol)
Zero-point correction=	0.680015 (Hartree/Particle)
Thermal correction to Energy=	0.718940
Thermal correction to Enthalpy=	0.719884
Thermal correction to Gibbs Free Energy=	0.606721

Sum of electronic and zero-point Energies= -1705.795516
Sum of electronic and thermal Energies= -1705.756591
Sum of electronic and thermal Enthalpies= -1705.755647
Sum of electronic and thermal Free Energies= -1705.868810

cartesian

12	-1.31694233	-0.37061539	0.06525776	1	0.59645766	-1.11561537	3.93375778
8	-4.11644220	-2.53401542	0.44225776	1	0.16045766	-0.56181538	2.31735778
6	-3.08504224	-3.52941537	0.32925773	8	-2.63994241	-1.15431535	1.43725777
1	-2.35734224	-3.21721530	-0.42234224	6	-3.75714231	-1.34901536	0.92015779
1	-3.59404230	-4.43581533	0.00475775	6	-4.93104267	-0.41631538	1.03005779
1	-2.59114242	-3.68531537	1.28935766	8	-2.81434226	-0.38061538	-1.08194232
8	0.29085767	-1.35411537	-0.04504225	6	-4.64674234	1.09118462	0.94165778
6	1.60835767	-1.38961542	-0.16484225	1	-5.68024254	-0.72811538	0.29905778
6	2.23075771	-1.23641539	-1.44854224	1	-5.35644245	-0.65131539	2.01625776
6	3.62595773	-1.14701545	-1.50544226	6	-3.49894238	0.36258462	-2.03124213
1	4.11855745	-1.00551534	-2.46184206	1	-3.60854220	1.29068458	1.22495770
6	4.42395735	-1.25001538	-0.36894226	1	-5.26184273	1.57818460	1.70725775
6	3.81735778	-1.51241541	0.85625780	1	-4.39934254	-0.18531539	-2.36784220
1	4.45565748	-1.65471542	1.72255766	1	-2.88744235	0.51458460	-2.93974209
6	2.42895770	-1.61201537	0.99135774	6	-4.98244238	1.75928462	-0.40544224
6	1.41225767	-1.20921540	-2.75604224	1	-5.91834259	1.33058465	-0.79064220
6	1.81855774	-1.99531543	2.35415792	1	-5.21224260	2.81228471	-0.19624224
6	0.56825763	0.07378461	-2.87534213	6	-3.92484236	1.73968458	-1.51574230
1	-0.19644235	0.14078462	-2.10044217	1	-3.02454233	2.26438451	-1.16584229
1	0.04755765	0.09988461	-3.84004211	1	-4.31594276	2.32548451	-2.36054206
1	1.20115769	0.96528459	-2.80724216	8	-0.88794237	1.52528465	0.82615775
6	0.50065762	-2.45101547	-2.83864212	6	0.17585765	2.15278459	0.88395774
1	-0.18944235	-2.49651551	-1.99614227	8	1.19855773	1.69108462	0.19675776
1	1.10365772	-3.36561537	-2.83794212	6	0.28685766	3.42208457	1.68575776
1	-0.08524235	-2.43131542	-3.76554227	6	2.54085779	2.23448467	0.29915774
6	2.31595778	-1.24731541	-4.00314236	6	0.65885764	4.65878439	0.84745777
1	2.97025776	-2.12531543	-4.01004219	6	2.68005776	3.59928465	-0.35244226
1	2.94025779	-0.35171539	-4.09834194	1	3.13985777	1.47878456	-0.20944224
1	1.68835771	-1.29841542	-4.89964199	1	0.38035762	5.54238462	1.43105769
6	2.89535761	-2.35411549	3.39385772	1	0.03975765	4.68448448	-0.05704224
1	3.53945780	-1.50351536	3.64385772	1	1.02575767	3.27288461	2.48315787
1	3.53195763	-3.18001533	3.05975795	1	-0.68324238	3.55868459	2.16525793
1	2.40715766	-2.67161536	4.32215786	6	2.14005780	4.76078463	0.48205775
6	0.92315763	-3.24171543	2.19795775	1	2.83755779	2.24498463	1.35305774
1	0.11915766	-3.06381536	1.48345768	1	2.19925761	3.56588459	-1.33704233

1	0.48095763	-3.51541543	3.16415787	1	3.74875760	3.75808454	-0.53714222
1	1.51495767	-4.09321547	1.84435773	1	2.72835779	4.83808470	1.40625775
6	1.00145769	-0.83561540	2.95365787	1	2.30405760	5.69788456	-0.06144224
1	1.63455772	0.04908461	3.09485793	1	5.50635767	-1.17701542	-0.44584227

TS-45_CC

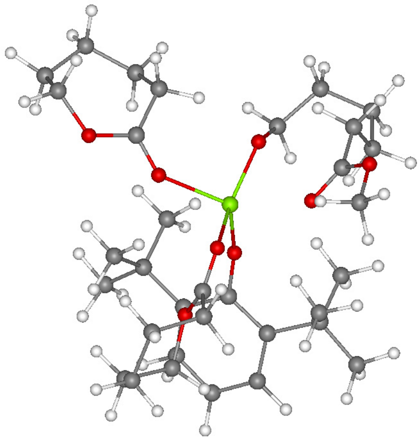

Zero-point vibrational energy	2202689.7 (Joules/Mol)
	526.45548 (Kcal/Mol)
Zero-point correction	0.838960 (Hartree/Particle)
Thermal correction to Energy	0.886166
Thermal correction to Enthalpy	0.887110
Thermal correction to Gibbs Free Energy	0.754432
Sum of electronic and zero-point Energies	-2090.658608
Sum of electronic and Thermal Energies	-2090.611402
Sum of electronic and Thermal Enthalpies	-2090.610457
Sum of electronic and Thermal Free Energies	-2090.743136

cartesian

8	0.70412910	-0.07527923	1.06377423	1	-0.63107091	5.26272058	1.27927423
6	1.82382905	-0.64457923	1.47887421	1	-0.53757089	3.50122070	1.12777424
6	1.86502910	-2.04957914	1.81007421	6	-3.06387091	2.92782068	2.33197427
6	3.10332918	-2.64157915	2.09337425	6	-2.17767096	4.18692064	2.34927416
1	3.14902902	-3.70697927	2.31467414	1	-3.87237096	3.03302073	1.58847427
6	4.28932905	-1.91067922	2.11867428	12	-0.63257092	0.50382078	-0.19302575
6	4.22962904	-0.53097922	1.92987418	1	-2.65407085	3.54242086	-0.24062575
1	5.15012932	0.04312077	2.02547431	1	-2.50997090	5.33692074	-0.34792575
6	3.03002906	0.13342077	1.64537418	1	5.23942900	-2.40107918	2.33437419
6	0.58752912	-2.91887927	1.86227417	1	-3.55997086	2.84952068	3.31527424
6	3.02282906	1.67692077	1.56157422	1	-2.82017088	5.07982063	2.44397426
6	0.07322913	-3.19937921	0.43377426	1	-1.55317092	4.16482067	3.25807428

1	-0.10647088	-2.27867913	-0.12752575
1	-0.86717087	-3.77427912	0.46567425
1	0.81082910	-3.79497910	-0.12552576
6	-0.50967085	-2.23287916	2.70977426
1	-0.76127088	-1.24047923	2.32277417
1	-0.16937086	-2.11447930	3.74907422
1	-1.41697085	-2.85827923	2.72387433
6	0.84032911	-4.29237938	2.51957417
1	1.24762905	-4.19117928	3.53547430
1	1.52582908	-4.92027950	1.93257427
1	-0.11497088	-4.83377934	2.59227419
6	4.37752914	2.29232073	1.96867418
1	5.18782902	2.01672077	1.27767420
1	4.67472887	1.99732077	2.98487425
1	4.28952932	3.38882089	1.95077419
6	1.96852911	2.24642086	2.53947425
1	0.97392911	1.83862078	2.33017421
1	1.92862916	3.34422088	2.45977426
1	2.23482919	1.99102080	3.57557416
6	2.73502922	2.16752076	0.12637424
1	3.50082922	1.78912079	-0.56902575
1	2.75852919	3.26782084	0.08527425
1	1.75262916	1.84552085	-0.22862574
8	-0.96197093	5.22452068	-2.21242571
6	-0.05207087	5.05812073	-3.32452583
1	-0.32717088	4.17622042	-3.91722584
1	-0.15127087	5.97182035	-3.91912580
1	0.97762913	4.93942070	-2.96402574
8	-0.30797088	3.17542076	-1.49242580
6	-0.99697089	4.17492056	-1.34032583
8	-2.08487082	1.41472077	0.66647422
6	-1.96017087	4.39722061	-0.20732576
6	-2.33467078	1.60282075	2.03517413
6	-1.23427093	4.34882069	1.14997423
1	-1.40297091	1.56862080	2.64277411
1	-2.97247100	0.78012079	2.43727422
8	-1.85177088	-0.95287919	-1.20672584
6	-3.02797079	-1.32247925	-1.18362582
6	-4.08907080	-0.66807920	-0.33992577
8	-3.32077098	-2.38067913	-1.96602583
6	-4.60017109	-1.57107925	0.80517423
1	-4.93307066	-0.36867923	-0.98432577
1	-3.60387087	0.24302077	0.06207424
6	-4.67177105	-2.93207932	-2.00302577
1	-3.74057078	-2.03897929	1.31037426
1	-5.07017088	-0.91677922	1.55337417
1	-5.37817097	-2.14397931	-2.30752587
1	-4.60617113	-3.66657925	-2.81452584
6	-5.61217070	-2.63637924	0.37157425
1	-6.51627111	-2.13227916	-0.01202575
1	-5.93277073	-3.21337914	1.25277424
6	-5.09987068	-3.60237932	-0.70222580
1	-4.25407076	-4.20017958	-0.32542574
1	-5.89807081	-4.31957960	-0.95412576
8	0.36852914	0.46722075	-2.06332564
6	1.40732908	-0.09357923	-2.42722583
6	2.02432919	0.19772077	-3.77482581
8	1.95822906	-0.98467922	-1.60702574
6	2.10482907	-1.02877915	-4.70922565
1	3.03352904	0.61522079	-3.61872578
1	1.40872908	0.98732078	-4.22042561
6	3.26302910	-1.59367919	-1.86402583
1	1.14712906	-1.57277918	-4.67942572
1	2.21472907	-0.65447921	-5.73792553
1	4.00082922	-0.79387921	-2.03062582
1	3.49092913	-2.07357931	-0.90472579
6	3.26742911	-1.98087931	-4.40552568
1	4.21632910	-1.43117917	-4.53172541
1	3.27612901	-2.78487921	-5.15762568
6	3.23952913	-2.60157919	-3.00452590
1	2.35722899	-3.24957919	-2.87792587
1	4.12092924	-3.25057912	-2.87892580

I-5_CC

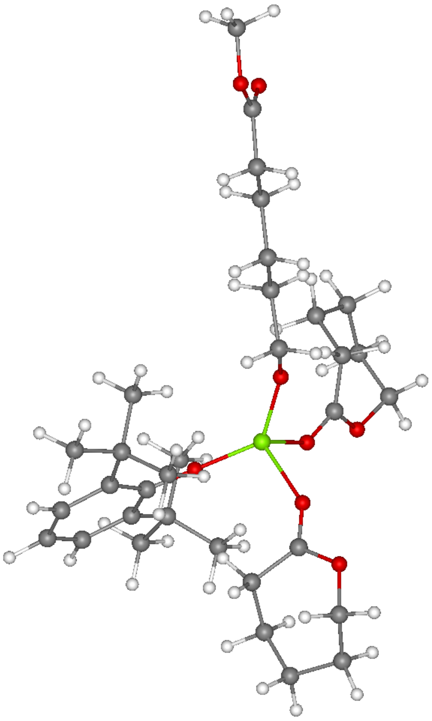

Zero-point vibrational energy	2200979.7 (Joules/Mol)
	526.04676 (Kcal/Mol)
Zero-point correction=	0.838309 (Hartree/Particle)
Thermal correction to Energy=	0.887169
Thermal correction to Enthalpy=	0.888113
Thermal correction to Gibbs Free Energy=	0.747361
Sum of electronic and zero-point Energies=	-2090.672967
Sum of electronic and thermal Energies=	-2090.624107
Sum of electronic and thermal Enthalpies=	-2090.623163
Sum of electronic and thermal Free Energies=	-2090.763914

cartesian

8	-1.25303173	0.46260831	-0.91945100	6	3.85036826	-1.57279170	3.39174891
6	-2.31623173	-0.32219172	-1.34725094	1	4.65946817	-1.17689168	3.99624896
6	-3.63003159	0.06570829	-0.66225100	6	3.15536833	-0.72649169	2.52264905
6	-4.82033205	-0.79089171	-1.09295106	6	0.49376822	-3.25419188	1.19834900
6	-6.13663197	-0.37349170	-0.43515098	6	3.50556827	0.77320832	2.47344899
6	-7.30663204	-1.25789165	-0.85415095	6	0.68556821	-3.28739166	-0.33005098
6	-8.63063145	-0.83559173	-0.26265097	1	0.85546821	-2.28999186	-0.73745096
8	-9.58473110	-1.76839173	-0.49635097	1	-0.20013174	-3.70659161	-0.82265097
1	-2.14063168	-1.39809167	-1.15685105	1	1.54656827	-3.91279173	-0.59415096
1	-2.45823169	-0.23199171	-2.44215107	6	-0.77013177	-2.44969177	1.56464911
1	-3.48663163	-0.00929171	0.42344904	1	-0.69683176	-1.40429175	1.26234913

1	-3.83713174	1.12320828	-0.87745100	1	-0.93383175	-2.47749186	2.64744902
1	-4.61243200	-1.84419167	-0.85795099	1	-1.65303171	-2.88319159	1.07914901
1	-4.92963171	-0.74169171	-2.18585110	6	0.22676824	-4.70809174	1.62934899
6	-10.88983154	-1.44729173	0.00434902	1	0.04166825	-4.79279137	2.70524907
12	0.57596827	0.37700829	-0.56125098	1	1.05096829	-5.38069153	1.36754894
8	1.06506824	2.38550806	-0.53875101	1	-0.66793174	-5.07649136	1.11474895
6	0.46066827	3.45830822	-0.55155098	6	4.59316826	1.15640831	3.49334908
6	-1.03763175	3.55460835	-0.57595098	1	5.54676819	0.65420830	3.29634905
8	1.21646833	4.55430841	-0.52225101	1	4.29316807	0.93280828	4.52264929
6	-1.62233174	4.11240864	0.73474902	1	4.77766800	2.23520851	3.43304896
1	-1.34573174	4.18380833	-1.42095089	6	2.26036835	1.61260831	2.82554889
1	-1.39723170	2.53350830	-0.75925100	1	1.42876828	1.39430833	2.15494895
6	0.63196826	5.87720823	-0.49725097	1	2.49066830	2.68340826	2.75934887
1	-1.13893175	3.62210846	1.58774900	1	1.93516827	1.39760828	3.84944892
1	-2.67433167	3.81350851	0.77374899	6	4.04736805	1.16900826	1.08664894
1	-0.00253175	6.01190853	-1.38015103	1	4.97326803	0.62250829	0.87244904
1	1.50166833	6.52620840	-0.60745096	1	4.27406788	2.24180841	1.05214906
6	-1.53173172	5.63130856	0.86954904	1	3.32676840	0.95480829	0.29784903
1	-2.13903165	6.09420824	0.07974902	8	1.67166829	-0.03809170	-2.27645111
1	-1.98383164	5.93690825	1.81974912	6	2.59546828	-0.70059168	-2.74295115
6	-0.11243175	6.19640827	0.79124904	8	2.68436837	-0.72869170	-4.07065105
1	0.49536824	5.85000849	1.63574910	6	3.59966826	-1.42799175	-1.89015102
1	-0.15833175	7.28900862	0.87234902	6	3.68036819	-1.52029169	-4.75895071
1	-6.36563206	0.66840827	-0.68395096	6	5.03696823	-0.89369172	-2.03445101
1	-6.03283215	-0.40499172	0.65564901	6	5.09836817	-0.99479175	-4.60405064
1	-7.13383198	-2.30629158	-0.58665097	1	3.35866833	-1.45909166	-5.79925060
1	-7.42783213	-1.25289166	-1.94535089	1	5.58996820	-1.22489166	-1.15055108
8	-8.85013103	0.19820829	0.33494902	1	5.02446795	0.20120829	-1.99075103
1	-11.52273178	-2.29269171	-0.26415098	1	3.57056832	-2.49759150	-2.13305116
1	-10.86513138	-1.31809175	1.08874893	1	3.25776839	-1.32359171	-0.85955095
1	-11.26413155	-0.52939171	-0.45475098	6	5.77266788	-1.37129176	-3.28545094
8	1.49596834	-0.52699172	0.81274903	1	3.59416819	-2.56529188	-4.44165087
6	2.10396838	-1.27949166	1.71854901	1	5.08116817	0.09320830	-4.74055099
6	1.72176826	-2.64909172	1.90644908	1	5.68516827	-1.40139174	-5.43645096
6	2.46516824	-3.43399191	2.79384899	1	5.87846804	-2.46359158	-3.24065113
1	2.20576835	-4.47759151	2.93744898	1	6.79066801	-0.96629167	-3.27635098
6	3.53486824	-2.92119169	3.51984906	1	4.09686804	-3.55799150	4.19894934

S1.4. PP sequence, mononuclear mechanism

I-1_PP

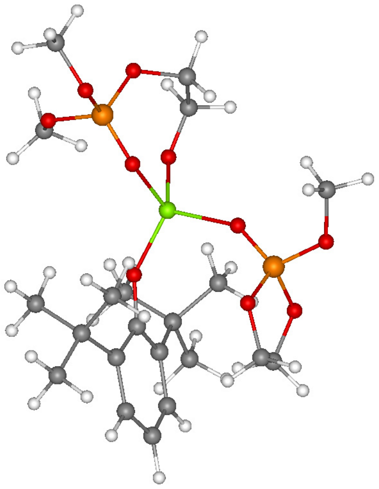

Zero-point vibrational energy	1565337.6 (Joules/Mol)
	374.12466 (Kcal/Mol)
Zero-point correction=	0.596206 (Hartree/Particle)
Thermal correction to Energy=	0.637320
Thermal correction to Enthalpy=	0.638265
Thermal correction to Gibbs Free Energy=	0.519129
Sum of electronic and zero-point Energies=	-2457.219233
Sum of electronic and thermal Energies=	-2457.178118
Sum of electronic and thermal Enthalpies=	-2457.177174
Sum of electronic and thermal Free Energies=	-2457.296310

cartesian

12	1.04106259	0.46429029	-0.51320416	6	-0.06723739	-2.78090978	2.05299592
8	4.91156292	0.12579030	1.73039579	1	-0.61943740	-3.70210981	1.83579588
8	-0.31403738	-0.82090962	-0.21170413	1	0.64906263	-2.99660969	2.85539603
6	6.29556274	-0.26580968	1.72299588	1	0.48886263	-2.49670959	1.15899587
1	6.69126272	0.01549029	2.69829607	1	6.38236284	-1.34560966	1.58329582
1	6.83856249	0.26389030	0.93609583	8	2.61926270	0.40089029	0.77769583
6	-1.59023738	-1.07430971	0.01999586	15	3.99266267	-0.09800971	0.45569587
6	-2.53813720	-1.09500968	-1.05890417	8	4.71986294	0.61379027	-0.76440418
6	-3.89733744	-1.21540964	-0.74570417	8	2.13116264	0.34819031	-2.06280398
1	-4.63643742	-1.20110965	-1.53980422	6	4.10096264	1.66549027	-1.56730413
6	-4.34753752	-1.36200964	0.56409585	6	3.16106272	1.07349026	-2.61140394
6	-3.41013718	-1.47650969	1.58849585	1	4.94266272	2.19369030	-2.02360415
1	-3.77173710	-1.67380965	2.59279585	1	3.56926274	2.34689021	-0.89510417
6	-2.03503728	-1.37450969	1.35289586	1	2.81276274	1.93389034	-3.21850395

6	-2.09403729	-1.04770970	-2.53580403	1	3.77196264	0.45199031	-3.29300404
6	-1.03533745	-1.66200972	2.49179602	8	4.15866280	-1.65220964	0.19549584
6	-1.08473742	-2.18080974	-2.81260395	1	-5.41103745	-1.44600964	0.77579582
1	-0.20483740	-2.09350967	-2.17450404	6	3.75476265	-2.29200959	-1.04470420
1	-0.75613737	-2.14680958	-3.85840416	1	3.04986262	-1.66840971	-1.60130417
1	-1.54763734	-3.15780973	-2.63390398	1	3.29206276	-3.23720980	-0.76170415
6	-1.47173738	0.30949029	-2.92030406	1	4.65366268	-2.47220969	-1.63720417
1	-0.48333740	0.45689029	-2.48410416	8	0.26696262	2.31459022	-0.12050415
1	-2.11753726	1.14269030	-2.62430406	15	-0.92173743	3.08829021	0.33689585
1	-1.33363736	0.36309028	-4.00710392	8	-2.28433728	2.97299027	-0.47570416
6	-3.28023720	-1.26570964	-3.49430394	8	-1.45113742	2.65049028	1.78199589
1	-4.01353741	-0.45250973	-3.44590402	6	-3.29463720	2.30009031	0.32909587
1	-3.79953718	-2.21080971	-3.30380416	6	-2.89643717	2.56189036	1.78299582
1	-2.90643740	-1.29980969	-4.52360392	8	-0.63553739	4.64799023	0.32499588
6	-1.73983741	-2.16340971	3.76529598	6	0.69686264	5.16429043	0.53309584
1	-2.33063722	-3.06740975	3.58289599	1	1.41316259	4.62679052	-0.08940414
1	-2.39683723	-1.40670967	4.20929575	1	0.96996260	5.07759047	1.58709586
1	-0.98373735	-2.41200972	4.51829576	1	0.65566260	6.21339035	0.24409586
6	-0.23563738	-0.40990970	2.89459586	1	-3.17413735	1.73489034	2.43549585
1	0.35836262	-0.02070969	2.06919599	1	-3.29583716	3.50559044	2.16549587
1	0.45746261	-0.65010965	3.71009588	1	-4.25633717	2.74199033	0.06929585
1	-0.89983737	0.38719028	3.24399590	1	-3.28363729	1.23719025	0.08139586

TS-12_PP

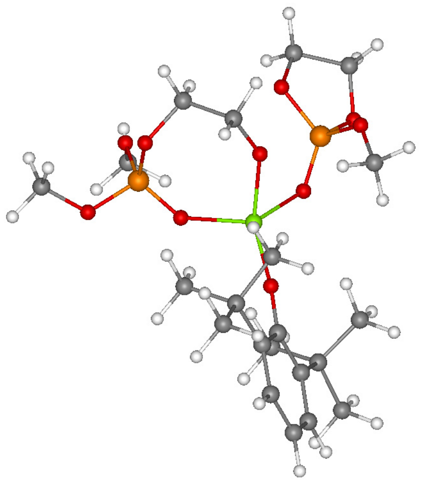

Zero-point vibrational energy	1565406.4 (Joules/Mol)
	374.14112 (Kcal/Mol)
Zero-point correction=	0.596232 (Hartree/Particle)
Thermal correction to Energy=	0.636346
Thermal correction to Enthalpy=	0.637290
Thermal correction to Gibbs Free Energy=	0.521406
Sum of electronic and zero-point Energies=	-2457.215883

Sum of electronic and thermal Energies= -2457.175769

Sum of electronic and thermal Enthalpies= -2457.174824

Sum of electronic and thermal Free Energies= -2457.290708

cartesian

12	-0.52497226	-0.17979024	-0.12329715	6	1.74002779	2.58320999	0.97760284
8	-1.52607226	3.78060985	-1.48109710	1	2.36912775	3.24040985	0.36730283
8	1.32692766	-0.28209025	-0.04479715	1	1.15312767	3.21320987	1.65830290
6	-2.17277217	5.04501009	-1.23559713	1	1.05622768	2.07070994	0.29970282
1	-1.55597234	5.79461002	-1.72909713	1	-3.17937207	5.05160999	-1.66159713
1	-2.21477222	5.24060965	-0.16279715	8	-1.54717231	-1.89929020	-0.48859715
6	2.65212774	-0.23429024	-0.07189716	15	-2.66597223	-2.24179006	0.45280284
6	3.38422775	-1.07299030	-0.97249711	8	-3.40027213	-3.54679012	-0.20009714
6	4.78042746	-1.00739026	-0.96089715	8	-4.02247238	-1.35909021	0.58510286
1	5.35512781	-1.63579023	-1.63339710	6	-4.78337240	-3.55689001	0.14750285
6	5.47362757	-0.15549025	-0.10729715	6	-5.18627262	-2.08489013	0.13610286
6	4.75622749	0.66180974	0.75950289	1	-4.91617250	-4.00729036	1.13770282
1	5.31192780	1.32280970	1.41640282	1	-5.31997252	-4.14729023	-0.59679711
6	3.35882783	0.65200973	0.80300289	1	-5.43677235	-1.73999023	-0.87139714
6	2.66772771	-2.03529024	-1.93849719	1	-6.00477219	-1.85739028	0.82110286
6	2.61422777	1.59060979	1.77120292	8	-2.29977226	-2.77099013	1.90330291
6	1.76542771	-1.24719024	-2.91059732	6	-0.97427225	-2.69909024	2.47160268
1	1.05002773	-0.61489022	-2.38289714	1	-0.22947228	-3.05059004	1.75500286
1	1.20802772	-1.93279028	-3.56089735	1	-0.76527226	-1.67189026	2.76710272
1	2.37132788	-0.59329027	-3.54749727	1	-0.99397230	-3.36129022	3.33700275
6	1.84472764	-3.07219005	-1.14809716	8	-1.23447227	1.29740977	-1.30989718
1	1.10632765	-2.59329009	-0.50549716	15	-2.17387223	2.41730976	-1.00219715
1	2.50552773	-3.67749023	-0.51719713	8	-2.56817222	2.69550991	0.49400285
1	1.31592774	-3.74739003	-1.83199716	8	-1.81887233	-0.08349024	1.30320287
6	3.65212798	-2.83289003	-2.81189728	6	-3.36147213	1.78760970	1.33310282
1	4.31552744	-3.46729016	-2.21429729	6	-2.47447228	0.82770973	2.10920286
1	4.27032757	-2.18389010	-3.44129729	1	-4.06287241	1.24480975	0.69690287
1	3.08582783	-3.49229002	-3.47989726	1	-3.91117215	2.45500994	2.00170279
6	3.57382774	2.43960977	2.62310266	1	-1.76767230	1.42110980	2.71360278
1	4.20922756	3.08850980	2.01130271	1	-3.14887214	0.32020974	2.82270265
1	4.22012758	1.82330978	3.25710273	8	-3.58397222	2.25010991	-1.73679709
1	2.98872781	3.08820987	3.28560281	6	-3.63007212	1.68810976	-3.06689715
6	1.75452769	0.77990973	2.76230264	1	-3.20187211	2.38930988	-3.78739715
1	1.01782775	0.16180976	2.24810266	1	-3.08767223	0.74170977	-3.09869719
1	1.22522771	1.45250976	3.44960284	1	6.56072760	-0.12759024	-0.11899716
1	2.38882780	0.11770976	3.36220264	1	-4.68467236	1.52890980	-3.28849721

I-2_PP

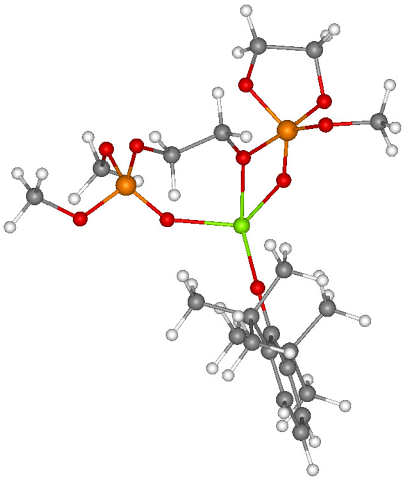

Zero-point vibrational energy	1568556.2 (Joules/Mol)
	374.89393 (Kcal/Mol)
Zero-point correction=	0.597432 (Hartree/Particle)
Thermal correction to Energy=	0.637567
Thermal correction to Enthalpy=	0.638511
Thermal correction to Gibbs Free Energy=	0.521551
Sum of electronic and zero-point Energies=	-2457.215133
Sum of electronic and thermal Energies=	-2457.174997
Sum of electronic and thermal Enthalpies=	-2457.174053
Sum of electronic and thermal Free Energies=	-2457.291014

cartesian

12	-0.47606266	-0.23479718	-0.38737077	6	1.81143737	-2.51239705	-2.02967072
8	-1.57166266	3.96430278	-0.85387081	1	2.43263745	-3.32379723	-1.63487077
8	1.34773731	-0.14809719	-0.10777077	1	1.27093732	-2.89569712	-2.90347075
6	-2.33476257	5.14460278	-0.53717077	1	1.07743740	-2.26639724	-1.26197076
1	-1.65846264	5.98560238	-0.68187076	1	-3.19396257	5.23730278	-1.20627069
1	-2.67326260	5.11390257	0.50102919	8	-1.57836270	-1.81929719	-0.78307080
6	2.67413735	-0.19149718	-0.07857077	15	-2.66296268	-1.92169714	0.29882926
6	3.37373734	0.32940280	1.05532932	8	-3.39906263	-3.32069707	-0.31647074
6	4.76963758	0.25970283	1.07052922	8	-4.10376263	-1.11669719	0.27312922
1	5.32083750	0.64420283	1.92242932	6	-4.78666258	-3.33379722	-0.06447077
6	5.49023724	-0.29149717	0.01622923	6	-5.20756245	-1.87939715	-0.22137077
6	4.80353737	-0.78299713	-1.08907068	1	-5.00706244	-3.69749713	0.95092922
1	5.38213730	-1.20449710	-1.90447068	1	-5.27376270	-3.99249721	-0.78797078
6	3.40833735	-0.74899715	-1.17277074	1	-5.36786270	-1.61799717	-1.27257073
6	2.61933732	0.97030282	2.23452926	1	-6.09146261	-1.61339712	0.36322922
6	2.69663739	-1.31049716	-2.41767073	8	-2.28846264	-2.54669714	1.75832927
6	1.67213726	-0.05039717	2.89742923	6	-2.06786251	-3.95279717	1.94012928

1	0.94883740	-0.45749718	2.19002938	1	-3.00696254	-4.51109743	1.92112923
1	1.12673736	0.41680282	3.72822928	1	-1.39666259	-4.34999752	1.17602932
1	2.24403739	-0.89069718	3.30572939	1	-1.60306263	-4.04949760	2.92282939
6	1.83453727	2.20370293	1.74262929	8	-1.17306256	1.52170289	-1.10807073
1	1.15783739	1.94630289	0.92522919	15	-2.22626257	2.51640296	-0.74887079
1	2.52183723	2.97060275	1.36902928	8	-2.91696262	2.39860296	0.66722924
1	1.25713730	2.64590287	2.56632924	8	-1.99426270	-0.32629719	0.96792918
6	3.56783724	1.46570289	3.34052920	6	-2.22866249	1.88070285	1.84442925
1	4.26293755	2.23160291	2.98052931	6	-2.53346276	0.41120282	2.04002929
1	4.15163755	0.65010285	3.78002930	1	-2.61276269	2.46820283	2.68122935
1	2.97723746	1.91480279	4.14752913	1	-1.15336263	2.05930281	1.75622928
6	3.68533754	-1.82619715	-3.47867084	1	-2.09126282	0.09190283	2.99382925
1	4.29543734	-2.65649724	-3.10767078	1	-3.61646271	0.26670283	2.08802938
1	4.35583735	-1.03839719	-3.83827066	8	-3.51036263	2.50840282	-1.68177068
1	3.12283731	-2.19779706	-4.34277105	6	-3.34076262	2.32500291	-3.10577083
6	1.86713743	-0.20729718	-3.10837078	1	-2.85966253	1.36620283	-3.30537081
1	1.17833734	0.28830281	-2.42187071	1	-2.74936271	3.13920283	-3.53197074
1	1.29153740	-0.62359715	-3.94447064	1	6.57613754	-0.33439717	0.05392923
1	2.52743745	0.57020283	-3.50827074	1	-4.34516239	2.33640289	-3.52577066

TS-24_PP

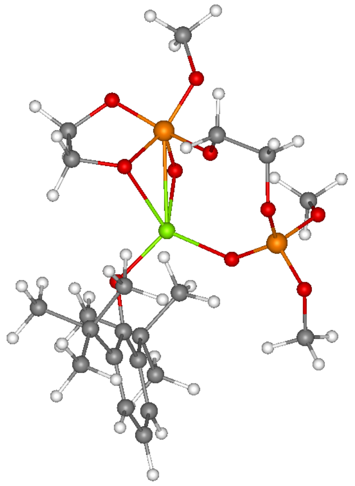

Zero-point vibrational energy	1569309.1 (Joules/Mol)
	375.07388 (Kcal/Mol)
Zero-point correction=	0.597718 (Hartree/Particle)
Thermal correction to Energy=	0.636766
Thermal correction to Enthalpy=	0.637710
Thermal correction to Gibbs Free Energy=	0.524904
Sum of electronic and zero-point Energies=	-2457.202690
Sum of electronic and thermal Energies=	-2457.163642
Sum of electronic and thermal Enthalpies=	-2457.162698

Sum of electronic and thermal Free Energies= -2457.275504

cartesian

12	-0.69835955	-0.22166945	0.20521393	1	3.10524035	-2.19056964	-2.88118625
8	-2.25265956	3.34293056	1.38901401	1	1.66334033	-1.88716948	-3.86748624
8	0.97394043	-1.03766942	-0.14218606	1	1.50864053	-2.18006945	-2.11758614
6	-2.47685957	2.42083049	2.48761392	1	-2.80295968	1.45003057	2.11401391
1	-1.56295943	2.31483054	3.07451391	8	-2.23395944	-1.00926936	1.27501392
6	2.19234037	-0.49366945	-0.04688606	15	-3.21295953	-1.07616937	0.08971395
6	2.86104035	-0.45216948	1.21611392	8	-3.58445954	-2.69436955	-0.12918606
6	4.07714081	0.23343053	1.30341399	8	-2.05855966	-1.24076939	-1.25578606
1	4.59104061	0.30513054	2.25591397	6	-2.47995949	-3.45106959	-0.59728611
6	4.66724062	0.82983053	0.19351393	6	-1.85265946	-2.58706951	-1.68678606
6	4.05984068	0.69293052	-1.05078602	1	-2.85575962	-4.40176916	-0.98098606
1	4.56074047	1.11843061	-1.91408598	1	-1.77975965	-3.63766956	0.22461393
6	2.84274030	0.02283055	-1.21168602	1	-0.78165954	-2.76376963	-1.79908597
6	2.29874039	-1.18836939	2.44871378	1	-2.35455942	-2.72276950	-2.65058613
6	2.26334047	-0.19376947	-2.62368608	8	-4.78305960	-0.74036950	0.38671395
6	2.18814039	-2.69546962	2.13821387	6	-5.75085926	-1.69966936	0.83411390
1	1.55914044	-2.86656952	1.26231396	1	-6.01995945	-2.38836956	0.03151394
1	1.75314045	-3.23166943	2.99121380	1	-5.37945938	-2.26636958	1.69041395
1	3.17824030	-3.12036943	1.94111395	1	-6.62275934	-1.11276937	1.13041401
6	0.91294044	-0.65226948	2.87511396	8	-0.48225954	1.74703062	0.41721395
1	0.10264045	-1.10756946	2.30101395	15	-1.26775956	3.00493050	0.18891394
1	0.86014050	0.43893054	2.78961396	8	-2.11575961	3.12113047	-1.13998604
1	0.70684046	-0.91006953	3.92081380	8	-3.03755951	0.60613054	-0.30018604
6	3.22134042	-1.04326940	3.67271376	6	-3.49725962	2.70243049	-1.26288605
1	3.30294037	-0.00436945	4.01211405	6	-3.61085963	1.20613062	-1.44578600
1	4.22964048	-1.42216945	3.47741389	1	-3.87425971	3.23293042	-2.13908625
1	2.81104040	-1.62716937	4.50411367	1	-4.04555941	3.02843046	-0.37558606
6	3.18444037	0.36993054	-3.72038603	1	-4.67555952	0.95213050	-1.53008604
1	4.18404055	-0.07546945	-3.68998623	1	-3.08855963	0.88033050	-2.35358620
1	3.28994036	1.45873058	-3.65688610	8	-0.32305953	4.25733089	-0.00768606
1	2.75634050	0.14193054	-4.70288610	6	0.92944044	4.33853054	0.71091390
6	0.89734048	0.49683052	-2.80488610	1	1.48804045	3.40653038	0.61351395
1	0.11614048	0.00313054	-2.22478604	1	0.74034041	4.56723070	1.76261401
1	0.58714044	0.45553052	-3.85648608	1	5.60934067	1.36433053	0.29101396
1	0.94434041	1.55023062	-2.51048613	1	-3.25675964	2.88123035	3.09311390
6	2.12214041	-1.70716941	-2.88658619	1	1.47884035	5.15773058	0.24991393

I-4_PP

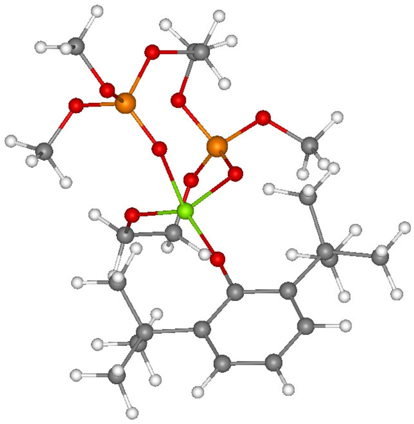

Zero-point vibrational energy	1566502.0 (Joules/Mol)
	374.40297 (Kcal/Mol)
Zero-point correction=	0.596649 (Hartree/Particle)
Thermal correction to Energy=	0.637223
Thermal correction to Enthalpy=	0.638167
Thermal correction to Gibbs Free Energy=	0.521953
Sum of electronic and zero-point Energies=	-2457.222104
Sum of electronic and thermal Energies=	-2457.181530
Sum of electronic and thermal Enthalpies=	-2457.180586
Sum of electronic and thermal Free Energies=	-2457.296800

cartesian

12	0.49715561	-0.04940699	-0.48172364	1	-1.86334443	-2.08550715	-3.29362369
8	2.33065557	-3.19190717	2.15207624	1	-1.48534441	-0.61670697	-2.37682366
8	-1.31954432	0.15189300	-0.13852362	1	3.90665579	-4.53280687	1.83787644
6	3.34785557	-4.07690668	2.65877628	8	1.56215572	1.69349301	-0.17762363
1	2.82125568	-4.84600687	3.22187638	15	2.91635561	1.90569305	-0.78972358
1	4.02645540	-3.53060699	3.31777620	8	2.98425579	1.93989301	-2.35932374
6	-2.61174440	0.10679300	0.15277638	8	1.48415565	-0.54320699	-2.03182364
6	-3.17904425	1.07909298	1.03837645	6	1.90285563	1.49599302	-3.24522376
6	-4.54674435	1.01259303	1.31867635	6	1.77715564	-0.01810699	-3.26842380
1	-4.99704409	1.73969305	1.98657644	1	2.17965555	1.90519309	-4.21962357
6	-5.36714411	0.03479301	0.76577640	1	0.97335565	1.96399307	-2.90572381
6	-4.80984449	-0.90830696	-0.09132362	1	1.00155568	-0.23490700	-4.02962351
1	-5.46354437	-1.66440701	-0.51352358	1	2.72375560	-0.42880699	-3.66572380
6	-3.45024443	-0.90490699	-0.41692364	8	1.45885563	-1.22220695	0.87467641
6	-2.31664443	2.18409300	1.67637634	15	2.69345570	-1.99880695	1.16987646
6	-2.88564444	-1.97700691	-1.36742365	8	3.83555579	-1.18040693	1.92127645
6	-1.69944429	3.07889295	0.58247638	8	4.04915571	0.87509304	-0.35482362
1	-1.08474445	2.50289297	-0.10892362	6	3.83035564	0.25799301	2.03117633

1	-1.07444429	3.85909295	1.03687644	6	4.66045570	0.91689301	0.95017642
1	-2.49014425	3.57369280	0.00707638	1	4.28945541	0.47209302	2.99967623
6	-1.21474433	1.56219304	2.55887628	1	2.80305576	0.63029301	2.03917623
1	-0.58704442	0.87069303	1.99617636	1	4.85945559	1.95489299	1.23227644
1	-1.66284442	1.00479305	3.38907623	1	5.61035585	0.39279300	0.83387637
1	-0.57514435	2.34619284	2.98477626	8	3.48845577	-2.61780715	-0.04692363
6	-3.13414431	3.11229300	2.59197617	6	2.77915573	-3.27150702	-1.13942361
1	-3.59164429	2.57299280	3.42827630	1	2.20415568	-2.51620698	-1.68382359
1	-3.92494440	3.64039302	2.04837632	1	2.13885570	-4.06760693	-0.75002360
1	-2.46934438	3.87209296	3.01917624	1	-6.42854452	0.00759301	1.00087643
6	-3.96874428	-2.95020700	-1.86692357	8	3.55725574	3.28839302	-0.31132364
1	-4.75004435	-2.44090700	-2.44122362	6	2.77075577	4.49509335	-0.39562362
1	-4.44514418	-3.49820709	-1.04722357	1	3.34325576	5.26469326	0.12067638
1	-3.50764441	-3.69170713	-2.52972364	1	2.63475561	4.77789307	-1.44232357
6	-1.83624434	-2.83900714	-0.63572359	1	1.80365562	4.35429335	0.09107638
1	-1.04534435	-2.23540711	-0.18822363	1	3.55915570	-3.70060706	-1.76732361
1	-1.37754440	-3.55750704	-1.32702363	6	-2.27954435	-1.32120693	-2.62522364
1	-2.30844426	-3.40410709	0.17557637	1	-3.05314422	-0.77450699	-3.17592382

TS-45_PP

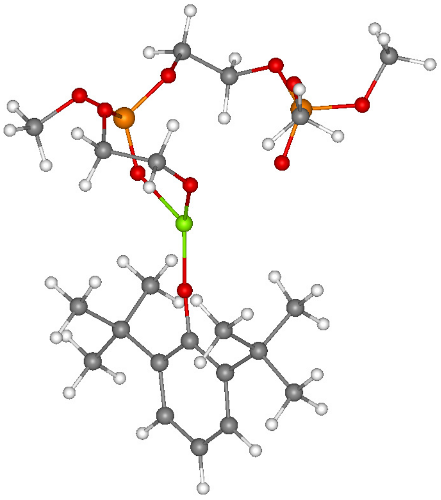

Zero-point vibrational energy	1563528.3 (Joules/Mol)
	373.69222 (Kcal/Mol)
Zero-point correction=	0.595516 (Hartree/Particle)
Thermal correction to Energy=	0.635976
Thermal correction to Enthalpy=	0.636920
Thermal correction to Gibbs Free Energy=	0.518929
Sum of electronic and zero-point Energies=	-2457.197842
Sum of electronic and thermal Energies=	-2457.157383
Sum of electronic and thermal Enthalpies=	-2457.156439

Sum of electronic and thermal Free Energies= -2457.274430

cartesian

12	0.15823469	0.15365970	-0.73387504	6	-2.25516534	-1.95184028	-2.38117504
8	3.91033459	-3.14894032	1.89312506	1	-3.14666533	-1.67634022	-2.95517492
8	-1.56066525	0.01215971	-0.10997502	1	-1.68906534	-2.68834043	-2.96387506
6	5.27503490	-3.58674049	2.00042486	1	-1.63856518	-1.05684030	-2.28687501
1	5.24343491	-4.55734015	2.49472499	1	5.72813463	-3.68854046	1.01092505
1	5.85233498	-2.88194036	2.60402513	8	1.16733468	1.81065977	-0.17477500
6	-2.82076550	-0.26074028	0.22072499	15	2.50983453	2.28455973	-0.65977502
6	-3.56826520	0.68765974	0.98602498	8	2.69293451	2.32705951	-2.22047496
6	-4.89256525	0.38255972	1.31432509	8	1.23543465	-0.29904029	-2.19867492
1	-5.48186541	1.08615971	1.89282501	6	1.72933471	1.84995973	-3.21497488
6	-5.49306536	-0.80884027	0.92222500	6	1.70313466	0.33405972	-3.32807493
6	-4.75656509	-1.72644031	0.18142499	1	2.06413460	2.31895971	-4.14307499
1	-5.24046516	-2.65094042	-0.11467502	1	0.74083471	2.24335957	-2.95337486
6	-3.42846537	-1.48914027	-0.18627501	1	1.08063471	0.12425971	-4.22007513
6	-2.94686532	2.01795959	1.45222509	1	2.72543454	0.00095971	-3.58277488
6	-2.66296530	-2.53834033	-1.01397491	8	2.08143473	-1.53884029	1.16042507
6	-2.51926541	2.86815953	0.23842499	15	3.53173470	-1.81384027	1.10422504
1	-1.78706539	2.34635973	-0.37877503	8	4.47453451	-0.69824028	1.77962506
1	-2.07886553	3.81635952	0.57332498	8	3.77593470	1.47835970	-0.16527501
1	-3.38636541	3.10305953	-0.38887504	6	3.95183468	0.59445965	2.11972499
6	-1.74346530	1.75085974	2.37882495	6	4.39443493	1.64825976	1.13102508
1	-0.97526526	1.15695977	1.88322508	1	4.37153482	0.84545970	3.09862494
1	-2.06586552	1.20605969	3.27282500	1	2.86273456	0.55295968	2.20452499
1	-1.29316533	2.69735956	2.70512509	1	4.17293453	2.64375973	1.52492499
6	-3.93816543	2.87665963	2.25782514	1	5.46683502	1.56925976	0.94582498
1	-4.27556515	2.37305951	3.16982508	8	4.20883465	-1.95244026	-0.33287501
1	-4.82006502	3.15575957	1.67132509	6	3.48443460	-2.62624049	-1.39397502
1	-3.44346547	3.80555964	2.56412506	1	2.63533449	-2.01284027	-1.70707500
6	-3.51846528	-3.78144050	-1.32157493	1	3.15333462	-3.61584044	-1.06657493
1	-4.40466499	-3.53994036	-1.91807497	1	-6.52476501	-1.02054024	1.19262505
1	-3.84416556	-4.29614019	-0.41167504	8	2.82123470	3.75255966	-0.12177501
1	-2.92046547	-4.49344015	-1.90177500	6	1.80583465	4.77515984	-0.20447502
6	-1.43276525	-3.05024028	-0.23687501	1	2.18753457	5.62366009	0.36162499
1	-0.77086532	-2.24684048	0.09012499	1	1.65563476	5.06655979	-1.24697495
1	-0.84986532	-3.74604034	-0.85227501	1	0.87133467	4.41905975	0.23272499
1	-1.74916518	-3.58264041	0.66642499	1	4.19973469	-2.73384047	-2.20947504

I-5_PP

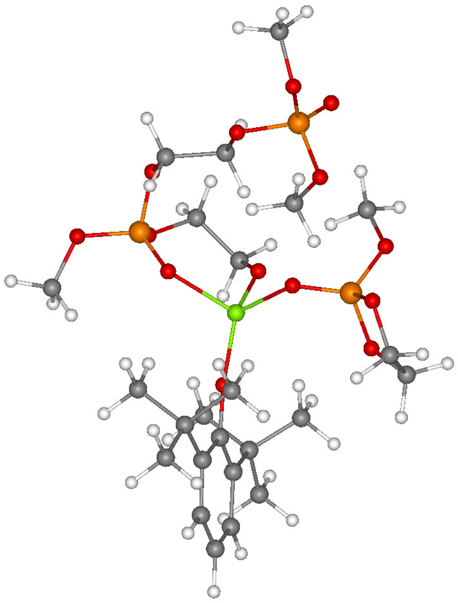

Zero-point vibrational energy	1870434.4 (Joules/Mol)
	447.04454 (Kcal/Mol)
Zero-point correction=	0.712411 (Hartree/Particle)
Thermal correction to Energy=	0.764194
Thermal correction to Enthalpy=	0.765139
Thermal correction to Gibbs Free Energy=	0.618509
Sum of electronic and zero-point Energies=	-3217.786351
Sum of electronic and thermal Energies=	-3217.734568
Sum of electronic and thermal Enthalpies=	-3217.733624
Sum of electronic and thermal Free Energies=	-3217.880253

cartesian

12	0.23214152	-0.61861616	0.58668512	6	-3.54245830	-2.53571630	-0.89501488
8	-4.43975830	1.95198393	-0.44291493	6	-3.91905832	-1.34491611	-0.03301492
8	1.88374150	-0.10371612	-0.14261490	1	-3.01985836	-2.21761632	-1.79991484
6	-4.06435871	2.26268387	-1.79871488	1	-4.42685843	-3.11001611	-1.17591488
1	-3.67575836	1.37468398	-2.30261493	1	-3.03305840	-0.75201613	0.21008509
1	-4.92495823	2.65948391	-2.34171510	1	-4.39445829	-1.67881608	0.89298511
6	3.08934164	0.32098389	-0.48951492	8	-6.68625832	1.09328389	-1.06241488
6	4.18484163	0.25448388	0.43378508	6	-7.88265848	0.29948390	-0.95531487
6	5.42814159	0.75468385	0.03578509	1	-8.24885845	0.30098388	0.07368509
1	6.26714182	0.72048384	0.72278512	1	-7.68875837	-0.72331613	-1.28841484
6	5.63984156	1.29678392	-1.22741485	1	6.61864138	1.67878389	-1.50761485
6	4.58484173	1.32368386	-2.13301492	8	-0.58805847	-2.04861617	-0.61641490
1	4.77184153	1.72838390	-3.12221503	15	-1.13415849	-3.39291620	-0.23101491
6	3.31274152	0.84418386	-1.80641484	8	-0.61905849	-4.05371618	1.09788513

6	4.02704144	-0.36971611	1.83288515
6	2.19304156	0.87258387	-2.86361504
6	3.02764153	0.43918389	2.68248510
1	2.05364156	0.53328383	2.20328498
1	2.87864161	-0.03441612	3.66038489
1	3.41024160	1.45188391	2.85218501
6	3.58044147	-1.84071612	1.70718515
1	2.65134168	-1.93661606	1.14328516
1	4.34604168	-2.42571616	1.18528509
1	3.43304157	-2.28451610	2.69938493
6	5.34834146	-0.38891611	2.62318492
1	6.12784147	-0.96681613	2.11528492
1	5.73434162	0.61848384	2.81308508
1	5.17564154	-0.85851616	3.59818506
6	2.67324162	1.42388391	-4.21761465
1	3.02184153	2.46008372	-4.14681482
1	3.47854161	0.81968385	-4.64851475
1	1.83854139	1.40968394	-4.92801476
6	1.67364156	-0.55511612	-3.12871504
1	1.29124153	-1.01651609	-2.21811509
1	0.86904144	-0.53831613	-3.87531495
1	2.48274159	-1.18261611	-3.51971507
6	1.03934145	1.78408396	-2.40141511
1	1.39744151	2.81648374	-2.30311513
1	0.22434148	1.78018391	-3.13681507
1	0.63774145	1.46308386	-1.44021487
8	-5.70735836	0.62798387	1.34078515
15	-5.44915819	0.77268386	-0.10381491
8	-2.71975851	-3.42551613	-0.10841491
8	-4.85075855	-0.55911613	-0.80291492
8	-0.38985848	-1.32831609	2.23558497
6	-1.00395846	-3.64931631	2.45698500
6	-0.17425850	-2.47881627	2.95978498
1	-2.06935835	-3.40431619	2.44498491
1	-0.84465849	-4.55181599	3.05238509
1	0.88414145	-2.79591632	2.97458506
1	-0.46675849	-2.35951614	4.02278519
8	-0.82315850	-4.48401642	-1.34041488
6	0.50904143	-4.59411621	-1.88991487
1	1.18784153	-5.00581598	-1.13971484
1	0.86194146	-3.61971617	-2.23191500
1	0.42674151	-5.28051615	-2.73141503
8	-1.04545844	0.98138386	0.55628508
15	-1.16165853	2.26738381	1.30458510
8	0.25034150	2.76828384	1.88808513
8	-1.52665854	3.57138371	0.44338506
6	0.44004151	4.17068386	1.61938512
6	-0.38095850	4.45028400	0.35938507
1	0.08244148	4.74488401	2.47908497
1	1.50694144	4.33848381	1.47298515
1	0.17454150	4.21068382	-0.55011487
1	-0.75505853	5.47348356	0.31478509
8	-2.23275852	2.26208377	2.44798493
6	-2.70465851	1.03978395	3.09898496
1	-2.05835843	0.19148389	2.85468507
1	-3.73075843	0.87748384	2.76868510
1	-2.67085838	1.24558389	4.16898537
1	-3.28215837	3.01628375	-1.72421491
1	-8.61545849	0.76738387	-1.61191487

S1.5. CP sequence, mononuclear mechanism

I-1_CP

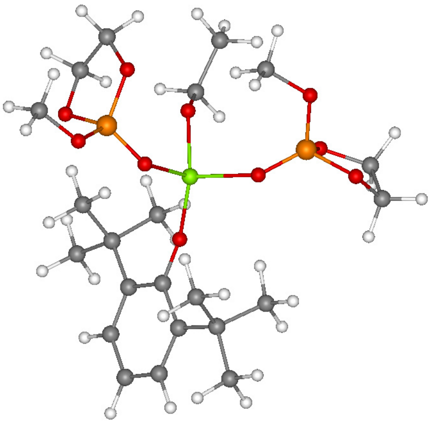

Zero-point vibrational energy	1638160.6 (Joules/Mol)
	391.52977 (Kcal/Mol)
Zero-point correction=	0.623942 (Hartree/Particle)
Thermal correction to Energy=	0.666703
Thermal correction to Enthalpy=	0.667647
Thermal correction to Gibbs Free Energy=	0.543208
Sum of electronic and zero-point Energies=	-2496.480479
Sum of electronic and thermal Energies=	-2496.437719
Sum of electronic and thermal Enthalpies=	-2496.436775
Sum of electronic and thermal Free Energies=	-2496.561213

cartesian

12	0.61740255	0.17141742	0.16157193	1	-1.30479741	1.32141745	3.73557210
8	1.97870255	0.01081741	-1.17392814	1	-0.86489737	0.18811741	2.44897199
8	-1.24289739	-0.05588260	0.07257193	6	3.28400254	0.73591739	-3.09402800
6	1.97560263	0.14851740	-2.56452799	8	1.49490261	-0.99968261	1.59577191
1	1.14980257	0.79141742	-2.91522789	15	2.53400254	-2.05778265	1.54237187
1	1.81180263	-0.82768261	-3.06222796	8	4.00910282	-1.57008255	1.12457192
6	-2.49749756	-0.47388262	0.17457193	8	2.36990237	-3.22068262	0.45137194
6	-3.02579761	-1.42018259	-0.76382810	6	4.43480253	-2.19108272	-0.11222808
6	-4.34529734	-1.85518253	-0.60912806	6	3.20340252	-2.90098262	-0.69282806
1	-4.75969744	-2.57748270	-1.30472803	1	5.24590254	-2.88268256	0.13177192
6	-5.16269732	-1.39028251	0.41517192	1	4.79240227	-1.40458250	-0.77622807
6	-4.65569735	-0.44968259	1.30527186	1	2.64110255	-2.23288274	-1.34672809
1	-5.30979776	-0.08498260	2.09047198	1	3.44850254	-3.84148264	-1.18622804
6	-3.34659743	0.03191741	1.21347189	8	2.61410236	-2.75338268	2.95797205
6	-2.18149757	-1.95198262	-1.93662798	6	3.54550242	-3.82268262	3.21027207

6	-2.85389757	1.09531748	2.21207190	1	3.34680247	-4.66408253	2.54297209
6	-0.96769738	-2.74448276	-1.41382813	1	4.57180262	-3.46718264	3.08917212
1	-0.33879742	-2.13978267	-0.76012808	1	3.37930250	-4.12248278	4.24377203
1	-0.35119745	-3.10078263	-2.24932790	8	1.04790258	2.03171730	0.89767194
1	-1.29819739	-3.61648273	-0.83902812	15	1.60740256	3.27651739	0.30687195
6	-1.72569740	-0.78298259	-2.83272791	8	1.17740262	4.65801716	1.00037193
1	-1.16219735	-0.04118259	-2.26632786	8	1.06320262	3.59051728	-1.17422807
1	-2.59599757	-0.28248259	-3.27202797	6	0.23190257	5.35401726	0.15877193
1	-1.09559739	-1.14838254	-3.65332794	6	0.60440254	4.95481730	-1.26962805
6	-2.96929741	-2.91578269	-2.84172797	1	0.34820256	6.42151737	0.34637195
1	-3.84809732	-2.43918276	-3.28882790	1	-0.77799737	5.03231716	0.42517191
1	-3.29869747	-3.81328273	-2.30702806	1	-0.24739745	4.97201729	-1.94962800
1	-2.32319760	-3.24528265	-3.66382790	1	1.41660261	5.56841755	-1.66952813
6	-3.94919729	1.52651751	3.20407200	8	3.18310237	3.33451724	0.31357193
1	-4.29589748	0.69521743	3.82727194	6	3.97180247	2.13351727	0.55597192
1	-4.81779766	1.96471751	2.70027208	1	3.93100238	1.88961744	1.61837196
1	-3.54259753	2.28911734	3.87847209	1	3.59490252	1.30451751	-0.04792808
6	-2.42649746	2.36861730	1.45257187	1	4.98750257	2.40101743	0.26737192
1	-1.65039742	2.14671731	0.71997190	1	3.27290249	0.80491740	-4.18892813
1	-2.04479742	3.12081742	2.15557194	1	3.44890237	1.74171746	-2.69252801
1	-3.28709745	2.79721737	0.92557192	1	4.13880253	0.11251741	-2.80412793
6	-1.69089746	0.54651743	3.06167197	1	-6.18459749	-1.74908257	0.51297194
1	-2.03389764	-0.29438260	3.67447209				

TS-12_CP

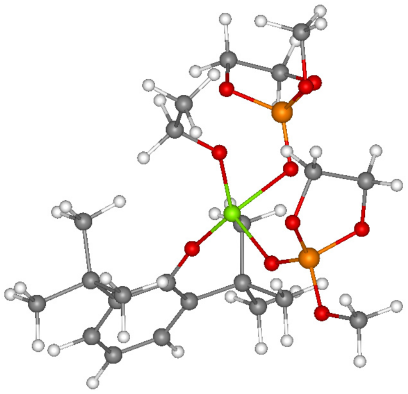

Zero-point vibrational energy	1639406.3 (Joules/Mol)
	391.82751 (Kcal/Mol)
Zero-point correction=	0.624417 (Hartree/Particle)
Thermal correction to Energy=	0.665903
Thermal correction to Enthalpy=	0.666847
Thermal correction to Gibbs Free Energy=	0.547305
Sum of electronic and zero-point Energies=	-2496.473564

Sum of electronic and thermal Energies= -2496.432077
Sum of electronic and thermal Enthalpies= -2496.431133
Sum of electronic and thermal Free Energies= -2496.550676

cartesian

12	0.41053650	-0.26081076	0.00927469	1	-0.99606347	0.42068923	3.92087483
8	1.70083654	0.20168923	-1.33782530	1	-0.92176342	-0.48121077	2.39757466
8	-1.44706345	-0.08801077	0.07387469	1	1.42073655	1.32478929	-3.04362535
6	1.65293658	0.29098922	-2.73432517	8	1.01333654	-2.01641083	0.84527469
1	0.84523654	-0.33471078	-3.14752531	15	2.32193661	-2.67321086	1.09177470
6	2.96323657	-0.13601077	-3.39512539	8	3.31803656	-1.86601079	2.06067467
6	-2.75746346	-0.23241076	0.21807469	8	3.31543660	-2.83241081	-0.15822530
6	-3.53666353	-0.83991075	-0.81902528	6	4.49573660	-1.42741072	1.34067476
6	-4.91456366	-0.97861075	-0.62862527	6	4.20833635	-1.68381071	-0.14432532
1	-5.52156353	-1.43981075	-1.40082526	1	5.34263659	-2.01081085	1.71227467
6	-5.54876328	-0.53931075	0.52877468	1	4.64263630	-0.36821079	1.55047476
6	-4.78966331	0.06338924	1.52637470	1	3.69033647	-0.84441078	-0.61892533
1	-5.30016327	0.40738922	2.42017484	1	5.09973669	-1.96281075	-0.70622528
6	-3.40726352	0.23388924	1.40677476	8	2.07273650	-4.11221075	1.68447471
6	-2.88956356	-1.32431078	-2.12922525	6	3.17173648	-4.96501112	2.06267476
6	-2.61666346	0.91208923	2.54017472	1	3.79483652	-5.18691111	1.19377470
6	-1.86916351	-2.44581079	-1.84572530	1	3.76103663	-4.49251080	2.85187483
1	-1.11956346	-2.14651084	-1.11232531	1	2.71853662	-5.88081121	2.43847466
1	-1.35426342	-2.74311066	-2.76802516	8	1.47173655	1.11868930	1.12057471
1	-2.37706351	-3.32891083	-1.44272530	15	2.18853641	2.15588927	0.32037470
6	-2.22156358	-0.13981077	-2.85652518	8	2.39523649	3.51798916	1.19717467
1	-1.47936344	0.35518926	-2.22922540	8	1.42913651	2.86898923	-0.91042531
1	-2.97606349	0.60538924	-3.13312531	6	1.36103654	4.45328903	0.85837471
1	-1.73136353	-0.47991079	-3.77752519	6	1.14133656	4.25988913	-0.64302528
6	-3.91676331	-1.91561079	-3.11122537	1	1.71523654	5.45428896	1.10787475
1	-4.67496347	-1.18321073	-3.40802526	1	0.46003652	4.22528887	1.43537474
1	-4.42716360	-2.79301071	-2.69992518	1	0.11253650	4.44938898	-0.95112526
1	-3.39906335	-2.23751068	-4.02232552	1	1.82723653	4.86548901	-1.24212527
6	-3.52086353	1.37238920	3.69767475	8	3.69383645	1.76108921	-0.00962530
1	-4.03886366	0.53598922	4.17897463	6	4.52263641	2.66868925	-0.75712526
1	-4.27116346	2.10148931	3.37317467	1	4.10643625	2.82318926	-1.75512528
1	-2.90396357	1.85578930	4.46417427	1	4.62253666	3.61878920	-0.22612530
6	-1.91006351	2.17748928	2.01087475	1	5.49823666	2.19028926	-0.83912528
1	-1.26306343	1.94288921	1.16517472	1	2.90783644	-0.02351077	-4.48502541
1	-1.30366349	2.63438916	2.80407476	1	3.80183649	0.47188926	-3.03512526
1	-2.65376353	2.91258931	1.68147469	1	3.18493652	-1.18611073	-3.17512536

6	-1.59826350	-0.07291076	3.14817476	1	-6.62266350	-0.66091073	0.64997470
1	-2.12096357	-0.91671073	3.61177468				

I-2_CP

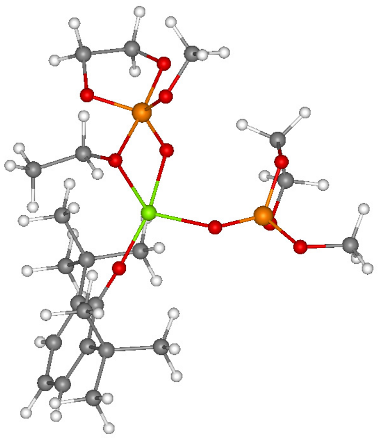

Zero-point vibrational energy	1643868.5 (Joules/Mol)
	392.89400 (Kcal/Mol)
Zero-point correction=	0.626116 (Hartree/Particle)
Thermal correction to Energy=	0.667507
Thermal correction to Enthalpy=	0.668451
Thermal correction to Gibbs Free Energy=	0.549132
Sum of electronic and zero-point Energies=	-2496.488615
Sum of electronic and thermal Energies=	-2496.447225
Sum of electronic and thermal Enthalpies=	-2496.446280
Sum of electronic and thermal Free Energies=	-2496.565600

cartesian

12	0.40965077	0.16324402	0.40965867	1	-0.26054922	-0.64395601	-3.51344132
8	1.38165081	-1.14505601	1.59255862	1	-0.62824929	0.37664402	-2.10824132
8	-1.41184926	0.10424402	0.11315867	1	2.22695065	-2.21815610	3.13935876
6	1.26205075	-1.77715588	2.87625861	8	1.00705075	2.09214401	0.63155866
6	0.16595076	-2.82685614	2.87855864	15	1.86575079	2.98484397	-0.18914133
1	1.05445075	-0.98825592	3.60785866	8	1.57025075	2.92744398	-1.76704133
6	-2.68734932	-0.06195597	-0.21684134	8	3.44295073	2.70274401	-0.24764132
6	-3.71524906	0.47494403	0.62185866	6	2.75785089	2.58334398	-2.51904130
6	-5.04834938	0.27384403	0.25275868	6	3.77115083	2.04324412	-1.50104129
1	-5.84524918	0.66924405	0.87385869	1	3.10675073	3.48874402	-3.02304125
6	-5.39964914	-0.42285597	-0.89884132	1	2.47655082	1.83074403	-3.25544143
6	-4.39484930	-0.93205601	-1.71394134	1	3.66755080	0.96604401	-1.35974133
1	-4.68664932	-1.46955609	-2.61034131	1	4.79785061	2.31524396	-1.74664128
6	-3.03974915	-0.77125597	-1.40884137	8	1.68635082	4.46114397	0.34075868

6	-3.38214922	1.26914406	1.89865863	6	2.35285068	5.56894398	-0.29484132
6	-1.96684933	-1.35285592	-2.34784126	1	3.43525076	5.42224407	-0.27984133
6	-2.57604933	2.53564405	1.54535866	1	1.99105084	5.68804407	-1.31894135
1	-1.64724922	2.29684401	1.02725863	1	2.09055066	6.44804430	0.29125869
1	-2.32734919	3.09714389	2.45485878	8	2.08045077	-0.42135596	-0.50814134
1	-3.16654921	3.19164395	0.89585871	15	2.79535079	-1.35045600	0.50555867
6	-2.59784913	0.39234403	2.89525867	8	4.18625069	-1.65595603	-0.44914132
1	-1.67254925	0.01654403	2.45655870	8	2.45885086	-2.96635604	0.55045867
1	-3.19764924	-0.47205597	3.20095873	6	4.00825071	-2.83215594	-1.20814133
1	-2.34644914	0.96554404	3.79645872	6	3.31445074	-3.78915596	-0.24654132
6	-4.64474916	1.74384403	2.64025879	1	4.98425102	-3.20155597	-1.53484130
1	-5.27504921	0.90924400	2.96625876	1	3.38335085	-2.63475609	-2.09034133
1	-5.25504923	2.41934395	2.03115869	1	2.69275069	-4.53425598	-0.74814135
1	-4.34694910	2.29644394	3.53865862	1	4.03095055	-4.29235601	0.41205868
6	-2.57644916	-2.05665612	-3.57384133	8	3.68005085	-0.71055597	1.71355867
1	-3.17654920	-1.37605596	-4.18724155	6	5.10645056	-0.60865599	1.74125862
1	-3.20284915	-2.90985608	-3.29314137	1	5.57895088	-1.59195590	1.69365871
1	-1.76834917	-2.43995595	-4.20754147	1	5.48005056	0.00764403	0.92145866
6	-1.12894917	-2.41915607	-1.61344135	1	5.34075069	-0.13295597	2.69595861
1	-0.66624916	-2.03695607	-0.70294130	1	0.06895077	-3.25945592	3.88075876
1	-0.33344921	-2.80135608	-2.26514125	1	-0.79834926	-2.39145613	2.60295868
1	-1.76394916	-3.26345611	-1.32354128	1	0.39985076	-3.62695599	2.17275858
6	-1.07044923	-0.22625598	-2.90234137	1	-6.44584942	-0.56425595	-1.15974128
1	-1.65874910	0.44984403	-3.53254128				

TS-23_CP

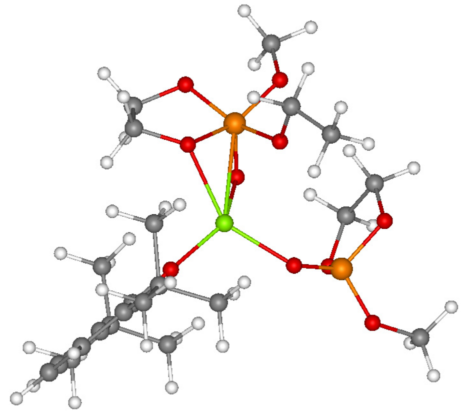

Zero-point vibrational energy	1643386.5 (Joules/Mol)
	392.77879 (Kcal/Mol)
Zero-point correction=	0.625933 (Hartree/Particle)
Thermal correction to Energy=	0.666641
Thermal correction to Enthalpy=	0.667585

Thermal correction to Gibbs Free Energy= 0.550969
Sum of electronic and zero-point Energies= -2496.472669
Sum of electronic and thermal Energies= -2496.431961
Sum of electronic and thermal Enthalpies= -2496.431016
Sum of electronic and thermal Free Energies= -2496.547633

cartesian

12	-0.46078813	-0.07986808	-0.08209602	1	0.91301191	1.99273193	3.36870384
8	-2.48428822	-1.05306804	-1.10749602	1	0.91201186	1.78903198	1.60400391
8	1.38161194	0.08873192	0.01360398	6	-2.78728819	-0.75856811	-3.46929598
6	-2.75938821	-1.75566804	-2.32859612	8	-1.19088817	1.73093200	-0.60479605
1	-3.72698808	-2.26016808	-2.22699594	15	-2.36728811	2.54853177	-0.20409602
1	-1.98288810	-2.51246810	-2.49289608	8	-2.74498820	2.51143193	1.34900391
6	2.69871187	0.22153193	0.09280398	8	-3.79758811	2.13223195	-0.79209602
6	3.50461197	0.11553192	-1.08599603	6	-3.96658802	1.76873195	1.58040392
6	4.89331198	0.21093193	-0.95479608	6	-4.52028799	1.35403192	0.19620398
1	5.52511168	0.12433192	-1.83249605	1	-4.64158821	2.43453193	2.12210393
6	5.50731182	0.41823190	0.27570397	1	-3.71168804	0.89563191	2.18000388
6	4.71571207	0.55553192	1.41080391	1	-4.35138798	0.29703194	-0.01009602
1	5.21011209	0.73443192	2.35990405	1	-5.57568789	1.60313201	0.07920398
6	3.32121181	0.47253191	1.35770392	8	-2.10258818	4.04253197	-0.63319600
6	2.87721181	-0.08456808	-2.47829604	6	-3.06578803	5.07783222	-0.35169601
6	2.49121189	0.66803193	2.64060402	1	-4.01688814	4.85243225	-0.83899605
6	1.93631196	1.09133196	-2.81299615	1	-3.19948816	5.18393230	0.72700393
1	1.16201186	1.23183191	-2.05769610	1	-2.64418817	5.99283218	-0.76419604
1	1.45181191	0.93043190	-3.78469610	8	-1.93638813	-0.66336811	1.22650397
1	2.50411177	2.02623200	-2.87339616	15	-2.33268809	-1.88506806	0.37540397
6	2.11921191	-1.42506802	-2.54819608	8	-2.15548801	-3.28226805	1.26180398
1	1.32821190	-1.49046803	-1.80049610	8	-0.69828808	-2.23676825	-0.30899602
1	2.80731177	-2.26076818	-2.37699604	6	-0.80588812	-3.57026815	1.60860395
1	1.66931200	-1.55966806	-3.54029608	6	0.00811189	-3.27786803	0.34980398
6	3.93321180	-0.12516807	-3.59779596	1	-0.75458813	-4.61816788	1.90980399
1	4.62861204	-0.96406811	-3.48509598	1	-0.50138813	-2.93066812	2.44340396
1	4.51591206	0.80043191	-3.65199614	1	1.02341187	-2.94686818	0.57900393
1	3.42891192	-0.24916808	-4.56309605	1	0.04901189	-4.14746809	-0.31559601
6	3.36911178	0.95323193	3.87230396	8	-3.96598816	-2.11186814	0.33580399
1	3.96691179	1.86293197	3.75290394	6	-4.69828796	-2.84896827	1.31940401
1	4.04631186	0.12363192	4.10240412	1	-4.53238821	-3.92236805	1.20970392
1	2.72491193	1.09993196	4.74690390	1	-4.42638826	-2.54566813	2.33400393
6	1.68521190	-0.60426807	2.96880388	1	-5.75148821	-2.62006807	1.13760400
1	0.99731183	-0.86216807	2.16320395	1	-3.01908803	-1.27436805	-4.40659618

1	1.09901190	-0.46506810	3.88550401	1	-3.54798818	0.00793192	-3.29829597
1	2.36111188	-1.45306802	3.12310386	1	-1.81888807	-0.26376808	-3.58309603
6	1.55111194	1.88073194	2.48340392	1	6.59051180	0.48293191	0.34750399
1	2.13561177	2.80023193	2.36880398				

I-3_CP

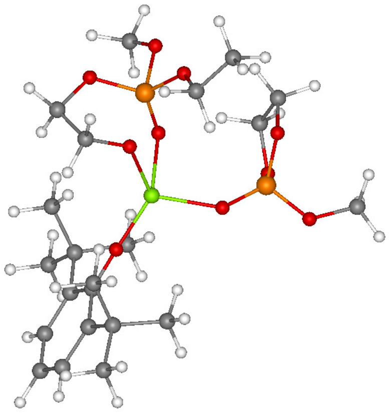

Zero-point vibrational energy	1643197.0 (Joules/Mol)
	392.73350 (Kcal/Mol)
Zero-point correction=	0.625861 (Hartree/Particle)
Thermal correction to Energy=	0.667501
Thermal correction to Enthalpy=	0.668446
Thermal correction to Gibbs Free Energy=	0.549508
Sum of electronic and zero-point Energies=	-2496.490683
Sum of electronic and thermal Energies=	-2496.449042
Sum of electronic and thermal Enthalpies=	-2496.448098
Sum of electronic and thermal Free Energies=	-2496.567035

cartesian

12	0.26832652	0.02891735	0.22843331	1	-0.89907342	-0.87418264	-3.62356663
8	3.69682670	-1.03028262	1.22113335	1	-0.97147352	0.19851735	-2.21186662
8	-1.57337356	0.05761734	0.09073333	1	2.99442649	0.64781737	2.22873354
6	3.61282659	-0.23198265	2.41413355	8	1.13502645	1.83421731	-0.07396667
6	5.02822638	0.15801734	2.79023337	15	2.30052662	2.34731722	-0.84406668
1	3.14902663	-0.81368262	3.21433353	8	2.44092655	1.90691733	-2.37596655
6	-2.88367343	0.09791735	-0.12576666	8	3.73302650	1.85031736	-0.32856667
6	-3.73677349	0.80691737	0.77853334	6	3.44752669	0.86461735	-2.49686646
6	-5.11367321	0.80421734	0.53603333	6	4.44272661	1.09821737	-1.34706664
1	-5.78077364	1.33021736	1.21073329	1	3.90732670	0.97901738	-3.47866654
6	-5.67097330	0.14531735	-0.55456668	1	2.94822669	-0.09948265	-2.40276647
6	-4.83347321	-0.52508265	-1.43906665	1	4.76852655	0.15991735	-0.89776665
1	-5.28387356	-1.02558267	-2.28966665	1	5.30332661	1.69971740	-1.65056670

6	-3.44737339	-0.56758261	-1.26056671	8	2.24772644	3.92411733	-0.80686671
6	-3.17327332	1.57501733	1.98953331	6	3.25982666	4.72041702	-1.45456672
6	-2.56567335	-1.31468260	-2.27936649	1	4.23022652	4.55481720	-0.98056668
6	-2.22547340	2.69541717	1.51593328	1	3.30352664	4.48571730	-2.52036667
1	-1.40347338	2.31101727	0.91163331	1	2.95402670	5.75601721	-1.31646669
1	-1.80107331	3.22901726	2.37593341	8	1.72392654	-1.00528264	-0.66266668
1	-2.77217340	3.42291737	0.90583330	15	2.52572656	-1.78088260	0.40713331
6	-2.43817329	0.61571735	2.94693351	8	2.02012658	-3.21408272	0.99613333
1	-1.62617350	0.09311734	2.44013333	8	1.16712654	-1.01538265	1.69013333
1	-3.12947345	-0.13818264	3.34023333	6	0.81632650	-3.30688286	1.76293337
1	-2.01907349	1.16701734	3.79813337	6	0.69402653	-2.01478267	2.55683351
6	-4.27647352	2.25521731	2.81883335	1	0.90812653	-4.19448280	2.39253354
1	-4.98867321	1.53441739	3.23453355	1	-0.02687347	-3.43258286	1.07593334
1	-4.83557320	2.99581718	2.23723340	1	-0.34417349	-1.82828259	2.84913349
1	-3.81747341	2.78351736	3.66223335	1	1.30932653	-2.05748272	3.46803355
6	-3.38597345	-1.94148266	-3.42106652	8	3.68002653	-2.48408270	-0.53786665
1	-3.93707323	-1.19048262	-3.99656653	6	3.25802660	-3.35738277	-1.58016670
1	-4.09937334	-2.68878269	-3.05796647	1	2.80382657	-4.26558256	-1.17096663
1	-2.70677352	-2.45028281	-4.11506701	1	2.54432654	-2.86478281	-2.25016665
6	-1.81787348	-2.47738266	-1.59666669	1	4.15442657	-3.62948275	-2.14206648
1	-1.20657349	-2.13058281	-0.76266670	1	5.01112652	0.75081736	3.71033335
1	-1.16347337	-2.99008274	-2.31206656	1	5.64592648	-0.72808266	2.95933342
1	-2.53227329	-3.20958281	-1.20436668	1	5.49062681	0.76041734	2.00393343
6	-1.57477355	-0.33848265	-2.94556665	1	-6.74617338	0.15901735	-0.71646667
1	-2.11707330	0.41211733	-3.53066659				

I-4_CP

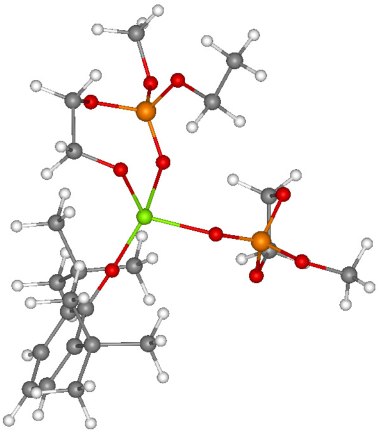

Zero-point vibrational energy 1640580.5 (Joules/Mol)

392.10815 (Kcal/Mol)

Zero-point correction= 0.624864 (Hartree/Particle)

Thermal correction to Energy= 0.667099

Thermal correction to Enthalpy= 0.668043
Thermal correction to Gibbs Free Energy= 0.546623
Sum of electronic and zero-point Energies= -2496.494176
Sum of electronic and thermal Energies= -2496.451941
Sum of electronic and thermal Enthalpies= -2496.450997
Sum of electronic and thermal Free Energies= -2496.572417

cartesian

12	0.39618522	-0.00831600	0.80612534	1	-0.33831477	-0.68631601	-3.48837471
8	3.54258537	-2.15861607	-1.44747472	1	-0.63811475	0.18698400	-1.96587467
8	-1.36531472	-0.18701601	0.21162534	1	5.41748524	-2.85931611	-0.85347468
6	4.53808498	-3.19411612	-1.40997469	8	0.95178527	1.96198404	0.72722536
1	4.81038523	-3.38791609	-2.44717479	15	1.35068536	2.95688391	-0.29767466
1	4.12698507	-4.10021591	-0.95947468	8	0.39468524	3.05168390	-1.58157468
6	-2.64871478	-0.36481601	-0.06987467	8	2.73628521	2.69568396	-1.07477462
6	-3.66251469	0.07508401	0.84232533	6	1.11698532	2.76018405	-2.80207467
6	-5.00301504	-0.12451599	0.49862534	6	2.43178535	2.10508394	-2.36687469
1	-5.78631496	0.20168400	1.17482531	1	1.27628517	3.70498395	-3.32907486
6	-5.38011503	-0.73411602	-0.69337469	1	0.49808526	2.09408402	-3.40257478
6	-4.39211512	-1.16281605	-1.57307470	1	2.33418536	1.02518404	-2.23507476
1	-4.70201492	-1.64001596	-2.49717474	1	3.26118541	2.33858395	-3.03437471
6	-3.03171468	-0.99821603	-1.29727471	8	1.46518517	4.37308407	0.38792533
6	-3.30861473	0.75348401	2.17912531	6	1.75338531	5.55788422	-0.38107467
6	-1.98071480	-1.50101602	-2.30407476	1	2.72298527	5.46388435	-0.87557465
6	-2.52911472	2.05998397	1.92722535	1	0.95998526	5.73498440	-1.11077464
1	-1.63121486	1.89248395	1.33272529	1	1.78208518	6.37598419	0.33662534
1	-2.23391461	2.52318406	2.87722516	8	1.87628531	-0.57601601	-0.52157468
1	-3.15691471	2.77548409	1.38372529	15	2.88228536	-1.61151600	-0.10287467
6	-2.49721479	-0.20691600	3.07182527	8	2.38328528	-2.94841599	0.58532536
1	-1.58361483	-0.55391598	2.58772516	8	1.26348519	-0.94961596	2.21922517
1	-3.09581470	-1.09091604	3.31862521	6	2.24408531	-3.11081600	2.02972531
1	-2.21691465	0.28428400	4.01192522	6	1.08688521	-2.27861595	2.55992532
6	-4.55741501	1.14458394	2.99002528	1	3.18908525	-2.80691600	2.48962522
1	-5.16711473	0.27548400	3.25942516	1	2.09378529	-4.18401575	2.17062521
1	-5.19241476	1.86008406	2.45662522	1	0.14578524	-2.70831609	2.17102528
1	-4.24181509	1.62268400	3.92452526	1	1.06778526	-2.43271589	3.65522528
6	-2.61491466	-2.13541603	-3.55437469	8	4.10738516	-1.08261597	0.76332533
1	-3.24151468	-1.42761600	-4.10787487	6	4.10708523	0.20038401	1.46172535
1	-3.22171474	-3.01371598	-3.31037474	1	3.97438526	0.98998398	0.71772534
1	-1.81931472	-2.46761608	-4.23167467	1	3.25978541	0.20028400	2.15102530
6	-1.10101485	-2.58901596	-1.65707469	6	5.43298483	0.32948402	2.17612529

1	-0.61651474	-2.22701597	-0.75047469	1	5.46268511	1.28628397	2.70632529
1	-0.32201475	-2.92431593	-2.35317469	1	5.56278515	-0.47031599	2.91002512
1	-1.71221471	-3.45731592	-1.38757467	1	6.26998520	0.29908401	1.47282529
6	-1.11541486	-0.32541600	-2.80157471	1	-6.43161488	-0.87481600	-0.93257469
1	-1.73981476	0.40008399	-3.33647490				

TS-45_CP

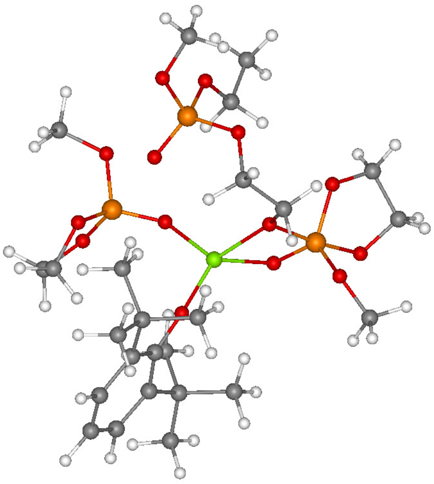

Zero-point vibrational energy	1949671.9 (Joules/Mol)
	465.98276 (Kcal/Mol)
Zero-point correction=	0.742591 (Hartree/Particle)
Thermal correction to Energy=	0.793301
Thermal correction to Enthalpy=	0.794245
Thermal correction to Gibbs Free Energy=	0.655007
Sum of electronic and zero-point Energies=	-3257.059837
Sum of electronic and thermal Energies=	-3257.009126
Sum of electronic and thermal Enthalpies=	-3257.008182
Sum of electronic and thermal Free Energies=	-3257.147421

cartesian

12	-0.10523887	0.60592556	0.45948553	6	-2.38783884	-0.08727442	-2.44541430
8	-3.87353897	-3.23817420	-1.69531453	1	-1.50293887	1.83552551	-2.67241430
8	1.60886109	0.37242556	-0.27001446	1	-3.14113879	1.85762560	-1.98391449
6	-5.22003889	-3.18237424	-2.19191432	1	-2.66273880	-0.08047442	-3.50501442
1	-5.36953878	-2.28767443	-2.80231452	1	-1.48033893	-0.68247443	-2.31961441
1	-5.93093872	-3.19097424	-1.36221457	8	-4.34663868	-1.86157441	0.30078554
6	2.89426112	0.16092558	-0.51581448	6	-4.16563892	-0.82287443	1.30668545
6	3.28926110	-0.53407443	-1.70731449	1	-3.25853896	-1.05067444	1.87138546
6	4.63826132	-0.86317444	-1.87411451	1	-4.03683901	0.13832557	0.80188555
1	4.95426130	-1.40937448	-2.75681448	6	-5.38843870	-0.82667446	2.19628549

6	5.60786152	-0.50327444	-0.94411451
6	5.23256111	0.25902557	0.15728553
1	6.00896120	0.58222556	0.84278554
6	3.90356088	0.62822556	0.39188552
6	2.27416110	-0.89087445	-2.81031442
6	3.57036114	1.56222558	1.57158554
6	1.53546107	0.38072556	-3.27601433
1	1.02306116	0.87532556	-2.45001435
1	0.79946113	0.12892558	-4.05061483
1	2.24286103	1.09712553	-3.70821428
6	1.27086115	-1.94707441	-2.31591439
1	0.72126114	-1.62297440	-1.43271446
1	1.78846121	-2.87887430	-2.06081438
1	0.54206109	-2.18627429	-3.10071445
6	2.95436120	-1.47947443	-4.05981445
1	3.44966102	-2.43497443	-3.85531449
1	3.69146109	-0.79427445	-4.49171448
1	2.19386101	-1.67007446	-4.82571459
6	4.83186150	2.05332565	2.30558562
1	5.51156139	2.59672570	1.64078546
1	5.39026117	1.23642552	2.77618551
1	4.53486156	2.74232578	3.10428572
6	2.70966101	0.84742558	2.62968564
1	1.79806113	0.42092556	2.21268559
1	2.41666102	1.54032552	3.42668557
1	3.27586102	0.02952558	3.09108567
6	2.85676122	2.82672572	1.04988551
1	3.51626110	3.37452579	0.36748552
1	2.59816122	3.49112558	1.88238549
1	1.93866110	2.59082580	0.51228553
8	-1.85423887	-2.27447438	-0.47051448
15	-3.25963879	-2.02737427	-0.85421449
8	-1.64153886	1.36812556	-0.66011447
8	-3.48503900	-0.70207441	-1.73141456
6	-2.18143892	1.33172560	-1.97311461

1	6.64876127	-0.77927446	-1.09591448
8	-0.76333886	2.24552560	1.33788550
15	-1.78053892	2.88952565	0.39088553
8	-2.06553888	4.26472569	1.34818542
8	-3.40323901	2.56412578	0.33818552
6	-3.37853885	4.75012589	1.18758547
6	-4.22463894	3.49252558	1.05098546
1	-3.46813893	5.38092566	0.28948551
1	-3.64923882	5.34512568	2.06388545
1	-4.46603870	3.06542563	2.03018546
1	-5.14243889	3.64172578	0.47708553
8	-1.24203885	3.70572567	-0.91811448
6	-0.60113883	4.98252583	-0.79711443
1	-1.32543886	5.77552557	-0.59441447
1	0.15396112	4.97362566	-0.00881447
1	-0.12213886	5.16152573	-1.76161456
8	-0.42933887	-0.88297445	1.76598549
15	-0.07483886	-2.30377436	2.02568555
8	1.04176116	-2.46877432	3.17738557
8	0.66666114	-3.14627433	0.88738555
6	2.21396112	-3.15127420	2.68018556
6	2.09196115	-3.14687443	1.15248549
1	2.21456122	-4.16377449	3.09418559
1	3.09436107	-2.60897422	3.02528548
1	2.53526115	-2.25737429	0.69858551
1	2.50616121	-4.04317427	0.69168556
8	-1.37093890	-3.07987428	2.47548556
6	-1.36163890	-4.50837421	2.65898561
1	-1.10353887	-5.00257444	1.72108543
1	-0.66403890	-4.78597450	3.45378566
1	-2.37553883	-4.77537441	2.95278549
1	-5.27103853	-0.07037442	2.97818565
1	-6.29173851	-0.59547442	1.62488544
1	-5.51973867	-1.79967439	2.67798567
1	-5.35413885	-4.07317448	-2.80591440

I-5_CP

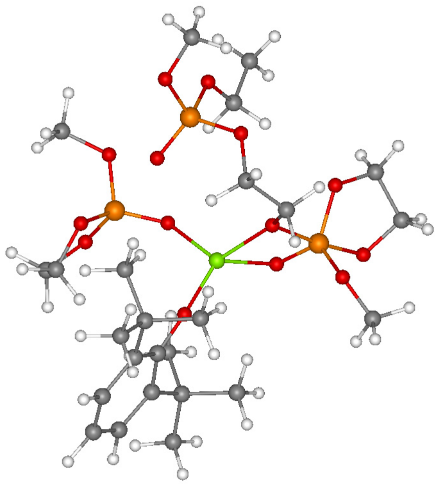

Zero-point vibrational energy	1944471.0 (Joules/Mol)
	464.73972 (Kcal/Mol)
Zero-point correction=	0.740610 (Hartree/Particle)
Thermal correction to Energy=	0.793970
Thermal correction to Enthalpy=	0.794915
Thermal correction to Gibbs Free Energy=	0.643328
Sum of electronic and zero-point Energies=	-3257.061075
Sum of electronic and thermal Energies=	-3257.007714
Sum of electronic and thermal Enthalpies=	-3257.006770
Sum of electronic and thermal Free Energies=	-3257.158357

cartesian

12	-0.78191978	0.80300331	0.25762117	6	3.13168025	0.86020327	3.39682126
8	0.83498025	1.41350329	-0.52077883	6	2.86308026	-0.56529677	2.91732121
8	-1.97581971	-0.58359671	-0.17047884	1	3.42058015	0.90120327	4.45042086
6	1.98348033	0.91010332	-1.10357881	1	3.86988020	1.35500324	2.76872134
1	1.86898029	0.72880328	-2.18597865	1	2.99818015	-0.65699673	1.83972120
1	2.30588007	-0.05429673	-0.66697884	1	3.47338009	-1.30459678	3.43492126
6	-2.67501974	-1.69249678	-0.35637885	8	0.04948026	0.65800327	4.76662111
6	-2.06531978	-2.85199666	-0.93887889	6	-1.32901967	0.31890327	5.04992104
6	-2.83681989	-4.00569677	-1.10437882	1	-1.98221982	0.68850327	4.25912094
1	-2.39181972	-4.89329672	-1.54187882	1	-1.42581975	-0.76419675	5.14692116
6	-4.17431974	-4.06279659	-0.72737885	1	-1.56361973	0.80240327	5.99712086
6	-4.76741982	-2.93019676	-0.17997883	8	-1.77261972	2.58810329	0.15462117
1	-5.81441975	-2.98629665	0.09952117	15	-1.81491971	3.70770335	-0.82487887
6	-4.06011963	-1.74009681	0.01262116	8	-3.18701982	4.53910303	-0.89547884
6	-0.59221977	-2.84669685	-1.38607883	8	-1.75891972	3.23600316	-2.35997868
6	-4.78031969	-0.50989676	0.59402114	6	-3.89301991	4.21950340	-2.11417866
6	0.33268026	-2.62909675	-0.17207883	6	-2.80141973	3.87070322	-3.12727880

1	0.08858025	-1.71499681	0.37112117	1	-4.47271967	5.09790325	-2.39887881
1	1.38138032	-2.57369685	-0.48997885	1	-4.55891943	3.37460327	-1.92227876
1	0.23568025	-3.46039677	0.53512114	1	-3.13741994	3.15920329	-3.88177896
6	-0.35921979	-1.76519680	-2.46047878	1	-2.38731980	4.76090336	-3.60927868
1	-0.62621975	-0.76899672	-2.10657883	8	-0.72921973	4.82210302	-0.57177883
1	-0.96761972	-1.97889686	-3.34657884	6	0.40698025	4.58150339	0.30762118
1	0.69298023	-1.75059676	-2.76957870	1	0.08268026	4.70280313	1.34242117
6	-0.16281976	-4.18299675	-2.01817870	1	0.81228024	3.58060336	0.13912116
1	-0.74441975	-4.42299652	-2.91477871	1	1.13378024	5.35040331	0.04802117
1	-0.24641974	-5.02189684	-1.31877887	8	4.39028025	1.47060323	-1.43347883
1	0.88838023	-4.11509657	-2.32167864	1	2.89718008	2.84540319	-1.43727887
6	-6.26611948	-0.77999675	0.89072114	1	3.22478008	2.14110327	0.16622117
1	-6.40481949	-1.56969678	1.63712120	1	-4.74891949	-4.97559690	-0.86547887
1	-6.82571936	-1.05659676	-0.00927883	15	5.32568026	0.49280328	-0.60427886
1	-6.72401953	0.13130328	1.29262114	8	5.15968037	-0.90089679	-1.35967886
6	-4.73731947	0.65070331	-0.42107886	8	6.80798054	0.91180325	-1.05567884
1	-3.70921993	0.90680325	-0.67797887	8	5.10408020	0.48060328	0.86602116
1	-5.22951937	1.53960323	-0.00387883	6	7.39258051	2.09380341	-0.48817885
1	-5.26451969	0.36560327	-1.33907890	6	5.92628050	-2.04749680	-0.91587889
6	-4.13671970	-0.08379671	1.92862117	6	5.49418020	-3.24299669	-1.73557889
1	-4.24311972	-0.88479674	2.66942120	1	6.98948050	-1.83019686	-1.05937886
1	-4.62931967	0.81360328	2.32482123	1	5.74068022	-2.20559669	0.15122117
1	-3.07411981	0.12820329	1.81142116	1	6.85728025	2.98380327	-0.83137888
6	3.11018014	1.91980326	-0.89847887	1	7.37968063	2.04400325	0.60362118
8	-0.40501976	0.71120328	2.26802135	1	8.42168045	2.12820339	-0.84587884
15	0.64738023	0.53580326	3.30532122	1	6.06318045	-4.12519693	-1.42677879
8	1.86738026	1.56330323	3.25702119	1	4.43068027	-3.44959664	-1.59247887
8	1.47488034	-0.83399677	3.24612117	1	5.67478037	-3.06909680	-2.79937863

S1.6. PC sequence, mononuclear mechanism

I-1_PC

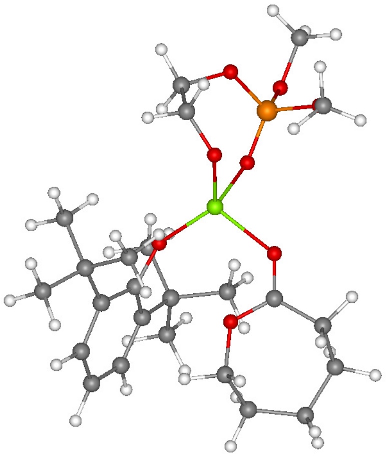

Zero-point vibrational energy	1675286.6 (Joules/Mol)
	400.40310 (Kcal/Mol)
Zero-point correction=	0.638083 (Hartree/Particle)
Thermal correction to Energy=	0.678025
Thermal correction to Enthalpy=	0.678969
Thermal correction to Gibbs Free Energy=	0.563290
Sum of electronic and zero-point Energies=	-2081.514540
Sum of electronic and thermal Energies=	-2081.474598
Sum of electronic and thermal Enthalpies=	-2081.473654
Sum of electronic and thermal Free Energies=	-2081.589333

cartesian

12	1.31035209	-0.13747860	0.45031193	1	0.20125212	1.82692134	-3.20378828
8	5.10425234	0.56482142	-1.80868804	1	0.24685213	1.17592132	-1.56848812
8	-0.08244789	-1.05097866	-0.44058806	1	6.93575239	1.32642138	-1.16308808
6	6.54165220	0.54452139	-1.81648803	8	0.70545208	1.71762133	1.17021191
1	6.83845234	0.73742139	-2.84688830	6	-0.42414787	2.13762140	1.44321191
1	6.91135216	-0.43477857	-1.50208807	8	-1.43434787	1.30272138	1.31841195
6	-1.37864792	-1.17727864	-0.67538810	6	-2.81774783	1.66482139	1.56751192
6	-2.20344782	-1.96447861	0.19571191	6	-3.35934782	2.70992136	0.60861194
6	-3.58804798	-1.94957864	-0.00608808	1	-2.91794777	1.96812141	2.61641192
1	-4.23614788	-2.51487851	0.65601194	1	-3.33244777	0.71472144	1.42511189
6	-4.17794800	-1.24257863	-1.05088806	1	-3.07964778	2.41602159	-0.40878808
6	-3.35814786	-0.58647859	-1.96518803	1	-4.45214796	2.63682151	0.65471190
1	-3.82764769	-0.10137860	-2.81528831	6	-0.63514793	3.55922151	1.89011192
6	-1.96674788	-0.55547857	-1.82728803	6	-1.43794787	4.40502167	0.88481194
6	-1.60224783	-2.85027862	1.30661190	1	-1.04054797	4.24382162	-0.12358808

6	-1.10204792	0.09852140	-2.92398810	6	-2.94444776	4.14912128	0.91291195
6	-0.98424792	-2.01227856	2.44281173	1	-1.25304794	5.45782137	1.12271190
1	-0.09634790	-1.46337867	2.12751174	8	2.83435225	0.37312141	-0.79398805
1	-0.67274785	-2.66417861	3.26851177	15	4.29295206	0.30532143	-0.46878806
1	-1.71294796	-1.29687870	2.84021187	8	4.82425213	-1.06827867	0.12361192
6	-0.53614783	-3.79107857	0.70811194	8	2.46865225	-0.83777857	1.79341197
1	0.25585210	-3.22737861	0.21381192	6	3.95875216	-2.19907856	0.46031192
1	-0.98974788	-4.46207857	-0.02968808	6	3.25525212	-1.96957862	1.79301190
1	-0.08974788	-4.40857840	1.49751198	1	4.63275242	-3.05897856	0.49081191
6	-2.66484785	-3.75257850	1.96221197	1	3.24005222	-2.33827853	-0.35408807
1	-3.17604780	-4.38757849	1.23091197	1	2.68335223	-2.89707851	1.99161196
1	-3.42194772	-3.18057847	2.51041174	1	4.03275204	-1.91687870	2.57831192
1	-2.17564774	-4.41387844	2.68611193	8	4.87185240	1.43252134	0.48921192
6	-1.93654788	0.51522142	-4.14878798	1	-5.25764799	-1.24947870	-1.18208802
1	-2.66814780	1.29652131	-3.91348815	1	-3.42824769	4.81812143	0.19261192
1	-2.46954775	-0.33277857	-4.59158802	1	-3.33364773	4.43252134	1.90051198
1	-1.26904786	0.92112142	-4.91718817	1	-1.14044785	3.55922151	2.86411190
6	-0.04574788	-0.90547860	-3.43148828	1	0.36145213	3.97842145	2.03331184
1	0.60545212	-1.24167860	-2.62468815	6	4.79045200	1.34972131	1.93491197
1	0.57355213	-0.44267857	-4.20958805	1	3.88965225	0.81352139	2.24501181
1	-0.53624785	-1.78327870	-3.86658812	1	4.77635241	2.38022137	2.29031181
6	-0.41104791	1.37652135	-2.41318822	1	5.68275213	0.83842140	2.30301189
1	-1.15664792	2.11892152	-2.10378814				

TS-12_PC

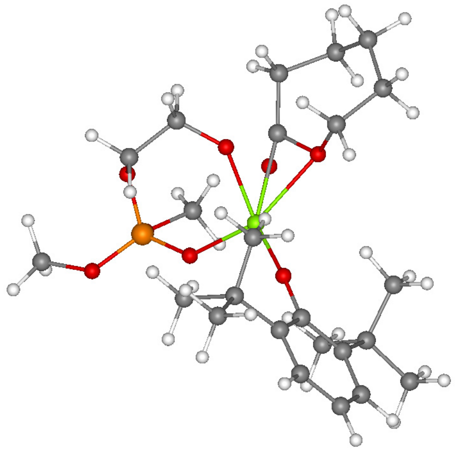

Zero-point vibrational energy	1676085.2 (Joules/Mol)
	400.59398 (Kcal/Mol)
Zero-point correction=	0.638387 (Hartree/Particle)
Thermal correction to Energy=	0.677014
Thermal correction to Enthalpy=	0.677958
Thermal correction to Gibbs Free Energy=	0.566048

Sum of electronic and zero-point Energies= -2081.492462
Sum of electronic and thermal Energies= -2081.453835
Sum of electronic and thermal Enthalpies= -2081.452891
Sum of electronic and thermal Free Energies= -2081.564802

cartesian

12	0.57755464	-0.08190262	-0.27801067	1	-1.59994531	1.98449743	-3.26191068
8	2.50175476	-4.09530258	-0.28111067	1	-1.32684529	1.60719740	-1.55051064
8	-1.25474524	-0.28190264	-0.10251068	1	4.02775478	-5.10330296	-1.28001070
6	3.64075470	-4.97860289	-0.26701069	8	2.17805457	0.95949745	-1.12331069
1	3.27445459	-5.93050289	0.11468932	6	2.12025476	1.70069742	-0.07751068
1	4.42205477	-4.58990288	0.39098933	8	0.81875461	2.22139716	0.03658932
6	-2.55504537	-0.53690267	-0.10161068	6	0.49185467	3.12589717	1.10698938
6	-3.18054533	-1.05020261	1.07948935	6	0.73275465	4.56669712	0.68308938
6	-4.55804539	-1.28680265	1.05728936	1	1.04665470	2.84579730	2.00868917
1	-5.05384541	-1.67320263	1.94188941	1	-0.56904536	2.95649719	1.30168939
6	-5.33034515	-1.04310262	-0.07391068	1	0.21805465	4.72199726	-0.27211070
6	-4.71354532	-0.55640262	-1.22181070	1	0.24155465	5.22229719	1.41298938
1	-5.32954550	-0.37730265	-2.09701085	6	3.26295471	2.64349723	0.26228932
6	-3.34064531	-0.29710263	-1.27411067	6	3.06725478	4.05399752	-0.31811067
6	-2.36714530	-1.34930265	2.35278916	1	2.65355468	3.98479748	-1.33051062
6	-2.70534539	0.23069736	-2.57411075	6	2.20435452	4.96749735	0.55968928
6	-1.69914532	-0.06530263	2.88518929	1	4.05675459	4.51389742	-0.41931069
1	-1.02834535	0.37779739	2.14868927	8	1.30345476	-1.92600262	-0.68101072
1	-1.12234533	-0.28140262	3.79388928	15	2.65205479	-2.56170273	-0.64241064
1	-2.46054530	0.68029737	3.14118934	8	3.64385462	-1.89550257	0.41308931
6	-1.30774534	-2.43280268	2.06278920	8	1.93635476	0.56909740	1.21878934
1	-0.66124535	-2.15890265	1.22788930	6	3.16825461	-1.49430263	1.72918940
1	-1.79274535	-3.37970281	1.80138934	6	3.06815481	0.01509738	1.84258938
1	-0.68594533	-2.60890269	2.95098925	1	3.91585469	-1.86940265	2.43338919
6	-3.24324536	-1.88980258	3.49668932	1	2.20775461	-1.96840262	1.95198929
1	-3.73804522	-2.83030272	3.23238921	1	3.04335475	0.25189736	2.91808915
1	-4.01024532	-1.17160261	3.80568933	1	3.99555469	0.44159737	1.43908930
1	-2.61204529	-2.08850265	4.37068939	8	3.49285460	-2.57520270	-1.97701061
6	-3.73604536	0.40349737	-3.70391083	1	-6.40074539	-1.23460269	-0.06221068
1	-4.51204538	1.13269734	-3.44811082	1	2.25455475	5.98859739	0.16338933
1	-4.22354507	-0.54060262	-3.96931076	1	2.65305471	5.01059723	1.56198931
1	-3.22724533	0.77129745	-4.60241032	1	3.39915466	2.71569729	1.34578931
6	-1.65264535	-0.76760268	-3.09981084	1	4.16495466	2.18829727	-0.15421069
1	-0.89614540	-1.00400257	-2.34991074	6	3.63635468	-1.39220262	-2.81171083
1	-1.15054536	-0.36520264	-3.98861074	1	2.84255481	-1.40610266	-3.55961084

1	-2.13024521	-1.71280265	-3.38051081	1	4.61065483	-1.48320258	-3.29031086
6	-2.07574534	1.61949742	-2.34281087	1	3.57415462	-0.47800264	-2.22021079
1	-2.84884524	2.34099722	-2.05541062				

I-2_PC

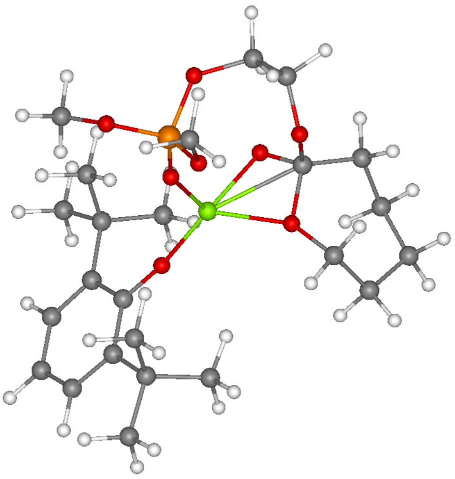

Zero-point vibrational energy	1678490.5 (Joules/Mol)
	401.16885 (Kcal/Mol)
Zero-point correction=	0.639303 (Hartree/Particle)
Thermal correction to Energy=	0.678320
Thermal correction to Enthalpy=	0.679264
Thermal correction to Gibbs Free Energy=	0.565282
Sum of electronic and zero-point Energies=	-2081.501711
Sum of electronic and thermal Energies=	-2081.462694
Sum of electronic and thermal Enthalpies=	-2081.461749
Sum of electronic and thermal Free Energies=	-2081.575732

cartesian

12	-0.42749742	0.09922667	0.37556538	1	1.72230256	0.69012666	3.91556525
8	-3.29609728	-2.16277337	-1.72723460	1	1.46440256	1.06872678	2.20546532
8	1.39050257	-0.03817332	0.09696537	1	-3.76929736	-4.00827312	-2.56813478
6	-4.26499748	-3.08267331	-2.27173471	8	-1.60419738	0.90922672	1.71946537
1	-4.68979740	-2.58597350	-3.14283466	6	-2.42499733	1.51502669	0.88166535
1	-5.05329752	-3.29797339	-1.54573464	8	-1.66289747	1.61202669	-0.38103464
6	2.69020271	-0.28937334	0.00016536	6	-2.12739730	2.42792654	-1.46593463
6	3.32950258	-0.25037333	-1.27913463	6	-1.52459741	3.82312655	-1.38873458
6	4.69920254	-0.52097332	-1.34793460	1	-3.22229743	2.44062662	-1.45963454
1	5.20760250	-0.49797332	-2.30643463	1	-1.80779743	1.92302668	-2.38163471
6	5.44920254	-0.82237333	-0.21583465	1	-0.43629742	3.71092653	-1.32333457
6	4.82040262	-0.85497332	1.02446544	1	-1.72689748	4.33532667	-2.33863473
1	5.42270231	-1.08807325	1.89646542	6	-2.95279741	2.87922668	1.34116542

6	3.45410275	-0.59627330	1.17026544	6	-1.97419739	4.04442692	1.15966535
6	2.54200268	0.08192669	-2.55993462	1	-0.95639741	3.71132660	1.39316535
6	2.80410266	-0.64347333	2.56526518	6	-2.03289747	4.68722677	-0.23203464
6	1.92320263	1.49112678	-2.45733476	1	-2.21849728	4.81862688	1.89626539
1	1.27900255	1.58502674	-1.58213460	8	-1.37069750	-1.58727324	-0.17203465
1	1.33410251	1.71772671	-3.35543466	15	-2.58259726	-2.43467331	-0.34083462
1	2.71120262	2.24752665	-2.37293482	8	-3.67189741	-2.35527349	0.80966532
6	1.44760251	-0.97627336	-2.80733466	8	-3.55359721	0.67342669	0.47216538
1	0.76430261	-1.06327331	-1.96193457	6	-4.70159769	-1.34217334	0.98076534
1	1.90140259	-1.96047330	-2.96953464	6	-4.18479729	-0.02877332	1.51996541
1	0.86320257	-0.72457331	-3.70183468	1	-5.39999771	-1.79577327	1.68686545
6	3.43520260	0.08882667	-3.81323481	1	-5.20719767	-1.18737328	0.02316536
1	3.89300275	-0.88797337	-4.00313425	1	-5.05269766	0.53142667	1.90506542
1	4.23270226	0.83652669	-3.74863482	1	-3.48669744	-0.20557332	2.34526539
1	2.82500267	0.33752668	-4.68913460	8	-2.24069738	-3.98467350	-0.37473464
6	3.81640267	-0.98137331	3.67436528	1	6.51390266	-1.02747333	-0.29903463
1	4.61110258	-0.23177332	3.75096536	1	-1.45169747	5.61712694	-0.22003464
1	4.28100252	-1.96257329	3.52986526	1	-3.07189727	4.98432684	-0.43293464
1	3.29800272	-1.00507331	4.63976574	1	-3.89669728	3.10302663	0.83106536
6	1.72790253	-1.74817324	2.61736536	1	-3.18619728	2.74692655	2.40216517
1	0.98830259	-1.63997328	1.82136536	6	-1.23199737	-4.54067326	0.49976537
1	1.20380259	-1.73527324	3.58086538	1	-0.30769742	-3.96677351	0.42106536
1	2.19200277	-2.73357344	2.49626517	1	-1.07159746	-5.56107330	0.15536535
6	2.20410275	0.72982669	2.93116522	1	-1.59449744	-4.54737329	1.52986538
1	2.99520278	1.48652673	2.97236538				

TS-24_PC

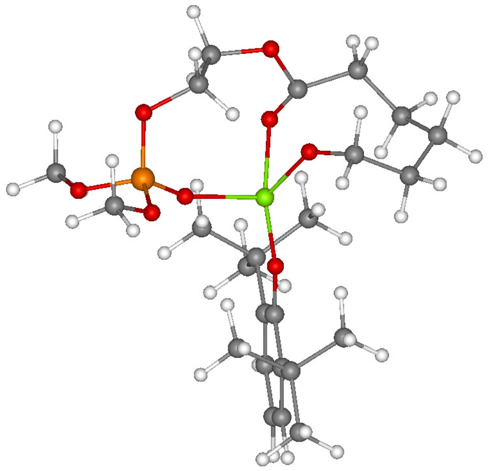

Zero-point vibrational energy	1675248.8 (Joules/Mol)
	400.39407 (Kcal/Mol)
Zero-point correction=	0.638069 (Hartree/Particle)

Thermal correction to Energy=	0.677002
Thermal correction to Enthalpy=	0.677946
Thermal correction to Gibbs Free Energy=	0.564773
Sum of electronic and zero-point Energies=	-2081.499104
Sum of electronic and thermal Energies=	-2081.460171
Sum of electronic and thermal Enthalpies=	-2081.459227
Sum of electronic and thermal Free Energies=	-2081.572400

cartesian

12	0.61558270	0.32051325	-0.11976940	1	-1.62871718	2.48651338	-2.94996929
8	2.89808273	-2.95788670	1.01483059	1	-1.42471719	1.94921327	-1.27486944
8	-1.19951725	-0.05128676	-0.02806940	1	3.76618290	-4.83858681	0.77983063
6	3.92308283	-3.91898680	1.34623063	8	1.50598264	1.61641335	-1.40606940
1	3.81438279	-4.11128664	2.41223073	6	2.33538270	2.27121329	-0.71386939
1	4.91398239	-3.50798678	1.13973057	8	1.87588263	1.33391333	0.98663056
6	-2.46261716	-0.44718677	-0.14106940	6	2.06528282	1.87341332	2.26953053
6	-3.09381723	-1.14538670	0.93803060	6	0.96608275	2.85101342	2.67533064
6	-4.43031740	-1.52958679	0.79513061	1	3.04838276	2.37311339	2.31533051
1	-4.92991734	-2.05448675	1.60253060	1	2.10248280	1.05521321	3.00623059
6	-5.15711737	-1.26118684	-0.35966939	1	0.00128272	2.34081340	2.56643057
6	-4.53471756	-0.59348679	-1.40856934	1	1.07608271	3.07411337	3.74553061
1	-5.11491728	-0.39508677	-2.30376935	6	2.13478279	3.71971321	-0.34016940
6	-3.20221710	-0.17638676	-1.33716941	6	0.83108270	4.08741283	0.37613058
6	-2.33401728	-1.48688674	2.23303056	1	0.03088272	3.40161324	0.07793060
6	-2.56541729	0.55711323	-2.53146935	6	0.93448275	4.17301321	1.90553057
6	-1.83011723	-0.20328675	2.92073059	1	0.52308273	5.07401323	0.01193060
1	-1.17451715	0.36811322	2.26353073	8	1.61178279	-1.41388679	-0.51806939
1	-1.28001738	-0.44788677	3.83833051	15	2.79398274	-2.31188679	-0.42176938
1	-2.67261720	0.44131327	3.19453073	8	4.17928267	-1.59058666	-0.75106937
6	-1.15631723	-2.43198681	1.92133069	8	3.67608285	2.02021337	-0.82706940
1	-0.48191726	-2.00968671	1.17533064	6	4.37558270	-0.22418675	-0.27576941
1	-1.52811718	-3.38518667	1.52883065	6	4.05628252	0.76751322	-1.39416933
1	-0.57941729	-2.64368677	2.83053064	1	5.41928244	-0.17668675	0.03783061
6	-3.21891713	-2.21328664	3.26213050	1	3.72808290	-0.01028675	0.57793063
1	-3.59351730	-3.17208672	2.88783073	1	4.93048239	0.96761328	-2.01746941
1	-4.07591724	-1.60668683	3.57433057	1	3.25098276	0.38251323	-2.02266955
1	-2.62581730	-2.42488670	4.15933037	8	2.79068279	-3.55468678	-1.40026939
6	-3.54551721	0.74111325	-3.70396948	1	-6.19651747	-1.57008672	-0.44166940
1	-4.41461754	1.34661317	-3.42556930	1	0.07688272	4.75161314	2.27033067
1	-3.90461707	-0.21428676	-4.10116959	1	1.82338262	4.76611328	2.16303062
1	-3.03401732	1.26231313	-4.52136946	1	3.00488281	4.05751324	0.22923060

6	-1.38051724	-0.25928676	-3.08876944	1	2.18378282	4.23661327	-1.30786943
1	-0.65221727	-0.50748676	-2.31526947	6	2.51688290	-3.37928677	-2.81006932
1	-0.86511725	0.29511324	-3.88256931	1	1.55888271	-2.87498665	-2.94526935
1	-1.74101710	-1.20418668	-3.51106930	1	2.47368288	-4.38278675	-3.22976947
6	-2.11911726	1.97261333	-2.11446929	1	3.32398272	-2.81268668	-3.27926946
1	-2.98881721	2.56641340	-1.81226933				

I-4_PC

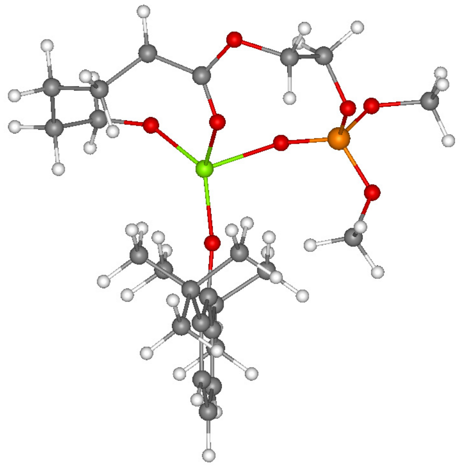

Zero-point vibrational energy	1677306.1 (Joules/Mol)
	400.88577 (Kcal/Mol)
Zero-point correction=	0.638852 (Hartree/Particle)
Thermal correction to Energy=	0.678429
Thermal correction to Enthalpy=	0.679373
Thermal correction to Gibbs Free Energy=	0.566976
Sum of electronic and zero-point Energies=	-2081.499513
Sum of electronic and thermal Energies=	-2081.459937
Sum of electronic and thermal Enthalpies=	-2081.458993
Sum of electronic and thermal Free Energies=	-2081.571389

cartesian

12	0.69860518	0.29152530	0.82502133	1	-1.23529482	-2.70487452	3.03842115
8	3.16020513	-3.54417467	1.05952132	1	-0.66229486	-2.07047462	1.49072134
8	-0.94459474	-0.15427467	0.02382131	1	2.85140514	-5.50627470	0.42962131
6	3.67540503	-4.81377506	0.60882133	8	2.09390521	0.84802526	-0.68377870
1	4.30720520	-5.18157482	1.41592133	6	3.10620499	1.53802538	-0.55757868
1	4.26830530	-4.69097471	-0.30117869	8	1.26910520	1.49112535	2.11752129
6	-2.22559500	-0.33477467	-0.28277868	6	0.99940515	2.54722548	2.97312117
6	-2.72709489	0.11842531	-1.54727864	6	0.62310517	3.85462546	2.26392126
6	-4.04739475	-0.18707469	-1.89197862	1	1.87580526	2.74832535	3.62092113
1	-4.44259501	0.12942532	-2.85157871	1	0.17260519	2.30392528	3.66502118

6	-4.89129496	-0.88577467	-1.03607869
6	-4.41989470	-1.26067472	0.21702132
1	-5.10359478	-1.77267480	0.88552129
6	-3.11099482	-0.99437469	0.63192129
6	-1.86659467	0.96402532	-2.50617886
6	-2.67239499	-1.38777471	2.05642128
6	-0.63229477	0.18032533	-2.99217868
1	0.01450518	-0.09877469	-2.16097879
1	-0.04609481	0.78812534	-3.69367886
1	-0.94239473	-0.73077470	-3.51737881
6	-1.42919481	2.26272535	-1.80007863
1	-0.88769484	2.05132532	-0.87687868
1	-2.30279493	2.87142539	-1.54327869
1	-0.78099477	2.85892534	-2.45467877
6	-2.63849497	1.38942528	-3.76767874
1	-3.52729487	1.98292542	-3.52997875
1	-2.94949484	0.53192532	-4.37457848
1	-1.98919475	2.01202536	-4.39377832
6	-3.81739473	-2.01877475	2.86992121
1	-4.17539501	-2.95557451	2.42932129
1	-4.67029476	-1.34117460	2.98172116
1	-3.45389485	-2.25297451	3.87682128
6	-2.23069501	-0.13507468	2.84082127
1	-1.42239475	0.40912533	2.34962130
1	-1.88819468	-0.41067469	3.84552121
1	-3.07089496	0.55932534	2.94872117
6	-1.54219472	-2.43437457	2.02062130
1	-1.88449466	-3.34737468	1.51972139
1	-0.27009478	3.67012548	1.65062141
1	0.32100520	4.57712507	3.03592134
6	3.21660519	2.74042535	0.31662130
6	2.09280515	3.77212548	0.10952131
1	1.20740521	3.27542543	-0.29677871
6	1.69730520	4.52412510	1.39212132
1	2.42190504	4.48802519	-0.65297872
8	1.76260519	-1.46887469	0.88052130
15	2.30410504	-2.61517453	0.10522132
8	3.24450517	-2.18207479	-1.12117863
8	4.24460506	1.19022536	-1.17247868
6	4.34290504	-1.26247478	-0.92677867
6	4.23010540	-0.06757468	-1.88097858
1	5.25310516	-1.83287477	-1.12497866
1	4.37980509	-0.91067469	0.10742132
1	5.09670496	-0.00397468	-2.53857875
1	3.32170510	-0.15367469	-2.47877884
8	1.28290522	-3.60197473	-0.57257867
1	-5.91199493	-1.11477470	-1.33287859
1	1.31550527	5.50902510	1.09602141
1	2.59420514	4.72452497	1.99512136
6	0.20890519	-3.13547468	-1.43917859
1	-0.29179481	-2.27257466	-0.99687868
1	-0.48249480	-3.97197461	-1.51947868
1	0.62400520	-2.88957453	-2.41777873
1	4.21030521	3.18322539	0.21762133
1	3.09900498	2.31212544	1.32302141

TS-45_PC

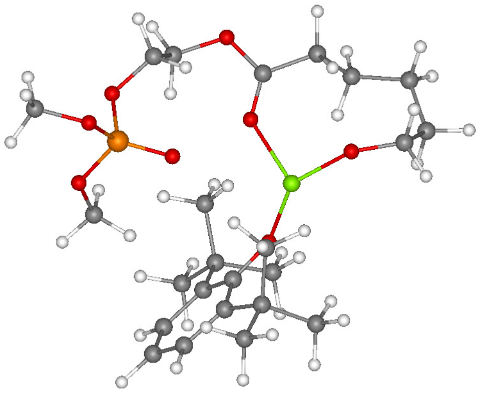

Zero-point vibrational energy 1675725.9 (Joules/Mol)
400.50810 (Kcal/Mol)

Zero-point correction=	0.638250 (Hartree/Particle)
Thermal correction to Energy=	0.677414
Thermal correction to Enthalpy=	0.678358
Thermal correction to Gibbs Free Energy=	0.564735
Sum of electronic and zero-point Energies=	-2081.478881
Sum of electronic and thermal Energies=	-2081.439717
Sum of electronic and thermal Enthalpies=	-2081.438773
Sum of electronic and thermal Free Energies=	-2081.552396

cartesian

12	1.30094266	-0.24956931	0.86555600	1	0.77594268	0.52253067	2.87405610
8	-4.13595724	-3.14476943	1.41965604	1	-0.80465734	0.12683070	2.05235600
8	0.74014258	1.35163069	0.08955602	1	-6.01725769	-2.86286926	0.56745601
6	-5.34535742	-3.67586923	0.84965605	8	0.86504269	-1.81116939	-0.40254396
1	-5.80265760	-4.28476954	1.62905598	6	1.45164263	-2.87006927	-0.65394396
1	-5.12075758	-4.29726934	-0.02154398	8	2.71184254	-0.97906935	1.78455603
6	-0.31835741	2.14493060	-0.04924398	6	4.02994251	-1.01886928	2.21215606
6	-0.70805740	2.59443069	-1.34964395	6	5.05804253	-0.89226937	1.08115602
6	-1.87475741	3.35753059	-1.46284401	1	4.22734261	-1.96226931	2.75715613
1	-2.20035744	3.70293069	-2.43884397	1	4.23864269	-0.21126932	2.93625593
6	-2.64145756	3.70473075	-0.35334396	1	4.86704254	0.05023068	0.54835600
6	-2.21095753	3.32633066	0.91515601	1	6.05124235	-0.78866935	1.54045606
1	-2.79825759	3.64013076	1.77195597	6	2.84214258	-3.18476939	-0.21034399
6	-1.04935741	2.57063055	1.10245597	6	3.89394236	-2.23746920	-0.82804400
6	0.14684260	2.28763056	-2.59344387	1	3.44404244	-1.26036930	-1.03254402
6	-0.51855743	2.28453064	2.51975608	6	5.13504267	-2.03646922	0.05845602
6	0.17394260	0.77383065	-2.88054395	1	4.19154263	-2.64626932	-1.80044401
1	0.56354260	0.21543068	-2.02924395	8	-1.98185742	-1.77106929	1.35105598
1	0.80454266	0.56103063	-3.75334406	15	-3.12205744	-2.27136922	0.55855602
1	-0.83515739	0.40593067	-3.10194397	8	-2.67425752	-3.20896935	-0.69104397
6	1.58424258	2.80863070	-2.38514400	8	0.84774268	-3.83136940	-1.34904397
1	2.05174255	2.35093069	-1.51244402	6	-1.45905733	-3.96706939	-0.57384396
1	1.57914269	3.89433050	-2.23934388	6	-0.52955741	-3.58546925	-1.72564399
1	2.19814253	2.59113073	-3.26824403	1	-1.72235739	-5.02736950	-0.60214400
6	-0.39775741	2.97243071	-3.85874391	1	-0.96735740	-3.74846935	0.37665603
1	-0.43355739	4.06213045	-3.75584388	1	-0.68715739	-4.19916916	-2.61214399
1	-1.39955735	2.61933064	-4.12754440	1	-0.66105735	-2.53276944	-1.97384405
1	0.26224259	2.74583054	-4.70364428	8	-4.08885717	-1.22696936	-0.14674398
6	-1.48745739	2.78453064	3.60625601	1	-3.54885745	4.29183054	-0.47364396
1	-2.45855737	2.28013062	3.55495596	1	5.98744249	-1.83256936	-0.60104400
1	-1.65375733	3.86453056	3.54595613	1	5.37704229	-2.97796941	0.57045603

1	-1.05995739	2.57943058	4.59375572	6	-3.53825760	-0.11536932	-0.89584398
6	0.80934262	3.04953074	2.71095610	1	-2.86305737	0.47253069	-0.27294397
1	1.54934263	2.75353074	1.96325600	1	-4.39055729	0.49333069	-1.19204402
1	1.22604263	2.85573077	3.70715594	1	-3.01975751	-0.48606932	-1.78254402
1	0.64594257	4.12803078	2.61605597	1	3.07244253	-4.22986937	-0.42824399
6	-0.29365739	0.76633060	2.78265595	1	2.82704258	-3.02116942	0.87265605
1	-0.71105742	0.46173069	3.74795604				

I-5_PC

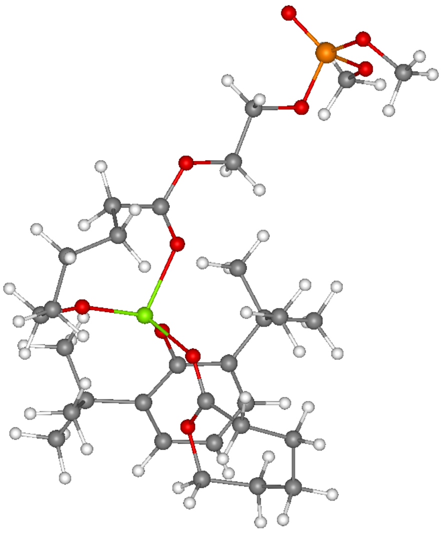

Zero-point vibrational energy	2091309.6 (Joules/Mol)
	499.83499 (Kcal/Mol)
Zero-point correction=	0.796538 (Hartree/Particle)
Thermal correction to Energy=	0.846224
Thermal correction to Enthalpy=	0.847169
Thermal correction to Gibbs Free Energy=	0.704841
Sum of electronic and zero-point Energies=	-2466.366023
Sum of electronic and thermal Energies=	-2466.316337
Sum of electronic and thermal Enthalpies=	-2466.315392
Sum of electronic and thermal Free Energies=	-2466.457719

cartesian

8	8.20359135	-0.16865267	-0.95956987	6	-1.30060852	-2.62235260	0.76143008
6	7.89339161	-0.79575270	-2.21596980	6	-3.76970816	-0.23085265	2.60673022
1	8.53209114	-0.31695265	-2.95786977	6	0.19319150	-2.72135258	0.39363009
1	6.84279156	-0.64115268	-2.47646976	6	-4.09580851	0.97574735	1.70623004
1	8.11269093	-1.86435258	-2.16776991	1	-3.20800829	1.43124735	1.26633000
8	1.23279142	1.57554734	0.27893010	1	-4.58810854	1.76024735	2.29403019
6	1.98279154	2.53424740	0.48623008	1	-4.77850819	0.68764734	0.89893013

8	-1.29560852	3.10914731	0.94553012	6	-2.77720833	0.19174734	3.71053028
6	-1.89000857	4.35414743	0.97843009	1	-1.81020844	0.47624731	3.29553008
6	-1.50310862	5.26074743	-0.19816990	1	-2.61760855	-0.63165265	4.41573000
1	-1.63490856	4.88604736	1.91703010	1	-3.17750835	1.04524732	4.27083015
1	-2.99360847	4.27514744	0.97993010	6	-5.08320856	-0.60235268	3.32173014
1	-1.77510846	4.74664736	-1.13106990	1	-4.96370840	-1.46795261	3.98163009
1	-2.12730837	6.16474724	-0.14826991	1	-5.89920855	-0.81285268	2.62113023
6	1.52409148	3.93294740	0.74143010	1	-5.40410852	0.24224734	3.94143009
6	0.99099147	4.59434748	-0.55026990	6	0.62779152	-4.17085266	0.10993010
1	0.53079152	3.83494735	-1.19057000	1	0.13469151	-4.59365273	-0.77256989
6	-0.03670853	5.70814753	-0.28666991	1	0.43409151	-4.83275270	0.96043009
1	1.84569144	4.99804735	-1.10596991	1	1.70639145	-4.19165277	-0.08446990
8	7.86989164	0.19724734	1.55993009	6	1.05669141	-2.22455263	1.57173002
15	7.36879158	-0.48895270	0.35853010	1	0.82399154	-1.18995261	1.82553005
8	5.82269144	-0.15505266	-0.00276990	1	2.12219143	-2.29795265	1.31633008
8	3.30449152	2.37944746	0.47913009	1	0.88139153	-2.84255266	2.45923018
6	5.31519175	1.15864742	0.28593010	6	0.51629150	-1.90845263	-0.87336987
6	3.80409145	1.04774737	0.23003009	1	-0.06500852	-2.28125262	-1.72536993
1	5.67639160	1.87074733	-0.46316987	1	1.57929146	-2.00285268	-1.13056993
1	5.65119171	1.48384738	1.27373004	1	0.29639149	-0.84975266	-0.74146986
1	3.46179152	0.70524734	-0.74896991	8	-1.18410850	1.22854733	-1.67386997
1	3.42379141	0.36344731	0.99103010	6	-2.11140871	0.64044732	-2.23866987
8	7.34019184	-2.08705258	0.35233009	8	-3.01320839	0.05054734	-1.47816992
1	0.04089150	6.43604755	-1.10416996	6	-4.11640835	-0.73175269	-2.00486994
1	0.23959148	6.25714731	0.62413013	6	-3.68110847	-1.97675252	-2.75746989
6	6.77039146	-2.77985263	1.48003006	1	-4.75380850	-0.07715267	-2.61086988
1	7.23789167	-2.44315267	2.40753007	1	-4.65680838	-1.00685263	-1.09986997
1	6.97719145	-3.83765268	1.32063007	1	-2.91560841	-2.48665261	-2.16246986
1	5.69039154	-2.61655259	1.51723003	1	-4.54720831	-2.64835262	-2.77466989
1	2.34039164	4.51794720	1.17133009	6	-2.20850849	0.59554732	-3.74026990
1	0.69599152	3.84544730	1.45063007	6	-2.00300837	-0.81185269	-4.32916975
12	-0.85670853	1.40324736	0.39643008	1	-1.11920846	-1.26975262	-3.87136984
8	-1.17310846	-0.27815264	1.20213008	6	-3.21430874	-1.73365259	-4.19266987
6	-1.87510860	-1.39885259	1.24533010	1	-1.76940858	-0.68985265	-5.39216995
6	-3.19500828	-1.42185259	1.80983007	1	-4.06220818	-4.63325262	0.98493010
6	-3.95270824	-2.59045267	1.67423010	1	-2.97700834	-2.69695258	-4.65736961
1	-4.96520853	-2.62275267	2.06313014	1	-4.04700851	-1.31255257	-4.77276993
6	-3.44650841	-3.74135256	1.07583010	1	-3.18460846	0.98974735	-4.04906988
6	-2.11670828	-3.75425267	0.66573012	1	-1.44310856	1.27944744	-4.10926962
1	-1.71130860	-4.68195248	0.27433008				

S1.7. LL sequence, mononuclear mechanism

I-1_LL

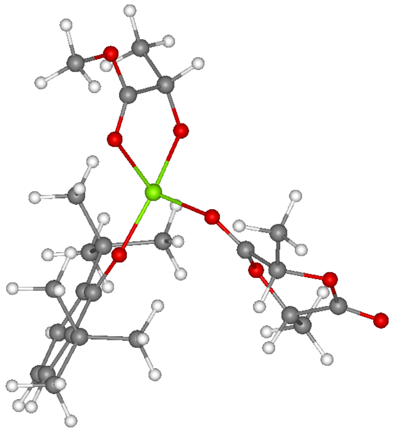

Zero-point vibrational energy	1512976.3 (Joules/Mol)
	361.61000 (Kcal/Mol)
Zero-point correction=	0.576262 (Hartree/Particle)
Thermal correction to Energy=	0.614223
Thermal correction to Enthalpy=	0.615167
Thermal correction to Gibbs Free Energy=	0.501162
Sum of electronic and zero-point Energies=	-1737.213316
Sum of electronic and thermal Energies=	-1737.175356
Sum of electronic and thermal Enthalpies=	-1737.174412
Sum of electronic and thermal Free Energies=	-1737.288417

cartesian

8	-1.82028842	-1.42980576	-0.37500733	6	2.51421142	3.28749442	0.97629267
6	-4.08538818	-0.72760570	-0.80200732	1	3.08151150	3.63079429	1.83519268
6	-2.77188849	-0.73690581	-0.04470732	6	1.97221160	1.99879420	0.97329271
8	-2.68808842	0.06179425	1.00569272	6	0.31601161	2.02799439	-2.55650735
6	-3.75938845	1.02419424	1.22529268	6	2.20821142	1.06499422	2.17359281
6	-5.11728811	0.34599426	1.08219266	6	-1.15778840	1.73399425	-2.21140718
8	-5.19058800	-0.59640574	0.11149268	1	-1.21068835	0.96799421	-1.43750727
8	-6.07348824	0.60819423	1.76349270	1	-1.70028830	1.38729417	-3.10110736
1	-4.06098843	0.15589425	-1.45670736	1	-1.65028834	2.64259434	-1.84450734
6	-4.30638838	-1.97620583	-1.62900734	6	0.97441155	0.78309429	-3.18550730
6	-3.54348850	1.64609420	2.58539271	1	1.00001168	-0.05480576	-2.48850727
1	-3.67138839	1.78609419	0.44019267	1	2.00471163	1.00819421	-3.48150730
1	-4.31448841	2.39659429	2.76449275	1	0.42651159	0.47379425	-4.08480740
1	-2.56168842	2.12099433	2.61929274	6	0.30811161	3.12069440	-3.63940740
1	-3.60718846	0.88919425	3.36919260	1	1.31841159	3.37749434	-3.97480726

1	-5.26028824	-1.89660573	-2.15340734	1	-0.18618840	4.03859425	-3.30320740
1	-4.32318830	-2.86170578	-0.99090731	1	-0.24198841	2.75639439	-4.51480722
1	-3.50318837	-2.08330584	-2.35910726	6	3.00371146	-0.17810576	1.71959269
12	0.20461160	-1.38890576	0.23319268	1	4.01781178	0.11619425	1.42799270
8	0.31901157	-2.77470565	1.53379273	1	2.56121159	-0.67010576	0.85109270
6	0.78061157	-3.98930573	1.10319269	1	3.07691145	-0.91330576	2.52829266
6	1.10891151	-3.92460561	-0.39080733	6	0.86821157	0.67949426	2.83549261
8	1.53521156	-5.04500580	-0.95240736	1	0.43321157	1.55649424	3.32829261
6	1.83911157	-4.98810577	-2.36100721	1	1.01021171	-0.10120575	3.59039259
6	2.00851154	-4.48020601	1.89059269	1	0.12741160	0.32659426	2.11599278
8	0.96821159	-2.87980556	-1.04350734	6	3.05221152	1.72069418	3.28139281
1	0.94731158	-4.71810579	-2.92910719	1	2.56741142	2.60769439	3.70259261
1	2.17421150	-5.98990583	-2.62380719	1	4.04821157	2.00709438	2.92849278
1	2.62631154	-4.25610590	-2.54820728	1	3.18911147	1.00429416	4.09939289
8	0.65741158	0.36919424	-0.20930734	1	2.78901148	5.15489388	-0.06650732
6	1.22191167	1.57289422	-0.16980731	1	0.00891158	-4.78360605	1.17799270
6	1.07661152	2.45619440	-1.28770733	1	2.34371161	-5.47050571	1.56739271
6	1.64811158	3.72979426	-1.21440732	1	1.73031163	-4.52560568	2.94609261
1	1.54701161	4.41549397	-2.04890728	1	2.83421159	-3.76940560	1.79029274
6	2.35671163	4.15769386	-0.09670732				

I-2_LL

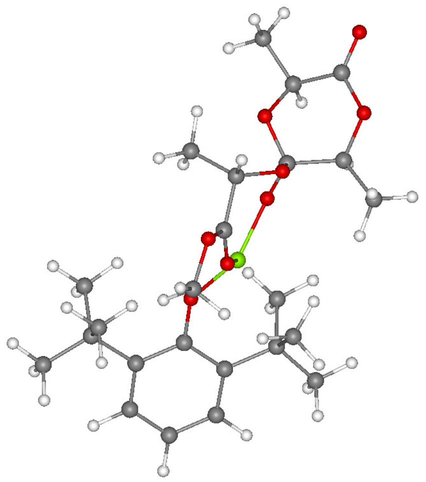

Zero-point vibrational energy	1519322.5 (Joules/Mol)
	363.12680 (Kcal/Mol)
Zero-point correction=	0.578679 (Hartree/Particle)
Thermal correction to Energy=	0.615067
Thermal correction to Enthalpy=	0.616012
Thermal correction to Gibbs Free Energy=	0.509377
Sum of electronic and zero-point Energies=	-1737.186784
Sum of electronic and thermal Energies=	-1737.150396

Sum of electronic and thermal Enthalpies= -1737.149452

Sum of electronic and thermal Free Energies= -1737.256087

cartesian

8	1.68072319	-0.92900151	0.78145951	1	-1.16517687	-1.06760144	-1.80194044
8	3.71452332	-0.45760149	1.74355936	1	-1.47927678	-2.70270157	-2.28234053
6	3.00292301	-1.06420159	0.64155942	1	-0.28067678	-2.47430158	-1.02984047
6	3.39162302	-2.57170153	0.54245943	6	-3.13907671	-3.51940179	-0.63954055
8	4.78822327	-2.81200147	0.87885946	1	-3.60397673	-3.49170136	-1.63504052
6	5.20462322	-2.31710148	2.08185935	1	-3.92207670	-3.72410178	0.10275948
6	4.19232321	-1.37650156	2.74175930	1	-2.43477678	-4.36450148	-0.61974055
8	6.29982328	-2.59860158	2.51685929	6	-2.00287676	3.26999831	-0.94664049
12	0.23452324	-0.27520150	-0.21554053	1	-2.46717691	3.27319837	-1.94414043
8	3.55882311	-0.46140152	-0.57244051	1	-1.16867685	2.55939841	-0.95164055
6	3.43082333	0.92609847	-0.86044049	1	-1.60817683	4.27979851	-0.75234056
6	2.28232336	1.15609848	-1.84844065	6	-2.39887667	2.95949841	1.53815937
8	2.62232304	1.96529841	-2.84554052	1	-3.15027666	2.75529838	2.31465936
6	1.56442320	2.30869842	-3.79224062	1	-1.99137676	3.96659851	1.71875930
6	3.28392315	1.92779851	0.29855949	1	-1.59067678	2.22679853	1.63855934
8	1.13822317	0.68159848	-1.76524043	6	-4.14617682	3.96819854	0.08995944
1	1.21722317	1.40299845	-4.30164051	1	-4.91777658	3.79959846	0.85455948
1	2.03402328	3.00039840	-4.49574041	1	-4.63737679	4.01919842	-0.89254057
1	0.72982323	2.78309846	-3.26384068	1	-3.69507694	4.95179844	0.28705949
8	-1.43447673	0.49279848	0.10385948	1	-6.57137680	-0.10180151	-0.79714054
6	-2.74117661	0.33089849	-0.12514052	1	4.34862328	1.18509853	-1.41044044
6	-3.29097700	-0.97170156	-0.36134052	1	3.21202326	2.94339848	-0.11694053
6	-4.66507673	-1.09570146	-0.60054052	1	4.16542339	1.86959839	0.94385952
1	-5.09657669	-2.07930160	-0.78174055	1	2.40032339	1.72349846	0.91385943
6	-5.50367689	0.01569849	-0.60974056	1	2.76392317	-3.07350159	1.29625940
6	-4.96567678	1.28069854	-0.37354052	6	3.16312313	-3.20170140	-0.81914055
1	-5.63657665	2.13809848	-0.38074052	6	4.80512333	-0.57290155	3.87335920
6	-3.60107660	1.47659850	-0.12934053	1	3.35042334	-1.98140168	3.13435936
6	-2.38217688	-2.21470165	-0.32294053	1	5.18762350	-1.24970150	4.64665937
6	-3.04467678	2.89189839	0.13395947	1	4.04612350	0.08639848	4.31285954
6	-1.79437673	-2.39170146	1.10115957	1	5.64272356	0.03179848	3.50405931
1	-1.30827677	-1.48350167	1.48225951	1	3.34422302	-4.28260136	-0.76334053
1	-1.07127678	-3.22140145	1.12965941	1	3.83812332	-2.76230168	-1.56164050
1	-2.60547686	-2.62610149	1.80395937	1	2.12652302	-3.03990149	-1.14234054
6	-1.25307679	-2.09040165	-1.39864051				

TS-13_LL

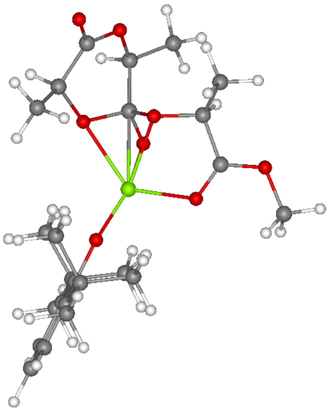

Zero-point vibrational energy	1516662.9 (Joules/Mol)
	362.49112 (Kcal/Mol)
Zero-point correction=	0.577666 (Hartree/Particle)
Thermal correction to Energy=	0.613556
Thermal correction to Enthalpy=	0.614501
Thermal correction to Gibbs Free Energy=	0.509629
Sum of electronic and zero-point Energies=	-1737.195133
Sum of electronic and thermal Energies=	-1737.159243
Sum of electronic and thermal Enthalpies=	-1737.158299
Sum of electronic and thermal Free Energies=	-1737.263170

cartesian

12	0.55945081	-0.22443183	-0.48413911	1	-1.20244908	2.31796837	-0.02063915
8	-1.18204927	-0.04123183	0.08546085	8	2.04645085	-1.38383186	-1.26863921
6	-2.50364923	-0.20773183	0.12726085	6	2.72085094	-1.14363182	-0.19213915
6	-3.12154913	-1.31283188	-0.53753918	8	1.92725086	-1.48793185	0.95546085
6	-4.51064920	-1.44993186	-0.45463914	8	0.94885081	1.47736812	-1.61273921
1	-5.00154924	-2.28253174	-0.94753915	8	2.73565078	0.39586818	0.05676085
6	-5.29794931	-0.54503185	0.24816084	6	2.11335087	1.86696815	-1.71133912
6	-4.68884897	0.53176814	0.88446081	6	3.27125096	1.21646821	-0.96553916
1	-5.31914902	1.22986817	1.42526078	1	3.76575089	0.60426813	-1.72953916
6	-3.30624914	0.73296815	0.84416085	1	-6.37604904	-0.67623186	0.29866087
6	-2.29164910	-2.33863163	-1.32983911	8	2.46535087	2.86076832	-2.50003910
6	-2.68134904	1.94776821	1.55476081	6	1.40905070	3.47706819	-3.27603912
6	-1.56914926	-1.64733183	-2.50813913	1	1.89795089	4.26616812	-3.84293914
1	-1.06634927	-0.72093183	-2.21653914	1	0.64965081	3.88706827	-2.60983896
1	-0.83404917	-2.31543183	-2.97103906	1	0.95925081	2.73976827	-3.94203901
1	-2.29554915	-1.35733187	-3.27473903	6	4.11945057	-1.73833179	-0.00333915
6	-1.30094910	-3.06533170	-0.39523914	6	2.56245089	-1.47563183	2.25166106
1	-0.68334919	-2.38263178	0.19036084	6	3.99655080	-0.93793184	2.25066090

1	-1.85324907	-3.67873168	0.32496086	1	2.65325093	-2.52903175	2.55466104
1	-0.63734919	-3.72613168	-0.96483916	1	3.93595099	-2.80313182	0.19276084
6	-3.15744901	-3.44303179	-1.96303916	8	4.75095081	-1.15613186	1.14926088
1	-3.69124889	-4.03053188	-1.20923913	8	4.48645067	-0.41073185	3.21986103
1	-3.89054918	-3.04243183	-2.67063904	6	4.25495052	2.22126818	-0.37543914
1	-2.51134920	-4.13363171	-2.51723909	1	3.75795078	2.85326838	0.36466086
6	-3.73324919	2.81686831	2.26536083	1	4.68485069	2.85586834	-1.15313911
1	-4.46974897	3.23186827	1.56886089	1	5.05575085	1.67586815	0.12766086
1	-4.26934910	2.26616836	3.04566097	6	1.68325090	-0.74143183	3.24426103
1	-3.23254919	3.66246819	2.75026107	1	1.62975073	0.32086819	2.99716091
6	-1.68394923	1.48676813	2.63586092	1	2.09275079	-0.84043187	4.25086117
1	-0.88424915	0.88366818	2.20556092	1	0.67685080	-1.16493189	3.22376084
1	-1.23714924	2.35286832	3.14036083	6	5.07745075	-1.62523186	-1.17143917
1	-2.19294906	0.88216817	3.39466095	1	5.93655109	-2.27963161	-1.00403917
6	-1.97404909	2.85816836	0.52956080	1	5.45905066	-0.60883182	-1.28653920
1	-2.69604921	3.24886823	-0.19653915	1	4.57245111	-1.93433189	-2.08903909
1	-1.50974917	3.71406817	1.03626084				

I-3_LL

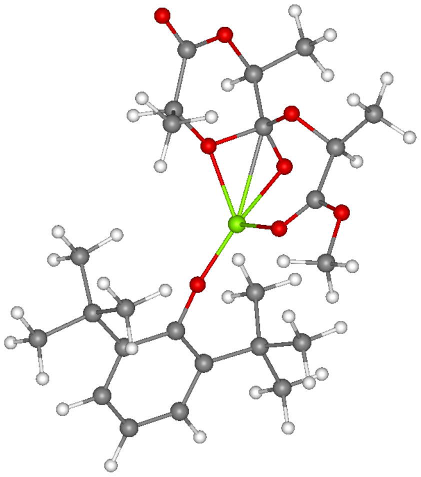

Zero-point vibrational energy	1516772.9 (Joules/Mol)
	362.51742 (Kcal/Mol)
Zero-point correction=	0.577708 (Hartree/Particle)
Thermal correction to Energy=	0.613538
Thermal correction to Enthalpy=	0.614483
Thermal correction to Gibbs Free Energy=	0.510079
Sum of electronic and zero-point Energies=	-1737.206633
Sum of electronic and thermal Energies=	-1737.170803
Sum of electronic and thermal Enthalpies=	-1737.169858
Sum of electronic and thermal Free Energies=	-1737.274262

cartesian

8	-1.33347368	0.07431158	0.19583914	8	3.69382644	-0.51208842	-0.04446086
6	-2.66207361	0.14941159	0.25663912	6	3.44652629	-1.59208834	-0.92736083
6	-3.43087363	-0.98608845	0.65563911	1	3.03482628	-2.43718839	-0.35866088
6	-4.82317400	-0.86098844	0.69903910	6	2.34162641	-1.24128830	-1.93046093
1	-5.43317366	-1.70678830	0.99783915	12	0.44522631	-0.32718843	0.24103914
6	-5.46627378	0.32691157	0.36993915	1	-6.55037403	0.39561158	0.41393912
6	-4.70667410	1.42661166	-0.01496086	8	2.61642647	-1.56128836	-3.17886090
1	-5.22637367	2.34501171	-0.26596087	8	1.25602627	-0.73918843	-1.62196076
6	-3.31157351	1.37481165	-0.08416086	6	1.58122635	-1.31308842	-4.16006088
6	-2.76367354	-2.31968832	1.04103911	1	2.00112629	-1.65028834	-5.10506105
6	-2.51157355	2.61621165	-0.52126086	1	1.34892631	-0.24828842	-4.19376087
6	-1.97097373	-2.89628839	-0.15126087	1	0.68402636	-1.87898839	-3.90686083
1	-1.25557375	-2.17998838	-0.56286085	6	4.76792622	-1.97788835	-1.57246077
1	-1.42807364	-3.80178833	0.14593914	8	1.88202643	-1.27858841	1.20323920
1	-2.65217352	-3.16278839	-0.96666092	6	3.61932635	-0.04278841	2.31713915
6	-1.85627365	-2.13428831	2.27463913	6	2.79542637	-0.34618843	1.06843913
1	-1.09337378	-1.36688840	2.12993908	8	2.03042626	0.91241157	0.78933913
1	-2.45327353	-1.82828832	3.14003921	6	2.75132632	2.14741158	0.65783912
1	-1.34887362	-3.07268834	2.52823925	6	4.10972643	2.14861155	1.36363924
6	-3.79097342	-3.39958835	1.42773914	8	4.48382616	1.10051167	2.11663914
1	-4.38557386	-3.11108828	2.30063915	8	4.83982611	3.10751176	1.25713921
1	-4.47597408	-3.63328838	0.60613912	1	2.89842629	0.21001159	3.10143924
1	-3.26337361	-4.32488823	1.68563914	6	4.49602604	-1.19428837	2.75583911
6	-3.42037368	3.81881166	-0.83186090	6	2.89102626	2.57441163	-0.79786086
1	-4.11377382	3.61281157	-1.65396082	1	2.15222621	2.89251161	1.19273913
1	-4.00297403	4.13431168	0.03993914	1	4.64552593	-2.83698845	-2.23486090
1	-2.80087352	4.67061186	-1.13486087	1	5.47472620	-2.24138832	-0.78326088
6	-1.55787373	3.06781173	0.60423911	1	5.18532610	-1.14848840	-2.14736080
1	-0.86737365	2.27361155	0.89273912	1	3.34542632	3.56561160	-0.84586090
1	-0.97467369	3.94091153	0.28483915	1	1.90232635	2.61471152	-1.26206076
1	-2.12667370	3.35321164	1.49573922	1	3.52082634	1.87321162	-1.34866083
6	-1.72307372	2.31721163	-1.81316078	1	5.02012634	-0.93288845	3.67823911
1	-2.41047359	2.07421160	-2.63096070	1	5.23772621	-1.42438841	1.98773921
1	-1.13777375	3.19451165	-2.11646080	1	3.87602639	-2.07458830	2.93883920
1	-1.04567361	1.47101164	-1.68736076				

TS-34_LL

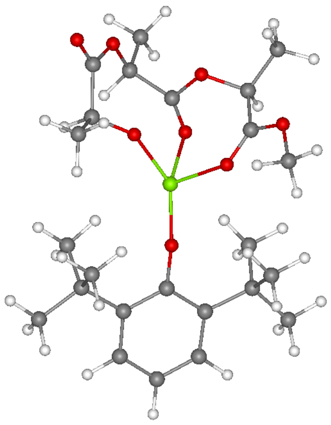

Zero-point vibrational energy	1514951.2 (Joules/Mol)
	362.08202 (Kcal/Mol)
Zero-point correction=	0.577014 (Hartree/Particle)
Thermal correction to Energy=	0.613392
Thermal correction to Enthalpy=	0.614336
Thermal correction to Gibbs Free Energy=	0.508324
Sum of electronic and zero-point Energies=	-1737.199817
Sum of electronic and thermal Energies=	-1737.163439
Sum of electronic and thermal Enthalpies=	-1737.162495
Sum of electronic and thermal Free Energies=	-1737.268507

cartesian

8	1.26826394	-0.05315214	-0.05739420	8	-3.68013620	0.88504785	0.55160576
6	2.58456373	-0.18795215	0.09410580	6	-3.24133635	2.21564794	0.23200580
6	3.38156366	0.95354784	0.41810581	1	-2.58113623	2.56694794	1.03400576
6	4.76086378	0.78064781	0.56680578	6	-2.36623621	2.19434786	-1.02399421
1	5.38826370	1.63024783	0.81420577	12	-0.56503612	-0.07875215	-0.03229420
6	5.37016392	-0.45895216	0.40680581	1	6.44556379	-0.56375217	0.52790576
6	4.58706379	-1.56245220	0.08760580	8	-2.73893595	3.05654788	-1.94759429
1	5.08016396	-2.52055216	-0.03659420	8	-1.37443614	1.48174787	-1.15599418
6	3.20176411	-1.46565211	-0.07589421	6	-1.91713607	3.12424779	-3.13869405
6	2.75876379	2.35044789	0.59910578	1	-2.36933613	3.90234780	-3.74949408
6	2.38176394	-2.71875215	-0.43529421	1	-1.93693614	2.16324782	-3.65359402
6	2.07536411	2.80294776	-0.70749420	1	-0.89243615	3.38134789	-2.86949420
1	1.31106389	2.09444785	-1.02929425	6	-4.48283625	3.08064795	0.13110580
1	1.60986388	3.78834796	-0.57239425	8	-1.71163607	0.49254787	1.60730577
1	2.81346369	2.88834786	-1.51279426	6	-3.52913618	-1.09925210	1.87480581
6	1.75256383	2.34754777	1.76790571	6	-2.83013630	0.08004786	1.22260582
1	0.95436382	1.61854780	1.62440574	8	-2.13533616	-1.14905214	-0.31199419
1	2.25916386	2.09914780	2.70670581	6	-2.72793627	-2.39915204	-0.48979419

1	1.29756391	3.33944774	1.88530576	6	-4.21293592	-2.33085227	-0.10789420
6	3.81026363	3.42254782	0.93610579	8	-4.53863621	-1.65155220	1.03330576
1	4.33076382	3.21294785	1.87650573	8	-5.10633612	-2.84435225	-0.73709422
1	4.55896378	3.53324795	0.14470580	1	-2.74843597	-1.83475220	2.08870578
1	3.31176376	4.39204788	1.05000579	6	-4.20963621	-0.67635214	3.17010593
6	3.26116371	-3.97295213	-0.58919424	6	-2.57193613	-2.90725207	-1.91759419
1	4.00026369	-3.86485219	-1.38979423	1	-2.28123617	-3.14975214	0.19100580
1	3.78976393	-4.22515202	0.33600581	1	-4.21083593	4.11734772	-0.07459420
1	2.62716389	-4.82945204	-0.84469426	1	-5.01783609	3.04174781	1.08220577
6	1.37026393	-3.04125214	0.68340576	1	-5.14753628	2.72754788	-0.65919423
1	0.70656383	-2.20055223	0.89770579	1	-3.02843618	-3.89255214	-2.03819418
1	0.75366390	-3.90665221	0.41130581	1	-1.50823617	-2.97545218	-2.15869403
1	1.89486396	-3.27975225	1.61470580	1	-3.05063629	-2.21785212	-2.61749411
6	1.66756380	-2.52475214	-1.78849423	1	-4.64893627	-1.55435216	3.64900589
1	2.40226364	-2.37665224	-2.58709407	1	-5.00663614	0.04264786	2.96710587
1	1.07216382	-3.41095209	-2.03979421	1	-3.48183632	-0.23005214	3.85220599
1	1.01056385	-1.65295219	-1.78659427				

I-4_LL

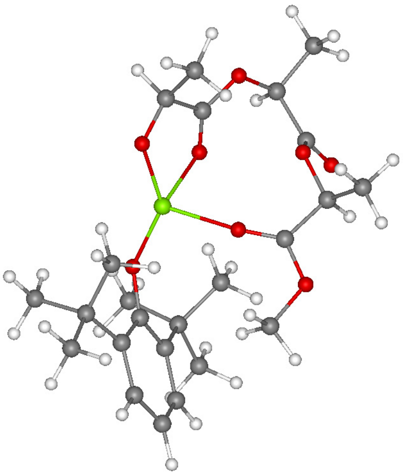

Zero-point vibrational energy	1516356.2 (Joules/Mol)
	362.41784 (Kcal/Mol)
Zero-point correction=	0.577550 (Hartree/Particle)
Thermal correction to Energy=	0.614647
Thermal correction to Enthalpy=	0.615591
Thermal correction to Gibbs Free Energy=	0.507992
Sum of electronic and zero-point Energies=	-1737.217347
Sum of electronic and thermal Energies=	-1737.180250
Sum of electronic and thermal Enthalpies=	-1737.179305
Sum of electronic and thermal Free Energies=	-1737.286905

cartesian

12	0.34762606	1.18167973	0.69020432	1	-1.43967390	-0.89832032	3.06810427
8	-1.29397392	0.41607967	1.19210422	8	1.32472610	2.80427980	0.90390432
6	-2.31117392	-0.13982034	0.53870434	6	3.87452602	-1.26782024	0.00830429
6	-3.21587396	0.68047965	-0.20719571	6	4.57512617	-0.55282032	1.16300428
6	-4.19177389	0.05497966	-0.98989564	8	4.29882574	0.86177963	1.20300424
1	-4.87017393	0.65217966	-1.59019578	6	3.01612592	1.20307970	1.30170429
6	-4.33607388	-1.32982028	-1.01749575	6	2.66392589	2.68667960	1.17350423
6	-3.52347398	-2.11152029	-0.20099571	8	3.77492595	-2.47182035	-0.02579571
1	-3.68807411	-3.18402028	-0.19059572	6	3.54962587	3.38527966	0.13240428
6	-2.51967406	-1.55512023	0.59980434	1	2.92752600	3.11237979	2.16610432
6	-3.17497396	2.21737981	-0.09319571	1	4.19112587	-1.02052033	2.07260442
6	-1.69687390	-2.45182037	1.54480422	6	6.08412600	-0.71182036	1.06730425
6	-3.47907400	2.61727977	1.36620426	8	3.45252609	-0.42562035	-0.96269566
1	-2.76737404	2.15607977	2.05440426	6	2.72112608	-0.99402040	-2.05379558
1	-3.42067409	3.70637965	1.48500431	6	1.26052606	-0.55932033	-1.95399570
1	-4.48767424	2.29907966	1.65030420	8	0.53262603	-1.16182029	-2.87269568
6	-1.80837381	2.81817961	-0.50929570	6	-0.88617384	-0.84482032	-2.91179562
1	-1.13527393	2.94947982	0.34290430	6	3.35742593	-0.53522032	-3.36159563
1	-1.31717396	2.22557974	-1.29019570	1	2.74652600	-2.08442020	-1.97629583
1	-1.93487394	3.82707977	-0.91779566	8	0.81462610	0.25057966	-1.15379572
6	-4.24187422	2.88417983	-0.98039573	1	-1.01737392	0.21357965	-3.13869572
1	-4.07527399	2.69347978	-2.04699564	1	-1.28747392	-1.46372032	-3.71089554
1	-5.25507402	2.55737972	-0.72649568	1	-1.35547388	-1.08652031	-1.95759571
1	-4.20667410	3.96887970	-0.83199567	1	-5.10117388	-1.79152024	-1.63709569
6	-2.21047401	-3.90242028	1.55900431	8	2.13982606	0.34287965	1.44790423
1	-3.26217413	-3.96442032	1.85770428	1	4.61292601	3.33277965	0.38410428
1	-2.09827399	-4.39752007	0.58790433	1	3.24582601	4.43347979	0.08830429
1	-1.62937391	-4.48212051	2.28470445	1	3.39502597	2.94537973	-0.85719568
6	-0.21927392	-2.51062036	1.11420429	1	6.55802584	-0.25082034	1.93660426
1	0.25642607	-1.53142023	1.17040431	1	6.47012615	-0.23352034	0.16380429
1	0.34492606	-3.18712020	1.76720428	1	6.33942604	-1.77302027	1.04810429
1	-0.12697393	-2.88942027	0.08930429	1	2.81092596	-0.94762033	-4.21159554
6	-1.80027401	-1.92482030	2.99110436	1	4.38922596	-0.89092034	-3.40309572
1	-2.84097409	-1.94912028	3.33260441	1	3.36022592	0.55517966	-3.43269563
1	-1.20987391	-2.55602026	3.66670442				

I-5_LL

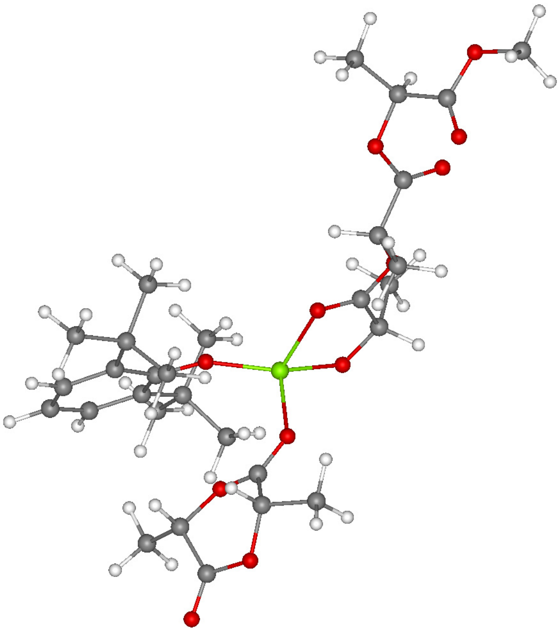

Zero-point vibrational energy	1887836.8 (Joules/Mol)
	451.20383 (Kcal/Mol)
Zero-point correction=	0.719039 (Hartree/Particle)
Thermal correction to Energy=	0.768340
Thermal correction to Enthalpy=	0.769284
Thermal correction to Gibbs Free Energy=	0.629422
Sum of electronic and zero-point Energies=	-2271.313314
Sum of electronic and thermal Energies=	-2271.264013
Sum of electronic and thermal Enthalpies=	-2271.263069
Sum of electronic and thermal Free Energies=	-2271.402931

cartesian

8	1.41838157	2.05061722	0.95030344	6	0.54788154	-1.51258278	-2.69589663
6	2.62498140	3.85281730	-0.09969656	1	0.18978155	-1.49498272	-1.66579652
6	2.48478174	2.47541714	0.51950347	1	0.69938153	-2.55738282	-2.98749661
8	3.57378149	1.73691726	0.57090342	1	-0.23011847	-1.09118295	-3.34499645
6	4.74558163	2.18001723	-0.17609654	6	2.25498152	-0.78478283	-4.33449697
6	5.01178122	3.65511727	0.09780344	1	2.42198133	-1.81288266	-4.67139673
8	3.90288162	4.42621708	0.22850345	1	3.15448141	-0.20018283	-4.55789661
8	6.11028147	4.13551712	0.19630346	1	1.44108152	-0.37138283	-4.94089699
1	2.57098150	3.70971727	-1.18899655	6	2.07988167	-3.20888281	1.93810344
6	1.54988158	4.82151699	0.34660345	1	2.10528135	-4.16968298	1.41260350
6	5.89748144	1.27751708	0.19700345	1	1.24108148	-2.63878274	1.53840351
1	4.50508165	2.06111717	-1.23999655	1	1.88448155	-3.40518284	2.99840355
1	6.77528143	1.56161714	-0.38569653	6	3.47398138	-1.18818283	2.60940337

1	5.63548136	0.24111716	-0.02459656
1	6.13898134	1.37971711	1.25680351
1	1.70638156	5.78391743	-0.14399655
1	1.58498156	4.96351719	1.42830348
1	0.56648153	4.43601704	0.07470345
12	0.69328153	0.09491716	1.29220343
8	-0.04081847	-0.01908283	3.03870344
6	-1.40401852	0.05381717	3.14560342
6	-2.02471852	0.28861716	1.76670349
8	-3.34141827	0.42941716	1.73840344
6	-3.97221851	0.63731718	0.45770347
6	-2.02551842	-1.19938278	3.78760338
8	-1.32681847	0.34751716	0.74270344
6	-4.06291866	2.12601709	0.14290345
6	-5.36061859	0.02371716	0.55760342
1	-3.40011835	0.11711717	-0.31289655
8	1.65878153	-0.89978284	0.03500345
6	2.73638153	-1.44278288	-0.51479656
6	2.94288158	-1.31958294	-1.92799664
6	4.13328123	-1.80908275	-2.47529650
1	4.31958151	-1.71118283	-3.53979659
6	5.09588146	-2.44508266	-1.69689655
6	4.84618139	-2.64178276	-0.34209657
1	5.58308125	-3.18608284	0.23920345
6	3.68268156	-2.17298269	0.27690345
6	1.86228156	-0.71868283	-2.84819651
6	3.42848158	-2.47808266	1.76610351
6	1.61168158	0.76651716	-2.52439642
1	1.27848148	0.88891715	-1.49379659
1	0.84178156	1.18221712	-3.18649650
1	2.52978134	1.34891725	-2.67929649
1	4.49678135	-0.79838282	2.65380335
1	3.13178158	-1.37208295	3.63310337
1	2.86278152	-0.38648283	2.19170356
6	4.49568129	-3.41318274	2.36440349
1	5.49748135	-2.97078276	2.35140347
1	4.53688145	-4.37548256	1.84360349
1	4.24758148	-3.61698270	3.41200352
1	6.01268148	-2.81648278	-2.14829659
1	-1.73241842	0.92591715	3.74720335
1	-3.11101866	-1.11888289	3.89460349
1	-1.57561851	-1.32448292	4.77520323
1	-1.78441846	-2.08718276	3.19430351
8	-5.80751848	-0.26108283	-0.68669653
8	-5.98681879	-0.16008282	1.57290351
6	-7.17111874	-0.69168282	-0.77939653
6	-8.09281826	0.50361717	-0.54279655
1	-7.37171841	-1.43578291	-0.00429656
6	-7.36531878	-1.28468275	-2.16709661
8	-9.35301781	0.08151717	-0.35509652
8	-7.74551868	1.66311717	-0.55199653
6	-10.32661819	1.11991715	-0.14329655
1	-11.27691841	0.60481715	-0.01299655
1	-10.36511803	1.78711724	-1.00659657
1	-10.07511806	1.69511724	0.74950343
1	-4.61221838	2.27551723	-0.78889656
1	-3.05791855	2.53911734	0.03230345
1	-4.58681870	2.65191722	0.94410348
1	-8.39231777	-1.63778281	-2.27989650
1	-6.68671846	-2.12978268	-2.30419660
1	-7.15751839	-0.54018283	-2.93969655

S1.8. LP sequence, mononuclear mechanism

I-1_LP

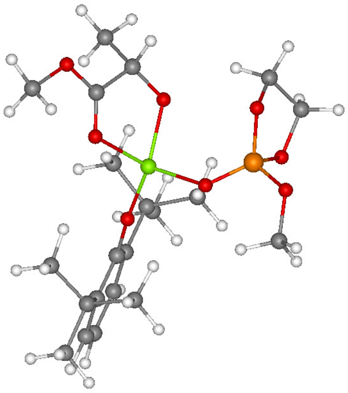

Zero-point vibrational energy	1445611.2 (Joules/Mol)
	345.50936 (Kcal/Mol)
Zero-point correction=	0.550604 (Hartree/Particle)
Thermal correction to Energy=	0.587427
Thermal correction to Enthalpy=	0.588371
Thermal correction to Gibbs Free Energy=	0.477829
Sum of electronic and zero-point Energies=	-1963.699849
Sum of electronic and thermal Energies=	-1963.663026
Sum of electronic and thermal Enthalpies=	-1963.662082
Sum of electronic and thermal Free Energies=	-1963.772624

cartesian

12	0.92811191	0.39101970	-0.07618640	1	-1.11408806	-2.71438026	2.30341363
8	-0.90228808	0.10001969	-0.06178640	1	-0.01908806	-1.70538032	3.26951361
6	-2.18398809	-0.21938032	0.06521360	1	-0.10218808	-1.51418030	1.50911355
6	-3.04738808	-0.16918030	-1.07568634	1	-5.96438789	-1.14838028	0.42151362
6	-4.39358807	-0.50718033	-0.90988642	8	1.86371195	-1.04768026	-1.18008637
1	-5.06718779	-0.47438031	-1.75978637	15	3.02651191	-1.92128038	-0.84098643
6	-4.91268778	-0.89028031	0.32241359	8	4.42691231	-1.25468028	-0.47028640
6	-4.06788778	-0.93758035	1.42601359	8	2.79431176	-2.76888037	0.50151360
1	-4.48928785	-1.23758030	2.37971377	6	4.63931227	-1.36788034	0.96591353
6	-2.71108818	-0.61188030	1.33711362	6	3.95851183	-2.67878032	1.35671353
6	-2.52278805	0.24381970	-2.46318626	1	5.71651220	-1.37908030	1.13161361
6	-1.82358801	-0.67438030	2.59371376	1	4.15381193	-0.51048028	1.43991363
6	-1.43898797	-0.74548030	-2.93888640	8	3.39691186	-2.88508034	-2.03738642
1	-0.61678803	-0.82058030	-2.22668624	6	2.39511180	-3.30998039	-2.99438620
1	-1.03348804	-0.43208033	-3.90988636	1	2.94941187	-3.66868043	-3.85998631
1	-1.86898792	-1.74648035	-3.06028628	1	1.75261199	-2.47328043	-3.26918626

6	-1.96728814	1.68211973	-2.42318630	1	1.80231202	-4.11998034	-2.56528640
1	-1.16818798	1.79021966	-1.68988645	8	2.33631182	0.79961967	1.19131362
1	-2.76278806	2.38791966	-2.15868640	6	2.87881184	2.05871964	1.10821354
1	-1.57658803	1.96911979	-3.40798640	1	3.97911191	2.03371978	0.96721363
6	-3.62488818	0.23121969	-3.53718638	6	2.35121202	2.77191973	-0.13758640
1	-4.43258810	0.93591964	-3.31228638	8	2.88281178	3.95451975	-0.40198642
1	-4.06398773	-0.76328033	-3.67198634	8	1.48101199	2.26711965	-0.85828638
1	-3.19228816	0.52861965	-4.49948645	6	2.38501191	4.63951969	-1.57078636
6	-2.60338807	-1.11628032	3.84511375	1	2.93421197	5.57841969	-1.60898638
1	-3.03188825	-2.11838031	3.73621368	1	1.31381190	4.82141972	-1.47298646
1	-1.92118800	-1.14478028	4.70251369	1	2.57461190	4.04401970	-2.46528625
1	-3.41178823	-0.42118031	4.09561348	1	3.60811186	-2.67588043	2.38861370
6	-1.24828804	0.72061968	2.91301370	1	4.58951187	-3.55278039	1.17251360
1	-0.68558800	1.13281965	2.07471371	6	2.59981179	2.91971970	2.35341382
1	-0.58108807	0.67341965	3.78221369	1	3.07161188	3.90521955	2.29241371
1	-2.05988812	1.41961968	3.14371371	1	2.99321175	2.39081979	3.22491360
6	-0.69398808	-1.70768034	2.40471363	1	1.52221203	3.04351974	2.49661374

TS-12_LP

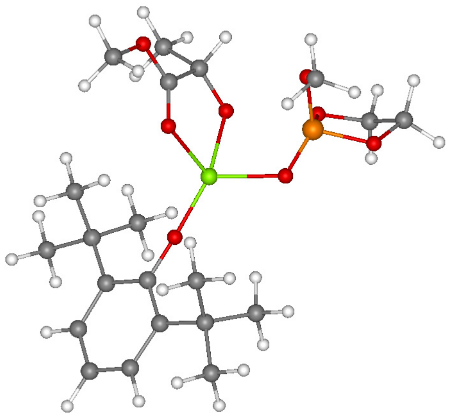

Zero-point vibrational energy	1446277.9 (Joules/Mol)
	345.66872 (Kcal/Mol)
Zero-point correction=	0.550858 (Hartree/Particle)
Thermal correction to Energy=	0.586395
Thermal correction to Enthalpy=	0.587339
Thermal correction to Gibbs Free Energy=	0.481887
Sum of electronic and zero-point Energies=	-1963.687541
Sum of electronic and thermal Energies=	-1963.652004
Sum of electronic and thermal Enthalpies=	-1963.651060
Sum of electronic and thermal Free Energies=	-1963.756513

cartesian

12	-0.70673352	0.03032728	0.13930909	1	2.65036654	3.06662726	0.20700909
8	1.09776652	-0.24557272	-0.09839091	1	1.44556653	3.53472733	-1.00699091
6	2.41486645	-0.41127270	-0.15279092	1	1.15846658	2.13982725	0.05820908
6	3.02256656	-1.54417276	0.47480911	8	-2.00903344	-1.50707269	0.06000908
6	4.41236639	-1.67817271	0.40400910	15	-3.36153340	-1.15287268	-0.50269091
1	4.89726639	-2.52767277	0.87340909	8	-4.10563374	-2.60807276	-0.65609092
6	5.21096659	-0.75187272	-0.25829092	8	-3.64223337	-0.65767276	-2.00409079
6	4.61056662	0.34052730	-0.87489092	6	-5.00673342	-2.57727265	-1.76489091
1	5.24746656	1.04992723	-1.39269102	6	-4.32603359	-1.66697276	-2.78129101
6	3.22726655	0.54142725	-0.84429091	1	-5.97683334	-2.17637277	-1.44919097
6	2.18276644	-2.61157274	1.20120907	1	-5.13433361	-3.59757280	-2.12979102
6	2.61286640	1.76782727	-1.54419100	1	-3.57973337	-2.20097280	-3.37529087
6	1.42326653	-1.99027276	2.39150906	1	-5.02633333	-1.15057278	-3.43869090
1	0.80046654	-1.14767277	2.08680916	8	-4.36733341	-0.35057271	0.45490912
1	0.78086650	-2.73887277	2.87140894	6	-4.56673336	-0.85707271	1.79040909
1	2.12836647	-1.61697268	3.14250898	1	-4.95673370	-1.87747276	1.75860906
6	1.20456648	-3.28147268	0.21490909	1	-3.63093352	-0.83457273	2.35420895
1	0.53636646	-2.56137276	-0.25709090	1	-5.29633331	-0.19387272	2.25450897
1	1.76076651	-3.79097271	-0.57969087	8	-1.11883354	1.37502730	1.72440898
1	0.58966649	-4.02927256	0.73090911	8	-2.24113345	0.97502732	-0.63049090
6	3.04896641	-3.74037266	1.78830910	6	-1.98013353	2.19532728	1.38000906
1	3.60356641	-4.28147268	1.01440907	6	-2.67923355	2.10712719	0.02410909
1	3.76456642	-3.37327266	2.53190899	1	-3.75693345	2.04622722	0.25460911
1	2.40026641	-4.46707249	2.29090905	1	6.28976631	-0.88247275	-0.29719090
6	3.67216635	2.63742733	-2.24439096	8	-2.35273361	3.19342732	2.16290903
1	4.40786648	3.04362726	-1.54199100	6	-1.67713344	3.30632734	3.43420911
1	4.20866632	2.08862734	-3.02569103	1	-2.10683346	4.18572760	3.91010904
1	3.17766643	3.48892736	-2.72559094	1	-0.60493350	3.43422723	3.27940893
6	1.61926651	1.32732725	-2.63869095	1	-1.85573351	2.41442728	4.03690910
1	0.81276655	0.71302724	-2.23689103	6	-2.43833351	3.38762736	-0.78919089
1	1.17416656	2.20152736	-3.13019085	1	-2.81463337	4.27692747	-0.27489090
1	2.13546658	0.73742729	-3.40389085	1	-2.95753360	3.28062725	-1.74419093
6	1.91896653	2.67812729	-0.51029092	1	-1.37223351	3.51552725	-0.99599087

I-2_LP

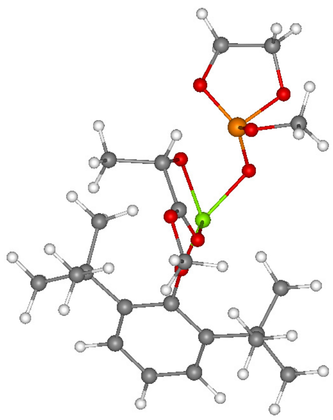

Zero-point vibrational energy	1448529.0 (Joules/Mol)
	346.20674 (Kcal/Mol)
Zero-point correction=	0.551716 (Hartree/Particle)
Thermal correction to Energy=	0.586692
Thermal correction to Enthalpy=	0.587636
Thermal correction to Gibbs Free Energy=	0.484447
Sum of electronic and zero-point Energies=	-1963.689561
Sum of electronic and thermal Energies=	-1963.654584
Sum of electronic and thermal Enthalpies=	-1963.653640
Sum of electronic and thermal Free Energies=	-1963.756829

cartesian

12	-0.62488794	0.24474543	-0.06886065	1	0.69031197	-1.27885461	1.93073928
8	1.19961214	0.12354544	0.07353935	1	0.88371199	-2.97345448	2.41683936
6	2.50961208	0.20324543	0.28933936	1	1.89111209	-1.70995462	3.14623952
6	3.21601200	1.41044545	-0.00616065	6	1.84991217	-2.82255435	-0.16306065
6	4.59261227	1.44964540	0.23583935	1	2.62521219	-3.05915451	-0.90026069
1	5.15251207	2.35414553	0.02293935	1	1.30371213	-3.74835443	0.05893935
6	5.28341198	0.35714546	0.74873936	1	1.16461205	-2.10845447	-0.62326068
6	4.58821201	-0.81495458	1.02643931	8	-1.89988804	1.36014545	0.93613935
1	5.14371204	-1.65795457	1.42373931	15	-3.25818801	0.64844543	0.92863935
6	3.21211219	-0.92845458	0.80813932	8	-4.12828827	1.85424542	1.71903932
6	2.49761200	2.65034556	-0.57186067	8	-3.77008796	-0.49355456	1.98953938
6	2.48751211	-2.25095439	1.12013936	6	-5.13668776	1.32604539	2.55813956
6	1.83781219	2.32144547	-1.92736065	6	-4.54458809	0.02544544	3.07833934
1	1.15801191	1.47054541	-1.86156070	1	-6.06248808	1.14004540	1.99333930
1	1.27931213	3.18814564	-2.30306053	1	-5.34488773	2.04434538	3.35453939
1	2.60211205	2.06704545	-2.67006063	1	-3.87888789	0.19494544	3.93023944
6	1.45411205	3.17514563	0.43553936	1	-5.29408789	-0.72455460	3.33813953
1	0.72011203	2.41944551	0.71753931	8	-4.10058784	0.68474543	-0.47026068

1	1.94661212	3.50124550	1.35783935	6	-4.83178806	1.85274541	-0.87556064
1	0.91451198	4.03504562	0.02033935	1	-5.72738791	1.99054539	-0.26666063
6	3.46391201	3.81864548	-0.83786064	1	-4.21048784	2.74834561	-0.81376064
1	3.95611191	4.16664553	0.07623935	1	-5.11908770	1.67194545	-1.91316068
1	4.23691225	3.55864549	-1.56876063	8	-1.15708804	-0.24995457	-2.06636047
1	2.90061212	4.66584539	-1.24556065	8	-2.33598804	-0.85575455	0.17253935
6	3.44411206	-3.33405447	1.64993930	6	-2.07678795	-1.07045460	-2.14966059
1	4.22831202	-3.58285451	0.92713934	6	-2.77258801	-1.62755454	-0.91396070
1	3.92241192	-3.04085445	2.59033942	1	-3.85058784	-1.52415454	-1.06846070
1	2.87731218	-4.25145483	1.84553933	1	6.35401201	0.41814545	0.92903936
6	1.42041206	-2.03855443	2.21353936	8	-2.50938797	-1.54415452	-3.30116057
6	-2.42688799	-3.10415435	-0.70846069	6	-1.83898807	-1.06645453	-4.49016094
1	-2.76218796	-3.70705438	-1.55686069	1	-2.32838798	-1.57575452	-5.31766081
1	-2.93088794	-3.44915438	0.19653934	1	-0.77898800	-1.31985462	-4.44906092
1	-1.35088801	-3.24245453	-0.57576066	1	-1.95628786	0.01464544	-4.57516098

TS-24_LP

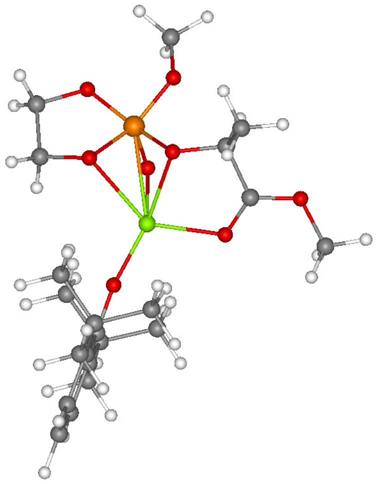

Zero-point vibrational energy	1449157.9 (Joules/Mol)
	346.35705 (Kcal/Mol)
Zero-point correction=	0.551955 (Hartree/Particle)
Thermal correction to Energy=	0.587005
Thermal correction to Enthalpy=	0.587949
Thermal correction to Gibbs Free Energy=	0.484436
Sum of electronic and zero-point Energies=	-1963.676642
Sum of electronic and thermal Energies=	-1963.641592
Sum of electronic and thermal Enthalpies=	-1963.640648
Sum of electronic and thermal Free Energies=	-1963.744162

cartesian

12	0.83857894	-0.06782274	-0.23654543	1	-2.04072094	3.29787731	0.61435461
8	-0.97402108	-0.17032273	0.04475456	1	-0.80892098	3.25557733	1.89015460
6	-2.30332088	-0.15072274	0.05855456	1	-0.70342100	2.15537739	0.49795458
6	-3.04412079	-0.97302276	-0.84744543	8	2.28797913	-1.37452281	-0.83894539
6	-4.44062090	-0.93432271	-0.79024541	15	3.13317919	-1.09572279	0.41665456
1	-5.02562094	-1.55042279	-1.46494544	8	3.65417910	-2.54152274	1.06595457
6	-5.12032080	-0.12392274	0.11255457	8	1.90497899	-1.01972282	1.63525462
6	-4.39132071	0.67667729	0.98535460	6	2.82157898	-3.14262271	2.04405451
1	-4.93772078	1.30397725	1.68155456	6	1.53177893	-2.32552266	2.06755447
6	-2.99322081	0.69037730	0.98675460	1	3.34387875	-3.10742259	3.00615454
6	-2.34172106	-1.88612282	-1.87054539	1	2.63817883	-4.18452311	1.77035463
6	-2.23342085	1.60087717	1.97025454	1	0.78407896	-2.74302268	1.38525462
6	-1.50302100	-1.04532278	-2.85514545	1	1.10227895	-2.24072266	3.06695437
1	-0.78912103	-0.39912274	-2.34174562	8	4.67447901	-0.66512275	0.08895457
1	-0.94702101	-1.69412279	-3.54254556	6	5.69647932	-1.61572278	-0.24534543
1	-2.15372086	-0.39642274	-3.45144558	1	6.04097891	-2.13902259	0.64745456
6	-1.45992100	-2.92612267	-1.15034544	1	5.33757925	-2.33942270	-0.98064542
1	-0.70782101	-2.45162272	-0.51904541	1	6.51207924	-1.02982271	-0.67464542
1	-2.07592106	-3.56782269	-0.51084542	8	1.24857903	1.56777716	-1.45814538
1	-0.94302106	-3.56472278	-1.87674546	8	2.77507877	0.67527729	0.51145458
6	-3.33962107	-2.68432260	-2.72844553	6	2.36247897	2.08487725	-1.34884536
1	-3.96522093	-3.34922266	-2.12374544	6	3.38447905	1.64847720	-0.31154543
1	-3.99572086	-2.03372264	-3.31594563	1	4.22427893	1.22077715	-0.86804539
1	-2.78492117	-3.31212258	-3.43514562	1	-6.20742083	-0.11562274	0.13525458
6	-3.18152094	2.40477729	2.87755442	8	2.77277899	3.05917740	-2.13664556
1	-3.83892107	3.06997728	2.30745435	6	1.84687901	3.49517727	-3.15974545
1	-3.80502081	1.75677717	3.50235438	1	2.36527896	4.29017687	-3.69134545
1	-2.58792114	3.03357744	3.55095458	1	0.93087900	3.86677742	-2.69944549
6	-1.33912098	0.76317728	2.90725446	1	1.61447895	2.66607738	-3.82904553
1	-0.61232102	0.16707727	2.35485458	6	3.88157892	2.81337738	0.54115456
1	-0.79402101	1.41537726	3.60115457	1	4.33857918	3.58867741	-0.07804543
1	-1.95262110	0.07767726	3.50245452	1	4.62647915	2.43177724	1.24185455
6	-1.39062107	2.63527727	1.19635463	1	3.05897903	3.24857736	1.11435461

I-4_LP

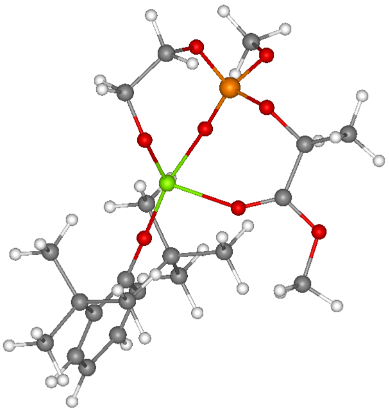

Zero-point vibrational energy	1446312.2 (Joules/Mol)
	345.67691 (Kcal/Mol)
Zero-point correction=	0.550871 (Hartree/Particle)
Thermal correction to Energy=	0.587327
Thermal correction to Enthalpy=	0.588272
Thermal correction to Gibbs Free Energy=	0.480780
Sum of electronic and zero-point Energies=	-1963.692808
Sum of electronic and thermal Energies=	-1963.656352
Sum of electronic and thermal Enthalpies=	-1963.655408
Sum of electronic and thermal Free Energies=	-1963.762900

cartesian

12	0.71300149	0.75139695	0.51992869	1	-2.51169872	2.50359702	-2.00287127
8	-1.02909851	0.12829691	0.40542874	1	-1.63069868	3.69579697	-1.02777123
6	-2.31559873	-0.17360309	0.28792873	1	-1.09799862	2.00509691	-1.07597125
6	-2.72869849	-1.54450309	0.30412874	8	2.14990139	-0.65170306	0.97862870
6	-4.08999872	-1.83450317	0.17292874	15	3.57850146	-0.19860309	0.89762872
1	-4.42659855	-2.86590290	0.18382874	8	4.13530159	0.79189694	1.97312880
6	-5.04669857	-0.83530307	0.02982873	8	4.56360149	-1.44590306	0.98402870
6	-4.63859844	0.49419692	0.01902873	6	4.29660130	-2.51380301	1.91982877
1	-5.39909840	1.26039684	-0.08867127	1	4.56390142	-2.19370294	2.92922878
6	-3.29509854	0.86099696	0.14432873	1	3.24510145	-2.80330300	1.87712872
6	-1.71259856	-2.68950295	0.47142875	1	4.93130159	-3.34480309	1.61482882
6	-2.90299869	2.35039711	0.13422874	8	1.39070153	0.67899692	-1.49437118
6	-0.69879860	-2.68310308	-0.69107127	8	3.97350144	0.46999690	-0.50337130
1	-0.17159855	-1.73020315	-0.75097132	6	2.26180148	0.20739692	-2.20787120
1	0.04160145	-3.48290300	-0.56107128	6	3.67590141	-0.15970308	-1.75317121
1	-1.21429849	-2.85210299	-1.64387119	1	3.68570137	-1.25120306	-1.63597119
6	-0.98469859	-2.56480289	1.82532883	1	-6.09939861	-1.08970308	-0.06757127
1	-0.46159858	-1.61290312	1.91682875	8	2.06790137	-0.08440308	-3.48277116

1	-1.70309854	-2.63630295	2.64932871	6	0.72980142	0.14359692	-3.99397135
1	-0.25279856	-3.37410307	1.94382882	1	0.76250148	-0.19240308	-5.02797127
6	-2.38119864	-4.07560301	0.45732874	1	0.48930144	1.20539689	-3.93597126
1	-3.09329867	-4.20130301	1.27972877	1	0.00720146	-0.43220308	-3.41567135
1	-2.90449858	-4.27470303	-0.48427126	6	4.75500154	0.25439692	-2.74297118
1	-1.61209869	-4.84810305	0.57242870	1	4.58790159	-0.21800309	-3.71157122
6	-4.12189865	3.27759695	-0.02617126	1	5.72670126	-0.05710308	-2.35547113
1	-4.65949869	3.10169697	-0.96417135	1	4.76090145	1.33929694	-2.87097120
1	-4.83189869	3.17699695	0.80162871	6	3.87580132	2.23819709	2.07672882
1	-3.78119850	4.31919670	-0.03677126	1	4.50670147	2.54639697	2.91342878
6	-2.23599863	2.74429703	1.46762872	6	2.40840149	2.55409694	2.30502868
1	-1.33679843	2.16339707	1.67452872	1	4.23210144	2.69359708	1.14962876
1	-1.95499849	3.80419707	1.45802879	1	2.37730145	3.64189696	2.51302886
1	-2.93229866	2.58549690	2.29832888	1	2.09560156	2.05719709	3.24362874
6	-1.97429872	2.65479708	-1.05947125	8	1.64220142	2.21579695	1.21892881

I-5_LPL

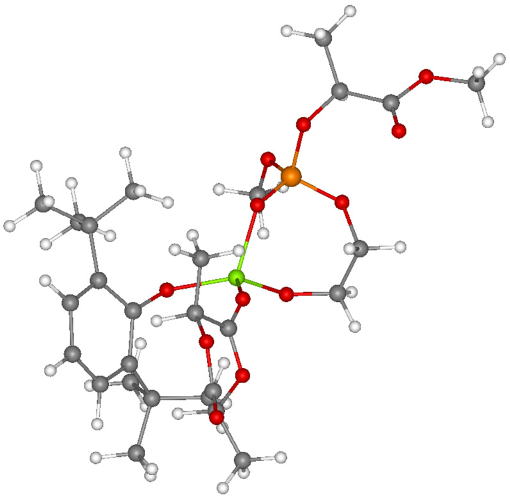

Zero-point vibrational energy	1819608.6 (Joules/Mol)
	434.89689 (Kcal/Mol)
Zero-point correction=	0.693052 (Hartree/Particle)
Thermal correction to Energy=	0.740863
Thermal correction to Enthalpy=	0.741807
Thermal correction to Gibbs Free Energy=	0.606007
Sum of electronic and zero-point Energies=	-2497.786012
Sum of electronic and thermal Energies=	-2497.738201
Sum of electronic and thermal Enthalpies=	-2497.737257
Sum of electronic and thermal Free Energies=	-2497.873057

cartesian

12	-0.38059509	-0.04624899	-0.77887499	15	-3.47739506	0.31735098	-0.19807494

8	-4.46909523	0.20575102	1.03842509	8	-3.94239497	-0.71954900	-1.29817498
8	0.92520493	1.34535110	-0.63227499	8	-1.27389514	-0.49164897	-2.38177490
6	-5.89009523	0.13515101	0.86922503	6	-3.14139509	-1.87634897	-1.70187497
6	-6.31179523	-1.32874894	0.73762500	6	-2.15109515	-1.47244895	-2.78747487
1	-6.19279480	0.66385102	-0.04057495	1	-3.87109494	-2.60894895	-2.05487490
6	2.13340497	1.35105109	-0.08937495	1	-2.63619494	-2.27604914	-0.81777495
6	3.25820494	0.83225101	-0.81777495	1	-1.64289510	-2.40914917	-3.09167480
6	4.45530510	0.62455100	-0.11987495	1	-2.73509502	-1.14544892	-3.66757488
1	5.31770515	0.21065103	-0.63437498	8	-3.86919498	1.72975111	-0.80567497
6	4.60240507	0.98455101	1.21952498	1	5.54120493	0.80755097	1.73902500
6	3.56880498	1.68275106	1.84372509	6	-3.34389496	2.16655087	-2.08847475
1	3.74100494	2.06315088	2.84582520	1	-2.91479492	3.15625095	-1.93057501
6	2.34640503	1.92605102	1.20842505	1	-4.18169498	2.22285104	-2.78497481
6	3.21000504	0.65965098	-2.35247493	1	-2.58389497	1.47665107	-2.46657491
6	1.31230485	2.87605095	1.84752500	8	4.58250475	-4.49004889	1.80872500
6	2.81040502	2.01305103	-2.97867489	8	3.00560498	-3.03674912	2.40532517
1	1.82890475	2.33445096	-2.62477493	6	3.82330513	-3.60694909	1.49732506
1	2.77100492	1.92675102	-4.07147503	6	3.70070505	-3.05624914	0.08422505
1	3.54180503	2.78825092	-2.72587490	8	2.30980492	-2.74064898	-0.21677494
6	2.22650504	-0.42344898	-2.83607483	6	1.64060485	-2.01134896	0.66232502
1	1.18190479	-0.12424899	-2.73897481	6	2.25790501	-1.85714889	2.03652501
1	2.37140489	-1.37154889	-2.30947495	1	2.94780493	-1.00024903	1.97522497
1	2.38480496	-0.61674899	-3.90427494	8	0.56250489	-1.51774895	0.35522506
6	4.59200478	0.29155099	-2.92167497	1	4.25340509	-2.10884905	0.03522505
1	4.93580484	-0.69534898	-2.58867478	6	1.22760487	-1.62824893	3.12262511
1	5.35870504	1.02785110	-2.65997481	1	1.73910487	-1.48114896	4.07552481
1	4.53260517	0.25705099	-4.01467514	1	0.55940491	-2.48834896	3.20612526
6	1.88500488	3.59015083	3.08502507	1	0.63770491	-0.74264902	2.89282513
1	2.80270505	4.14205122	2.85602522	6	4.18860483	-4.02114916	-0.97147501
1	2.09480500	2.89875102	3.90892506	1	3.62520504	-4.95574903	-0.93237495
1	1.14940488	4.31285095	3.45502520	1	5.24100494	-4.24664879	-0.79387498
6	0.03560488	2.14925098	2.30322504	1	4.07890511	-3.57434916	-1.96137488
1	-0.47299510	1.64625108	1.48182499	8	-7.60819483	-1.39174891	0.38142505
1	-0.67189509	2.86685085	2.73702526	8	-5.60619497	-2.28874898	0.94082505
1	0.26340491	1.41145110	3.08002520	6	-8.15029526	-2.71894908	0.26262504
6	0.94250494	3.97425103	0.82772505	1	-7.61779499	0.75935102	2.00202513
1	1.83000481	4.54935122	0.54152507	1	-6.20909500	1.83655107	2.14082503
1	0.21910490	4.66985130	1.27042508	1	-6.22119522	0.28305101	2.99922514
1	0.50520492	3.54605103	-0.07537495	1	-9.19579506	-2.58324909	-0.00947495
6	-6.52969503	0.79415101	2.08402514	1	-8.06529522	-3.25114918	1.21212506
8	-2.07839513	0.17345102	0.31272504	1	-7.62109518	-3.27634907	-0.51267499

I-5_LPP

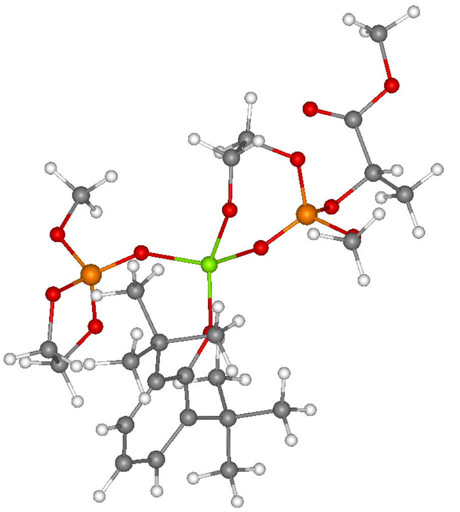

Zero-point vibrational energy	1752800.6 (Joules/Mol)
	418.92940 (Kcal/Mol)
Zero-point correction=	0.667607 (Hartree/Particle)
Thermal correction to Energy=	0.714352
Thermal correction to Enthalpy=	0.715296
Thermal correction to Gibbs Free Energy=	0.582249
Sum of electronic and zero-point Energies=	-2724.269713
Sum of electronic and thermal Energies=	-2724.222968
Sum of electronic and thermal Enthalpies=	-2724.222024
Sum of electronic and thermal Free Energies=	-2724.355071

cartesian

12	-0.10930753	0.29227048	0.66469258	8	-1.78880751	0.02857049	-0.46290740
8	-4.15530729	-0.36512953	-1.16370738	15	-3.09450769	-0.52312952	0.01269260
8	1.32659256	-0.86812955	0.25119260	8	-3.71900749	0.14057049	1.30849266
6	-5.53690767	-0.69302952	-0.97950739	8	-1.02300751	0.14437050	2.32219243
6	-6.29440784	0.53647053	-0.47760740	6	-3.12460756	1.29287052	1.98389268
1	-5.64350748	-1.49282956	-0.23990740	6	-2.03580761	0.83327049	2.94659257
6	2.59979248	-1.00332952	-0.07610740	1	-3.96050763	1.77567053	2.49639249
6	3.62199235	-0.95482951	0.93169260	1	-2.72950768	1.97357059	1.22399271
6	4.96019220	-0.94682950	0.52029258	1	-1.68640745	1.75077057	3.46239257
1	5.75129223	-0.88052946	1.26009262	1	-2.51970768	0.21507049	3.72609258
6	5.32409239	-1.02952957	-0.82150739	8	-3.15990758	-2.08272958	0.29409260
6	4.32829237	-1.21422958	-1.77860737	1	6.37239218	-1.01332951	-1.11110735
1	4.63259220	-1.36312962	-2.81000757	6	-2.52140760	-2.67762947	1.45709264
6	2.97059250	-1.24342942	-1.44330740	1	-1.88410747	-1.95552945	1.97529268
6	3.28319240	-0.96302956	2.43689251	1	-1.92910755	-3.51642966	1.09129262
6	1.91989243	-1.60972953	-2.51200747	1	-3.31220770	-3.03162956	2.12059259

6	2.37469244	-2.16692948	2.76249242	8	0.44119245	2.19967055	0.17499259
1	1.44589245	-2.13062954	2.19259262	15	1.52119255	3.08787036	-0.34050742
1	2.12419248	-2.17292953	3.83019257	8	2.90399241	3.16097045	0.44449261
1	2.88789248	-3.10652947	2.52889252	8	2.07209229	2.65547037	-1.77950740
6	2.60039234	0.34377050	2.88719249	6	3.96169233	2.55307055	-0.35040739
1	1.58529246	0.44257051	2.50129247	6	3.51849246	2.71247053	-1.80580735
1	3.18079233	1.22107053	2.58259249	8	1.05399251	4.60177040	-0.38190740
1	2.51289248	0.36457050	3.98029256	6	-0.33560753	4.94667053	-0.57910740
6	4.54529238	-1.11542940	3.30589247	1	-0.97020757	4.35827065	0.08449259
1	5.21609259	-0.25192952	3.23009253	1	-0.61740756	4.77727032	-1.62050736
1	5.11419249	-2.01722956	3.05589247	1	-0.41340753	6.00577021	-0.33880740
1	4.24669218	-1.19752955	4.35669279	1	3.86819220	1.89007044	-2.42870736
6	2.57359242	-2.02392960	-3.84260750	1	3.81469250	3.67487049	-2.23290753
1	3.25929236	-2.86922956	-3.72130752	1	4.88129234	3.09327054	-0.12620740
1	3.12119246	-1.20102954	-4.31620741	1	4.05329227	1.50457048	-0.06110740
1	1.79159248	-2.33352947	-4.54500723	8	-7.50040770	0.15897049	-0.01200740
6	0.97529250	-0.43722951	-2.83200741	8	-5.89970779	1.67817044	-0.51780742
1	0.39379245	-0.13012952	-1.96400738	6	-8.34770679	1.22787046	0.44239259
1	0.26339245	-0.72962952	-3.61360741	1	-8.54700756	1.92897058	-0.37070739
1	1.53239250	0.43227050	-3.19470739	1	-7.13870764	-1.41612959	-2.23370743
6	1.09939253	-2.82282948	-2.02480745	1	-5.52690744	-2.04132962	-2.65130734
1	1.75169253	-3.68982959	-1.87210739	1	-5.96350765	-0.37292951	-3.07310748
1	0.34499246	-3.09392953	-2.77350736	1	-9.26930714	0.74997050	0.77099258
1	0.59239244	-2.60562968	-1.08390737	1	-7.87540770	1.76007056	1.27039266
6	-6.08100748	-1.15922952	-2.32360744				

S1.9. PL sequence, mononuclear mechanism

I-1_PL

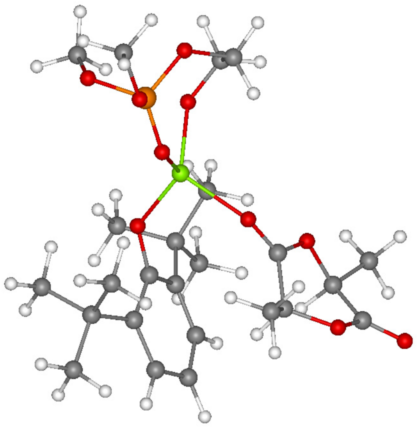

Zero-point vibrational energy	1632022.7 (Joules/Mol)
	390.06278 (Kcal/Mol)
Zero-point correction=	0.621605 (Hartree/Particle)
Thermal correction to Energy=	0.663734
Thermal correction to Enthalpy=	0.664678
Thermal correction to Gibbs Free Energy=	0.542884
Sum of electronic and zero-point Energies=	-2230.735845
Sum of electronic and thermal Energies=	-2230.693716
Sum of electronic and thermal Enthalpies=	-2230.692772
Sum of electronic and thermal Free Energies=	-2230.814566

cartesian

12	-1.26337743	-0.54803741	-0.29808521	1	-1.43327737	4.52216244	0.60421479
8	-5.12847710	-0.56613743	1.95051479	1	-1.10137737	3.19166255	-0.52328521
8	-0.28057736	1.04016256	-0.71098518	1	-7.04097700	-0.04573746	1.30131471
6	-6.55107737	-0.78373748	1.94021475	8	-2.84437752	-0.40503746	0.96341479
1	-6.87957716	-0.65943742	2.97131491	15	-4.27257729	-0.70243746	0.62271482
1	-6.77987719	-1.79603744	1.59791470	8	-4.55957699	-2.14813757	0.03341480
6	0.95962268	1.38916254	-0.40208519	8	-2.20627737	-1.58643746	-1.56998527
6	2.06232262	0.92876250	-1.20018530	6	-3.51177740	-3.13013744	-0.24018522
6	3.35832262	1.11106253	-0.70028520	6	-2.81097746	-2.82373738	-1.55808520
1	4.21302271	0.75226253	-1.26638520	1	-4.03457737	-4.08973742	-0.26458520
6	3.60192251	1.79286265	0.49191481	1	-2.80737734	-3.12593746	0.59801477
6	2.53442264	2.41506267	1.13911474	1	-2.09727740	-3.65583730	-1.71898520
1	2.75212264	3.04196262	1.99811471	1	-3.56237745	-2.90903735	-2.36468506
6	1.21412253	2.27596259	0.69771481	8	-5.00347710	0.30826253	-0.35568517
6	1.84662247	0.40136254	-2.63648510	1	4.61802292	1.91736269	0.85891479

6	0.09262264	3.13926268	1.31331480	6	-4.86827707	0.25286257	-1.80018520
6	1.09442258	1.48466253	-3.43948507	1	-4.94227743	1.28356266	-2.14618516
1	0.12612262	1.70496249	-2.98578525	1	-5.69447708	-0.33933747	-2.19878507
1	0.92392260	1.14236259	-4.46748495	1	-3.91007733	-0.18673745	-2.08918524
1	1.67642260	2.41166258	-3.48298502	8	4.80602264	-3.39023733	2.10171485
6	1.04932261	-0.91463745	-2.71448517	8	3.10912251	-2.09193754	2.72631478
1	-0.00777738	-0.78483742	-2.47928524	6	3.85492253	-2.71733737	1.79271472
1	1.46322274	-1.68383741	-2.05538511	6	3.39722252	-2.50783730	0.35661477
1	1.08542252	-1.30933738	-3.73758507	8	1.94102263	-2.47923732	0.28991479
6	3.18502259	0.15276253	-3.35478520	6	1.30042267	-1.69393754	1.14101481
1	3.76592255	-0.65493745	-2.89298511	6	2.10262251	-1.14613748	2.30271482
1	3.81252265	1.04906249	-3.39128518	1	2.59902263	-0.23313746	1.93621469
1	2.98632264	-0.14743745	-4.38908529	8	0.10802263	-1.45973754	0.98321480
6	0.65692264	4.21156263	2.26291490	1	3.74542260	-1.52093744	0.02451479
1	1.39102268	4.85566282	1.76751471	6	1.25062251	-0.83183748	3.51391482
1	1.12352252	3.78056240	3.15591478	1	1.88172245	-0.40303746	4.29441500
1	-0.16247737	4.85116243	2.60901475	1	0.77692264	-1.73843741	3.89721489
6	-0.92047739	2.31376266	2.12391472	1	0.47732264	-0.11443746	3.24461484
1	-1.41877747	1.55676246	1.52021480	6	3.87652278	-3.59213734	-0.58058518
1	-1.69907737	2.96966267	2.53261495	1	3.51042247	-4.57013750	-0.26148522
1	-0.43487737	1.82186270	2.97351480	1	4.96712303	-3.61523747	-0.57528520
6	-0.64477736	3.88756275	0.18211479	1	3.52662253	-3.38823748	-1.59408522
1	0.04822263	4.53286266	-0.36858517				

TS-12_PL

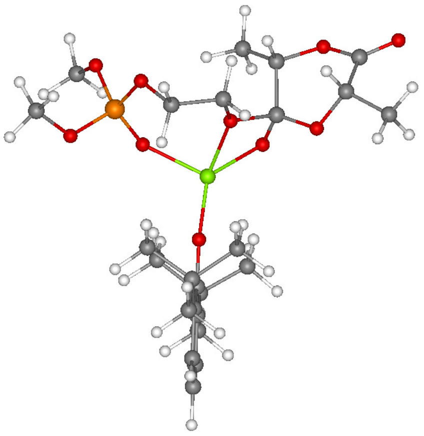

Zero-point vibrational energy	1632019.7 (Joules/Mol)
	390.06208 (Kcal/Mol)
Zero-point correction=	0.621603 (Hartree/Particle)
Thermal correction to Energy=	0.662683
Thermal correction to Enthalpy=	0.663628
Thermal correction to Gibbs Free Energy=	0.544119

Sum of electronic and zero-point Energies= -2230.720704
Sum of electronic and thermal Energies= -2230.679624
Sum of electronic and thermal Enthalpies= -2230.678680
Sum of electronic and thermal Free Energies= -2230.798188

cartesian

12	-0.40561718	-0.00560797	-0.63097334	1	1.02428281	-2.10510802	-3.70697355
8	-0.09081718	4.11239243	0.26242664	1	0.76898289	-1.97390795	-1.95967329
8	1.35308290	-0.36250797	-0.20157336	1	-0.99801719	5.99639225	0.34502664
6	-0.39531720	5.33229208	0.96902668	8	-1.80571711	-1.21480799	-1.32327330
1	0.56468284	5.79599237	1.18922663	6	-2.70621705	-1.20050800	-0.40907335
1	-0.92481720	5.11379242	1.89902663	8	-2.72811723	-2.32170796	0.39182663
6	2.61538291	-0.76590800	-0.10667336	6	-3.98171711	-2.67600799	0.99012661
6	3.25468278	-0.78610796	1.17252672	6	-5.04161739	-2.79500794	-0.10327336
6	4.57938242	-1.22510803	1.25092661	1	-4.30371714	-1.88550794	1.68602669
1	5.08388281	-1.25590801	2.21102643	8	-5.87331724	-3.66770792	-0.16307336
6	5.28838253	-1.62930799	0.12492663	6	-4.12221718	-0.69810796	-0.75767332
6	4.66458273	-1.59130800	-1.11737335	8	-4.97681761	-1.82810795	-1.04587340
1	5.23578262	-1.90410793	-1.98507333	8	-0.60831720	1.99429202	-0.94967335
6	3.34088278	-1.16810799	-1.27187335	15	-1.24611723	3.15199208	-0.25747335
6	2.52048278	-0.31830797	2.44222665	8	-2.22121716	2.85379195	0.95652664
6	2.69628286	-1.15110803	-2.67007351	8	-2.06901717	0.04799203	0.56462663
6	1.27508283	-1.18880796	2.70782661	6	-1.87241721	1.91709197	2.01582646
1	0.57928276	-1.17390800	1.86802661	6	-2.54201722	0.57689202	1.77292669
1	0.75038278	-0.84030795	3.60772657	1	-2.23671722	2.37129211	2.94032645
1	1.56608284	-2.23130798	2.87532663	1	-0.78631723	1.80639207	2.08272648
6	2.12498283	1.16529202	2.29692650	1	-2.31221724	-0.07600798	2.62862659
1	1.51998281	1.32929206	1.40292668	1	-3.62971711	0.73159200	1.74152672
1	3.01858282	1.79269207	2.20702648	8	-2.22781730	4.00499201	-1.16527331
1	1.56808281	1.50349200	3.18202662	1	6.31798267	-1.96730804	0.21412665
6	3.39878273	-0.41840798	3.70152664	1	-4.55221748	-0.17590797	0.10402664
1	4.29838276	0.20219202	3.63122654	6	-1.84871721	4.29589224	-2.53187346
1	3.70678306	-1.44830799	3.90992665	1	-0.98501718	4.96449232	-2.55247355
1	2.82828283	-0.06720798	4.56932640	1	-2.71151710	4.78739214	-2.97797346
6	3.67828298	-1.57900798	-3.77617335	1	-1.62181711	3.37179208	-3.06567335
1	4.03128242	-2.60680795	-3.64277339	6	-4.14611721	0.20329203	-1.96947336
1	4.55068254	-0.91940796	-3.83567357	1	-5.17521763	0.49749202	-2.19007349
1	3.16888285	-1.53540802	-4.74577332	1	-3.72111726	-0.31390798	-2.83097339
6	2.23778296	0.27589202	-3.03777337	1	-3.55731726	1.10129201	-1.77197337
1	1.58608282	0.71499205	-2.28017354	6	-3.78511715	-3.96980786	1.75112665
1	1.71078277	0.27579203	-4.00017357	1	-3.46431708	-4.76270771	1.07302666

1	3.10468292	0.93959200	-3.13017344	1	-4.72301722	-4.27310753	2.21922660
6	1.52128279	-2.14860797	-2.73017335	1	-3.02311707	-3.83120799	2.52142644
1	1.88738275	-3.17070794	-2.58597350				

I-2_PL

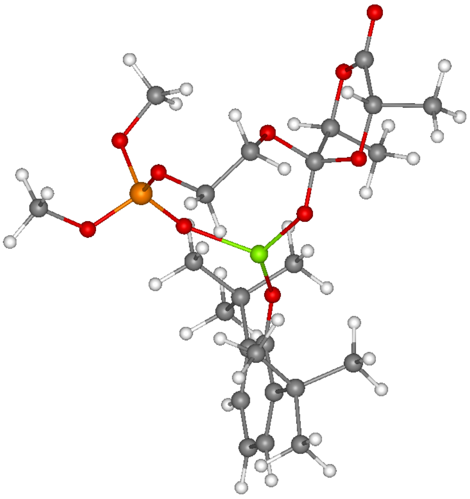

Zero-point vibrational energy	1638080.9 (Joules/Mol)
	391.51073 (Kcal/Mol)
Zero-point correction=	0.623912 (Hartree/Particle)
Thermal correction to Energy=	0.664837
Thermal correction to Enthalpy=	0.665781
Thermal correction to Gibbs Free Energy=	0.547768
Sum of electronic and zero-point Energies=	-2230.720964
Sum of electronic and thermal Energies=	-2230.680039
Sum of electronic and thermal Enthalpies=	-2230.679095
Sum of electronic and thermal Free Energies=	-2230.797108

cartesian

12	0.03015167	0.08813065	0.30872402	1	0.31965166	-0.37136933	2.52082396
8	-0.53154832	4.41483021	0.79322404	1	1.52375162	0.85953069	1.91722405
8	1.67525172	-0.50216937	-0.29387599	1	-1.34684825	6.14133024	-0.04567598
6	-1.13104832	5.72053051	0.93772405	8	-0.43354833	2.00703073	0.11432403
1	-0.39014834	6.32993031	1.45252407	15	-1.32904840	3.20213079	0.17362402
1	-2.04284835	5.65653038	1.53582406	8	-2.65054822	2.98503065	1.02262402
6	2.98025155	-0.50676930	-0.01267598	8	-3.51894832	0.39583066	0.10442402
6	3.93055177	-0.26626936	-1.04937601	6	-2.83464837	1.87273073	1.94182408
6	5.28635120	-0.25946936	-0.70957595	6	-3.85694838	0.90273070	1.38282406
1	6.03265190	-0.07526934	-1.47497594	1	-3.19824839	2.31093073	2.87512398
6	5.72545147	-0.48356935	0.59242404	1	-1.88114834	1.37763071	2.12492394
6	4.79245186	-0.74026930	1.59102404	1	-3.97954822	0.09283065	2.11242390

1	5.15265179	-0.92676938	2.59772396	1	-4.81754875	1.41693068	1.27062404
6	3.42145157	-0.76586938	1.31932402	8	-1.83554828	3.77253079	-1.20757592
6	3.48625159	-0.02966934	-2.50387597	1	6.78765202	-0.46706936	0.82392406
6	2.40775156	-1.11846936	2.42112398	6	-2.64794827	2.98203063	-2.11437607
6	2.63345170	1.25093067	-2.60547614	1	-3.34274840	2.34563065	-1.56457591
1	1.73815179	1.18673062	-1.98527598	1	-1.98794830	2.37903070	-2.73967600
1	2.32335162	1.42013073	-3.64427614	1	-3.19144821	3.69853067	-2.72817612
1	3.20805168	2.12503052	-2.27947617	8	-6.24664879	-1.88366938	-1.60197592
6	2.68925166	-1.24426937	-3.02187610	8	-4.10284805	-1.43916929	-1.92197597
1	1.80745173	-1.43356931	-2.40847611	6	-5.13544846	-1.77676928	-1.13047600
1	3.31235170	-2.14516926	-3.00757599	6	-4.90524864	-1.99956942	0.36352402
1	2.36765170	-1.07486928	-4.05717564	8	-3.56744838	-1.83126926	0.82562405
6	4.68225193	0.15723065	-3.45397615	6	-2.80334830	-0.87516928	0.10622402
1	5.33915138	-0.71906936	-3.47077608	6	-2.77324843	-1.29756927	-1.37337601
1	5.28555202	1.03523064	-3.19807601	1	-2.33734846	-0.47796932	-1.95377600
1	4.31195164	0.30513066	-4.47467566	8	-1.59694839	-0.79346931	0.65202403
6	3.07845163	-1.31236935	3.79232383	1	-5.55514812	-1.25586927	0.84642404
1	3.60145164	-0.41056934	4.12862396	6	-5.36734867	-3.39146924	0.77692401
1	3.79305148	-2.14066935	3.78182387	1	-6.41274834	-3.53566933	0.49942401
1	2.31475163	-1.55316937	4.54062414	1	-5.26174831	-3.50016928	1.85922408
6	1.71765161	-2.45666933	2.07502389	1	-4.76004791	-4.16096973	0.29312402
1	1.22045171	-2.42356920	1.10332406	6	-1.99244833	-2.57936931	-1.60717595
1	0.97345161	-2.72066927	2.83602381	1	-2.38924837	-3.39326930	-0.99547595
1	2.46195173	-3.25816941	2.03672385	1	-0.93864834	-2.44786930	-1.35227597
6	1.34955168	0.01643066	2.59812403	1	-2.06264830	-2.86096931	-2.66087604
1	1.39515162	0.45683065	3.59942389				

TS-23_PL

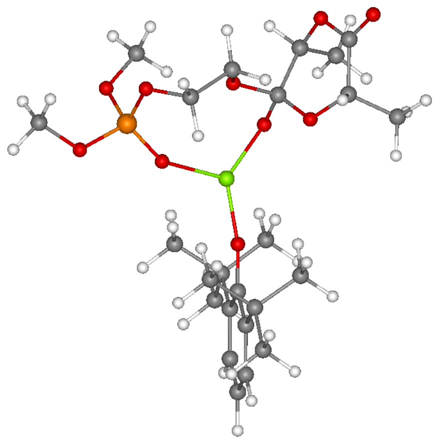

Zero-point vibrational energy 1635302.2 (Joules/Mol)

390.84660 (Kcal/Mol)

Zero-point correction= 0.622854 (Hartree/Particle)

Thermal correction to Energy= 0.663460
Thermal correction to Enthalpy= 0.664404
Thermal correction to Gibbs Free Energy= 0.547386
Sum of electronic and zero-point Energies= -2230.713051
Sum of electronic and thermal Energies= -2230.672444
Sum of electronic and thermal Enthalpies= -2230.671500
Sum of electronic and thermal Free Energies= -2230.788518

cartesian

12	-0.15403602	0.30497861	-0.49860129	1	1.25136399	0.90477860	2.04599881
8	-0.92023599	4.48237848	0.92329872	1	2.60346389	1.42187858	3.05299878
8	1.54946399	-0.18152142	-0.03280130	1	-1.97023606	6.16457844	0.27729869
6	-1.64513600	5.69677830	1.20839870	8	-0.60823601	2.15157843	0.08619870
1	-0.94193602	6.34517860	1.72809863	15	-1.62033606	3.23737860	0.25349870
1	-2.50313616	5.48617840	1.85089862	8	-2.88543606	2.86427879	1.14459872
6	2.86996388	-0.31912142	0.04369871	8	-2.97833610	0.42727858	-0.07650129
6	3.67596412	-0.21222141	-1.13140130	6	-2.97843599	1.65297854	1.93999863
6	5.06026411	-0.36272138	-1.00350130	6	-3.66723609	0.55597860	1.14869869
1	5.69566441	-0.28682142	-1.87960136	1	-3.55603600	1.92607856	2.82579875
6	5.66236401	-0.61232144	0.22489870	1	-1.97663605	1.34267855	2.24709868
6	4.86746407	-0.71932143	1.36179864	1	-3.62413597	-0.35362142	1.75619864
1	5.35526419	-0.91702139	2.31049871	1	-4.72093582	0.80647856	0.97179872
6	3.47766399	-0.58052140	1.30969870	8	-2.23143601	3.84777880	-1.07150137
6	3.04976392	0.05677859	-2.51180124	1	6.74156427	-0.72452140	0.29579869
6	2.63466406	-0.71072143	2.59189868	6	-3.07773614	3.07927847	-1.95990133
6	2.34836388	1.43207860	-2.52600121	1	-4.00013590	2.80497837	-1.44670129
1	1.66866398	1.57047856	-1.68070138	1	-2.55763602	2.18257856	-2.29870129
1	1.78866386	1.57727861	-3.45850110	1	-3.28963614	3.73827839	-2.80050111
1	3.08726406	2.23677874	-2.44960117	8	-5.56173563	-3.15702152	0.92699873
6	2.07186389	-1.07522142	-2.89290118	8	-4.89823580	-1.81002140	-0.70580125
1	1.29156399	-1.23642147	-2.14710116	6	-4.65213585	-2.59902143	0.35509869
1	2.61446404	-2.02152157	-2.99010110	6	-3.22213602	-2.72092152	0.87129873
1	1.59186399	-0.86342144	-3.85630131	8	-2.27963591	-1.77292144	0.35819870
6	4.10016441	0.10657859	-3.63620114	6	-2.61603594	-0.86902142	-0.69120127
1	4.63726425	-0.84182143	-3.73920131	6	-3.79533601	-1.36892140	-1.53460133
1	4.83506441	0.90407860	-3.48470116	1	-4.19653606	-0.49632144	-2.05260110
1	3.59886384	0.30267859	-4.59110165	8	-1.55143607	-0.61162144	-1.43220139
6	3.49796391	-1.00652146	3.83109879	1	-3.31523609	-2.52632141	1.94669867
1	4.21816397	-0.20752141	4.03709841	6	-2.68083596	-4.14082146	0.71629870
1	4.04716396	-1.94922149	3.73669887	1	-3.35113597	-4.84682131	1.21179867
1	2.84926391	-1.09482145	4.71009874	1	-1.69463611	-4.19522142	1.18279862

6	1.63556397	-1.87892139	2.46209884	1	-2.57853603	-4.42552137	-0.33260131
1	0.97256398	-1.75292146	1.60519862	6	-3.42183614	-2.42952156	-2.55460119
1	1.02446389	-1.96062148	3.37019873	1	-2.96713614	-3.30722141	-2.09150124
1	2.17146397	-2.82572126	2.33309889	1	-2.69883609	-2.01272154	-3.25730133
6	1.88966393	0.60907859	2.87999868	1	-4.31663609	-2.74422121	-3.09830117
1	1.26986396	0.50917858	3.78069878				

I-3_PL

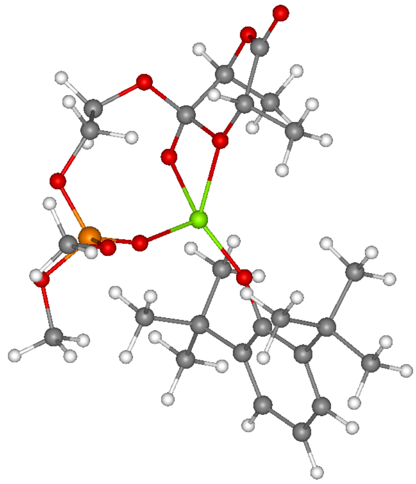

Zero-point vibrational energy	1636850.4 (Joules/Mol)
	391.21664 (Kcal/Mol)
Zero-point correction	XXX (Hartree/Particle)
Zero-point correction=	0.623443 (Hartree/Particle)
Thermal correction to Energy=	0.664748
Thermal correction to Enthalpy=	0.665692
Thermal correction to Gibbs Free Energy=	0.547195
Sum of electronic and zero-point Energies=	-2230.728551
Sum of electronic and thermal Energies=	-2230.687246
Sum of electronic and thermal Enthalpies=	-2230.686302
Sum of electronic and thermal Free Energies=	-2230.804799

cartesian

8	-1.18535161	-0.49704006	0.11351198	8	2.33434844	-1.17964005	-0.12448803
6	-2.50895166	-0.40464005	0.00391197	6	2.75974846	-1.20234013	1.32971191
6	-3.31475163	-0.13094005	1.15391195	6	3.01224852	-2.67714000	1.66081190
6	-4.70045185	-0.03814004	0.98951197	8	3.97694826	-3.27234006	0.75761199
1	-5.33305168	0.16795996	1.84651196	6	4.08684826	-2.89684010	-0.52398801
6	-5.30935192	-0.20744005	-0.24888802	6	3.24484825	-1.74234009	-1.07278800
6	-4.51915169	-0.48474005	-1.35938811	8	4.88204813	-3.44774008	-1.25318801
1	-5.01165152	-0.62284005	-2.31598806	1	3.98264837	-0.97913998	-1.35128808
6	-3.12835169	-0.59254003	-1.27118802	1	3.50864840	-2.70654011	2.63301206

6	-2.69485140	0.05405996	2.55151200	8	2.11224842	3.26365995	-1.71598804
6	-2.29915142	-0.91633999	-2.52738810	6	2.91334820	4.37836027	-2.16268802
6	-1.78305161	1.29865992	2.56971192	1	2.88784838	4.34656000	-3.25058794
1	-1.05265152	1.28845990	1.75761199	1	3.94184828	4.27506018	-1.80978811
1	-1.24185157	1.37805986	3.51991200	1	2.49024820	5.32046032	-1.80668807
1	-2.38445139	2.20625997	2.44401193	8	1.06444836	1.75325990	-0.03718802
6	-1.91835153	-1.21084011	2.97131205	15	1.91564846	2.97016001	-0.16428803
1	-1.14185154	-1.47764003	2.25411201	8	3.34454823	2.90256000	0.51131201
1	-2.60375166	-2.06194019	3.04671192	8	4.02614832	-0.55494004	1.42421198
1	-1.44735157	-1.06694007	3.95101190	6	4.10774851	1.65935993	0.39871198
6	-3.75575161	0.28935996	3.64211202	6	4.00054836	0.83855999	1.68971193
1	-4.44345188	-0.55744004	3.73511195	1	5.12934828	1.97615993	0.18581197
1	-4.34565163	1.19445992	3.46281195	1	3.74304867	1.07205987	-0.44608805
1	-3.25615168	0.41215995	4.60971212	1	4.85354853	1.03915989	2.34221196
6	-3.17615175	-1.11234009	-3.77678800	1	3.08624840	1.10405993	2.22651196
1	-3.74565172	-0.21194005	-4.03058815	8	1.34294844	4.27416039	0.52141201
1	-3.87905169	-1.94444013	-3.66408801	6	-0.05775160	4.61276007	0.35601199
1	-2.53425169	-1.34274006	-4.63468790	1	-0.68635154	3.79235983	0.70361197
6	-1.52105153	-2.23254013	-2.32638812	1	-0.27275157	4.83736038	-0.69118798
1	-0.85895157	-2.17944002	-1.46118808	1	-0.21775156	5.49756002	0.96891201
1	-0.92185152	-2.46304011	-3.21628809	6	2.47374821	-2.17063999	-2.31178808
1	-2.21585178	-3.06394005	-2.16538811	1	1.94294846	-1.31394005	-2.73478794
6	-1.33505154	0.24325997	-2.84948802	1	1.74314845	-2.94573998	-2.06888795
1	-1.89815152	1.15355992	-3.08448815	1	3.16894865	-2.56194019	-3.05638814
1	-0.71585155	-0.00174005	-3.72178817	6	1.74954844	-3.51613998	1.69201195
1	-0.67665160	0.47225994	-2.01018810	1	1.21734846	-3.47943997	0.73711193
12	0.58104843	-0.12514004	0.48641199	1	1.08294845	-3.15514016	2.47731185
1	-6.38965178	-0.13204005	-0.34638801	1	2.00984859	-4.55694008	1.90081191
8	1.76454842	-0.63444000	1.96641195				

TS-34_PL

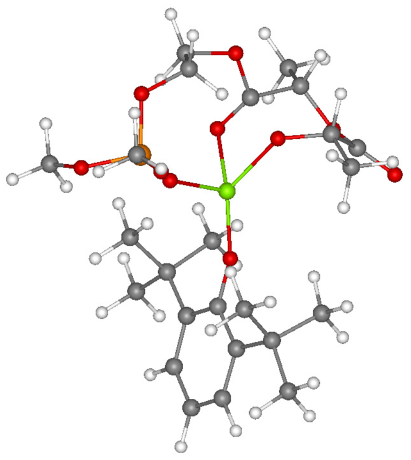

Zero-point vibrational energy	1634251.2 (Joules/Mol)
	390.59540 (Kcal/Mol)
Zero-point correction=	0.622453 (Hartree/Particle)
Thermal correction to Energy=	0.663365
Thermal correction to Enthalpy=	0.664310
Thermal correction to Gibbs Free Energy=	0.547005
Sum of electronic and zero-point Energies=	-2230.725698
Sum of electronic and thermal Energies=	-2230.684786
Sum of electronic and thermal Enthalpies=	-2230.683842
Sum of electronic and thermal Free Energies=	-2230.801147

cartesian

12	-0.56152666	0.32094792	0.04691336	1	1.31957340	2.01294804	3.40881324
8	-2.51862669	-3.10005212	-1.54468668	1	1.27187347	1.86434782	1.64261341
8	1.27987337	0.23234792	-0.04408664	1	-3.07052660	-5.10855246	-1.45698667
6	-3.39902663	-4.17985201	-1.92608666	8	-1.66152668	1.24844790	1.47081339
1	-3.31922674	-4.25685215	-3.00908685	6	-2.53332663	1.88524783	0.81191337
1	-4.42902660	-3.95215201	-1.64218664	8	-2.00382662	1.32854784	-0.93668664
6	2.54677320	-0.14925209	0.10201336	6	-2.09972668	2.33624792	-1.91468668
6	3.31467319	-0.52295208	-1.04578662	6	-2.47422671	3.40144801	0.60531336
6	4.63727331	-0.93505204	-0.85918665	8	-1.32172656	3.82034802	-0.13318664
1	5.23747349	-1.22555208	-1.71478665	6	-1.25112665	3.52914810	-1.46638668
6	5.22517347	-0.98255205	0.40011337	8	-1.38472676	-1.53175211	0.07421336
6	4.47957325	-0.59725207	1.50821340	15	-2.43662667	-2.57455206	-0.05968664
1	4.95897341	-0.62705207	2.48111320	8	-3.88882661	-2.07565212	0.38071337
6	3.15107322	-0.17415208	1.39921331	8	-3.85292673	1.52694786	0.87971336
6	2.72467327	-0.45925209	-2.46638680	6	-4.28612661	-0.71105206	0.05741337
6	2.38057327	0.25784791	2.66031313	6	-4.13012648	0.18564792	1.28831339
6	2.29747319	0.98514795	-2.79398680	1	-5.32302666	-0.78285205	-0.27378663
1	1.57697344	1.36914790	-2.07118678	1	-3.67552662	-0.30955210	-0.75458664
1	1.85557318	1.03754783	-3.79698682	1	-5.05092669	0.23524792	1.87231338
1	3.16547322	1.65284789	-2.77798676	1	-3.32282662	-0.18185209	1.92451334
6	1.53347325	-1.42865217	-2.60408664	8	-2.22532654	-3.88305211	0.80001336
1	0.75457340	-1.22905207	-1.86698663	1	6.25687313	-1.30645216	0.51531339
1	1.86407328	-2.46325207	-2.45898676	6	-1.93012667	-3.79985189	2.21511316
1	1.08977342	-1.35385215	-3.60498667	1	-1.06582665	-3.15505195	2.38071322
6	3.74217319	-0.87005204	-3.54578686	1	-1.70292664	-4.81695223	2.52921319
1	4.08397341	-1.90385211	-3.42518663	1	-2.80352664	-3.42775202	2.75491333
1	4.62017345	-0.21575208	-3.55868673	8	-0.51002663	4.16434765	-2.17458677
1	3.26817322	-0.79725206	-4.53138685	6	-2.41342664	4.10604763	1.95101333

6	3.25017333	0.21144792	3.92971325	6	-1.69022655	1.83954787	-3.29188681
1	4.11317348	0.88174796	3.86111331	1	-3.14732671	2.67964792	-1.97238660
1	3.61407328	-0.79755205	4.15171337	1	-3.38032675	3.70084810	0.06881337
1	2.65147328	0.53784794	4.78791332	1	-1.79462671	2.63574791	-4.03278685
6	1.19987345	-0.70195204	2.91641331	1	-2.32892680	0.99954796	-3.57838678
1	0.55737334	-0.80545205	2.04031324	1	-0.64892662	1.51184785	-3.29328680
1	0.58477336	-0.35215211	3.75451326	1	-2.38812661	5.18664789	1.79421341
1	1.57577324	-1.70255208	3.15981317	1	-1.51512671	3.80214810	2.49161315
6	1.89517331	1.71534789	2.52381325	1	-3.29262662	3.85604811	2.55021334
1	2.75437331	2.38904810	2.43681335				

I-4_PL

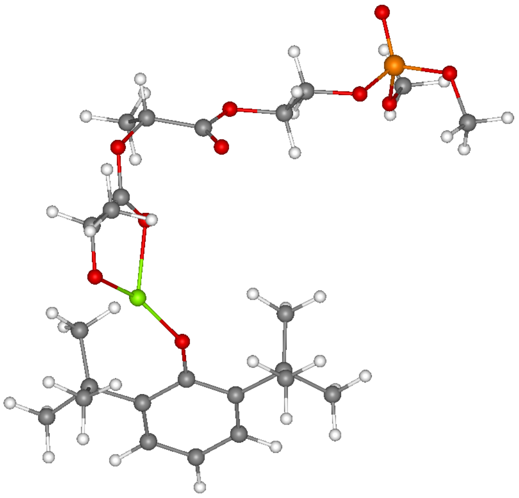

Zero-point vibrational energy	1633166.1 (Joules/Mol)
	390.33606 (Kcal/Mol)
Zero-point correction=	0.622040 (Hartree/Particle)
Thermal correction to Energy=	0.664669
Thermal correction to Enthalpy=	0.665614
Thermal correction to Gibbs Free Energy=	0.538677
Sum of electronic and zero-point Energies=	-2230.717129
Sum of electronic and thermal Energies=	-2230.674499
Sum of electronic and thermal Enthalpies=	-2230.673555
Sum of electronic and thermal Free Energies=	-2230.800491

cartesian

12	-3.49188519	2.15444803	-0.57736975	6	0.29411629	4.82924891	0.58003026
8	-3.45548511	0.30874813	-0.67226970	8	-0.94948345	5.25834894	-0.01686999
6	-3.96098518	-0.81945193	-0.16777006	6	-1.88008475	4.35964870	-0.29216987
6	-5.07467985	-0.77965200	0.72543031	6	-3.13578510	4.88324881	-0.98876971
6	-5.56427956	-1.98135138	1.24532902	1	-5.38717985	-4.12725115	1.32462895

1	-6.41037989	-1.97475147	1.92522895
6	-4.99307966	-3.20335126	0.90883023
6	-3.91498518	-3.23285127	0.02762999
1	-3.48968506	-4.19795132	-0.22697009
6	-3.37708521	-2.06965137	-0.52996987
6	-5.73687983	0.56004810	1.08292902
6	-2.19538450	-2.14245129	-1.51347101
6	-6.33277988	1.20734811	-0.19147007
1	-5.64547968	1.22474813	-1.04357076
1	-6.68187952	2.22834802	-0.00146998
1	-7.18827963	0.61644804	-0.53276986
6	-4.71688080	1.49404788	1.79372895
1	-3.67668509	1.29794812	1.50262892
1	-4.72068071	1.31194806	2.87293100
1	-4.96337986	2.55644870	1.65692902
6	-6.91607952	0.40714809	2.05953097
1	-6.60997963	-0.05015180	3.00573111
1	-7.72527981	-0.19335181	1.63282895
1	-7.32917976	1.39594805	2.29043102
6	-1.74318480	-3.58995104	-1.77127099
1	-2.53988457	-4.20085096	-2.20926905
1	-1.39068437	-4.08485126	-0.85966969
1	-0.90958357	-3.58555126	-2.48236895
6	-0.97668350	-1.38495219	-0.94736969
1	-1.20788395	-0.33305168	-0.77456969
1	-0.13488387	-1.44485223	-1.64877105
1	-0.65558386	-1.82745171	0.00253001
6	-2.60108495	-1.54905200	-2.87846899
1	-3.43458509	-2.11385131	-3.31046891
1	-1.75988483	-1.60175192	-3.58066893
1	-2.90798521	-0.50665176	-2.78516889
8	-4.05528450	3.86974859	-1.11367106
6	1.00991511	3.86414862	-0.36816978
8	-1.76178479	3.16094851	0.00903002
1	0.89031535	5.74714899	0.56633031
1	-3.50648522	5.69694901	-0.33506984
8	0.75101584	3.73654866	-1.54137099
8	6.69432020	-0.38645169	-1.07597101
6	5.95202017	-1.31365216	-1.88887107
1	6.66852045	-1.73885167	-2.59106898
1	5.16362047	-0.78875196	-2.43246889
1	5.51882029	-2.10655141	-1.27387106
8	6.93902016	1.26064813	0.87783027
15	6.03692055	0.32504815	0.18722996
8	4.68862057	0.93144822	-0.44646978
8	2.01771498	3.24404860	0.26703021
6	3.86301947	1.77384794	0.37373021
6	2.82661486	2.37604833	-0.55396980
1	4.47132015	2.55534863	0.83753031
1	3.38431621	1.17504823	1.15442896
1	3.29831600	2.95694876	-1.34997106
1	2.19661498	1.60684788	-1.00546980
8	5.42882013	-0.84775198	1.11042893
6	6.25552034	-1.40945232	2.14873099
1	6.69662046	-0.61655188	2.75603104
1	7.04662037	-2.02675128	1.71412897
1	5.59782028	-2.03045130	2.75673103
6	-2.75378489	5.51264906	-2.33896899
1	-2.06258488	6.35224867	-2.22416902
1	-3.67298508	5.86624908	-2.81116891
1	-2.29778457	4.76424885	-2.99336910
6	0.13821614	4.36374855	2.02063107
1	-0.37228391	3.40344858	2.08553100
1	-0.42668396	5.11244869	2.58193111
1	1.12541509	4.26804829	2.47643089

I-5_PLL

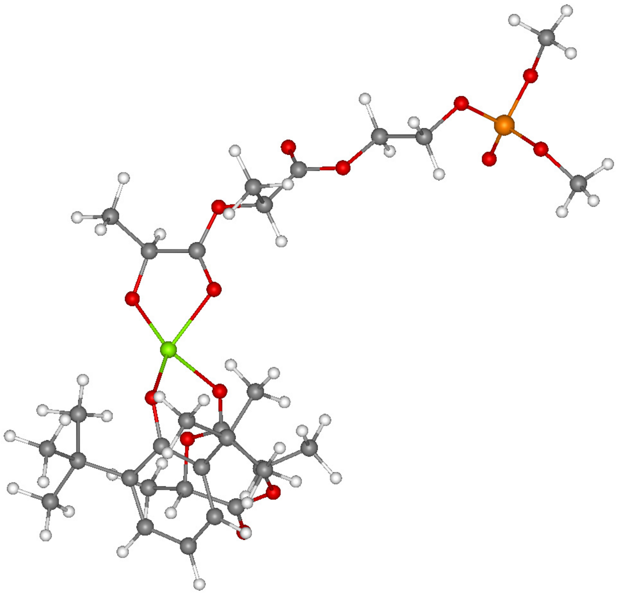

Zero-point vibrational energy	2007393.5 (Joules/Mol)
	479.77857 (Kcal/Mol)
Zero-point correction=	0.764576 (Hartree/Particle)
Thermal correction to Energy=	0.818377
Thermal correction to Enthalpy=	0.819321
Thermal correction to Gibbs Free Energy=	0.667725
Sum of electronic and zero-point Energies=	-2764.827696
Sum of electronic and thermal Energies=	-2764.773896
Sum of electronic and thermal Enthalpies=	-2764.772951
Sum of electronic and thermal Free Energies=	-2764.924547

cartesian

12	1.67067671	-1.24639022	0.80215716	6	1.12657666	3.34460974	-0.19014284
8	2.85827684	-1.32649028	-0.69464284	1	1.24117661	4.26980972	-0.75774288
6	3.66077685	-0.35919032	-1.11964285	1	0.31907672	3.46920967	0.53465712
6	4.90087652	-0.10589030	-0.44484282	1	0.87117666	2.53670979	-0.87414289
6	5.61117649	1.05300975	-0.78384280	6	4.98557663	2.42580962	3.75675726
1	6.54347658	1.28730977	-0.27794284	1	4.29397631	2.61400962	4.58035707
6	5.18347645	1.91010976	-1.79764295	1	5.82087660	3.12200975	3.84245706
6	4.07427645	1.54960978	-2.56354284	1	5.36057663	1.40330970	3.82765722
1	3.81617665	2.17400980	-3.41324282	8	1.34677672	-2.36619020	2.30045724
6	3.31587672	0.40920970	-2.27954292	6	-4.28292322	-1.83269024	1.12015724
6	5.51627636	-1.15659022	0.50425714	6	-2.99932313	-2.22319031	0.39475715
6	2.21887684	-0.06729031	-3.25284290	8	-2.09352326	-2.71079016	1.40155721
6	5.77707672	-2.43879032	-0.31604284	6	-0.82072330	-2.34949017	1.33485711
1	4.84757662	-2.82279038	-0.74234283	6	0.08597672	-2.88039017	2.44495726

1	6.21017647	-3.21799016	0.32325715	8	-0.38172328	-1.61469030	0.43745714
1	6.47787666	-2.24219036	-1.13434279	1	-2.57622313	-1.32539022	-0.06264284
6	4.62557650	-1.52079022	1.71155715	1	-0.40042329	-2.54619026	3.38295722
1	3.83507657	-2.22409034	1.44395721	8	-4.49992323	-1.96359038	2.29955721
1	4.18377638	-0.63869035	2.18635702	8	-10.30252266	1.37360978	1.09515715
1	5.22057676	-2.02699018	2.48075724	6	-11.39162254	0.67560971	0.46495718
6	6.86987638	-0.68899035	1.06715715	1	-12.26862335	0.86560965	1.08295715
1	6.77557659	0.20550969	1.69485712	1	-11.18272305	-0.39579031	0.43295717
1	7.59977674	-0.47799030	0.27945715	1	-11.56072330	1.05450964	-0.54634285
1	7.29287672	-1.48049033	1.69435716	8	-7.86922359	2.18420982	1.22465718
6	2.14497685	0.81600964	-4.50994301	15	-8.86772251	1.46920967	0.41175717
1	3.09697676	0.84900975	-5.04984283	8	-8.56712341	-0.06989031	0.06025717
1	1.84307671	1.84450972	-4.28114271	8	-5.15252352	-1.30319023	0.23395717
1	1.39567661	0.40550971	-5.19554281	6	-7.25512362	-0.43659028	-0.40384281
6	0.81407672	-0.06829031	-2.62344289	6	-6.41272354	-0.86759031	0.78405714
1	0.74747676	-0.75759029	-1.78304291	1	-6.78212357	0.39170969	-0.93904281
1	0.07147670	-0.38009033	-3.36834288	1	-7.39782333	-1.26379025	-1.10214281
1	0.52927667	0.93160975	-2.27964282	1	-6.25052357	-0.03729030	1.47475719
6	2.56307673	-1.49879026	-3.71784282	1	-6.88672352	-1.69079030	1.32355714
1	3.53287673	-1.51679027	-4.22654295	8	-9.10092258	2.07350969	-1.06394279
1	1.80497670	-1.85999036	-4.42364264	6	-9.07162285	3.50480962	-1.23214281
1	2.60507679	-2.18769026	-2.87204289	1	-8.18372345	3.92750978	-0.75764287
1	5.75407648	2.80320978	-2.04124284	1	-9.97082329	3.95640969	-0.80424285
8	4.15387630	4.99610996	2.85985708	1	-9.04702282	3.68340969	-2.30694294
8	2.74557686	4.15410995	1.35545719	6	0.06387675	-4.41949034	2.44295716
6	3.74777675	4.03840971	2.25215721	1	-0.94012332	-4.82329035	2.60365725
6	4.30097675	2.63090968	2.42495728	1	0.71827668	-4.76089048	3.24815726
8	3.22927690	1.64790976	2.32555723	1	0.46387672	-4.80449009	1.49925721
6	2.39027667	1.74440968	1.30735719	6	-3.21722317	-3.29789019	-0.66314286
6	2.43197680	3.02880979	0.50805718	1	-3.60712314	-4.21259022	-0.20954284
1	3.23697686	2.89990973	-0.23344284	1	-2.26912332	-3.52429032	-1.15614283
8	1.62137675	0.82360971	1.05385709	1	-3.92422318	-2.94389033	-1.41574287
1	4.98957634	2.42680979	1.59465718				

I-5_PLP

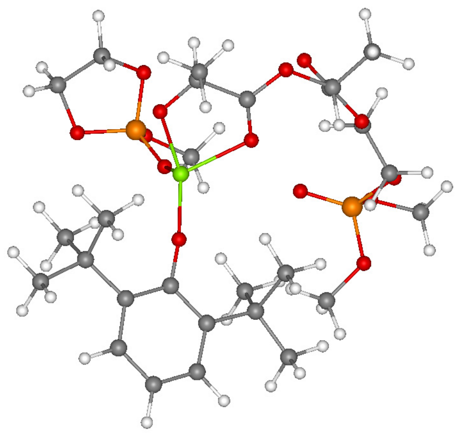

Zero-point vibrational energy	1939469.4 (Joules/Mol)
	463.54431 (Kcal/Mol)
Zero-point correction=	0.738705 (Hartree/Particle)
Thermal correction to Energy=	0.791599
Thermal correction to Enthalpy=	0.792544
Thermal correction to Gibbs Free Energy=	0.642759
Sum of electronic and zero-point Energies=	-2991.317977
Sum of electronic and thermal Energies=	-2991.265082
Sum of electronic and thermal Enthalpies=	-2991.264138
Sum of electronic and thermal Free Energies=	-2991.413923

cartesian

8	2.20130348	-0.79025316	0.15639110	6	-2.08059669	1.18014681	-3.32890892
6	2.80280352	-1.97205317	0.15799110	1	-2.55339646	0.84734678	-4.25000906
6	2.07160354	-3.14145303	0.54179108	1	-2.76139665	1.81614673	-2.76520896
6	2.73060346	-4.37415314	0.54139107	1	-1.76839674	0.32704681	-2.72930884
1	2.19560337	-5.27295351	0.82999104	1	3.13010335	3.76394701	-3.68920898
6	4.06850338	-4.49465322	0.18049107	1	1.66360331	4.28584671	-4.57530880
6	4.77260303	-3.35475302	-0.19290891	12	1.52190328	0.92304683	-0.05890891
1	5.81530333	-3.46755314	-0.47130889	8	2.21060348	2.69804692	0.27819109
6	4.17950296	-2.08895302	-0.21780892	6	-2.50459647	2.50444698	2.04409099
6	0.58920336	-3.06155300	0.95039111	6	-1.50119674	2.55104685	3.19539094
6	5.00630331	-0.86105323	-0.64170891	8	-0.31409666	3.24184680	2.75909114
6	-0.26049668	-2.52895308	-0.22150892	6	0.42020336	2.62704682	1.83679104
1	0.08870333	-1.55645323	-0.56950891	6	1.64270329	3.38434696	1.32189107
1	-1.31189668	-2.43335319	0.08059110	8	0.12260333	1.50354683	1.41619098
1	-0.21339667	-3.22175312	-1.06940889	1	-1.24009669	1.52874672	3.47629094
6	0.42300335	-2.16455317	2.19379115	1	1.24080324	4.36954689	1.01059103
1	0.79990327	-1.15725327	2.01629114	8	-2.57449651	3.32184696	1.15689099
1	0.97270334	-2.58695316	3.04239106	8	-7.00179672	-0.84895325	-1.83090901

1	-0.63519669	-2.09505320	2.47829103	6	-8.17509651	-1.41845322	-1.22120893
6	0.00700334	-4.43705320	1.32089102	1	-8.96269703	-1.36015320	-1.97180891
1	0.51780331	-4.88725328	2.17879105	1	-8.45649719	-0.84085321	-0.33820891
1	0.04860333	-5.14495325	0.48599112	1	-7.99979687	-2.46165299	-0.94650888
1	-1.04769671	-4.31875324	1.59639108	8	-4.51309681	-0.24995318	-1.88690901
6	6.45980310	-1.22375321	-0.99600887	15	-5.58319664	-0.90545321	-1.11170888
1	6.52160311	-1.92935312	-1.83140898	8	-5.92829704	-0.30065319	0.33499110
1	6.99340296	-0.31475320	-1.29720891	8	-3.32719660	1.44834673	2.18299103
1	6.99950314	-1.64985323	-0.14350891	6	-4.87399673	-0.10835320	1.29789102
6	5.08590317	0.16094682	0.51059109	6	-4.32059669	1.29814672	1.14999104
1	4.10230303	0.50324678	0.83219105	1	-4.08419657	-0.85325325	1.16799104
1	5.66540337	1.04084682	0.20519109	1	-5.32929659	-0.25435320	2.27919102
1	5.58310318	-0.28815320	1.37749100	1	-3.86309648	1.43824673	0.16979109
6	4.40180302	-0.22105318	-1.90820897	1	-5.10619688	2.04684687	1.28409100
1	4.48250341	-0.90785325	-2.75780892	8	-5.30819702	-2.44485307	-0.73970890
1	4.93440342	0.70244682	-2.16690898	6	-4.59289694	-3.27815318	-1.67630899
1	3.34240341	0.01234680	-1.78870893	1	-3.66729665	-2.79245305	-1.98880899
1	4.55720329	-5.46605349	0.19029109	1	-5.21729660	-3.49075317	-2.54810905
8	0.44680330	1.13164675	-1.76140893	1	-4.37039661	-4.20385313	-1.14680898
15	0.30070332	2.21004677	-2.78610897	6	2.62270331	3.63414693	2.48299098
8	0.18050334	3.71004701	-2.23420906	1	2.17060351	4.21374655	3.29399109
8	1.58260334	2.40634680	-3.72370887	1	3.47890353	4.18514681	2.08689094
6	1.46680331	4.36474657	-2.39070892	1	2.99140334	2.68304682	2.87899113
6	2.04020333	3.77914691	-3.68280888	6	-2.05809665	3.32574701	4.37859106
1	1.27920330	5.43634653	-2.46010900	1	-2.30809665	4.34814644	4.08629084
1	2.07680345	4.11834669	-1.51520896	1	-1.31719673	3.35814691	5.18079090
8	-0.92159665	1.96214676	-3.74190903	1	-2.95709658	2.83234692	4.75549126

S1.10. Transesterification of PP

PP

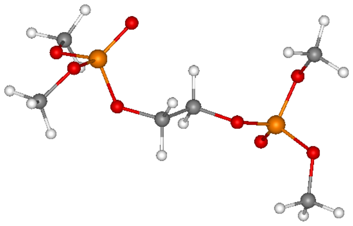

Zero-point vibrational energy	662155.7 (Joules/Mol)
	158.25901 (Kcal/Mol)
Zero-point correction=	0.252202 (Hartree/Particle)
Thermal correction to Energy=	0.273321
Thermal correction to Enthalpy=	0.274265
Thermal correction to Gibbs Free Energy=	0.197927
Sum of electronic and zero-point Energies=	-1522.311875
Sum of electronic and thermal Energies=	-1522.290756
Sum of electronic and thermal Enthalpies=	-1522.289812
Sum of electronic and thermal Free Energies=	-1522.366150

cartesian

8	-2.23931909	-1.30730009	1.28460002	15	3.11028099	-0.37279999	-0.36570004
15	-2.87011909	-0.34270000	0.36739996	8	4.19738150	0.62959999	0.24689996
8	-3.79121900	0.72490001	1.10769999	8	3.13768101	-0.61720002	-1.81729996
6	-4.51551867	1.72620010	0.37159997	8	3.33588099	-1.68499994	0.50709999
1	-3.84041905	2.29600000	-0.27230003	1	-4.96001863	2.38569999	1.11660004
1	-5.29891872	1.25830007	-0.22840002	1	-0.30411887	1.85730004	-0.35800004
8	-3.85401893	-0.90130001	-0.76230001	1	-0.61371887	0.93699998	1.13250005
6	-3.41521907	-1.98600006	-1.60169995	1	0.23648113	-1.13279998	0.03799997
1	-3.06591892	-2.82200003	-0.99180001	6	3.34458113	-1.63849998	1.94540000
1	-2.62231898	-1.64650011	-2.27300000	6	4.41398144	1.89820004	-0.39650002
1	-4.28501892	-2.28920007	-2.18359995	1	5.26738119	2.35189986	0.10699997
8	-1.82561886	0.51059997	-0.52860004	1	3.53478098	2.53740001	-0.27940005
6	-0.55301887	0.87229997	0.04279997	1	4.63858128	1.75410008	-1.45560002
6	0.48948115	-0.15080000	-0.37120003	1	3.41308093	-2.67340016	2.27940011
8	1.74268115	0.30109999	0.17809996	1	2.42268109	-1.18889999	2.32279992
1	0.55938113	-0.21260001	-1.46070004	1	4.21018124	-1.07360005	2.29820013

I-1_L_PP

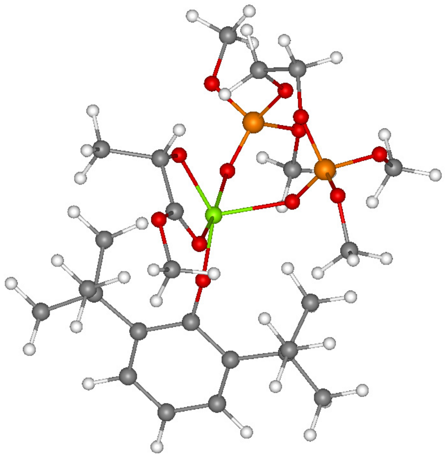

Zero-point vibrational energy	1805576.4 (Joules/Mol)
	431.54311 (Kcal/Mol)
Zero-point correction=	0.687708 (Hartree/Particle)
Thermal correction to Energy=	0.736443
Thermal correction to Enthalpy=	0.737388
Thermal correction to Gibbs Free Energy=	0.603169
Sum of electronic and zero-point Energies=	-2725.458342
Sum of electronic and thermal Energies=	-2725.409606
Sum of electronic and thermal Enthalpies=	-2725.408662
Sum of electronic and thermal Free Energies=	-2725.542881

cartesian

12	0.36787078	0.46353373	0.43730843	8	3.91067076	-0.15746626	-2.42979169
8	-1.40112925	0.02943373	-0.10029157	6	3.40087080	-1.47216618	-2.75199175
6	-2.64592934	-0.34476626	-0.32619157	1	2.31117082	-1.48406625	-2.71749163
6	-3.05842924	-0.71616632	-1.65109158	1	3.80237079	-2.19366646	-2.03949165
6	-4.37832928	-1.12416625	-1.86209154	1	3.75257063	-1.69486618	-3.75959158
1	-4.70242929	-1.41056621	-2.85749173	8	0.17597078	2.65273356	0.11660843
6	-5.30982924	-1.17556620	-0.83059156	8	1.46197081	1.17433381	1.89710844
6	-4.91432905	-0.79936630	0.44910842	6	0.75437081	3.25823355	1.01910841
1	-5.65392923	-0.83466631	1.24250841	6	1.53587079	2.52633357	2.10910845
6	-3.61202908	-0.38136625	0.73630846	6	1.03937078	2.94623375	3.50310826
6	-2.07682920	-0.66596627	-2.83529162	1	2.57207084	2.91343355	2.00310850
6	-3.24582934	0.03823373	2.17120838	1	-6.33082914	-1.49736619	-1.02219152
6	-1.54742932	0.76943368	-3.02619171	8	0.75157082	4.58753347	1.10170841
1	-1.06842923	1.13963377	-2.11929154	6	0.00927079	5.28683376	0.08640844
1	-0.82522923	0.80193371	-3.85349154	1	0.06837079	6.34003353	0.35720843
1	-2.37252927	1.44653380	-3.27399158	1	-1.02812910	4.94883347	0.07190844
6	-0.91082925	-1.64636624	-2.60019159	1	0.45917079	5.11953354	-0.89439154

1	-0.38342923	-1.41936624	-1.67319155
1	-1.29142928	-2.67316628	-2.54149175
1	-0.19692922	-1.60066628	-3.43419170
6	-2.73052931	-1.07506621	-4.16679144
1	-3.10322928	-2.10496640	-4.14839172
1	-3.56002927	-0.41496626	-4.44189167
1	-1.98512936	-1.01346624	-4.96889162
6	-4.44152927	-0.04816628	3.13670826
1	-5.26752949	0.60493368	2.83460832
1	-4.82682943	-1.06916618	3.23210835
1	-4.12012911	0.27203372	4.13460827
6	-2.15702939	-0.89086628	2.74450827
1	-1.26852918	-0.93136632	2.11560845
1	-1.85422921	-0.56006628	3.74580836
1	-2.54422927	-1.91296625	2.82990837
6	-2.78212929	1.50883377	2.18720841
1	-3.60312939	2.16653371	1.87920845
1	-2.47382927	1.80383372	3.19830823
1	-1.95072937	1.67793381	1.50250840
8	1.75977087	0.37903374	-1.14519155
15	3.10967064	0.88293368	-1.52629149
8	3.11237073	2.25493360	-2.31939173
6	2.09687090	2.49663353	-3.31669164
1	2.22787070	1.81663382	-4.16239166
1	1.10247076	2.38103366	-2.88419175
8	4.10017061	1.30263376	-0.36409158
6	4.86747074	0.39083374	0.44450843
6	4.03817081	-0.43936628	1.39620841
8	3.58087063	-1.62836623	0.68370843
1	3.18257070	0.14343372	1.77780843
15	2.19337082	-2.31126642	1.07810843
8	2.27567077	-3.57776642	0.11420844
8	0.96567076	-1.49846625	0.93860847
8	2.32897067	-2.84126639	2.57600832
1	2.24937081	3.52283359	-3.64809155
1	1.13777077	4.02293348	3.67350841
1	1.63337088	2.41223359	4.24950838
1	-0.00602922	2.65463376	3.63570833
1	5.47937059	-0.24256626	-0.20519157
1	5.52517080	1.04153371	1.02340841
1	4.67487049	-0.77576631	2.22100830
6	3.37137079	-3.76176643	2.94340825
6	1.05567074	-4.31236649	-0.15729158
1	0.73617077	-4.85476637	0.73600847
1	1.30547071	-5.01646614	-0.94959152
1	0.26857078	-3.63286638	-0.48499158
1	3.26627064	-3.92256641	4.01560831
1	4.35777092	-3.34226632	2.72800827
1	3.25227070	-4.70846653	2.41110826

TS-12_L_PP

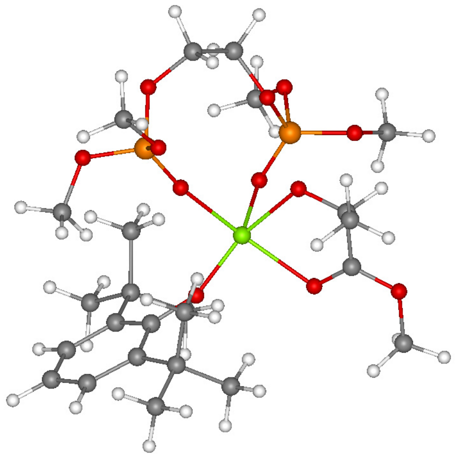

Zero-point vibrational energy	1804775.0 (Joules/Mol)
	431.35159 (Kcal/Mol)
Zero-point correction=	0.687403 (Hartree/Particle)

Thermal correction to Energy= 0.735223
Thermal correction to Enthalpy= 0.736168
Thermal correction to Gibbs Free Energy= 0.602895
Sum of electronic and zero-point Energies= -2725.440521
Sum of electronic and thermal Energies= -2725.392700
Sum of electronic and thermal Enthalpies= -2725.391756
Sum of electronic and thermal Free Energies= -2725.525029

cartesian

12	0.62231660	0.57682663	-0.00058195	8	4.23031664	-0.79087335	-2.03178215
8	-1.22688329	0.73242664	-0.42718196	6	3.63361669	-0.99977338	-3.31778216
6	-2.48148322	0.34672663	-0.55478191	1	3.36951661	-0.05037338	-3.79058194
6	-2.95438337	-0.20377338	-1.79568195	1	2.73191667	-1.61217332	-3.24398208
6	-4.26728344	-0.68097335	-1.86168194	1	4.38621664	-1.51377344	-3.91908216
1	-4.63638353	-1.11477339	-2.78548217	8	0.82891667	2.75272655	0.18431805
6	-5.13828373	-0.61227334	-0.77858192	8	2.34171653	0.77862662	1.03791809
6	-4.70238352	-0.00527338	0.39561805	6	1.75461662	3.06232667	0.93321806
1	-5.40718365	0.08302662	1.21621811	6	2.64671659	2.00992656	1.58101809
6	-3.40558338	0.49722660	0.53681803	6	2.46111655	2.02762651	3.10681796
6	-2.05678344	-0.26597339	-3.04658198	1	3.68371677	2.30722666	1.36071801
6	-3.00618339	1.22762656	1.83341801	1	-6.15288353	-0.99517339	-0.85968190
6	-1.49108338	1.13152671	-3.37488198	8	2.04041672	4.32622671	1.23181808
1	-0.92408335	1.54482651	-2.54088211	6	1.18021667	5.32562637	0.64821810
1	-0.83548331	1.07732654	-4.25328159	1	1.55241668	6.27922678	1.01901805
1	-2.30718327	1.82542658	-3.60618210	1	0.14741664	5.16482639	0.96081805
6	-0.91138339	-1.27627349	-2.84108210	1	1.23891664	5.28562641	-0.44098195
1	-0.30458334	-1.04087329	-1.96748197	8	3.60231662	-1.21097326	0.31551805
1	-1.31428337	-2.28807354	-2.71278214	6	3.94781661	-2.57207346	0.04541805
1	-0.24888334	-1.28937340	-3.71608210	6	3.20511675	-3.44857335	1.02921808
6	-2.82658339	-0.72647339	-4.29788160	8	1.83431661	-3.67577338	0.63721806
1	-3.20078325	-1.75237346	-4.20918179	1	3.25131655	-3.01527333	2.03231812
1	-3.67318320	-0.06977338	-4.52538204	15	0.60821664	-2.79227352	1.14951801
1	-2.15218329	-0.70407337	-5.16168165	8	-0.56738341	-3.78557348	0.77211809
6	-4.19128370	1.40282655	2.80061793	8	0.47641665	-1.42607331	0.60451806
1	-5.01188374	1.97072649	2.34961796	8	0.72001660	-2.73997355	2.74291801
1	-4.58938360	0.44542664	3.15461802	1	5.60171652	2.63332653	-1.07848191
1	-3.85428357	1.95832658	3.68371797	1	2.71571660	3.00052667	3.53861785
6	-1.93318343	0.43572664	2.60431790	1	3.11511660	1.26482654	3.53621793
1	-1.06328332	0.21172664	1.98731804	1	1.42961669	1.77672672	3.37151790
1	-1.59728336	0.99352664	3.48761797	1	3.71101665	-2.86617351	-0.97828197
1	-2.34028339	-0.52107334	2.95151806	1	5.02541637	-2.69487333	0.20041806

6	-2.49928331	2.64632654	1.50081801	1	3.66291666	-4.44087362	1.04471803
1	-3.30218339	3.23372650	1.04091799	6	0.63631666	-3.94397354	3.52591801
1	-2.18908334	3.16352654	2.41881800	6	-1.91568327	-3.27657342	0.57421809
1	-1.65948343	2.62172651	0.80641806	1	-2.37258339	-3.05557346	1.54061806
8	2.03211665	0.17412663	-1.42518198	1	-2.45868325	-4.07847309	0.07721805
15	3.38831663	-0.08517338	-0.80728191	1	-1.90618324	-2.38167334	-0.04778195
8	4.41131639	1.10862660	-0.48228195	1	0.77101660	-3.63837337	4.56291819
6	5.02941656	1.83672667	-1.55538189	1	1.42151666	-4.65137339	3.24451804
1	5.69471645	1.18762660	-2.12798214	1	-0.34108338	-4.41487360	3.39971805
1	4.27601671	2.27902651	-2.21378207				

I-2_L_PP

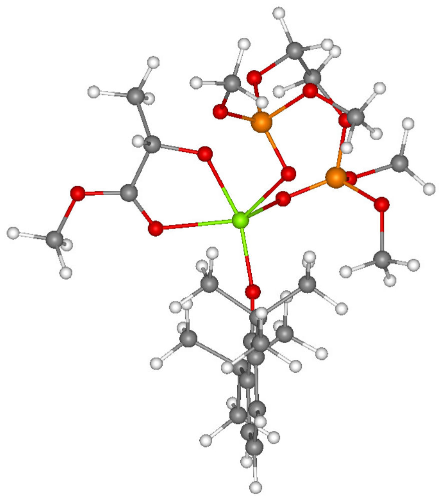

Zero-point vibrational energy	1808418.4 (Joules/Mol)
	432.22237 (Kcal/Mol)
Zero-point correction=	0.688790 (Hartree/Particle)
Thermal correction to Energy=	0.736646
Thermal correction to Enthalpy=	0.737590
Thermal correction to Gibbs Free Energy=	0.605468
Sum of electronic and zero-point Energies=	-2725.444065
Sum of electronic and thermal Energies=	-2725.396209
Sum of electronic and thermal Enthalpies=	-2725.395265
Sum of electronic and thermal Free Energies=	-2725.527388

cartesian

12	0.34140223	0.15011327	0.48016381	8	3.99010229	0.73071325	-1.86023617
8	-1.44929779	-0.10288674	-0.09183620	6	3.49400234	0.18591326	-3.08083630
6	-2.71569777	0.04901326	-0.46133620	1	2.68550229	0.79351330	-3.49953628
6	-3.06579757	0.94911331	-1.52443624	1	3.12040234	-0.83118671	-2.93643618
6	-4.40279770	1.03461325	-1.92433619	1	4.33750200	0.17741327	-3.77713633

1	-4.68119812	1.70191324	-2.73293614
6	-5.40589809	0.28671324	-1.32003617
6	-5.06979799	-0.55868673	-0.26933622
1	-5.86699772	-1.12268674	0.20246381
6	-3.75639749	-0.69748670	0.19066380
6	-2.01339769	1.82251322	-2.23423624
6	-3.48299766	-1.61798680	1.39786375
6	-1.33159781	2.76551318	-1.22193623
1	-0.93169785	2.22471333	-0.36293620
1	-0.51129782	3.31981325	-1.69363618
1	-2.05579758	3.49121332	-0.83503622
6	-0.97759783	0.93971330	-2.95693612
1	-0.43049774	0.29641324	-2.27033615
1	-1.47949779	0.30791324	-3.69883633
1	-0.24429777	1.56241322	-3.48473620
6	-2.62879777	2.73311329	-3.31293631
1	-3.09069777	2.16031313	-4.12423611
1	-3.37839770	3.41871333	-2.90363622
1	-1.83549774	3.34461331	-3.75813627
6	-4.75289774	-2.33278680	1.89516377
1	-5.52169800	-1.63208675	2.23776388
1	-5.19329786	-2.98008680	1.12866378
1	-4.49199772	-2.96908689	2.74866390
6	-2.47339773	-2.72508669	1.04366386
1	-1.50509775	-2.30488682	0.77746379
1	-2.33049774	-3.39778686	1.89866376
1	-2.84419775	-3.32368684	0.20276380
6	-2.95329762	-0.78358674	2.57976389
1	-3.70839763	-0.05528674	2.89796376
1	-2.72659779	-1.43148673	3.43666387
1	-2.05069757	-0.23588674	2.31376386
8	1.74660218	0.22021325	-1.04163623
15	2.97830224	0.99301332	-0.55293620
8	3.12290239	2.61561322	-0.57363623
6	3.92340231	3.33441329	-1.51903617
1	4.97260189	3.03871322	-1.46013618
1	3.56330228	3.19011331	-2.53973627
8	-0.20199779	1.31361318	2.32796383
8	1.92750216	1.31231320	0.97726381
6	0.62450224	2.15781331	2.66356373
6	1.97620225	2.28361320	1.97796381
6	3.12760234	2.11081314	2.97006369
1	2.02890229	3.28971314	1.53806376
1	-6.43689775	0.36891323	-1.65613627
8	0.41370225	3.03161311	3.63756371
6	-0.87029779	2.95181322	4.29386425
1	-0.85949779	3.74291325	5.04126406
1	-0.99199784	1.97501326	4.76406384
1	-1.67149770	3.11051321	3.57076383
8	4.05610228	0.37361324	0.52346379
6	4.89310217	-0.74898672	0.28826380
6	4.22980213	-2.03328681	0.74276376
8	3.27770233	-2.46608686	-0.25753617
1	3.73800230	-1.88448679	1.70766377
15	1.75490224	-2.77148676	0.08296380
8	1.25020218	-3.07228684	-1.38283622
8	1.01220226	-1.76468682	0.89446378
8	1.73140216	-4.14338636	0.90346378
1	3.81750226	4.38581324	-1.24303627
1	3.08230233	2.87691331	3.74886370
1	4.07390213	2.20151329	2.43496370
1	3.09060240	1.12181318	3.43486381
1	5.18850231	-0.81568670	-0.76153624
1	5.79010201	-0.59288675	0.89756376
1	4.97610188	-2.82858682	0.83516377
6	2.30860233	-5.33378649	0.33846378
6	-0.15099776	-3.00168681	-1.75353622
1	-0.68769777	-3.85318685	-1.33103621
1	-0.16259778	-3.05048680	-2.84093618
1	-0.59749782	-2.06588674	-1.41333616
1	2.21220231	-6.10688639	1.09986377
1	3.36410236	-5.17928648	0.09856380
1	1.76720226	-5.63068676	-0.56283623

TS-23_L_PP

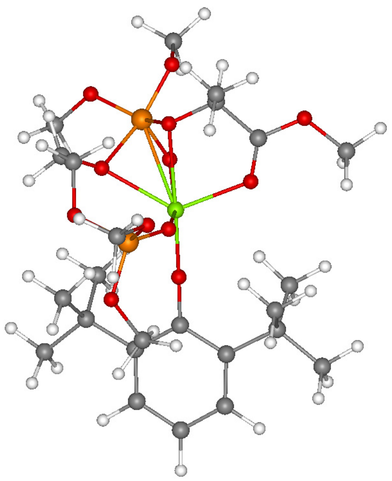

Zero-point vibrational energy	1809287.4 (Joules/Mol)
	432.43006 (Kcal/Mol)
Zero-point correction=	0.689121 (Hartree/Particle)
Thermal correction to Energy=	0.735856
Thermal correction to Enthalpy=	0.736800
Thermal correction to Gibbs Free Energy=	0.606774
Sum of electronic and zero-point Energies=	-2725.431937
Sum of electronic and thermal Energies=	-2725.385202
Sum of electronic and thermal Enthalpies=	-2725.384258
Sum of electronic and thermal Free Energies=	-2725.514284

cartesian

12	-0.35848781	0.22092885	-0.20759629	8	-3.80738783	1.87062883	2.01450372
8	1.50071216	0.33802885	-0.07209629	6	-3.49618769	1.44172883	3.33520365
6	2.80041218	0.45152885	0.17140371	1	-2.43288779	1.20502889	3.43650365
6	3.26791215	1.17512882	1.31720376	1	-4.08798790	0.56692886	3.62010360
6	4.64361191	1.21752882	1.56510377	1	-3.74438787	2.27812886	3.99530363
1	5.01791191	1.75112891	2.43230367	8	-0.97278780	0.94692886	-2.04419637
6	5.56671190	0.59912884	0.72910368	8	-3.36048770	0.19202885	-0.80699629
6	5.11011219	-0.06327115	-0.40509629	6	-2.05518794	1.25522888	-2.54229641
1	5.84511185	-0.51977116	-1.05949628	6	-3.40718770	0.70442885	-2.11279631
6	3.75031209	-0.14837116	-0.71939629	6	-3.78408766	-0.43777114	-3.06359625
6	2.30231214	1.92022896	2.25910354	1	-4.13388777	1.51372886	-2.20949626
6	3.31101227	-0.85677117	-2.01509643	1	6.63031197	0.64642888	0.95010370
6	1.51021218	2.98502874	1.47290373	8	-2.13888788	2.07562876	-3.57609630
1	0.95691222	2.55342889	0.63900369	6	-0.89318782	2.60212874	-4.08589649
1	0.79131216	3.49152875	2.12750363	1	-1.17378783	3.23492885	-4.92549658
1	2.19451213	3.73902893	1.06800377	1	-0.24478783	1.78782880	-4.41109657
6	1.34921217	0.93362892	2.96120358	1	-0.39058784	3.18412876	-3.31239629

1	0.75581223	0.35882887	2.25010371
1	1.91951227	0.22872886	3.57720375
1	0.65191215	1.46912885	3.61600375
6	3.04001212	2.67292881	3.38140368
1	3.59811234	1.99842882	4.03990364
1	3.73321223	3.42512894	2.99040365
1	2.30531216	3.19792891	4.00210381
6	4.50611210	-1.37167120	-2.83769631
1	5.18101215	-0.56457114	-3.14209628
1	5.09081221	-2.12337112	-2.29639626
1	4.13361216	-1.84787118	-3.75199628
6	2.43611217	-2.08517122	-1.70049620
1	1.54051208	-1.81497109	-1.14199626
1	2.12371230	-2.58317113	-2.62699628
1	3.00421214	-2.81177115	-1.10749626
6	2.54881215	0.13092884	-2.92199636
1	3.20451212	0.95912886	-3.21389627
1	2.21251225	-0.37267113	-3.83789635
1	1.67951226	0.55132884	-2.41609645
8	-1.62848783	1.24192882	0.82900369
15	-3.15218782	1.11432886	0.68200368
8	-3.70328784	2.40172887	-0.22409630
6	-3.66338778	3.74402881	0.26090372
1	-4.50348806	3.94352889	0.92920369
1	-2.72608781	3.94972873	0.78470373
8	-3.43948770	-0.39307114	1.41860378
6	-4.38458824	-1.37587118	1.08300376
6	-3.75638771	-2.52947116	0.32510370
8	-2.67928767	-3.11447120	1.11630380
1	-3.36888766	-2.18317103	-0.63179630
15	-1.17548776	-2.95887113	0.66040373
8	-0.43298781	-3.15117121	2.04540372
8	-0.81808782	-1.73767114	-0.11429629
8	-0.81948781	-4.21717119	-0.25629631
1	-3.73378778	4.38492870	-0.62359631
1	-3.85798788	-0.07917115	-4.09389639
1	-4.75788784	-0.83387113	-2.76459646
1	-3.04698777	-1.24427116	-3.01989627
1	-4.80928802	-1.74907112	2.02360368
1	-5.20828819	-0.98847115	0.47080371
1	-4.48398781	-3.33137107	0.17040370
6	-1.11788785	-5.55057144	0.19710371
6	1.01141214	-3.01097107	2.09240365
1	1.48541212	-3.83107114	1.54810381
1	1.27471209	-3.05767107	3.14730358
1	1.32011223	-2.05307126	1.67140377
1	-0.73818779	-6.22217131	-0.57169628
1	-2.19668794	-5.68317127	0.30850372
1	-0.62078786	-5.75777149	1.14840376

I-3_L_PP

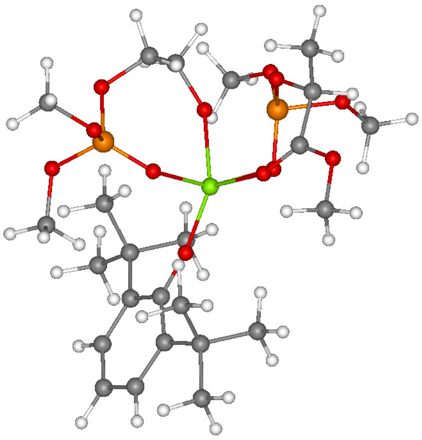

Zero-point vibrational energy	1805168.3 (Joules/Mol)
	431.44557 (Kcal/Mol)
Zero-point correction=	0.687552 (Hartree/Particle)
Thermal correction to Energy=	0.735150

Thermal correction to Enthalpy=	0.736094
Thermal correction to Gibbs Free Energy=	0.604724
Sum of electronic and zero-point Energies=	-2725.440129
Sum of electronic and thermal Energies=	-2725.392532
Sum of electronic and thermal Enthalpies=	-2725.391588
Sum of electronic and thermal Free Energies=	-2725.522958

cartesian

12	-0.57742542	0.12980369	-0.12309286	8	-3.41372538	2.28010368	1.83130717
8	1.24757457	0.56770366	-0.31139287	6	-2.53082538	2.55930376	2.92530727
6	2.55947447	0.71650368	-0.31089285	1	-1.48792553	2.37780356	2.66660714
6	3.23017454	1.25210369	0.83990717	1	-2.81012559	1.94940376	3.78850722
6	4.62697458	1.31330371	0.83450711	1	-2.67122555	3.61430359	3.17340708
1	5.15267467	1.70180368	1.70070720	8	-1.87582541	-0.51099634	-1.76739287
6	5.38247442	0.90130365	-0.25849286	8	-3.82422543	0.18370366	0.03850714
6	4.72507429	0.44750369	-1.39759278	6	-3.03102541	-0.41719633	-2.15769291
1	5.32687473	0.16600367	-2.25559282	6	-4.23822546	-0.04369632	-1.29659283
6	3.33177447	0.35490367	-1.46579278	6	-5.28352547	-1.15499628	-1.28419280
6	2.44627452	1.79510367	2.05040717	1	-4.66702557	0.86580366	-1.72709286
6	2.65797448	-0.10659632	-2.77209282	1	6.46817446	0.95800364	-0.23279285
6	1.48957467	2.91640377	1.59530711	8	-3.37892556	-0.63149631	-3.42159271
1	0.76867455	2.57320356	0.85240710	6	-2.30532551	-0.95219630	-4.33249331
1	0.94017464	3.31930375	2.45640707	1	-2.78232551	-1.08219635	-5.30209303
1	2.06047463	3.74040365	1.15310717	1	-1.80752540	-1.87049627	-4.01779318
6	1.66507459	0.66980368	2.75540709	1	-1.58342552	-0.13529631	-4.36099291
1	0.96677452	0.17560366	2.07920718	8	-1.96702552	0.29710367	1.47230721
1	2.35067463	-0.09029632	3.14660716	6	-2.57282543	-0.48689634	2.47970724
1	1.09177446	1.06780362	3.60230708	6	-2.58882546	-1.97919631	2.21370721
6	3.36847448	2.41380358	3.11600709	8	-1.28952539	-2.58709621	2.49020720
1	4.06087446	1.68320370	3.54860711	1	-2.90322542	-2.19269633	1.18980718
1	3.95337439	3.24950361	2.71790719	15	-0.30302542	-2.87969637	1.28490722
1	2.75667453	2.80520368	3.93700719	8	1.06767464	-3.02969623	2.05950713
6	3.67167449	-0.32449633	-3.90939283	8	-0.34482542	-1.89189637	0.17530714
1	4.23257446	0.58630365	-4.14419317	8	-0.67622542	-4.31299639	0.68840718
1	4.38807440	-1.12149632	-3.68229294	1	-4.45772552	4.27400351	-1.26159286
1	3.13527441	-0.62209630	-4.81789303	1	-5.64662552	-1.35089636	-2.29489279
6	1.93567443	-1.45169628	-2.56289291	1	-6.12272549	-0.83789635	-0.66139281
1	1.19357467	-1.40129638	-1.76639283	1	-4.87092543	-2.07929635	-0.87059283
1	1.43097448	-1.76639628	-3.48599291	1	-2.04922533	-0.32179633	3.43090725
1	2.65957451	-2.23259640	-2.30089283	1	-3.61692548	-0.17969632	2.61890721
6	1.66887450	0.96840370	-3.26829290	1	-3.27352548	-2.46929622	2.91010714

1	2.19917440	1.90200365	-3.48609281	6	-0.69022542	-5.47269630	1.54250717
1	1.18167448	0.63710368	-4.19499302	6	2.32067442	-2.87769628	1.33630717
1	0.90107459	1.18830371	-2.52619290	1	2.43387461	-3.68499637	0.60900712
8	-1.62772536	1.85010362	-0.24229285	1	3.10037446	-2.94269633	2.09270716
15	-2.94932556	1.53750372	0.46130714	1	2.35667443	-1.90749633	0.84010708
8	-4.00302553	2.47680378	-0.43429288	1	-0.96532542	-6.31249619	0.90590715
6	-3.70642543	3.85910368	-0.58469284	1	-1.42842531	-5.35289621	2.33960724
1	-3.77262545	4.37980366	0.37770715	1	0.29857457	-5.64229631	1.97530723
1	-2.70922542	4.00260353	-1.01279283				

TS-34_L_PP

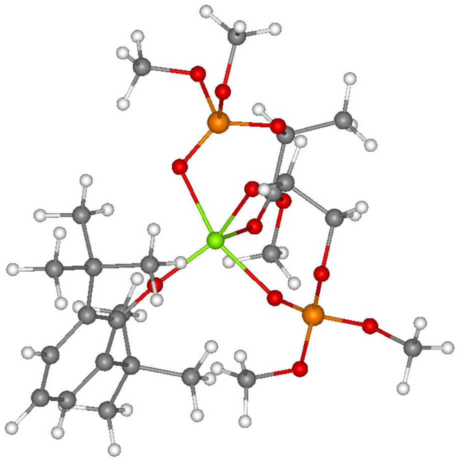

Zero-point vibrational energy	1808585.4 (Joules/Mol)
	432.26228 (Kcal/Mol)
Zero-point correction=	0.688854 (Hartree/Particle)
Thermal correction to Energy=	0.736437
Thermal correction to Enthalpy=	0.737381
Thermal correction to Gibbs Free Energy=	0.607186
Sum of electronic and zero-point Energies=	-2725.439618
Sum of electronic and thermal Energies=	-2725.392034
Sum of electronic and thermal Enthalpies=	-2725.391090
Sum of electronic and thermal Free Energies=	-2725.521285

cartesian

12	0.47402164	0.07619025	0.00941206	8	3.29002166	-1.74690974	2.01311207
8	-1.30277836	-0.54150975	-0.20968795	6	4.58232164	-2.22970963	2.42051196
6	-2.59367824	-0.81750977	-0.18918794	1	5.37522173	-1.58140981	2.03911209
6	-3.20347834	-1.38490975	0.98171210	1	4.74852180	-3.25290966	2.07901192
6	-4.58997822	-1.56490982	0.99471211	1	4.57232141	-2.19600964	3.51021194
1	-5.07137823	-1.97240984	1.87771213	8	1.74202168	0.51119024	-1.72988796
6	-5.39067841	-1.25010979	-0.09848794	8	3.84102154	-0.31190974	-0.07748793

6	-4.78577852	-0.78040975	-1.25968790	6	2.84892154	0.34359026	-2.20928812
1	-5.41747856	-0.57960975	-2.11898804	6	4.09212160	-0.15790977	-1.47028792
6	-3.40647840	-0.57040977	-1.34758794	6	5.26842165	0.80039024	-1.61598790
6	-2.36577845	-1.84230971	2.19181204	1	4.34872150	-1.12800980	-1.91298795
6	-2.79007840	-0.10380976	-2.68028808	1	-6.46747828	-1.39650977	-0.05578794
6	-1.32717836	-2.89080954	1.74351203	8	3.11882162	0.56239027	-3.49328804
1	-0.65597838	-2.50510955	0.97661209	6	2.00532150	0.99909025	-4.30578756
1	-0.72047836	-3.21440959	2.59911203	1	2.41352177	1.11969030	-5.30738783
1	-1.83637834	-3.77280974	1.33841205	1	1.61422169	1.94399023	-3.92658806
6	-1.66717839	-0.65200979	2.87551188	1	1.21662164	0.24629024	-4.29348803
1	-1.01687837	-0.10970975	2.18871188	8	1.77232170	0.22499025	1.44401205
1	-2.41157842	0.04899025	3.27051187	6	2.11892176	0.91979021	2.58161187
1	-1.05117834	-0.99730980	3.71491194	6	2.10892153	2.43069029	2.41441202
6	-3.22477841	-2.51570964	3.27811193	8	0.75102162	2.97189045	2.31091189
1	-3.96557856	-1.83410978	3.71111202	1	2.68582153	2.72399044	1.53171206
1	-3.74977827	-3.39950967	2.90041208	15	0.08842164	3.26229024	0.89581209
1	-2.57397842	-2.84770966	4.09541225	8	-1.34837830	3.81209040	1.27231205
6	-3.82687855	-0.04190975	-3.81658792	8	0.12012164	2.13219023	-0.06808794
1	-4.30157852	-1.01220977	-3.99788785	8	0.81512165	4.53249025	0.27001208
1	-4.61387825	0.69569021	-3.62558794	1	3.94652176	-4.60081005	-1.00788796
1	-3.32537842	0.25689024	-4.74478769	1	5.52542162	0.93429023	-2.66778803
6	-2.20197845	1.31279027	-2.54118800	1	6.13002157	0.38509023	-1.08918786
1	-1.44327831	1.36849022	-1.76188791	1	5.02762175	1.77279019	-1.17938793
1	-1.75047827	1.63729024	-3.48858809	1	1.46462166	0.67069024	3.43571186
1	-2.99377847	2.02879024	-2.29108810	1	3.14712167	0.66829026	2.90121198
6	-1.70507836	-1.10020971	-3.13858795	1	2.52732158	2.92199039	3.29761195
1	-2.14507842	-2.08880973	-3.31108809	6	0.80462164	5.79749012	0.95781207
1	-1.25827837	-0.76510978	-4.08458757	6	-2.41547847	2.89459038	1.62671208
1	-0.91647840	-1.21110976	-2.39378810	1	-3.33447838	3.47609043	1.57271206
8	1.54832160	-1.73920977	-0.07208794	1	-2.25787830	2.53049040	2.64281201
15	2.93332171	-1.53810978	0.47121206	1	-2.45607829	2.06219029	0.92241210
8	3.83122158	-2.73170972	-0.21558794	1	1.44572163	6.46079016	0.37791207
6	3.22722173	-4.01490974	-0.43328795	1	1.20132160	5.69149017	1.97101212
1	3.02622175	-4.52020979	0.51761210	1	-0.21127835	6.19588995	0.99351209
1	2.29222155	-3.91160941	-0.98758793				

I-4_L_PP

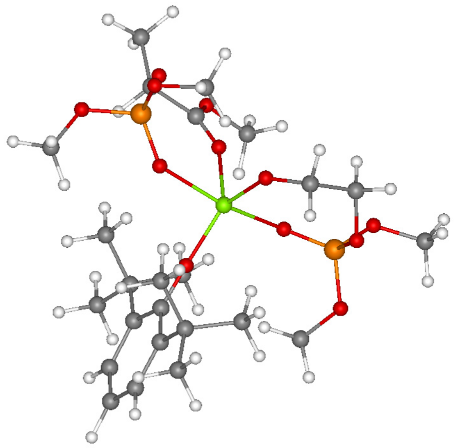

Zero-point vibrational energy	1804531.9 (Joules/Mol)
	431.29349 (Kcal/Mol)
Zero-point correction=	0.687310 (Hartree/Particle)
Thermal correction to Energy=	0.736113
Thermal correction to Enthalpy=	0.737057
Thermal correction to Gibbs Free Energy=	0.601785
Sum of electronic and zero-point Energies=	-2725.452142
Sum of electronic and thermal Energies=	-2725.403339
Sum of electronic and thermal Enthalpies=	-2725.402395
Sum of electronic and thermal Free Energies=	-2725.537668

cartesian

12	-0.38796747	-0.72640973	0.54118788	8	-4.01126766	-0.06290971	2.70028782
8	0.65093255	0.79829031	0.02078790	6	-3.39076734	-1.18350971	3.39798784
6	1.40003252	1.85789025	-0.20511210	1	-2.35816741	-1.33740962	3.06038785
6	2.23993254	2.40469027	0.82638788	1	-3.99876738	-2.07030964	3.20798779
6	3.07333255	3.48229027	0.51288790	1	-3.42436743	-0.92510974	4.45608807
1	3.72493267	3.89609027	1.27548790	8	-1.87836754	-1.46190965	-0.93021208
6	3.10073256	4.06059027	-0.75241208	8	-4.24816751	-0.62060970	0.28228790
6	2.24873257	3.56549025	-1.73391211	6	-2.89836740	-1.34080970	-1.58281219
1	2.26033258	4.04269028	-2.70851207	6	-4.10356760	-0.50570971	-1.14301217
6	1.39043248	2.48789024	-1.49781215	6	-5.42566776	-0.90010971	-1.77581215
6	2.21833253	1.85359037	2.26518774	1	-3.85996747	0.53089029	-1.40981221
6	0.44243255	2.00979018	-2.61311221	1	3.76353264	4.89619064	-0.96451205
6	0.80223250	1.99449027	2.85988784	8	-3.06526732	-1.89360964	-2.77691221
1	0.04893254	1.50049031	2.24638796	6	-1.93456745	-2.64400983	-3.28341222
1	0.76393259	1.56149030	3.86728787	1	-2.24276733	-2.98920989	-4.26831198
1	0.53263259	3.05439019	2.93768787	1	-1.72206748	-3.48400974	-2.62131214
6	2.67453265	0.38279027	2.30448794	1	-1.05716741	-2.00090981	-3.34591222

1	2.02433252	-0.26020971	1.71198785	8	-0.55206746	-1.83610964	2.08548784
1	3.69533253	0.28689027	1.91738784	6	0.09333253	-2.75250983	2.87188792
1	2.66963267	0.00529030	3.33418775	6	0.74723256	-3.90190983	2.11448789
6	3.16533256	2.62369013	3.20368791	8	1.95353258	-3.46450973	1.40668786
1	4.21393251	2.54339027	2.89658785	1	0.04343253	-4.34320974	1.40078783
1	2.90723252	3.68579030	3.27358794	15	1.89873254	-3.03970981	-0.11501210
1	3.08913255	2.20309019	4.21308804	8	3.39353251	-2.55710983	-0.33981210
6	0.55203259	2.86239028	-3.88961220	8	0.83893251	-2.08250976	-0.52821207
1	0.31283250	3.91569018	-3.70821214	8	1.72843254	-4.38480949	-0.97051209
1	1.54893255	2.80939031	-4.34031200	1	-4.24776745	3.79259038	0.98168790
1	-0.16076747	2.48919034	-4.63471174	1	-5.37286758	-0.80860972	-2.86171222
6	0.77123249	0.56139028	-3.02401209	1	-6.20836782	-0.23820971	-1.40001214
1	0.70463252	-0.12720971	-2.18131208	1	-5.68486738	-1.93020964	-1.52121210
1	0.08603254	0.22599031	-3.81541204	1	0.87803245	-2.29950976	3.50768781
1	1.78863251	0.50719029	-3.42901206	1	-0.60706747	-3.25490975	3.57468796
6	-1.02146745	2.11659026	-2.13811207	1	1.10273254	-4.67660952	2.80018783
1	-1.28306746	3.16509032	-1.95491219	6	2.68163252	-5.45170975	-0.82371211
1	-1.70306754	1.73349035	-2.91111207	6	3.67973256	-1.34310961	-1.08001220
1	-1.17756748	1.56379032	-1.21041214	1	3.59763265	-1.53980970	-2.15111208
8	-2.03396749	0.48629028	1.09228790	1	4.70493221	-1.07930970	-0.82451212
15	-3.50476742	0.39079028	1.27998781	1	2.99973273	-0.54220968	-0.78981209
8	-4.26746750	1.75809038	1.01208782	1	2.35123253	-6.24880934	-1.48931217
6	-3.68266749	3.00029016	1.47058785	1	2.69943261	-5.81480932	0.20728791
1	-3.79136729	3.07969022	2.55438781	1	3.67973256	-5.11460972	-1.11431217
1	-2.63076735	3.05269027	1.18588781				

S2. Polymerization experiments

The common reaction protocol of the polymerization experiments was presented in the section 4.3 of the manuscript. NMR spectra of the reaction mixtures and separated polymer samples are given below.

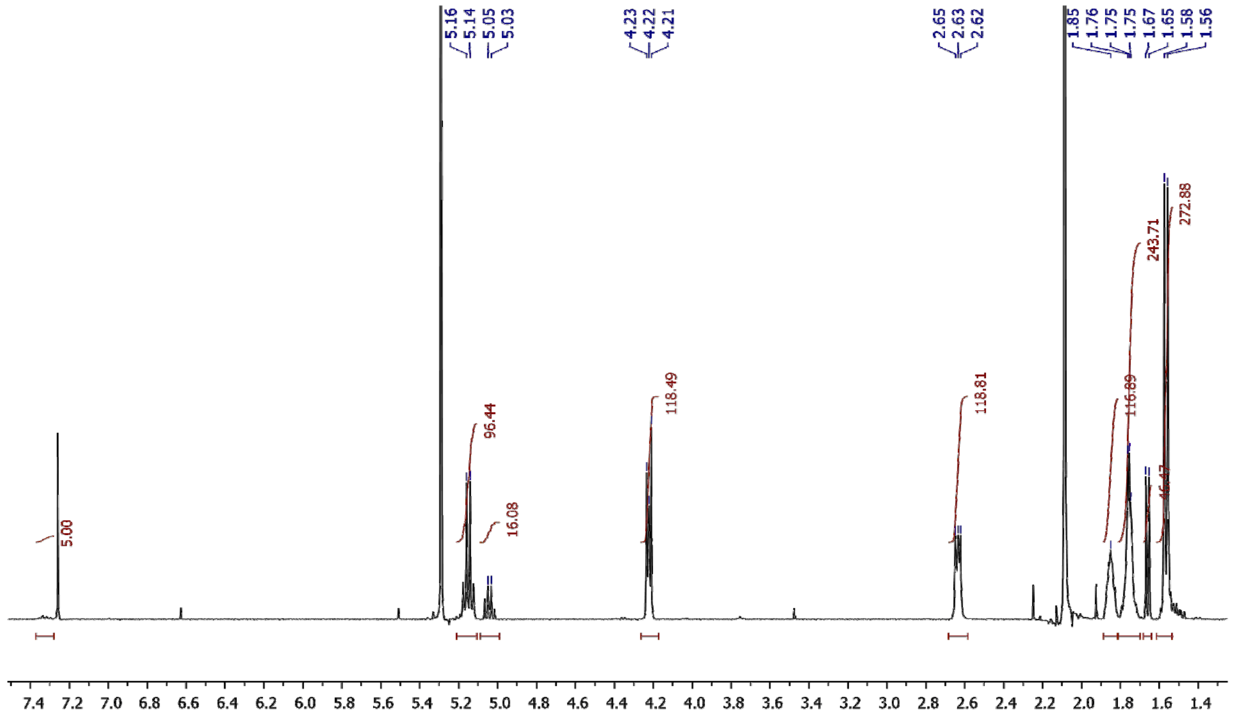

Figure S1. ^{1}H NMR spectrum of the probe taken from ε-CL/*L*-LA reaction mixtire after 10 min (Table 3, rin 1a).

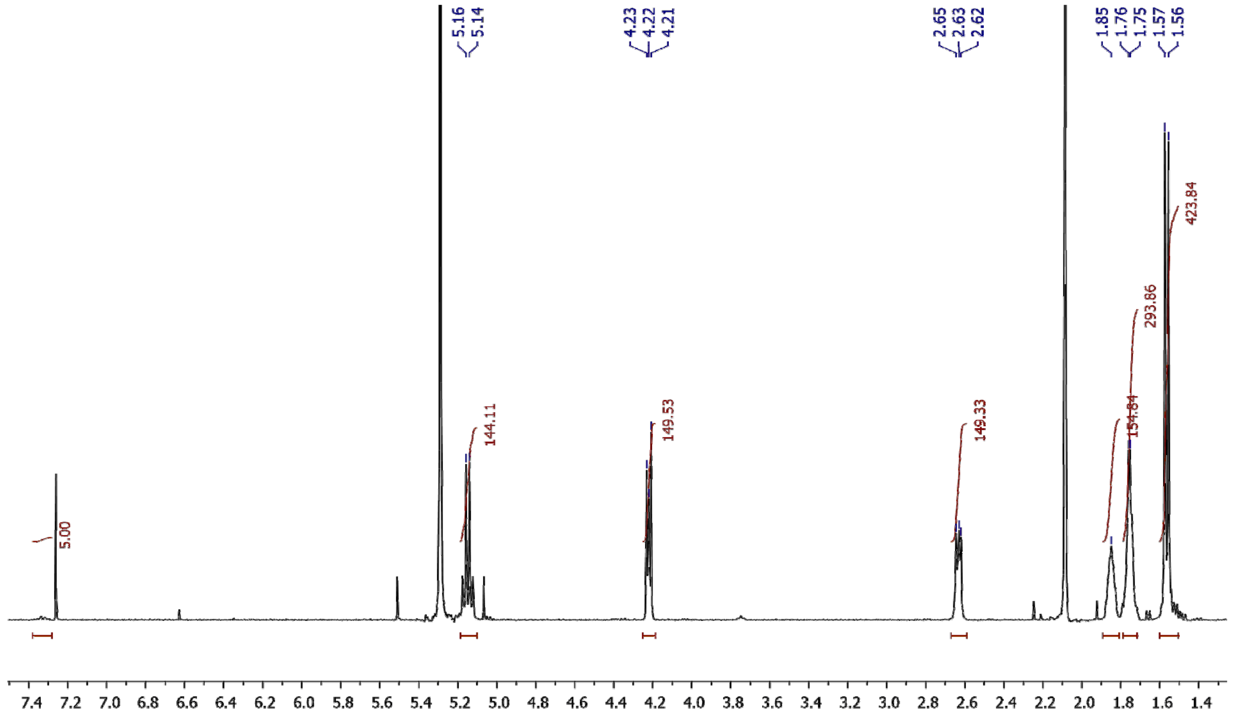

Figure S2. ^{1}H NMR spectrum of the probe taken from ε-CL/*L*-LA reaction mixtire after 30 h (Table 3, rin 1b).

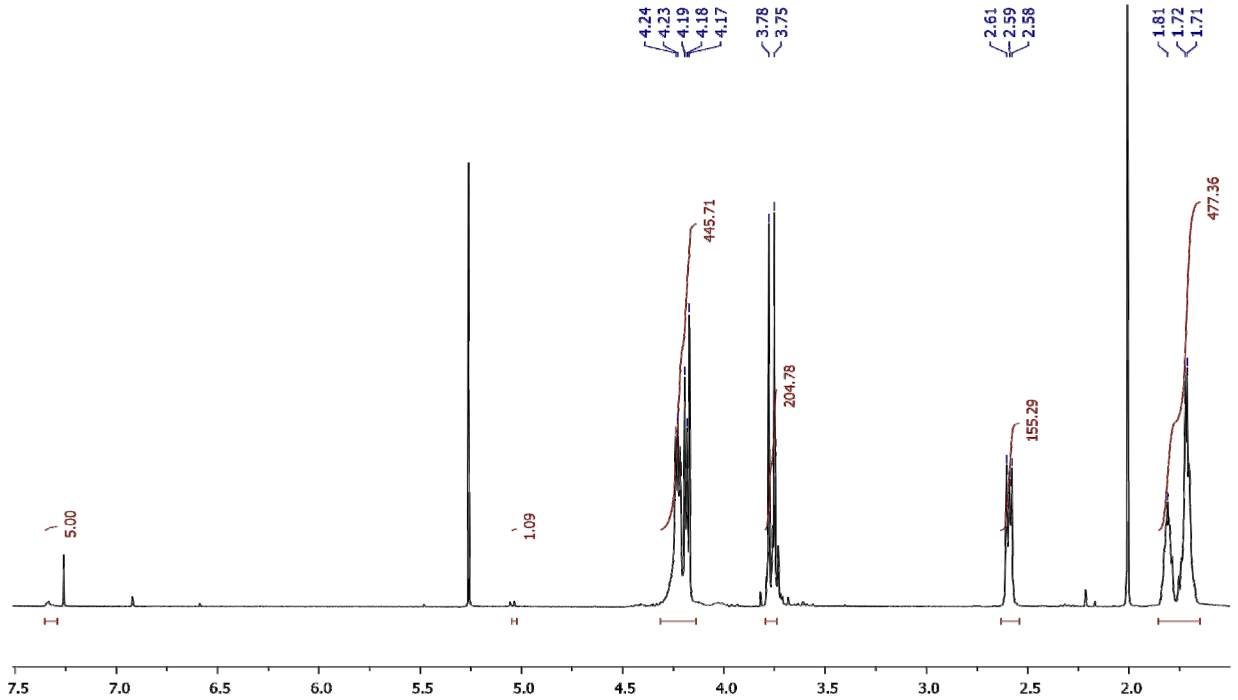

Figure S3. ^{1}H NMR spectrum of the probe taken from ε-CL/MeOEP reaction mixtire after 10 min (Table 3, rin 2a).

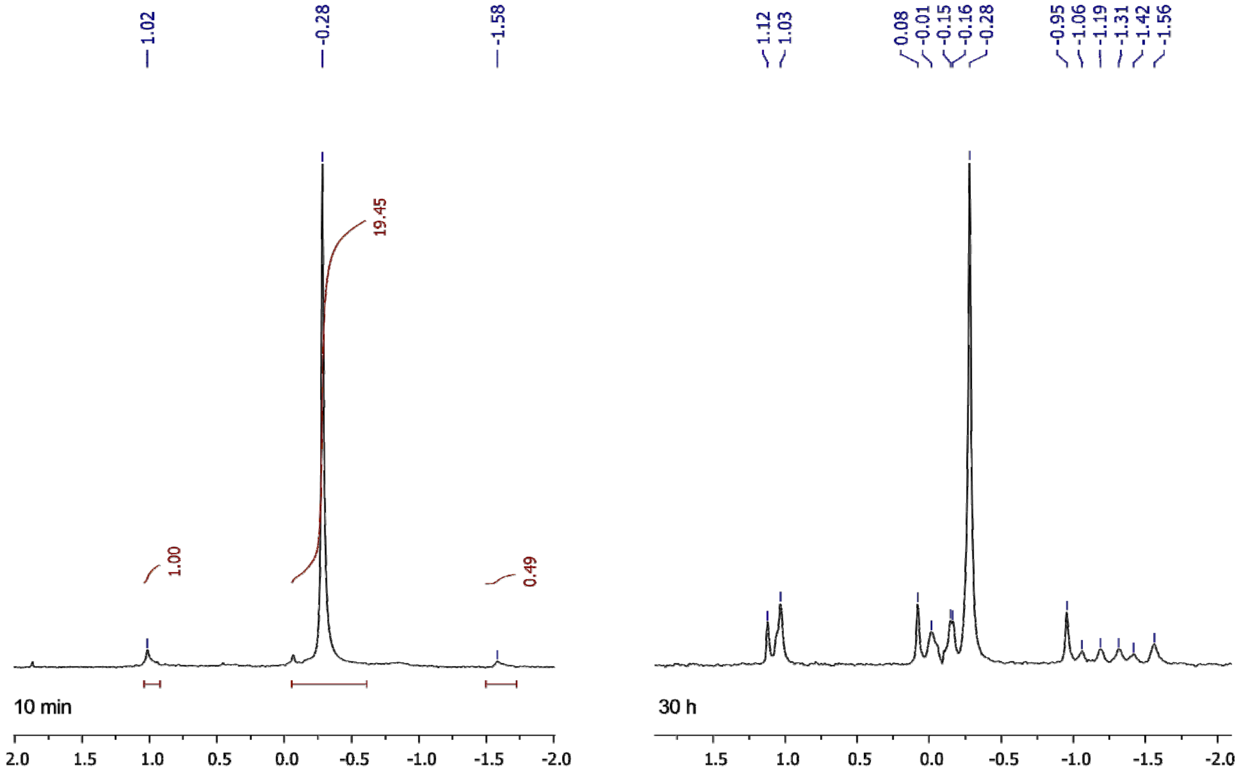

Figure S4. ^{31}P {^{1}H} NMR spectra of the probes taken from ε-CL/L-LA reaction mixtire after 10 min and 30 h (Table 3, rins 1a and 1b, respectively).
