# Supplementary figures and images for: DFT Visualization and Experimental Evidence of BHT-Mg-Catalyzed Copolymerization of Lactides, Lactones and Ethylene Phosphates
Source: Polymers (Basel). 2019 Oct 10;11(10):1641. doi: 10.3390/polym11101641 (PMC6836241; doi:10.3390/polym11101641)

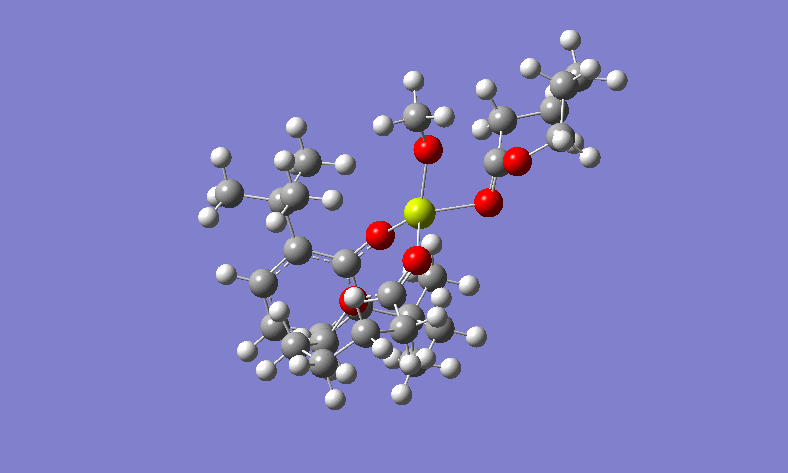

Supplement: Supplementary file 1 [file polymers-11-01641-s001.zip › 4-polymers-605538-SI-proof done/SI_Animations/TS-12_CC.gif]

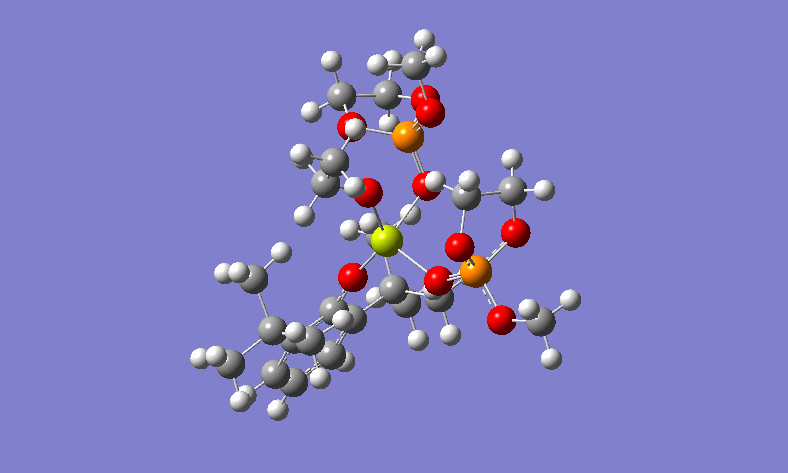

Supplement: Supplementary file 1 [file polymers-11-01641-s001.zip › 4-polymers-605538-SI-proof done/SI_Animations/TS-12_CP.gif]

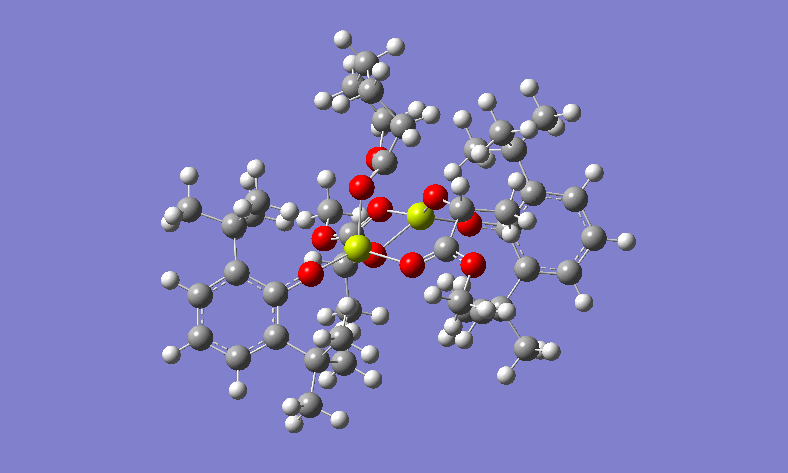

Supplement: Supplementary file 1 [file polymers-11-01641-s001.zip › 4-polymers-605538-SI-proof done/SI_Animations/TS-12_LC.gif]

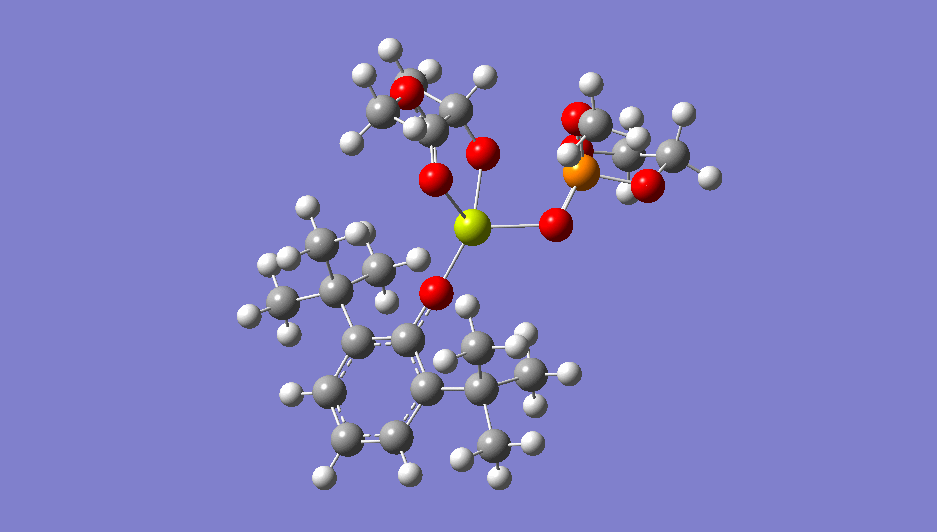

Supplement: Supplementary file 1 [file polymers-11-01641-s001.zip › 4-polymers-605538-SI-proof done/SI_Animations/TS-12_LP.gif]

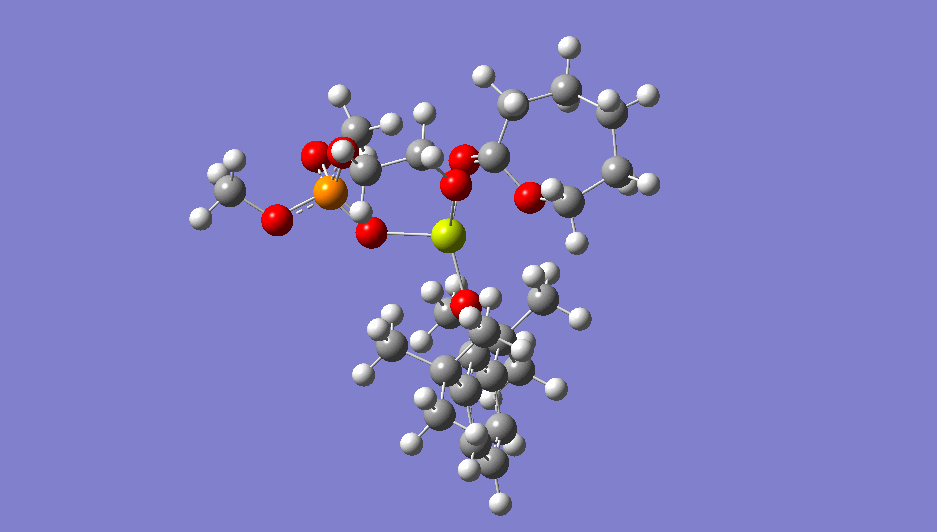

Supplement: Supplementary file 1 [file polymers-11-01641-s001.zip › 4-polymers-605538-SI-proof done/SI_Animations/TS-12_PC.gif]

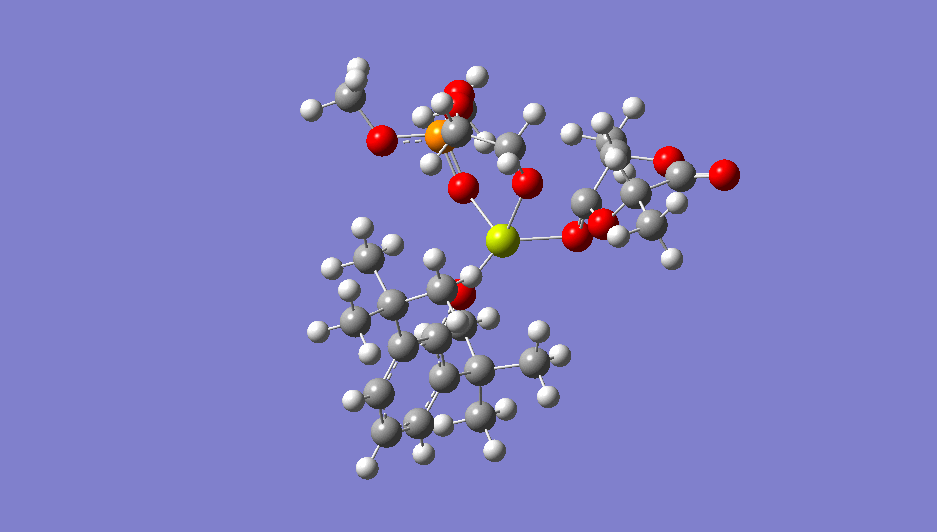

Supplement: Supplementary file 1 [file polymers-11-01641-s001.zip › 4-polymers-605538-SI-proof done/SI_Animations/TS-12_PL.gif]

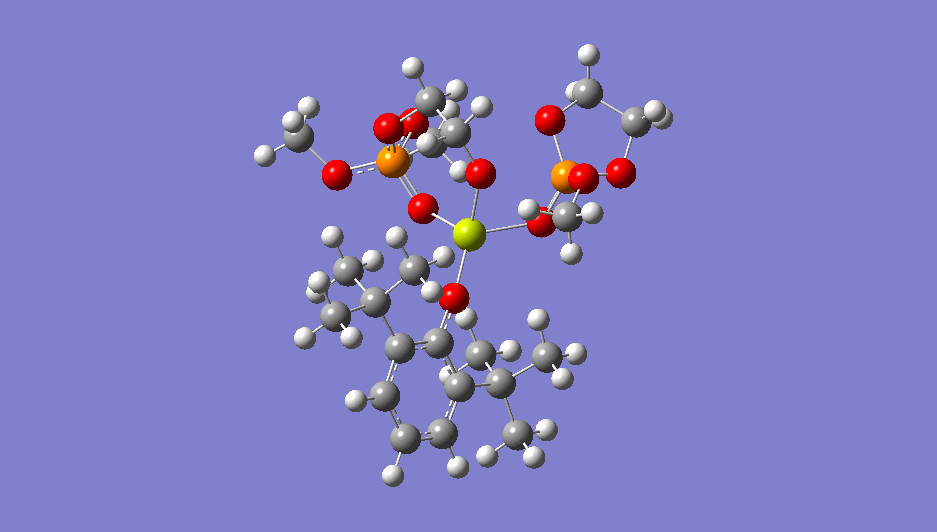

Supplement: Supplementary file 1 [file polymers-11-01641-s001.zip › 4-polymers-605538-SI-proof done/SI_Animations/TS-12_PP.gif]

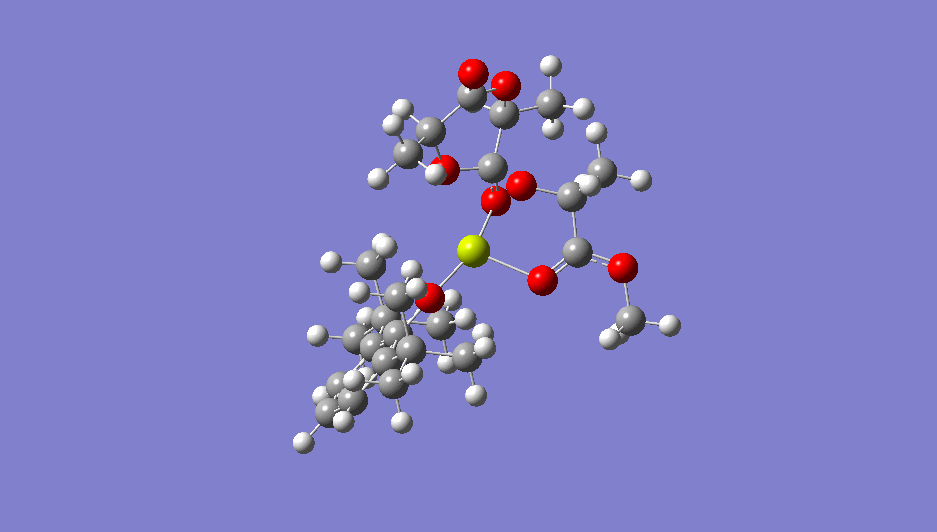

Supplement: Supplementary file 1 [file polymers-11-01641-s001.zip › 4-polymers-605538-SI-proof done/SI_Animations/TS-13_LL.gif]

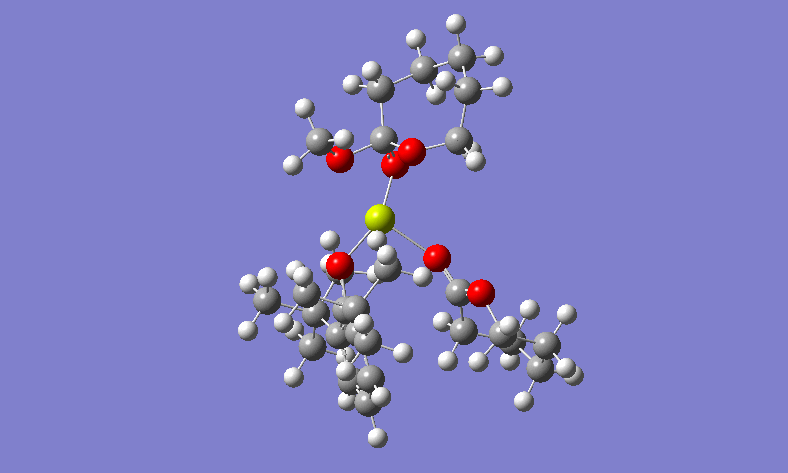

Supplement: Supplementary file 1 [file polymers-11-01641-s001.zip › 4-polymers-605538-SI-proof done/SI_Animations/TS-23_CC.gif]

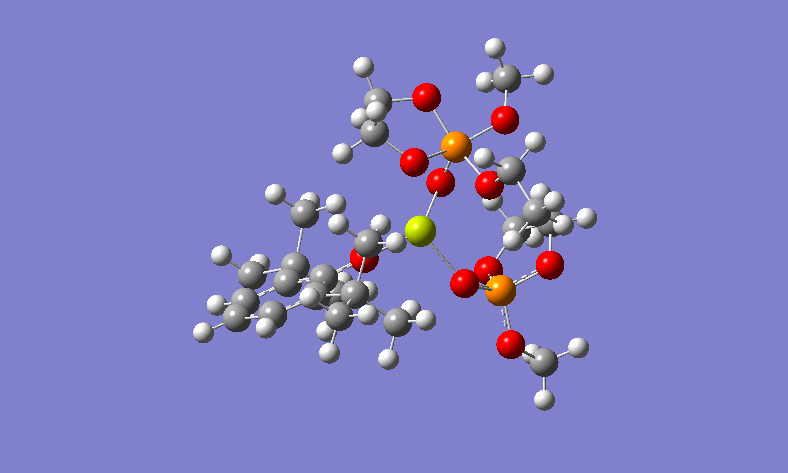

Supplement: Supplementary file 1 [file polymers-11-01641-s001.zip › 4-polymers-605538-SI-proof done/SI_Animations/TS-23_CP.gif]

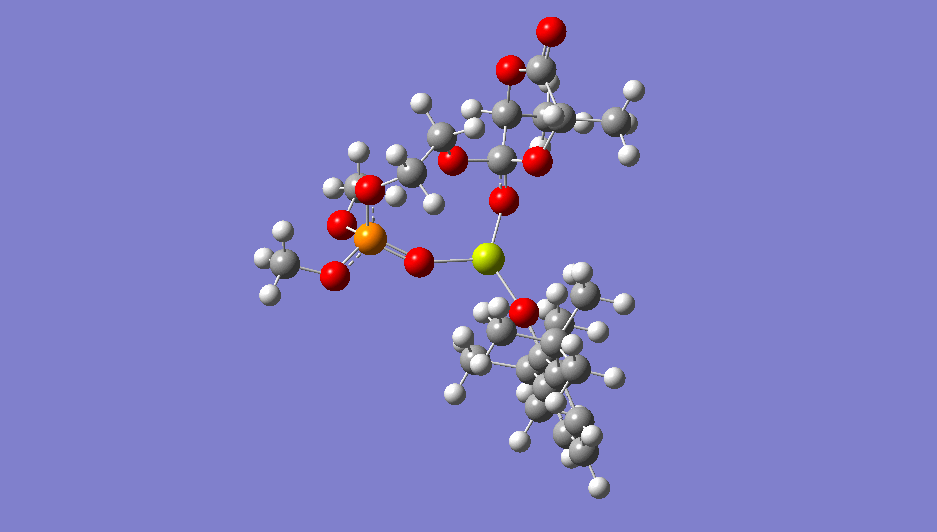

Supplement: Supplementary file 1 [file polymers-11-01641-s001.zip › 4-polymers-605538-SI-proof done/SI_Animations/TS-23_PL.gif]

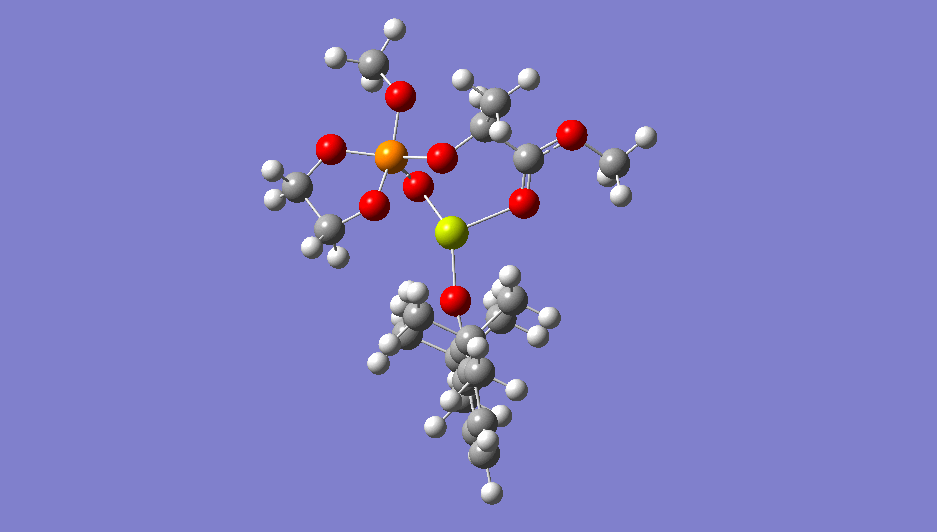

Supplement: Supplementary file 1 [file polymers-11-01641-s001.zip › 4-polymers-605538-SI-proof done/SI_Animations/TS-24_LP.gif]

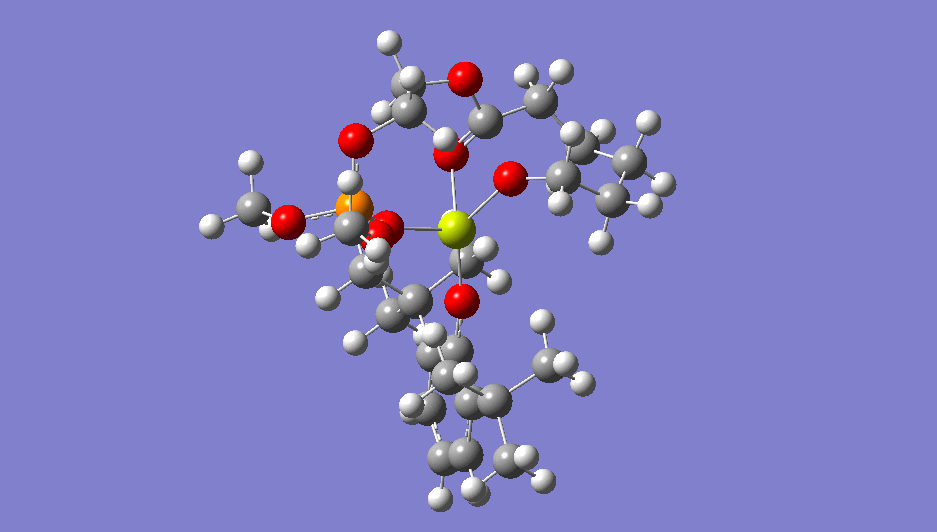

Supplement: Supplementary file 1 [file polymers-11-01641-s001.zip › 4-polymers-605538-SI-proof done/SI_Animations/TS-24_PC.gif]

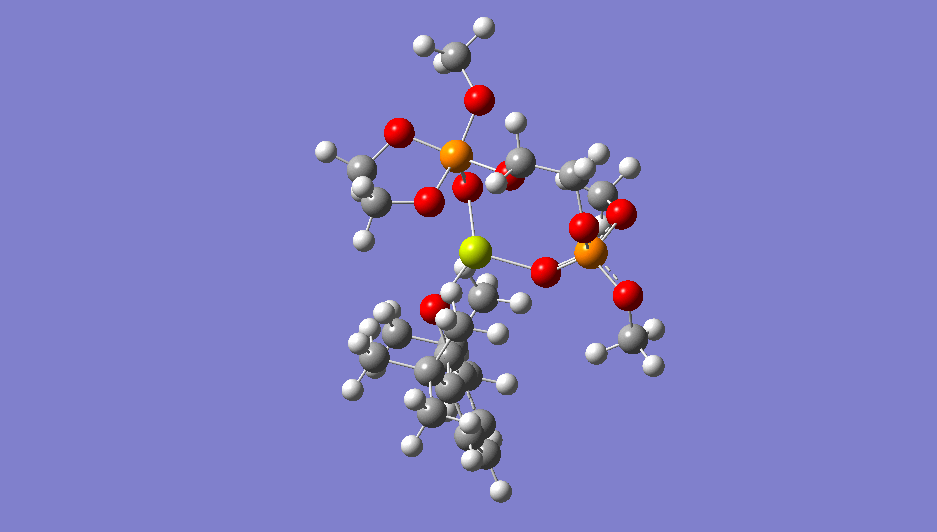

Supplement: Supplementary file 1 [file polymers-11-01641-s001.zip › 4-polymers-605538-SI-proof done/SI_Animations/TS-24_PP.gif]

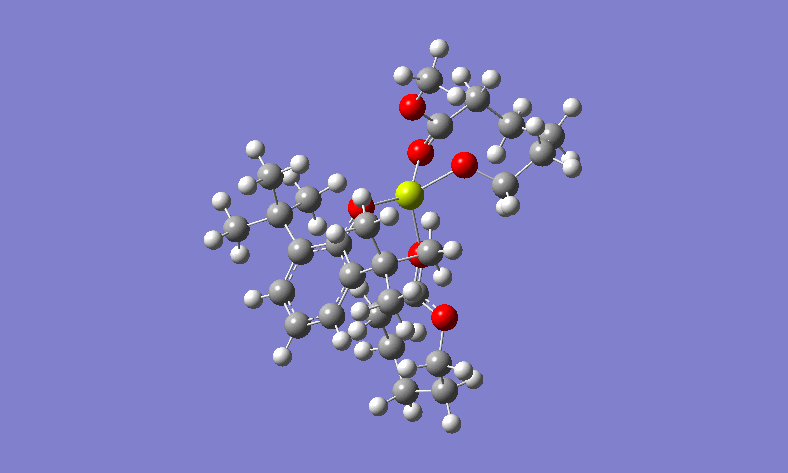

Supplement: Supplementary file 1 [file polymers-11-01641-s001.zip › 4-polymers-605538-SI-proof done/SI_Animations/TS-34_CC.gif]

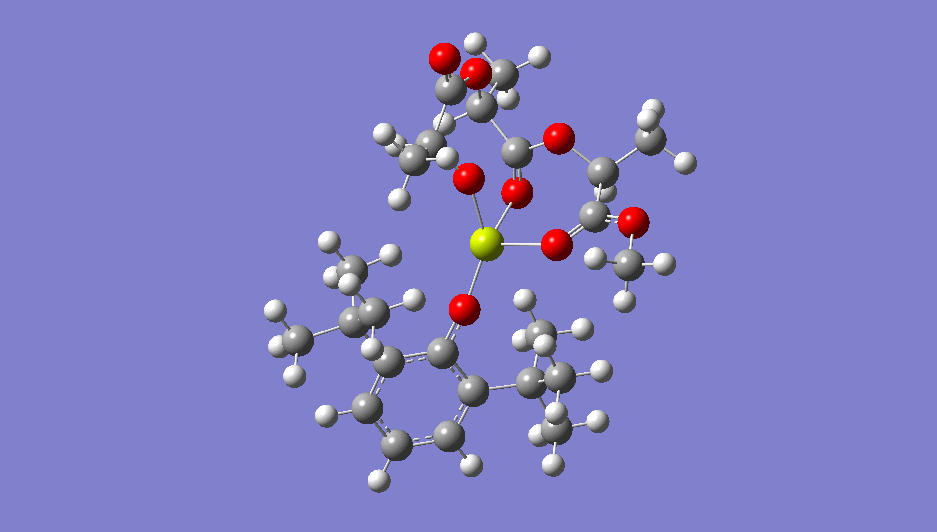

Supplement: Supplementary file 1 [file polymers-11-01641-s001.zip › 4-polymers-605538-SI-proof done/SI_Animations/TS-34_LL.gif]

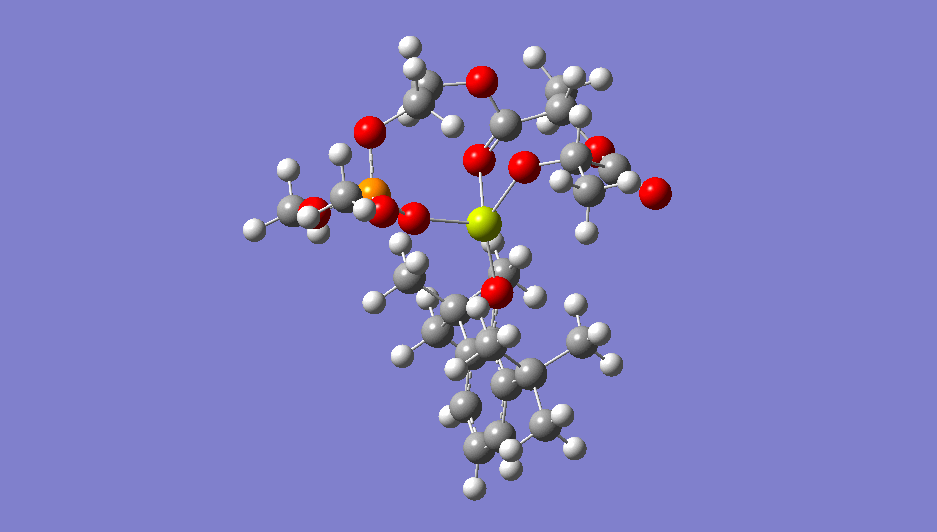

Supplement: Supplementary file 1 [file polymers-11-01641-s001.zip › 4-polymers-605538-SI-proof done/SI_Animations/TS-34_PL.gif]

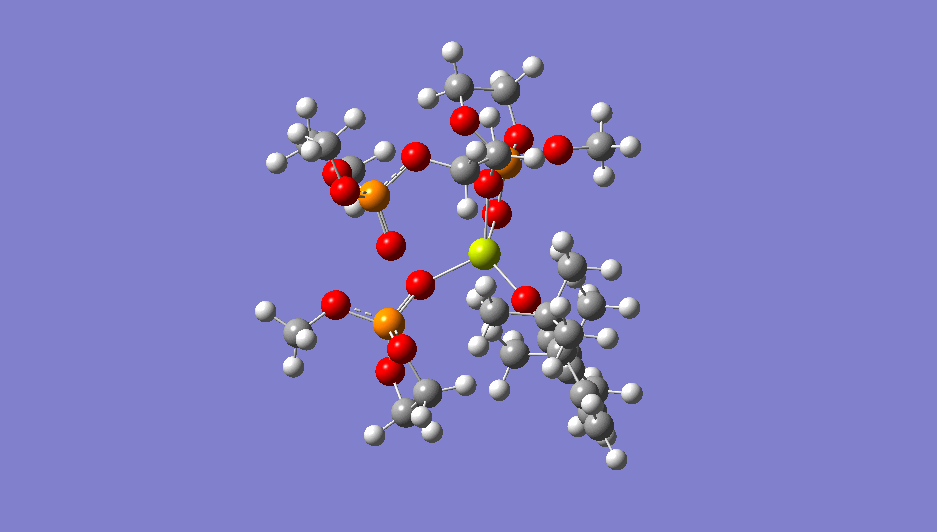

Supplement: Supplementary file 1 [file polymers-11-01641-s001.zip › 4-polymers-605538-SI-proof done/SI_Animations/TS-45_CP.gif]

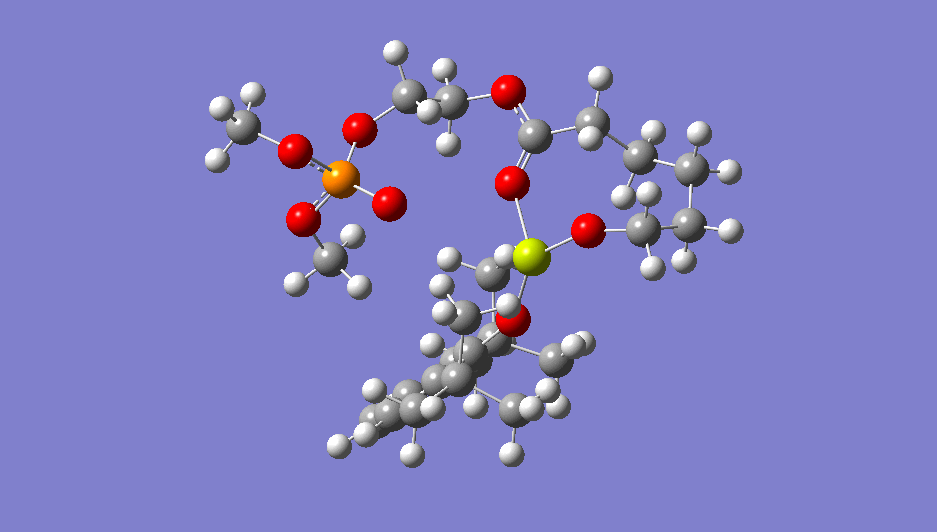

Supplement: Supplementary file 1 [file polymers-11-01641-s001.zip › 4-polymers-605538-SI-proof done/SI_Animations/TS-45_PC.gif]

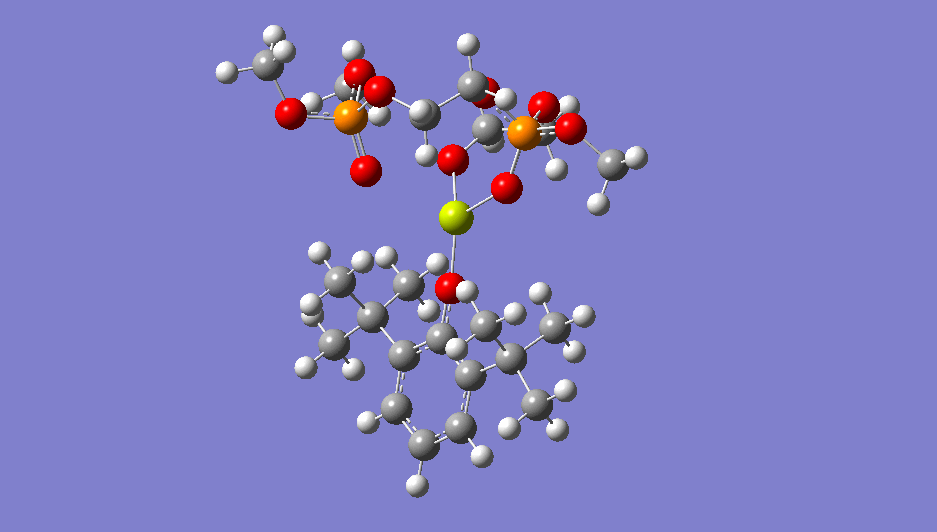

Supplement: Supplementary file 1 [file polymers-11-01641-s001.zip › 4-polymers-605538-SI-proof done/SI_Animations/TS-45_PP.gif]
